# Synthesis of Fused sp^3^‐Enriched Imidazoles

**DOI:** 10.1002/open.202400272

**Published:** 2024-11-13

**Authors:** Viacheslav Lysenko, Anton Portiankin, Tetiana Shvydenko, Svitlana Shishkina, Kostiantyn Nazarenko, Aleksandr Kostyuk

**Affiliations:** ^1^ Department of Organophosphorus Chemistry Institute of Organic Chemistry Academician Kukhar str. 5 02094 Kyiv-94 Ukraine; ^2^ SSI “Institute of Single Crystals” NAS of Ukraine Department of X-ray diffraction study and quantum chemistry 60 Nauky ave. 61001 Kharkiv; ^3^ Enamine Ltd. Winston Churchill Street 78 Kyiv, 02094 Ukraine

**Keywords:** Marckwald reaction, Neber rearrangement, Fused imidazoles, Aminoketones, Spinaceamine

## Abstract

A synthetic approach to imidazoles annulated to saturated six‐membered cycles featuring S, SO_2_, NH, NCbz was developed. It was achieved by combining the Neber rearrangement and the Marckwald reaction. The Neber rearrangement applied to cyclic ketones allowed us to prepare in hundred gram quantities previously unknown α‐amino ketones. Unstable tosyl ketoximes were used without purification immediately after their preparation. α‐Amino ketones react with potassium thiocyanate and cyanate in almost a quantitative yield affording highly pure imidazol‐2‐thiones or imidazol‐2‐ones. Desulfurization of imidazole‐2‐thiones using Raney nickel led to the formation of previously unknown 2‐unsubstituted fused imidazoles in high yields.

## Graphical abstract

Imidazoles annulated to saturated six‐membered cycles featuring S, SO_2_, NH, NCbz have been prepared in bulk quantities. The starting previously unknown cyclic α‐amino ketones were obtained via the Neber rearrangement. Unstable tosyl ketoximes were used without purification immediately after their preparation. Marckwald reaction with desulfurization using Raney nickel led to the formation of the annulated imidazoles.

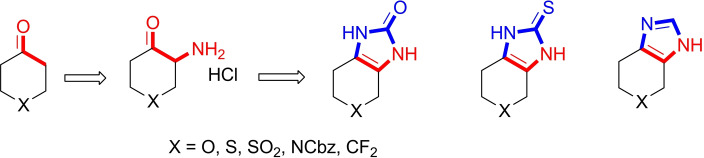

## Introduction

**Figure 1:**
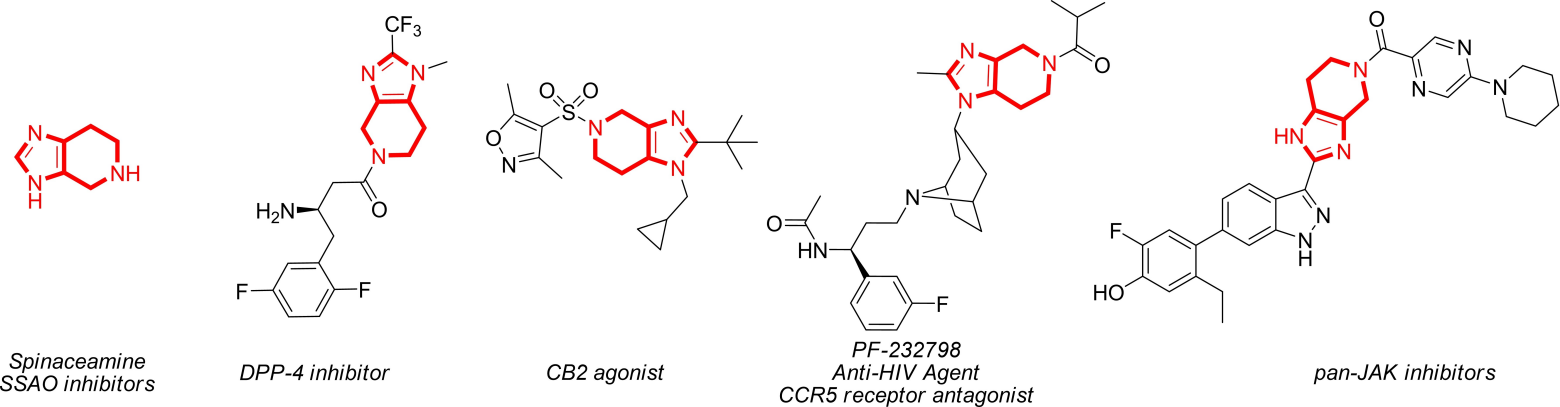
Bioactive compounds featuring partially saturated annulated imidazoles.

4,5,6,7‐Tetrahydro‐3H‐imidazo[4,5‐c]pyridine (spinaceamine), an aza analogue of tetrahydrobenzimidazole, is an alkaloid isolated from the skin of amphibians (Figure 1).[^1^, ^2^] Its various derivatives exhibit high potency as biologically active compounds. Specifically, 4‐substituted derivatives have been identified as activators of human carbonic anhydrases, including the cytosolic hCA I, II, and VII, as well as the membrane‐associated hCA IV.[^3^, ^4^, ^5^, ^6^, ^7^] Furthermore, the tetrahydroimidazopyridine scaffold found in numerous bioactive substances. These include pan‐JAK inhibitors,^[8]^ CCR5 antagonists used for HIV treatment,^[9]^ non‐hydrazine SSAO inhibitors,^[10]^ dipeptidyl peptidase IV inhibitors for managing diabetes,^[11]^ CB2 agonists,^[12]^ and more (Figure 1).

Imidazopyridin‐2‐ones have been investigated for their pharmaceutical applications as C5a receptor modulators^[13]^ and anticoagulant agents to treat and prevent thromboembolic disorders (factor Xa inhibitors).^[14]^ Hydroxy‐substituted 1H‐imidazopyridines can also serve as immunomodulators for treating various diseases, including viral and neoplastic conditions.^[15]^ Additionally, tetrahydroimidazopyridin‐2‐ones have been identified as autotaxin inhibitors.^[16]^ By leveraging the versatility and biological activity of its derivatives, medicinal chemists can explore their potential applications in developing novel pharmaceutical compounds with therapeutic benefits.

A common method for synthesizing annulated imidazoles involves cyclization of a saturated ring to the imidazole core. However, this method has primarily been applied to tetrahydroimidazopyridine derivatives. One example is the synthesis of partially saturated imidazopyridines, where the Pictet‐Spengler reaction plays a crucial role. This reaction involves the annulation of the tetrahydropyridine ring through the reaction of histamine with carbonyl compounds in the presence of an acid catalyst.[^17^, ^18^] Another strategy to obtain partially saturated fused imidazoles is through the annulation of the imidazole ring with a saturated fragment. This can be accomplished by reacting 2‐chloro or 2‐hydroxycyclohexanone with formamidine or formaldehyde in the presence of ammonia.[^19^, ^20^, ^21^] Tetrahydrobenzimidazoles can also be obtained by hydrogenation of benzimidazoles.[^22^, ^23^, ^24^, ^25^] Another approach is the reaction of α‐amino carbonyl compounds with iminoesters.^[26]^ It should be pointed out that there is a lack of methods in the literature for the synthesis of fused imidazole derivatives to the saturated heterocycles.

Further, the Marckwald reaction is worth mentioning as one of the effective methods for annulating an imidazole nucleus to saturated fragments. The method is based on the reaction of α‐amino carbonyl compounds (aminoketones or aminoacetals) with thiocyanates. The reaction results in imidazole‐2‐thiones, which can be further used for the synthesis of 2‐unsubstituted imidazoles by the subsequent desulfurization with Raney nickel, H_2_O_2_/AcOH or HNO_3_.[^27^, ^28^, ^29^, ^30^, ^31^] The method is synthetically simple, does not require harsh conditions, runs in a neutral or weakly acidic medium, and provides almost quantitative yields. Recently we have developed a convenient method for the synthesis of 6,7‐dihydro‐5H‐pyrrolo[1,2‐c]imidazole via reaction of commercially available *N*‐Boc protected prolinal with potassium thiocyanate.^[32]^ The Marckwald reaction represents a significant method for synthesizing sp^3^‐enriched imidazole derivatives. However, its practical application is constrained by the availability of the requisite starting α‐amino carbonyl compounds. In our opinion, a convenient method for the synthesis of cyclic α‐amino ketones required for the Marckwald reaction could be the Neber rearrangement. This is followed by the conversion of the O‐sulfonates of ketoximes into α‐amino ketones.[^33^, ^34^, ^35^, ^36^, ^37^] Although the Neber rearrangement has been extensively studied for acyclic structures, there is limited literature on its application to cyclic compounds,[^38^, ^39^] primarily focusing on benzannulated derivatives.[^40^, ^41^, ^42^, ^43^, ^44^, ^45^, ^46^, ^47^] Additionally, the utilization of cyclic amino ketones in the Marckwald reaction is well‐documented, particularly for 2‐aminocyclohexanone.^[48]^

Hence, by combining the Neber rearrangement and the Marckwald reaction, a straightforward synthetic approach for the synthesis of tetrahydrobenzimidazole derivatives and their heterocyclic analogues can be achieved. Notably, the literature lacks synthesis methods for fused imidazoles that incorporate heteroatoms other than nitrogen in the saturated fragment.

**Figure 2:**
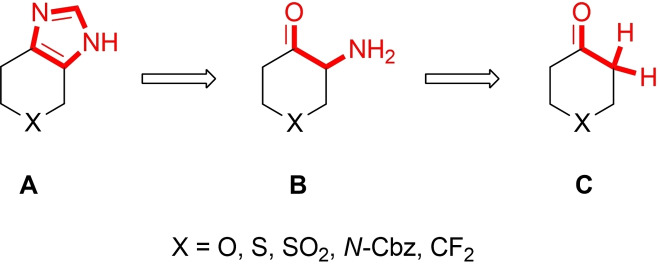
Retrosynthetic analysis to the fused sp^3^‐enriched imidazole derivatives.

Therefore, our objective was to develop a straightforward synthetic method for the production of tetrahydrobenzimidazole derivatives and their heterocyclic analogues, employing the sequential utilization of the Neber rearrangement and the Marckwald reaction. The retrosynthetic analysis of the proposed way to the fused sp^3^‐enriched imidazole derivatives is presented in Figure 2.

## Results and Discussion

**Scheme 1:**
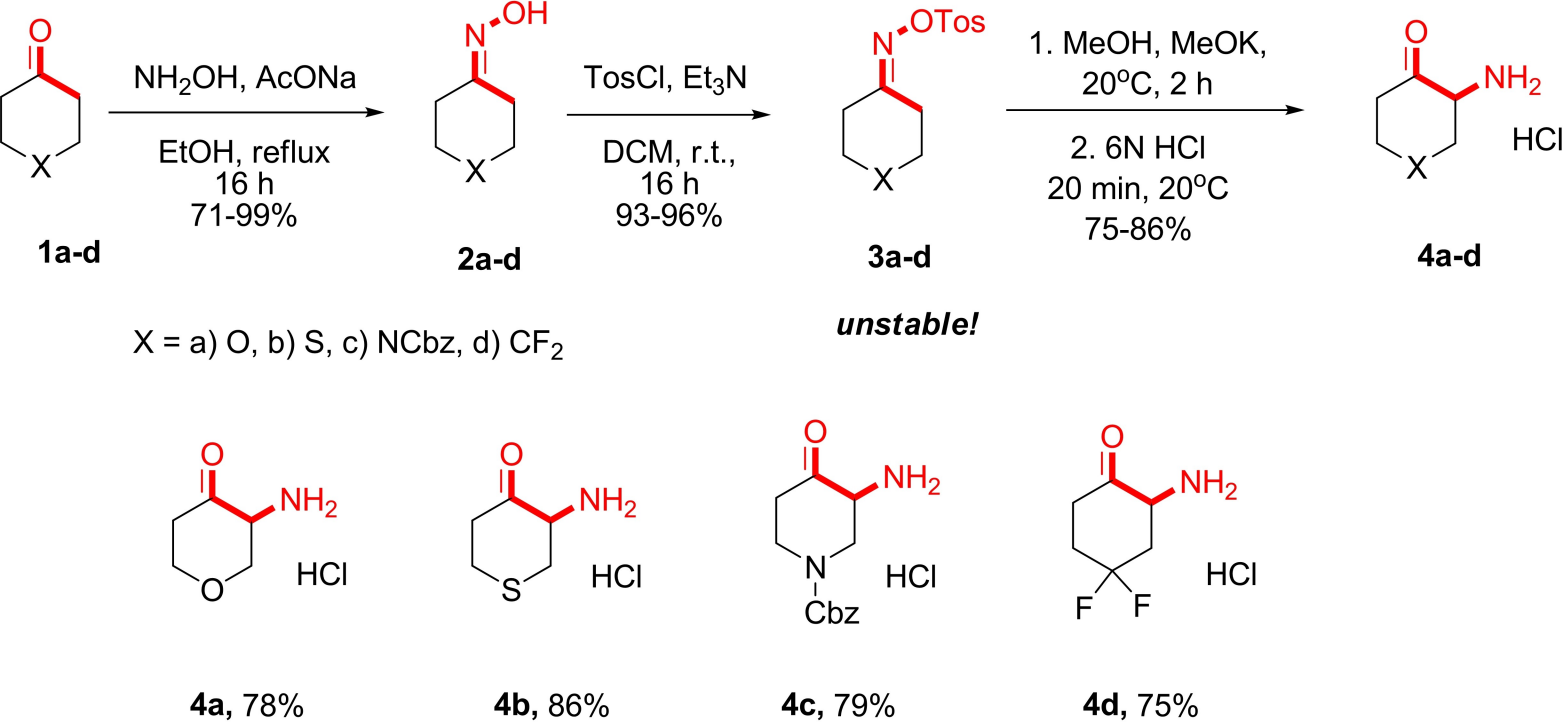
Synthesis of α‐amino ketones **4** via the Neber rearrangement.

In the initial phase of our research, we successfully obtained cyclic α‐amino ketones **4** via the Neber rearrangement. Notably, using our improved method, we were able to obtain previously unknown cyclic α‐amino ketones **4** as hydrochlorides in quantities exceeding 100 g. To synthesize the ketoximes **2**, we followed a known procedure involving the reaction of cyclic ketones **1** with hydroxylamine hydrochloride in ethanol in the presence of sodium acetate (Scheme 1). The resulting ketoximes **2** were then reacted with tosyl chloride in dichloromethane using 1.1 equivalents of triethylamine. It should be mentioned that the obtained ketoxime tosylates **3** are relatively unstable, with the rearrangement occurring spontaneously when the crude oxime tosylate is exposed to ambient environment for several days.^[37]^ The O‐Tos derivative of cyclopentanone ketoxime is particularly unstable. Due to their instability, all obtained O‐Tos ketoximes **3** were used without purification. Nevertheless, we were able to fully characterize these ketoximes with the exception of compound **3 c**. For the Neber rearrangement, the obtained tosylates were subjected to treatment with potassium methylate followed by a reaction with an aqueous hydrochloric acid at room temperature. The main practical improvements we made in the Neber rearrangement is that we used *t*‐BuOK in MeOH and the reaction mixture was filtered into a Büchner flask filled with concentrated HCl. These simple enhancements allowed for the isolation of α‐amino ketones **4** as hydrochlorides in quantities exceeding 100 g without any purification.

**Scheme 2:**
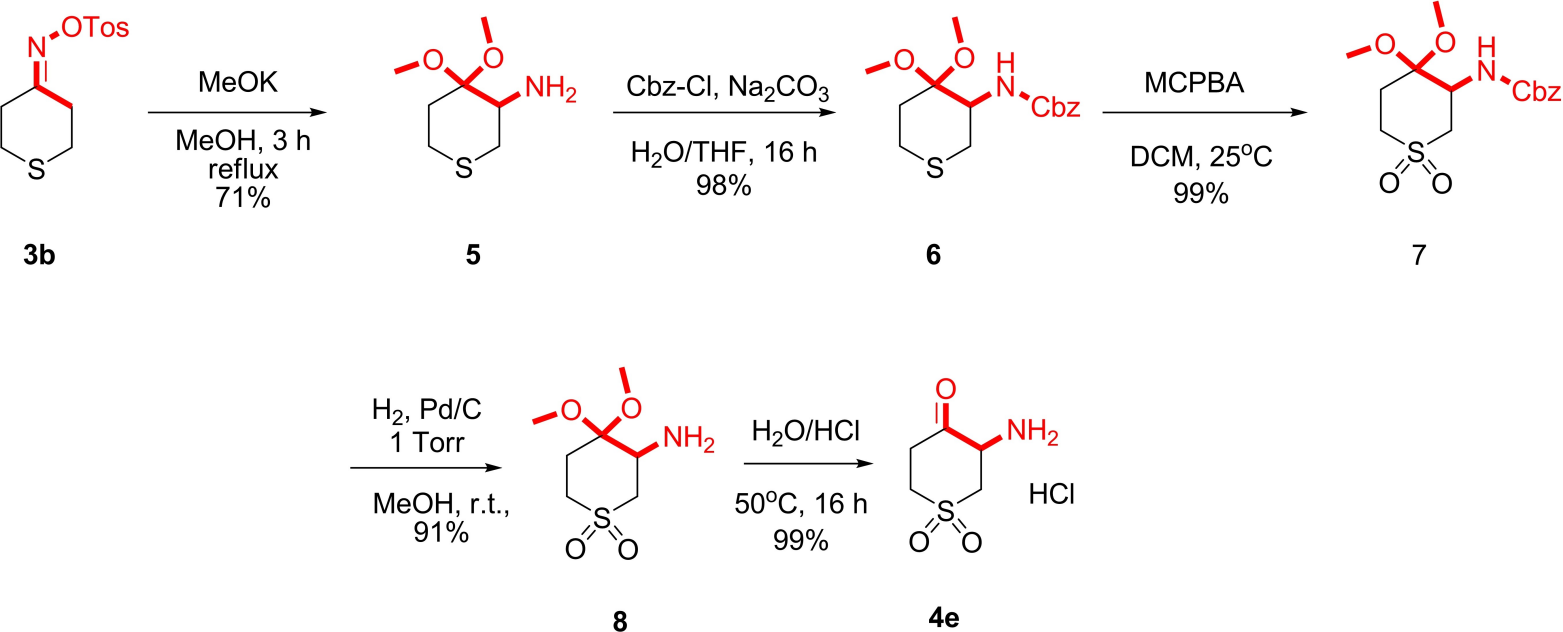
A modified approach to 3‐amino thiopyranone‐1,1‐dioxide derivative **4 e**.

Notably, while the thiopyran‐4‐one derivative (X=S) **1 b** showed good reactivity, the use of the dihydro‐2H‐thiopyran‐4(3H)‐one 1,1‐dioxide derivative in the Neber rearrangement resulted in the formation of an unidentified mixture of products. It is likely due to the higher acidity of the methylene groups in the α‐position of thiopyran‐4‐one‐1,1‐dioxide compared to the thiopyran‐4‐one derivative. In the synthesis of 3‐amino thiopyranone‐1,1‐dioxide derivative **4 e**, we employed a modified Neber rearrangement (Scheme 2). The tosyl ketoxime **3 b** was heated with methanol in the presence of potassium methylate, leading to the formation of acetal **5**. Since the oxidation reaction of thiopyran derivative **5** to thiopyran‐4‐one‐1,1‐dioxide **8** requires the use of oxidizing reagents (peroxy acids or hydrogen peroxide), we obtained *N*‐Cbz derivative **6**, which made it possible to carry out sulfur oxidation in a high yield using MCPBA. Subsequent Cbz deprotection and hydrolysis of the acetal afforded 3‐amino thiopyranone‐1,1‐dioxide **4 e** in a high yield.

It is worth noting that ^1^H NMR spectra of amino ketones featuring electron‐withdrawing substituents in the cycle **4 e**, **4 d** (such as SO_2_ and CF_2_) quite often exhibit both hydrate and ketone forms (Figure S1). For example, in the DMSO‐d_6_ solution (with traces of water), in ^1^H NMR spectra a mixture of ketone and hydrate forms was observed, with the keto form prevailing for the derivatives of thiopyranone‐1,1‐dioxide and 4,4‐difluorocyclohexanone. In the D_2_O solution ^1^H NMR spectra showed that these amino ketones exclusively exist in the hydrate form. Conversely, in the CF_3_COOD solution, we were able to obtain ^1^H NMR spectra of these amino ketones in the keto form.

**Scheme 3:**
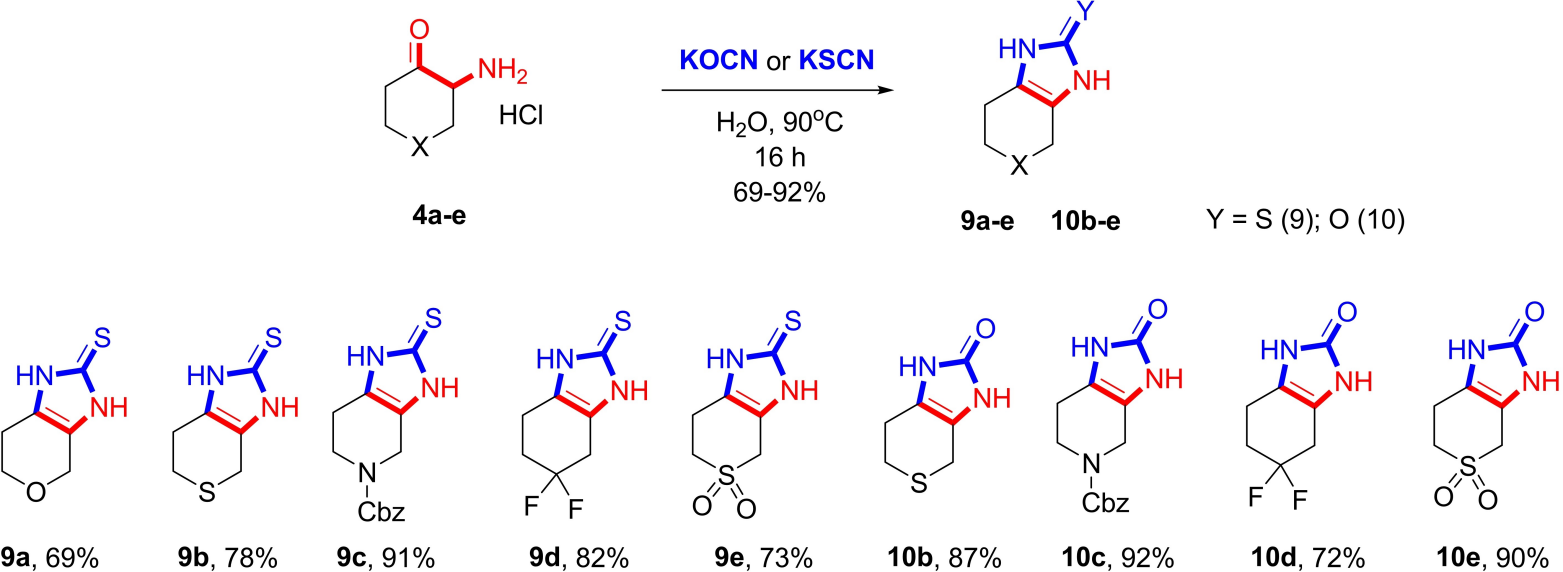
Marckwald approach to fused imidazoles.

The obtained amino ketones were then subjected to the conditions of the Marckwald reaction (Scheme 3), which resulted in the formation of imidazol‐2‐thiones **9 a–e** or imidazol‐2‐ones **10 b–e**. It should be noted that we failed to isolate compound **10 a**. It is probably due to its high solubility in water compared to the other imidazol‐2‐ones. Neither evaporation of the aqueous layer nor extraction delivered the product. Importantly, the Marckwald reaction provided these derivatives in almost quantitative yield without requiring purification. Similar to the amino ketones **4**, derivatives **9** and **10** were obtained in multigram quantities, thus providing a straightforward synthetic approach to sp^3^‐enriched imidazole derivatives, which hold promise in biomedical research.

**Scheme 4:**
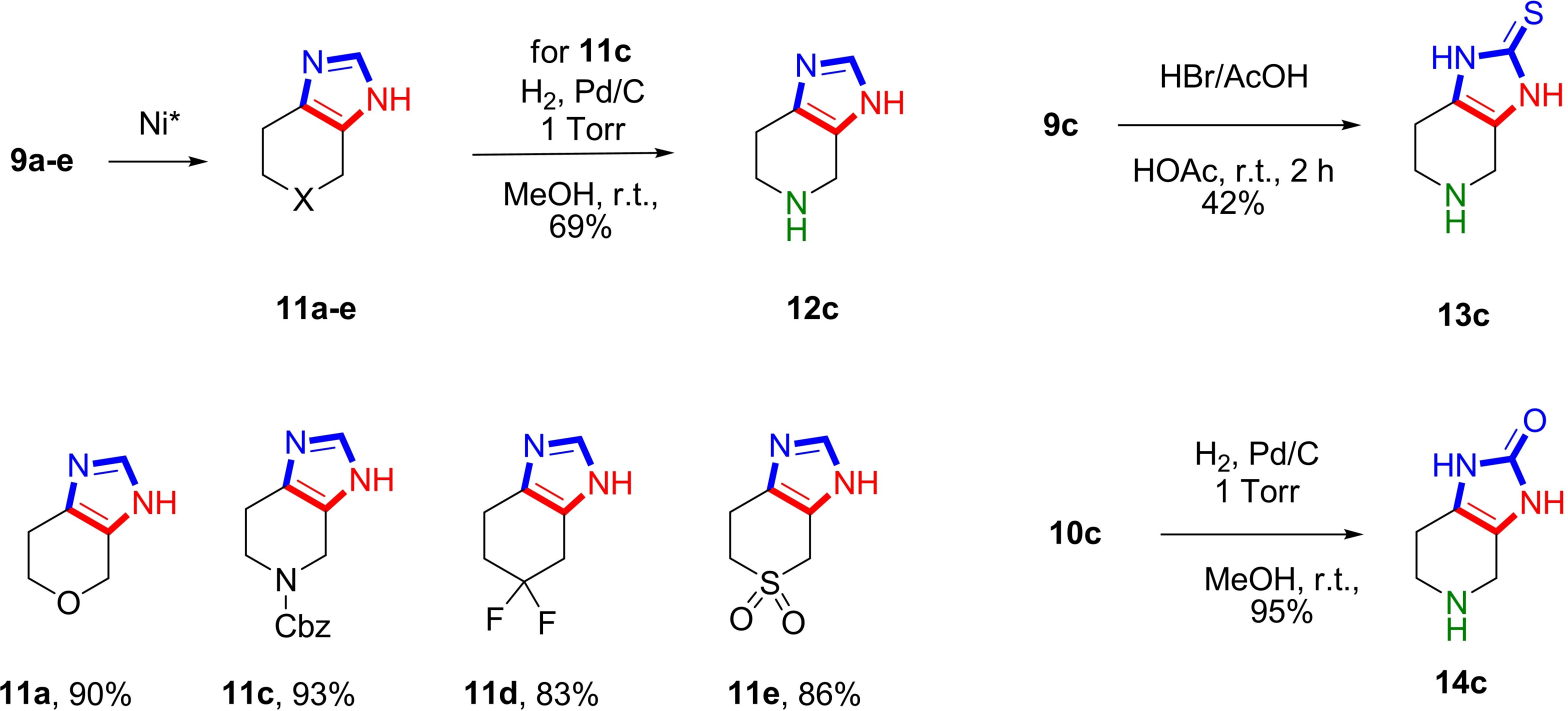
Synthesis of spinaceamine **12 c** and its analogues.

To explore further the functionalization of the resulting products **9**, we investigated the possibility of desulfurization of the derivatives **9** (Scheme 4). Desulfurization using Raney nickel led to the formation of previously unknown 2‐unsubstituted fused imidazoles **11** in high yields. Since compound **9 b** contains the tetrahydrothiopyrane nucleus that is sensitive to Raney nickel, we did not try to prepare compound **11 b**.

**Figure 3:**
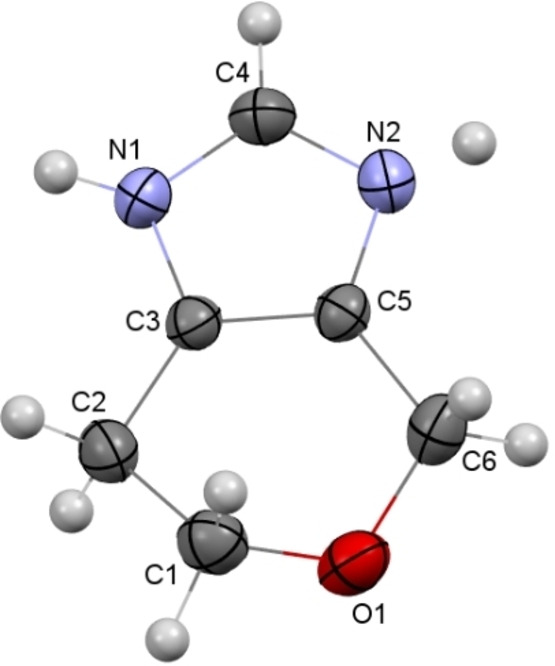
Molecular structure of compound **11 a** according to X‐ray diffraction data. Thermal ellipsoids of non‐hydrogen atoms are shown at 50 % probability level.

The molecular and crystal structure of compound **11 a** (Figure 3) was studied by the single crystal X‐ray diffraction method. The very close values of the C5−N2/C3−N1 (1.378(2) Å and 1.377(2) Å) and N2−C4/N1−C4 (1.337(2) Å and 1.334(2) Å) bond lengths as well as disordering of the hydrogen atom between two nitrogen atoms allows considering of the molecular structure of **11 a** as superposition of two tautomers. The tetrahydroheterocycle adopts a half‐chair conformation where the C2, C3, C5, C6 atoms lie in the plane with an accuracy of 0.003 Å and the C1 and O1 atoms deviate from this plane by 0.332(3) Å and −0.397(3) Å, respectively.

Notably, in the case of imidazol‐2‐thione **9 c** (X=N−Cbz), desulfurization with Raney nickel proceeded with the preservation of the Cbz protection affording fused imidazole **11 c**. The Cbz protection was subsequently removed using hydrogen on Pd/C. This approach enabled the synthesis of spinacine derivative **12 c** in moderate yield through a novel method. In contrast, for derivative **9 c** (X=N−Cbz), Cbz deprotection was achieved using HBr in acetic acid, which preserved the thione group and provided a simple route to the synthesis of the previously unknown 2‐thio derivative of spinacine **13 c**. In addition, 2‐oxo derivative of spinacine **14 c** was obtained via N‐Cbz deprotection of **10 c** using hydrogen on Pd/C (Scheme 4).

**Scheme 5:**
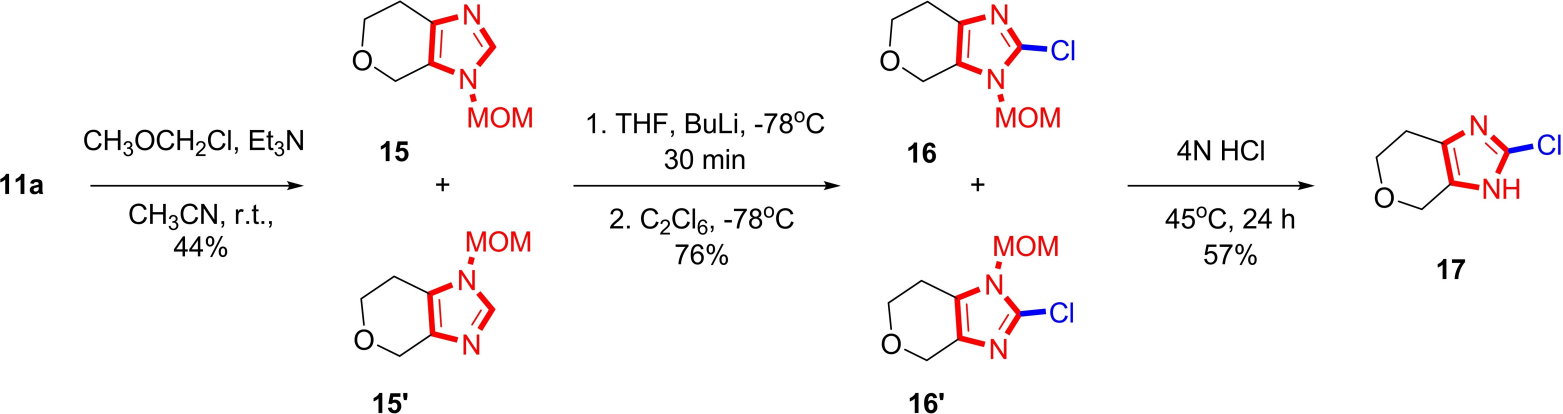
Halogenation of 2‐unsubstituted imidazole **11 a**.

It should be noted that our previous attempts to obtain 2‐halo‐substituted imidazole **16** using NCS or NBS were unsuccessful. Likewise, our efforts to obtain derivatives of type **17** by heating imidazole‐2‐thione **9 a** with SOCl_2_ or POCl_3_ were also unsuccessful. Therefore, we employed a protective group strategy for the imidazole nucleus. Initially, we obtained derivatives **15** through alkylation of the starting **11 a** with MOM‐chloride in the presence of a base. The alkylation reaction did not occur selectively, leading to the formation of two regioisomers in equal ratios. By lithiating the resulting mixture with BuLi at −78 °C, followed by reaction with C_2_Cl_6_, we obtained a mixture of regioisomers of 2‐chloro‐imidazole derivatives **16**. The removal of the protective group with hydrochloric acid facilitated a straightforward synthesis of 2‐chloroimidazole derivative **17** (Scheme 5).

## Conclusions

In conclusion, we have developed a method for the synthesis of previously unknown cyclic α‐amino ketones in bulk quantities via the Neber rearrangement. By combining the Neber rearrangement and the Marckwald reaction, we established a route to the synthesis of tetrahydrobenzimidazole derivatives and their heterocyclic analogues. Furthermore, the potential for further functionalization of these imidazole derivatives was investigated. The α‐amino ketones featuring electron‐withdrawing substituents in the cycle exist in equilibrium with their hydrates that was demonstrated by ^1^H NMR spectra.

## General Procedure for the Neber Rearrangement

To a solution of potassium *tert*‐butoxide (110.0 g, 0.98 mol, 1.15 eq.) in absolute methanol (1.2 L) was added *O*‐tosyl oxime **3 a** (230.0 g, 0.85 mol, 1 eq.) portion wise at 10 °C. The resulting mixture was stirred for 3 h at room temperature. Concentrated HCl (160 mL) was poured in a Büchner flask and the reaction mixture was filtered into it. The filtrate with concentrated HCl was stirred at room temperature for 15 minutes and evaporated till dryness under a reduced pressure at 35 °C. The residue was triturated with acetone (400 mL), filtered and washed with acetone (3×250 mL). It was dried under reduced pressure to afford aminoketones **4 a–d** as hydrochlorides.

### 3‐Aminodihydro‐2H‐Pyran‐4(3H)‐One Hydrochloride 4 a

M.p. 146 °C, beige powder, 101.1 g (78.1 % yield). ^1^H NMR (400 MHz, DMSO‐d_6_) δ 8.67 (br.s, 3H), 4.45–4.41 (m, 1H), 4.25–4.21 (m, 1H), 4.18–4.13 (m, 1H), 3.61–3.54 (m, 2H), 2.91–2.83 (m, 1H), 2.42–2.39 (m, 1H). ^13^C NMR (126 MHz, DMSO‐d_6_) δ 201.5, 68.5, 67.7, 55.1, 41.2. HRMS (ESI/TOF−Q) m/z: [M]^+^ calcd for C_5_H_9_NO_2_, 115.0633; found 115.0639.

## General Procedure for the Marckwald Synthesis

To a stirred solution of aminoketone **4 a** (100.0 g, 0.66 mol, 1 eq.) in water (500 mL) was added KSCN (192.4 g, 1.98 mol, 3 eq.). The resulting mixture was heated at 90 °C for 16 hours. Upon completion, the reaction mixture was allowed to cool to room temperature and filtered off. The filter cake was washed with cold water (3×350 mL) and dried under a reduced pressure to afford imidazole derivative **10 a**.

### 3,4,6,7‐Tetrahydropyrano[3,4‐d]Imidazole‐2(1H)‐Thione 10 a

M.p. 225–228 °C, brown powder, 72.0 g (69.9 % yield). ^1^H NMR (400 MHz, DMSO‐d_6_) δ 11.84 (s, 1H), 11.67 (s, 1H), 4.34 (s, 2H), 3.78 (t, J=5.1 Hz, 2H), 2.42 (br.s, 2H). ^13^C NMR (126 MHz, DMSO‐d_6_) δ 159.8, 120.9, 120.1, 63.8, 60.9, 21.5. HRMS (ESI/TOF−Q) m/z: [M]^+^ calcd for C_6_H_8_N_2_OS, 156.0357; found 156.0354.

## Conflict of Interests

The authors declare no conflict of interest.

1

## Supporting information

As a service to our authors and readers, this journal provides supporting information supplied by the authors. Such materials are peer reviewed and may be re‐organized for online delivery, but are not copy‐edited or typeset. Technical support issues arising from supporting information (other than missing files) should be addressed to the authors.

Supporting Information

## Data Availability

The data that support the findings of this study are available in the supplementary material of this article.

## References

[1] V. Erspamer , T. Vitali , M. Roseghini , J. M. Cei , Experientia 1963, 19, 346–347. 14067762 10.1007/BF02152309

[2] V. Erspamer , Annu. Rev. Pharmacol. 1971, 11, 327–350. 4948501 10.1146/annurev.pa.11.040171.001551

[3] M. Roseghini , V. Erspamer , G. F. Erspamer , J. M. Cei , Comp. Biochem. Physiol. C: Comp. Pharmacol. 1986, 85, 139–147. 10.1016/0742-8413(86)90064-2 2877780

[4] V. Erspamer , M. Roseghini , J. M. Cei , Biochem. Pharmacol. 1964, 13, 1083–1093. 14201130 10.1016/0006-2952(64)90104-2

[5] S. Akocak , N. Lolak , S. Bua , A. Nocentini , G. Karakoc , C. T. Supuran , Bioorg. Med. Chem. 2019, 27, 800–804. 30683554 10.1016/j.bmc.2019.01.017

[6] M. Ghiasi , P. Shahabi , C. T. Supuran , Bioorg. Med. Chem. 2021, 44, 116276. 34225168 10.1016/j.bmc.2021.116276

[7] M. A. Prezent , S. V. Baranin , ChemistrySelect 2020, 5, 14017–14020.

[8] P. Jones , R. I. Storer , Y. A. Sabnis , F. M. Wakenhut , G. A. Whitlock , K. S. England , T. Mukaiyama , C. M. Dehnhardt , J. W. Coe , S. W. Kortum , J. E. Chrencik , D. G. Brown , R. M. Jones , J. R. Murphy , T. Yeoh , P. Morgan , I. Kilty , J. Med. Chem. 2017, 60, 767–786. 27983835 10.1021/acs.jmedchem.6b01634

[9] P. A. Stupple , D. V. Batchelor , M. Corless , P. K. Dorr , D. Ellis , D. R. Fenwick , S. R. G. Galan , R. M. Jones , H. J. Mason , D. S. Middleton , M. Perros , F. Perruccio , M. Y. Platts , D. C. Pryde , D. Rodrigues , N. N. Smith , P. T. Stephenson , R. Webster , M. Westby , A. Wood , J. Med. Chem. 2011, 54, 67–77. 21128663 10.1021/jm100978n

[10] P. Matyus , B. Dajka-Halasz , A. Foldi , N. Haider , D. Barlocco , K. Magyar , Curr. Med. Chem. 2004, 11, 1285–1298. 15134520 10.2174/0929867043365305

[11] P. Chen , C. G. Caldwell , R. J. Mathvink , B. Leiting , F. Marsilio , R. A. Patel , J. K. Wu , H. He , K. A. Lyons , N. A. Thornberry , A. E. Weber , Bioorg. Med. Chem. Lett. 2007, 17, 5853–5857. 17869513 10.1016/j.bmcl.2007.08.030

[12] T. Ryckmans , M. P. Edwards , V. A. Horne , A. M. Correia , D. R. Owen , L. R. Thompson , I. Tran , M. F. Tutt , T. Young , Bioorg. Med. Chem. Lett. 2009, 19, 4406–4409. 19500981 10.1016/j.bmcl.2009.05.062

[13] S. Froidevaux, F. Hubler, M. Murphy, D. Renneberg, S. Stamm, „C5a receptor modulators“, Idorsia Pharmaceuticals Ltd patent US20200347029 A1.

[14] P. Lam, C. Clark, R. Li, D. Pinto, „Thrombin or factor Xa inhibitors“, Bristol Myers Squibb Pharma Co patent US6369227B1.

[15] J. Dellaria, Jr. Moser, M. Radmer, G. Gries-Graber. „Hydroxy substituted 1H-imidazopyridines and methods“, Pfizer Inc patent US20100152230A1.

[16] D. Lee, S. Chae, E. Jung, E. Yang, Y. Choi, C.-W. Chung, J. Shin, Y. Kim, H. Kwon, J. Ryu, E. Ban, Y. Kim, Y. Oh, J. Chae, „Compounds as autotaxin inhibitors and pharmaceutical compositions comprising the same“, Ligachem Biosciences Inc patent US11548883B2.

[17] B. Lippa , G. Pan , M. Corbett , C. Li , G. S. Kauffman , J. Pandit , S. Robinson , L. Wei , E. Kozina , E. S. Marr , G. Borzillo , E. Knauth , E. G. Barbacci-Tobin , P. Vincent , M. Troutman , D. Baker , F. Rajamohan , S. Kakar , T. Clark , J. Morris , Bioorg. Med. Chem. Lett. 2008, 18, 3359–3363. 18456494 10.1016/j.bmcl.2008.04.034

[18] D. Ramsbeck , N. Taudte , N. Jänckel , S. Strich , J.-U. Rahfeld , M. Buchholz , Pharmaceuticals (Basel) 2021, 14, 1206. 34959608 10.3390/ph14121206 PMC8709289

[19] Y. Lee , J. Jang , M. Bibi , K. B. Duggirala , S. H. Ji , J. H. Lee , S. Ahn , J. S. Song , C. H. Chae , S. H. Kim , K. Lee , Bull. Korean Chem. Soc. 2021, 42, 872–877.

[20] H. Bredereck , R. Gompper , H. G. V. Schuh , G. Theilig , Angew. Chem. 1959, 71, 753–774.

[21] R. Weidenhagen , H. Wegner , Berichte der deutschen chemischen Gesellschaft (A and B Series) 1938, 71, 2124–2134.

[22] E. Lebenstedt , W. Schunack , Arch. Pharm. (Weinheim) 1974, 307, 894–896. 4433230 10.1002/ardp.19743071119

[23] C. J. Lovely , H. Du , Y. He , H. V. Rasika Dias , Org. Lett. 2004, 6, 735–738. 14986962 10.1021/ol036403w

[24] N. Luise , E. W. Wyatt , G. J. Tarver , P. G. Wyatt , European J. Org. Chem. 2019, 2019, 1341–1349.

[25] M. Li , H. Liu , B. Zhong , Synth. Commun. 2007, 37, 1001–1009.

[26] V. A. Reader , Synlett 1998, 1998, 1077–1078.

[27] R. G. Jones , E. C. Kornfeld , K. C. McLaughlin , R. C. Anderson , J. Am. Chem. Soc. 1949, 71, 4000–4002.

[28] H. Heath, A. Lawson, C. Rimington, *J. Chem. Soc. (Resumed)* **1951**, 2215.

[29] R. A. F. Bullerwell, A. Lawson, *J. Chem. Soc. (Resumed)* **1951**, 3030.

[30] W. Marckwald , Ber. Dtsch. Chem. Ges. 1892, 25, 2354–2373.

[31] N. Xi , S. Xu , Y. Cheng , A. S. Tasker , R. W. Hungate , P. J. Reider , Tetrahedron Lett. 2005, 46, 7315–7319.

[32] V. Lysenko , A. Portiankin , T. Shvydenko , K. Shvydenko , S. Shishkina , A. Kostyuk , Synth. Commun. 2023, 53, 615–624.

[33] P. W. Neber , A. V. Friedolsheim , Justus. Liebigs. Ann. Chem. 1926, 449, 109–134.

[34] W. F. Berkowitz , Organic Reactions, John Wiley & Sons, Inc., Hoboken, NJ, USA, 2012, 321–410.

[35] C. O'Brien , Chem. Rev. 1964, 64, 81–89.

[36] M. M. A. Pereira , P. P. Santos , PATAI'S Chemistry of Functional Groups, John Wiley & Sons, Ltd, Chichester, UK, 2010.

[37] P. W. Neber , A. V. Friedolsheim , Justus. Liebigs. Ann. Chem. 1926, 449, 109–134.

[38] V. Sridharan , S. Muthusubramanian , S. Sivasubramanian , J. Heterocycl. Chem. 2005, 42, 1321–1330.

[39] A. Diez , A. Voldoire , I. López , M. Rubiralta , V. Segarra , L. Pagès , J. Palacios , Tetrahedron 1995, 51, 5143–5156.

[40] A. HORN , B. KAPTEIN , N. VERMUE , J. DEVRIES , T. MULDER , Eur. J. Med. Chem. 1988, 23, 325–328.

[41] A. S. Horn , B. Kaptein , T. B. A. Mulder , J. B. de Vries , H. Wynberg , J. Med. Chem. 1984, 27, 1340–1343. 6090664 10.1021/jm00376a020

[42] A. Delgado , D. Mauleón , G. Rosell , R. Granados , Eur. J. Med. Chem. 1988, 23, 31–38.

[43] J. Cai , M. Huang , Y. Wang , X. Chen , M. Ji , Bioorg. Med. Chem. Lett. 2021, 48, 128269. 34284107 10.1016/j.bmcl.2021.128269

[44] A. Delgado , J. M. Garcia , D. Mauleon , C. Minguillon , J. R. Subirats , M. Feliz , F. Lopez , D. Velasco , Can. J. Chem. 1988, 66, 517–527.

[45] K. Itoh , A. Miyake , N. Tada , M. Hirata , Y. Oka , Chem. Pharm. Bull. (Tokyo) 1984, 32, 130–151. 6144399 10.1248/cpb.32.130

[46] L. A. van Vliet , N. Rodenhuis , D. Dijkstra , H. Wikström , T. A. Pugsley , K. A. Serpa , L. T. Meltzer , T. G. Heffner , L. D. Wise , M. E. Lajiness , R. M. Huff , K. Svensson , S. Sundell , M. Lundmark , J. Med. Chem. 2000, 43, 2871–2882. 10956195 10.1021/jm0000113

[47] D. Huckle, I. M. Lockhart, N. E. Webb, *J. Chem. Soc. C: Organic* **1971**, 2252. 10.1039/j39710002252 5170054

[48] G. de Stevens , A. Halamandaris , J. Am. Chem. Soc. 1957, 79, 5710–5711.

