# Supplementary material for: Synthesis of Fused sp3‐Enriched Imidazoles
Source: ChemistryOpen. 2024 Nov 13;14(3):e202400272. doi: 10.1002/open.202400272 (PMC11891454; doi:10.1002/open.202400272)
Supplement: Supplementary file 1 — Supporting Information [file OPEN-14-e202400272-s001.pdf]

SYNTHESIS of FUSED sp^3-ENRICHED IMIDAZOLES

Viacheslav Lysenko,[a] Anton Portiankin,[c] Tetiana Shvydenko,[a] Svitlana Shishkina,[b] Kostiantyn Nazarenko,[a,c] and Aleksandr Kostyuk*[a]

[a] V. Lysenko, T. Shvydenko, K. Nazarenko, and Prof. A. Kostyuk
Department of Organophosphorus Chemistry
Institute of Organic Chemistry
Academician Kukhar str. 5, 02094, Kyiv-94, Ukraine
E-mail: a.kostyuk@yahoo.com; kostyuk@ioch.kiev.ua

[b] Dr. S. Shishkina
SSI "Institute of Single Crystals" NAS of Ukraine

[c] K. Nazarenko, A. Portiankin,
Enamine Ltd., Winston Churchill Street 78, Kyiv, 02094, Ukraine

TABLE OF CONTENT

1. Experimental section — **Pages S1-S18**
2. NMR spectra of new compounds — **Pages S19 –S168**
3. Single-crystal X-ray diffraction study — **Pages S169-S170**

Compound #	Description	Page	Compound #	Description	Page	Compound #	Description	Page	Compound #	Description	Page
**2a**	^{1}H NMR	S19	**2d**	^{13}C NMR	S32	**3d**	^{13}C NMR	S45	**4c**	^{13}C NMR	S58
**2a**	^{13}C NMR	S20	**2d**	^{19}F NMR	S33	**3d**	^{19}F NMR	S46	**4c**	HRMS	S59-60
**2a**	HRMS	S21	**2d**	HRMS	S34	**3d**	HRMS	S47	**4d**	^{1}H NMR (CF3COOD)	S61
**2b**	^{1}H NMR	S23	**3a**	^{1}H NMR	S36	**4a**	^{1}H NMR	S49	**4d**	^{1}H NMR (D2O)	S62
**2b**	^{13}C NMR	S24	**3a**	^{13}C NMR	S37	**4a**	^{13}C NMR	S50	**4d**	^{13}C NMR	S63
**2b**	HRMS	S25	**3a**	HRMS	S38	**4a**	HRMS	S51	**4d**	^{19}F NMR	S64
**2c**	^{1}H NMR	S27	**3b**	^{1}H NMR	S40	**4b**	^{1}H NMR	S53	**4d**	HRMS	S65-66
**2c**	^{13}C NMR	S28	**3b**	^{13}C NMR	S41	**4b**	^{13}C NMR	S54	**4e**	^{1}H NMR (D2O)	S67
**2c**	HRMS	S29-30	**3b**	HRMS	S42-43	**4b**	HRMS	S55-56	**4e**	^{1}H NMR (CF3COOD)	S68
**2d**	^{1}H NMR	S31	**3d**	^{1}H NMR	S44	**4c**	^{1}H NMR	S57	**4e**	^{1}H NMR (DMSO-d6)	S69

Compound #	Description	Page	Compound #	Description	Page	Compound #	Description	Page	Compound #	Description	Page
**4e**	^{13}C NMR	S70	**8**	HRMS	S87	**9d**	HRMS	S104	**10d**	HRMS	S121
**4e**	HRMS	S71	**9a**	^{1}H NMR	S89	**9e**	^{1}H NMR	S106	**10e**	^{1}H NMR	S123
**5**	^{1}H NMR	S73	**9a**	^{13}C NMR	S90	**9e**	^{13}C NMR	S107	**10e**	^{13}C NMR	S124
**5**	^{13}C NMR	S74	**9a**	HRMS	S91	**9e**	HRMS	S108	**10e**	HRMS	S125
**5**	HRMS	S75	**9b**	^{1}H NMR	S93	**10b**	^{1}H NMR	S110	**11a**	^{1}H NMR	S127
**6**	^{1}H NMR	S77	**9b**	^{13}C NMR	S94	**10b**	^{13}C NMR	S111	**11a**	^{13}C NMR	S128
**6**	^{13}C NMR	S78	**9b**	HRMS	S95	**10b**	HRMS	S112	**11a**	HRMS	S129
**6**	HRMS	S79	**9c**	^{1}H NMR	S97	**10c**	^{1}H NMR	S114	**11c**	^{1}H NMR	S131
**7**	^{1}H NMR	S81	**9c**	^{13}C NMR	S98	**10c**	^{13}C NMR	S115	**11c**	^{13}C NMR	S132
**7**	^{13}C NMR	S82	**9c**	HRMS	S99	**10c**	HRMS	S116	**11c**	HRMS	S133
**7**	HRMS	S83	**9d**	^{1}H NMR	S101	**10d**	^{1}H NMR	S118	**11d**	^{1}H NMR	S135
**8**	^{1}H NMR	S85	**9d**	^{13}C NMR	S102	**10d**	^{13}C NMR	S119	**11d**	^{13}C NMR	S136
**8**	^{13}C NMR	S86	**9d**	^{19}F NMR	S103	**10d**	^{19}F NMR	S120	**11d**	^{19}F NMR	S137

Compound #	Description	Page	Compound #	Description	Page	Compound #	Description	Page	Compound #	Description	Page
**11d**	HRMS	S138	**13c**	^{1}H NMR	S148	**15+15’**	^{13}C NMR	S157	**17**	^{1}H NMR	S164
**11e**	^{1}H NMR	S140	**13c**	^{13}C NMR	S149	**15+15’**	APT	S158	**17**	^{13}C NMR	S165
**11e**	^{13}C NMR	S141	**13c**	HRMS	S150-151	**15+15’**	LCMS	S159	**17**	GCMS	S166-168
**11e**	HRMS	S142-143	**14c**	^{1}H NMR	S152	**16+16’**	^{1}H NMR	S160			
**12c**	^{1}H NMR	S144	**14c**	^{13}C NMR	S153	**16+16’**	^{13}C NMR	S161			
**12c**	^{13}C NMR	S145	**14c**	HRMS	S154-155	**16+16’**	APT	S162			
**12c**	HRMS	S146-147	**15+15’**	^{1}H NMR	S156	**16+16’**	LCMS	S163			

Experimental part

Solvents were purified according to the standard procedures. Melting points were measured on MPA100 OptiMelt automated melting point system. Analytical TLC was performed using Polychrom SI F254 plates. Column chromatography was performed using Kieselgel Merck 60 (230–400 mesh) as the stationary phase. ^{1}H and ^{13}C NMR spectra were recorded on a Bruker 170 Avance 500 spectrometer (at 500 MHz for ^{1}H NMR, 126 MHz for ^{13}C NMR and 470 MHz for ^{19}F NMR) and Varian Unity Plus 400 spectrometer (at 400 MHz for ^{1}H NMR, 101 MHz for ^{13}C NMR and 376 MHz for ^{19}F NMR). NMR chemical shifts are reported in ppm (δ scale) downfield from TMS as an internal standard and are referenced using residual NMR solvent peaks at 7.26 and 77.16 ppm for ^{1}H and ^{13}C in $CDCl_3$, 2.50 and 39.52 ppm for ^{1}H and ^{13}C in DMSO-d6. High-resolution mass spectra (HRMS) were obtained on an Agilent 1260 Infinity UHPLC instrument coupled with an Agilent 6224 Accurate Mass TOF mass spectrometer.

General procedure for the preparation of oximes 2a-d.

To a solution of ketone **1a** (150.0 g, 1.49 mol, 1 eq.) in absolute ethanol (1200 mL) were added hydroxylamine hydrochloride (205.6 g, 2.98 mol, 2 eq.) and sodium acetate (256.6, 3.13 mol, 2.1 eq.). The mixture was heated at reflux overnight. The reaction mixture was allowed to cool to room temperature and volatiles were removed under reduced pressure. The residue was partitioned between water and EtOAc (1000 mL/1000 mL). The aqueous layer was extracted with EtOAc (4×300 mL), the combined organic layers were washed with saturated solution of $NaHCO_3$ and brine, dried over Na_2SO_4 and concentrated under reduced pressure to dryness to afford the corresponding oximes **2a-d** as solids.

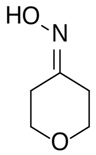

Dihydro-2H-pyran-4(3H)-one oxime 2a

M.p. 87°C, white solid, 148.7 g (86.2% yield). ^{1}H NMR (400 MHz, $CDCl_3$) δ 8.79 (br.s, 1H), 3.84-3.81 (m, 2H), 3.78-3.75 (m, 2H), 2.70-2.67 (m, 2H), 2.40-2.37 (m, 2H). ^{13}C NMR (126 MHz, $CDCl_3$) δ 155.4, 67.7, 66.2, 31.7, 25.5. HRMS (ESI/TOF-Q) *m/z*: $[M]^+$ calcd for $C_5H_9NO_2$, 115.0633; found 115.0633.

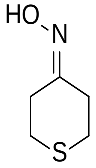

Dihydro-2H-thiopyran-4(3H)-one oxime 2b

M.p. 83°C, a yellowish solid, 141.6 g (99% yield). ^{1}H NMR (400 MHz, $CDCl_3$) δ 9.44 (br.s, 1H), 2.87-2.85 (m, 2H), 2.79-2.77 (m, 2H), 2.74-2.72 (m, 2H), 2.57-2.55 (m, 2H). ^{13}C NMR (126 MHz, $CDCl_3$) δ 157.7, 33.4, 29.2, 27.8, 26.2. HRMS (ESI/TOF-Q) *m/z*: $[M]^+$ calcd for C_5H_9NOS, 131.0405; found 131.0402.

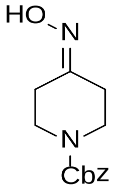

Benzyl 4-(hydroxyimino)piperidine-1-carboxylate 2c

M.p. 106°C, a white solid, 138.1 g (74.9% yield). ^{1}H NMR (400 MHz, $CDCl_3$) 9.18 (br.s, 1H), 7.39-7.34 (m, 5H), 5.18 (s, 2H), 3.66-3.61 (m, 4H), 2.65 (br.s, 2H), 2.39 (br.s, 2H). ^{13}C NMR (126 MHz, $CDCl_3$) 155.9, 154.8, 136.0, 128.0, 127.7, 127.5, 66.96, 43.44, 41.74, 30.39, 24.23. HRMS (ESI/TOF-Q) *m/z*: $[M]^+$ calcd for $C_{13}H_{16}N_2O_3$, 248.1161; found 248.1164.

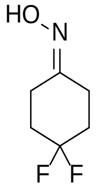

4,4-Difluorocyclohexanone oxime 2d

M.p. 106°C, a white solid, 162.4 g (71.8% yield). ^{1}H NMR (500 MHz, $CDCl_3$) δ 9.58 (br.s, 1H), 2.73 (t, J =7.00 Hz, 2H), 2.46 (t, J =7.00 Hz, 2H), 2.14-2.02 (m, 4H). ^{13}C NMR (126 MHz, $CDCl_3$) δ 155.9 (s), 122.0 (t, J_{CF} =242 Hz), 32.8 (t, J_{CF} =24 Hz), 31.6 (t, J_{CF} =25 Hz), 27.1 (t, J_{CF} =6 Hz), 19.5 (t, J_{CF} =6 Hz). ^{19}F (376 MHz, $CDCl_3$) δ -99.19 (s, 2F). HRMS (ESI/TOF-Q) *m/z*: $[M]^+$ calcd for $C_6H_9F_2NO$, 149.0652; found 149.0650.

General procedure for the tosylation of oximes 3a-d

To an ice cold solution of oxime **2a** (100.0 g, 0.87 mol, 1 eq.) and triethylamine (176.1 g, 1.74 mol, 2 eq.) in anhydrous dichloromethane (1000 mL) was added 4-methylbenzene-1-sulfonyl chloride (174.2 g, 0.91 mol, 1.05 eq.) portion wise, and the reaction mixture was stirred overnight at room temperature. Water (500 mL) was added to the reaction mixture; the organic layer was separated and washed with water (4×300 mL). The organic layer was dried over Na_2SO_4 and evaporated under reduced pressure to give O-tosyl oximes **3a-d** as dark drown solids. All products **3a-d** are unstable and were used immediately in the next step without any purification.

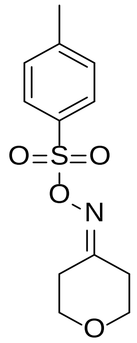

Dihydro-2H-pyran-4(3H)-one O-tosyl oxime 3a

M.p. 84-86°C, a brown powder, 222.7 g (95.2% yield). ^{1}H NMR (400 MHz, $CDCl_3$) δ 7.86 (d, J =4.00 Hz, 2H), 7.35 (d, J =3.80 Hz, 2H), 3.79 (t, J =5.60 Hz, 2H), 3.73 (t, J =6.00 Hz, 2H), 2.67 (t, J =5.60 Hz, 2H), 2.46 (s, 3H), 2.40 (t, J =6.00 Hz, 2H). ^{13}C NMR (126 MHz, $CDCl_3$) δ 164.7, 144.5, 132.2, 129.1, 128.3, 67.1,

65.8, 31.5, 27.5, 21.2. HRMS (ESI/TOF-Q) *m/z*: $[M]^+$ calcd for $C_{12}H_{15}NO_4S$, 269.0722; found 269.0717.

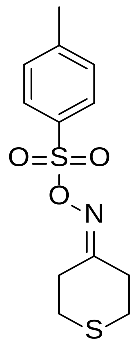

Dihydro-2H-thiopyran-4(3H)-one O-tosyl oxime 3b

M.p. 73-75°C, a brown powder, 246.3 g (96.4% yield). ^{1}H NMR (400 MHz, $CDCl_3$) δ 7.85 (d, *J* =4.00 Hz, 2H), 7.34 (d, *J* =4.20 Hz, 2H), 2.88-2.85 (m, 2H), 2.79-2.76 (m, 2H), 2.73-2.70 (m, 2H), 2.60-2.57 (m, 2H), 2.45 (s, 3H). ^{13}C NMR (126 MHz, $CDCl_3$) δ 166.7, 144.5, 132.1, 129.1, 128.3, 33.1, 28.9, 28.3, 27.7, 21.2. HRMS (ESI/TOF-Q) *m/z*: $[M]^+$ calcd for $C_{12}H_{15}NO_3S_2$, 285.0493; found 285.0480.

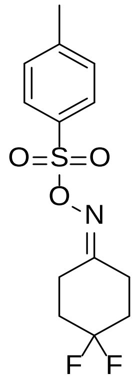

4,4-Difluorocyclohexanone O-tosyl oxime 3d

M.p. 81-84°C, a yellow powder, 230.7 g (93.7% yield). ^{1}H NMR (400 MHz, $CDCl_3$) δ 7.85 (d, *J* =3.60 Hz, 2H), 7.35 (d, *J* =3.60 Hz, 2H), 2.76-2.72 (m, 2H), 2.50-2.43 (m, 5H), 2.14-1.99 (m, 4H). ^{13}C NMR (100 MHz, $CDCl_3$) δ 165.0, 145.2, 132.5, 129.7, 128.8, 121.6 (t, J_{CF} =240.0 Hz), 32.8 (t, J_{CF} =26.0 Hz), 31.8 (t, J_{CF} =26.00 Hz), 27.6 (t, J_{CF} =5.00 Hz), 22.2 (t, J_{CF} =5.0 Hz), 21.7. ^{19}F (376 MHz, $CDCl_3$) δ -99.78. HRMS (ESI/TOF-Q) *m/z*: $[M]^+$ calcd for $C_{13}H_{15}F_2NO_3S$, 303.0741; found 303.0736

General procedure for the Neber rearrangement of compounds 3a-d

To a solution of potassium tert-butoxide (110.0 g, 0.98 mol, 1.15 eq.) in absolute methanol (1200 mL) was added O-tosyl oxime **3a** (230.0 g, 0.85 mol, 1 eq.) portion wise at 10˚C. The resulting mixture was stirred for 3 h at room temperature. Concentrated HCl (160 mL) was poured in a Büchner flask and the reaction mixture was filtered into it. The filtrate with concentrated HCl was stirred at room temperature for 15 minutes and evaporated till dryness under a reduced pressure at 35˚C. The residue was triturated with acetone (400 mL), filtered and washed with acetone (3×250 mL). It was dried under reduced pressure to afford aminoketone **4a-d** as hydrochlorides.

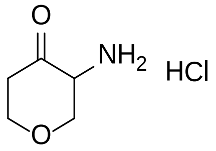

3-Aminodihydro-2H-pyran-4(3H)-one hydrochloride 4a

M.p. 146˚C, a beige powder, 101.1 g (78.1% yield). ^{1}H NMR (400 MHz, DMSO-d_6) δ 8.67 (br.s, 3H), 4.45-4.41 (m, 1H), 4.25-4.21 (m, 1H), 4.18- 4.13 (m, 1H), 3.61-3.54 (m, 2H), 2.91-2.83 (m, 1H), 2.42-2.39 (m, 1H). ^{13}C NMR (126 MHz, DMSO-d_6) δ 201.5, 68.5, 67.7, 55.1, 41.2. HRMS (ESI/TOF-Q) *m/z*: $[M]^+$ calcd for $C_5H_9NO_2$, 115.0633; found 115.0639.

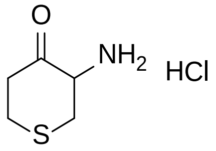

3-Aminodihydro-2H-thiopyran-4(3H)-one hydrochloride 4b

M.p. 171˚C, a beige powder, 109.7 g (86.7% yield). ^{1}H NMR (400 MHz, DMSO-d_6) δ 8.61 (br.s, 3H), 4.27-4.23 (m, 1H), 3.48 (br.s, 1H), 3.29-3.24 (m, 1H), 3.01-2.90 (m, 4H), 2.75-2.72 (m, 1H). ^{13}C NMR (126 MHz, DMSO-d_6) δ 202.8, 57.8, 43.3, 32.3, 29.3. HRMS (ESI/TOF-Q) *m/z*: $[M]^+$ calcd for C_5H_9NOS, 131.0405; found 131.0405.

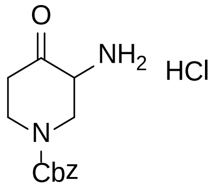

Benzyl 3-amino-4-oxopiperidine-1-carboxylate hydrochloride 4c

M.p. 173-175°C, a beige powder, 96.7 g (79.6% yield). ^{1}H NMR (400 MHz, DMSO-d$_6$) δ 8.62 (br.s, 3H), 7.43-7.34 (m, 5H), 5.15 (s, 2H), 4.64-4.59 (m, 1H), 4.30-4.25 (m, 1H), 4.21-4.16 (m, 1H), 3.24-3.12 (m, 2H), 2.83-2.74 (m, 1H), 2.44-2.40 (m, 1H). ^{13}C NMR (126 MHz, DMSO-d$_6$) δ 202.2, 154.2, 136.4, 128.4, 127.9, 127.6, 66.9, 66.6, 54.1, 45.5, 42.9. HRMS (ESI/TOF-Q) *m/z*: $[M]^+$ calcd for $C_{13}H_{16}N_2O_3$, 248.1161; found 248.1164.

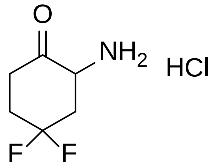

2-Amino-4,4-difluorocyclohexanone hydrochloride 4d

M.p. 139°C, a beige powder, 106.9 g (75.3% yield). ^{1}H NMR (400 MHz, CF_3COOD) δ 8.03 (br.s, 1H), 5.04-5.01 (m, 1H), 3.53 (br.s, 1H), 3.35-3.28 (m, 1H), 3.21-3.18 (m, 1H), 3.12-3.00 (m, 2H), 2.81-2.69 (m, 1H). ^{13}C NMR (126 MHz, D_2O) δ 122.3 (t, J_{CF} =240 Hz), 91.3 (s), 53.6 (d, J_{CF} =10.3 Hz), 34.1 (t, J_{CF} =26.2 Hz), 31.8 (d, J_{CF} =10.3 Hz), 29.9 (t, J_{CF} =24.6 Hz). ^{19}F (376 MHz, D_2O) δ -93.85, -94.50, -95.51, -96.16, -99.90, -100.55, -100.91, -101.57. HRMS (ESI/TOF-Q) *m/z*: $[M]^+$ calcd for $C_6H_9F_2NO$, 149.0652; found 149.0653.

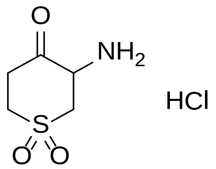

3-Aminodihydro-2H-thiopyran-4(3H)-one 1,1-dioxide hydrochloride 4e

To a concentrated HCl (400 mL) was added compound **8** (90.0 g, 0.43 mol) and the resulting solution was heated at 50°C for 16 hours. Upon completion, the

reaction mixture was allowed to cool to room temperature and evaporated under a reduced pressure to afford **4e** as hydrochloride**.**

Mp. ≥200°C decomposition, a beige powder, 85.9 g (quantitative yield)**.** ^{1}H NMR (400 MHz, D_2O) δ 3.90-3.86 (m, 1H), 3.66-3.64 (m, 2H), 3.58-3.50 (m, 1H), 3.45-3.38 (m, 1H), 2.57-2.50 (m, 1H), 2.48-2.40 (m, 1H). ^{1}H NMR (400 MHz, CF_3COOD) δ 5.24-5.20 (m, 1H), 4.42-4.39 (m, 2H), 4.07-3.99 (m, 1H), 3.86-3.81 (m, 1H), 3.57-3.48 (m, 1H), 3.34-3.30 (m, 1H). ^{13}C NMR (126 MHz, D_2O) δ 90.1, 53.9, 49.7, 48.0, 32.9. HRMS (ESI/TOF-Q) *m/z*: $[M]^+$ calcd for $C_5H_9NO_3S$, 163.0303; found 163.0302.

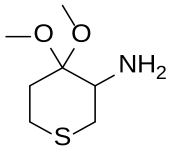

4,4-Dimethoxytetrahydro-2H-thiopyran-3-amine 5

To a solution of potassium tert-butoxide (157.1 g, 1.4 mol, 1.95 eq.) in absolute methanol (1 L) was added O-tosyl oxime **3b** (205.0 g, 0.72 mol, 1 eq.) portion wise at room temperature, and the mixture was refluxed for 3 h. Upon completion, the reaction mixture was allowed to cool to room temperature and evaporated under a reduced pressure to dryness. The residue was triturated with MTBE (1000 mL), filtered off and filter cake was washed with MTBE (3×350 mL). The combined filtrate was washed with water (3×300 mL), dried over Na_2SO_4 and concentrated to afford a crude product. The pure aminoacetal **5** was obtained by distillation as a thick oil.

B.p. 73-76°C at 1 Torr, a yellowish oil, 91.0 g (71.5% yield). ^{1}H NMR (400 MHz, $CDCl_3$) δ 3.19-3.10 (m, 8H), 2.71-2.64 (m, 1H), 2.52-2.47 (m, 1H), 2.32-2.26 (m, 1H), 1.98-1.93 (m, 1H), 1.84-1.76 (m, 1H), 1.62 (br.s, 2H). ^{13}C NMR (126 MHz, $CDCl_3$) δ 99.6, 47.8, 47.1, 46.2, 32.3, 27.7, 24.5. HRMS (ESI/TOF-Q) *m/z*: $[M]^+$ calcd for $C_7H_{15}NO_2S$, 177.0823; found 177.0818.

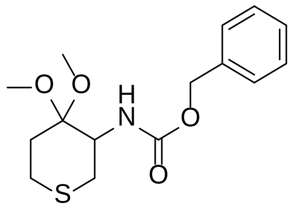

Benzyl (4,4-dimethoxytetrahydro-2H-thiopyran-3-yl)carbamate 6

To a stirred solution of Na_2CO_3 (81.6 g, 0.77 mol, 1.5 eq.) and water (900 mL) was added 4,4-dimethoxytetrahydro-2H-thiopyran-3-amine **5** (90.0 g, 0.51 mol, 1 eq.) in THF (450 mL)**.** To the ice cooled reaction mixture was added benzyl chloroformate (92.1 g, 0.54 mol, 1.05 eq.) dropwise and the mixture was stirred at room temperature overnight. After completion, volatiles were removed under reduced pressure and the resulting mixture was extracted with DCM (4×350 mL). The combined organic extracts were dried over Na_2SO_4 and evaporated under a reduced pressure to dryness to afford the target compound **6**.

A yellow oil, 155.4 g (98.3% yield). ^{1}H NMR (500 MHz, $CDCl_3$) δ 7.37-7.29 (m, 5H), 5.64 (d, *J* =4.25 Hz, 1H), 5.11 (s, 2H), 4.17 (d, *J* =3.25 Hz, 1H), 3.23-3.15 (m, 7H), 2.68 (t, *J* =12.50 Hz, 1H), 2.54 (d, *J* =7.00 Hz, 1H), 2.34 (d, *J* =6.50 Hz, 1H), 2.10 (d, *J* =7.50 Hz, 1H), 1.68 (t, *J* =13.50 Hz, 1H). ^{13}C NMR (150 MHz, $CDCl_3$) δ 155.6, 136.4, 128.5, 128.1, 125.1, 98.7, 66.9, 48.5, 47.8, 47.8, 32.2, 29.8, 24.8. HRMS (ESI/TOF-Q) *m/z*: $[M]^+$ calcd for $C_{15}H_{21}NO_4S$, 311.1191; found 311.1182.

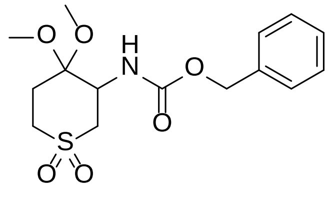

Benzyl (4,4-dimethoxy-1,1-dioxidotetrahydro-2H-thiopyran-3-yl)carbamate 7

To a stirred solution of benzyl (4,4-dimethoxytetrahydro-2H-thiopyran-3-yl)carbamate **6** (150.0 g, 0.48 mol, 1 eq.) in DCM (1200 mL) was added m-CPBA (207.1 g, 90% purity, 1.2 mol, 2.5 eq.) in portions at 5°C and stirred overnight at room temperature (25°C). To the reaction mixture was added a saturated solution of $Na_2S_2O_3$ (200 mL) to quench m-CPBA excess. The resulting suspension was filtered,

filter cake was washed with with DCM (3×350 mL). The filtrate was separated and the organic layer was washed with K_2CO_3 solution (20% in water, 3×300 mL), dried over Na_2SO_4 and concentrated to give **7**.

M.p. 133-135°C, a white powder, 165.3 g (quantitative yield). ^{1}H NMR (500 MHz, $CDCl_3$) δ 7.37-7.33 (m, 5H), 6.07 (d, J =5.00 Hz, 1H), 5.13 (s, 2H), 4.54-4.52 (m, 1H), 3.53-3.50 (m, 1H), 3.27 (s, 6H), 3.14-3.08 (m, 1H), 3.04-2.94 (m, 2H), 2.27-2.24 (m, 2H). ^{13}C NMR (126 MHz, $CDCl_3$) δ 155.7, 136.1, 128.5, 128.2, 128.1, 97.8, 67.2, 51.7, 49.4, 49.2, 48.5, 48.0, 25.9. HRMS (ESI/TOF-Q) *m/z*: $[M]^+$ calcd for $C_{15}H_{21}NO_6S$, 343.1090; found 311.1082.

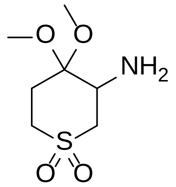

3-Amino-4,4-dimethoxytetrahydro-2H-thiopyran 1,1-dioxide 8

To a solution of benzyl (4,4-dimethoxy-1,1-dioxidotetrahydro-2H-thiopyran-3-yl)carbamate **7** (150.0 g, 0.44 mol, 1 eq.) in methanol (1300 mL) was added 10% Pd/C (20 g) and the reaction mixture was stirred under hydrogen atmosphere (balloon pressure) until the reaction is complete (PMR monitoring). The reaction mixture was filtered, the filtered cake was washed with DCM (3×400 mL). The combined organic extracts were dried over Na_2SO_4, and evaporated under a reduced pressure to afford acetal **8** as a white solid.

M.p. 159°C, a white powder, 91.1 g (99% yield).

^{1}H NMR (500 MHz, $CDCl_3$) δ 3.50-3.48 (m, 1H), 3.46-3.42 (m, 1H), 3.27 (s, 3H), 3.24 (s, 3H), 3.16-3.06 (m, 2H), 2.99-2.93 (m, 1H), 2.45-2.39 (m, 1H), 2.20-2.14 (m, 1H), 1.88 (br.s, 2H). ^{13}C NMR (100 MHz, $CDCl_3$) δ 99.2, 52.4, 50.6, 48.5, 48.3, 48.3, 24.6. HRMS (ESI/TOF-Q) *m/z*: $[M]^+$ calcd for $C_7H_{15}NO_4S$, 209.0722; found 209.0717.

General procedure for the Marckwald synthesis

To a stirred solution of aminoketone **4a** (100.0 g, 0.66 mol, 1 eq.) in water (500 mL) was added KSCN (192.4 g, 1.98 mol, 3 eq.) and the resulting mixture was heated at 90˚C for 16 hours. Upon completion, the reaction mixture was allowed to cool to room temperature and filtered off. The filter cake was washed with cold water (3×350 mL) and dried under a reduced pressure to afford imidazole derivative **10a**.

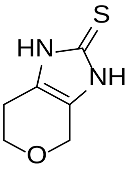

3,4,6,7-Tetrahydropyrano[3,4-d]imidazole-2(1H)-thione 9a

M.p. 225-228˚C, a brown powder, 72.0 g (69.9% yield). ^{1}H NMR (400 MHz, DMSO-d_6) δ 11.84 (s, 1H), 11.67 (s, 1H), 4.34 (s, 2H), 3.78 (t, J = 5.1 Hz, 2H), 2.42 (br.s, 2H). ^{13}C NMR (126 MHz, DMSO-d_6) δ 159.8, 120.9, 120.1, 63.8, 60.9, 21.5. HRMS (ESI/TOF-Q) *m/z*: $[M]^+$ calcd for $C_6H_8N_2OS$, 156.0357; found 156.0354.

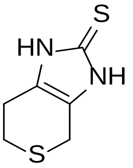

3,4,6,7-Tetrahydrothiopyrano[3,4-d]imidazole-2(1H)-thione 9b

M.p. ≥240˚C, a beige powder, 50.1 g (78.4% yield). ^{1}H NMR (400 MHz, DMSO-d_6) δ 11.75 (s, 2H), 3.39 (s, 2H), 2.80 (br.s, 2H), 2.52 (br.s, 2H). ^{13}C NMR (126 MHz, DMSO-d_6) δ 158.5, 122.9, 119.0, 24.5, 22.3, 20.4. HRMS (ESI/TOF-Q) *m/z*: $[M]^+$ calcd for $C_6H_8N_2S_2$, 172.0129; found 172.0127.

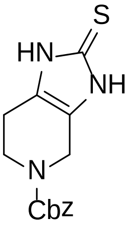

Benzyl 2-thioxo-2,3,6,7-tetrahydro-1H-imidazo[4,5-c]pyridine-5(4H)-carboxylate 9c

M.p. 186-188°C, a brown powder, 55.9 g (91.0% yield). ^{1}H NMR (400 MHz, DMSO-d_6) δ 11.87-11.75 (m, 2H), 7.36-7.32 (m, 5H), 5.10 (s, 2H), 4.25 (br.s, 2H), 3.64 (br.s, 2H), 2.42 (br.s, 2H). ^{13}C NMR (126 MHz, DMSO-d_6) δ 160.3, 154.8, 136.6, 128.4, 127.9, 127.6, 121.1, 119.6, 66.6, 40.9, 20.8, 20.4. HRMS (ESI/TOF-Q) *m/z*: $[M]^+$ calcd for $C_{14}H_{15}N_3O_2S$, 289.0885; found 289.0884.

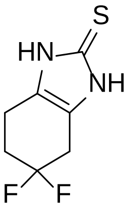

5,5-Difluoro-4,5,6,7-tetrahydro-1H-benzo[d]imidazole-2(3H)-thione 9d

M.p. ≥240°C, a brown powder, 63.7 g (82.2% yield). ^{1}H NMR (400 MHz, DMSO-d_6) δ 11.85 (br.s, 1H), 11.80 (br.s, 1H), 3.34 (br.s, 2H), 2.94 (t, J =13.2 Hz, 2H), 2.23-2.15 (m, 2H). ^{13}C NMR (126 MHz, DMSO-d_6) δ 161.1 (s), 123.2 (t, J_{CF} =242 Hz), 120.6 (s), 117.9 (s), 30.8 (t, J_{CF} = 28.4 Hz), 29.7 (t, J_{CF} = 24.7 Hz), 17.3 (s). ^{19}F (376 MHz, DMSO-d_6) δ -94.82. HRMS (ESI/TOF-Q) *m/z*: $[M]^+$ calcd for $C_7H_8F_2N_2S$, 190.0376; found 190.0375.

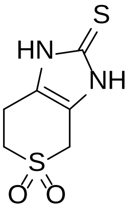

3,4,6,7-Tetrahydrothiopyrano[3,4-d]imidazole-2(1H)-thione 5,5-dioxide 9e

M.p. ≥240°C, a grey powder, 27.3 g (73.2% yield). ^{1}H NMR (400 MHz, DMSO-d_6) δ 12.04 (s, 1H), 11.91 (s, 1H), 4.16 (br.s, 2H), 3.38 (br.s, 2H), 2.82 (br.s, 2H). ^{13}C NMR (126 MHz, DMSO-d_6) δ 161.5, 119.9, 114.8, 47.8, 46.7, 19.6. HRMS (ESI/TOF-Q) *m/z*: $[M]^+$ calcd for $C_6H_8N_2O_2S_2$, 204.0027; found 204.0027.

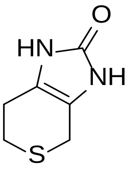

3,4,6,7-Tetrahydrothiopyrano[3,4-d]imidazol-2(1H)-one 10b

M.p. ≥250 °C, a beige powder, 37.1 g (87.8% yield). ^{1}H NMR (400 MHz, DMSO-d_6) δ 11.76 (s, 2H), 3.39 (s, 2H), 2.81 (t, *J* = 5.5 Hz, 2H), 2.54-2.52 (m, 2H). ^{13}C NMR (126 MHz, DMSO-d_6) δ 153.4, 115.4, 111.5, 24.5, 22.1, 20.5. HRMS (ESI/TOF-Q) *m/z*: $[M]^+$ calcd for $C_6H_8N_2OS$, 156.0357; found 156.0360.

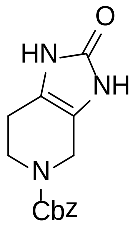

Benzyl 2-oxo-2,3,6,7-tetrahydro-1H-imidazo[4,5-c]pyridine-5(4H)-carboxylate 10c

M.p. 228-230°C, a white powder, 44.9 g (92.3% yield). ^{1}H NMR (500 MHz, DMSO-d_6) δ 9.68-9.54 (m, 2H), 7.40-7.33 (m, 5H), 5.09 (s, 2H), 4.12 (s, 2H), 3.62 (s, 2H), 2.29 (s, 2H). ^{13}C NMR (100 MHz, DMSO-d_6) δ 155.0, 137.3, 128.9, 128.4, 128.1, 113.9, 112.3, 67.0, 41.7, 41.3, 21.5, 21.0. HRMS (ESI/TOF-Q) *m/z*: $[M]^+$ calcd for $C_{14}H_{15}N_3O_3$, 273.1113; found 273.1115.

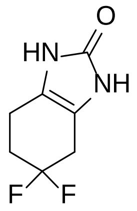

5,5-Difluoro-4,5,6,7-tetrahydro-1H-benzo[d]imidazol-2(3H)-one 10d

M.p. ≥200°C decomposition, a brown powder, 29.6 g (72.1% yield). ^{1}H NMR (400 MHz, DMSO-d_6) δ 9.71 (s, 1H), 9.64 (s, 1H), 2.82 (t, *J* = 14.0 Hz, 2H), 2.41-2.38 (m, 2H), 2.21-2.11 (m, 2H). ^{13}C NMR (100 MHz, DMSO-d_6) δ 155.5, 123.9 (t, J_{CF} =*240.0* Hz), 113.6, 110.6, 31.6 (t, J_{CF} =28.0 Hz), 30.4 (t, J_{CF} =25.0 Hz), 18.3 (t, J_{CF} =*6.0* Hz). ^{19}F (376 MHz, DMSO-d_6) δ -94.09. HRMS (ESI/TOF-Q) *m/z*: $[M]^+$ calcd for $C_7H_8F_2N_2O$, 174.0605; found 174.0608.

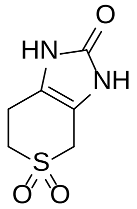

3,4,6,7-tetrahydrothiopyrano[3,4-d]imidazol-2(1H)-one 5,5-dioxide **10e**

M.p. ≥230°C, a yellow powder, 36.4 g (90.7% yield). ^{1}H NMR (400 MHz, DMSO-d_6) δ 9.89 (s, 1H), 9.71 (s, 1H), 3.99 (s, 2H), 3.33 (br.s, 2H), 2.71-2.68 (m, 2H). ^{13}C NMR (100 MHz, DMSO-d_6) δ 154.9, 113.3, 107.6, 48.5, 47.6, 20.7. HRMS (ESI/TOF-Q) *m/z*: $[M]^+$ calcd for $C_6H_8N_2O_3S$, 188.0256; found 188.0259.

General method for desulfurization of 9a-e.

To a stirring suspension of **9a** (50 g, 0.32 mol, 1 eq.) in ethanol (300 mL) was added Raney nickel (90 g, 4.8 eq.) in ethanol (300 mL). The resulting suspension was refluxed for 24 hours, cooled to room temperature and filtered off. Volatiles were removed under reduced pressure to give crude **11a** as solid. The crude product was triturated with ether, filtered off, the filter cake was washed with ether (3×100 mL) and dried under reduced pressure to afford **11a**.

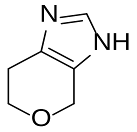

3,4,6,7-Tetrahydropyrano[3,4-d]imidazole 11a

M.p. 166°C, a white powder, 35.8 g (90.3% yield). ^{1}H NMR (400 MHz, DMSO-d_6) δ 11.66 (br.s, 1H), 7.48 (s, 1H), 4.52 (br.s, 2H), 3.82 (t, *J* = 5.4 Hz, 2H), 2.62-2.59 (m, 2H). ^{13}C NMR (126 MHz, DMSO-d_6) δ 133.7, 130.0, 123.9, 64.4, 64.3, 23.2. HRMS (ESI/TOF-Q) *m/z*: $[M]^+$ calcd for $C_6H_8N_2O$, 124.0637; found 124.0636.

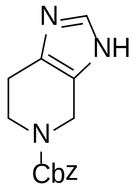

Benzyl 6,7-dihydro-3H-imidazo[4,5-c]pyridine-5(4H)-carboxylate 11c

A brown oil, 36.9 g (93.2 % yield). ^{1}H NMR (500 MHz, DMSO-d_6) δ 11.84 (br.s, 1H), 7.48 (s, 1H), 7.38-7.30 (m, 5H), 5.10 (s, 2H), 4.41-4.36 (m, 2H), 3.67 (s, 2H), 2.60-2.56 (m, 2H). ^{13}C NMR (100 MHz, DMSO-d_6) δ 155.4, 137.4, 134.8, 128.9, 128.4, 128.1, 66.9, 43.1, 42.3, 42.0, 22.7, 22.4. HRMS (ESI/TOF-Q) *m/z*: $[M]^+$ calcd for $C_{14}H_{15}N_3O_2$, 257.1164; found 257.1170.

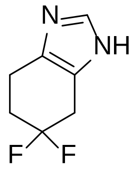

6,6-Difluoro-4,5,6,7-tetrahydro-1H-benzo[d]imidazole 11d

M.p. 165°C, a brown powder, 24.8 g (83.7 % yield). ^{1}H NMR (400 MHz, DMSO-d_6) δ 11.77 (br.s, 1H), 7.51 (s, 1H), 3.07 (t, J = 13.0 Hz, 2H), 2.67 (br.s, 2H), 2.26-2.20 (m, 2H). ^{13}C NMR (126 MHz, DMSO-d_6) δ 135.1 (s), 126.9 (br.s), 125.1 (br.s), 124.6 (t, J_{CF} =241 Hz), 33.5 (t, J_{CF} =27.4 Hz), 30.7 (t, J_{CF} =24.7 Hz), 18.4 (s). ^{19}F (376 MHz, DMSO-d_6) δ -94.65. HRMS (ESI/TOF-Q) *m/z*: $[M]^+$ calcd for $C_7H_8F_2N_2$, 158.0656; found 158.0654.

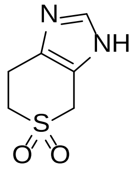

3,4,6,7-Tetrahydrothiopyrano[3,4-d]imidazole 5,5-dioxide 11e

M.p. 178-180°C, brown powder, 18.1 g (86.2 % yield). ^{1}H NMR (400 MHz, DMSO-d_6) δ 10.29 (br.s, 1H), 7.62 (s, 1H), 4.27 (s, 2H), 3.39-3.36 (m, 2H), 3.01-2.97 (m, 2H). ^{13}C NMR (126 MHz, DMSO-d_6) δ 158.6, 135.7, 124.5, 122.9, 51.3, 47.4, 20.4. HRMS (ESI/TOF-Q) *m/z*: $[M]^+$ calcd for $C_6H_8N_2O_2S$, 172.0306; found 172.0308.

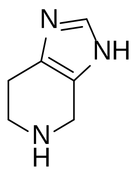

4,5,6,7-Tetrahydro-3H-imidazo[4,5-c]pyridine 12c

The compound was prepared according to the method for **8.**

M.p. 271-274°C, a yellow powder, 20.8 g (69.3% yield). ^{1}H NMR (400 MHz, DMSO-d_6) δ 10.30 (br.s, 2H), 9.07 (s, 1H), 4.25 (m, 2H), 3.42-3.39 (m, 2H), 2.96-2.94 (m, 2H). ^{13}C NMR (126 MHz, DMSO-d_6) δ 134.2, 125.0, 121.3, 40.7, 38.6, 18.4. HRMS (ESI/TOF-Q) *m/z*: $[M]^+$ calcd for $C_6H_9N_3$, 123.0796; found 123.0797.

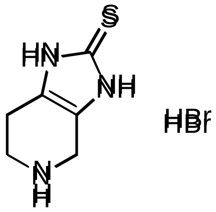

4,5,6,7-Tetrahydro-1H-imidazo[4,5-c]pyridine-2(3H)-thione hydrobromide 13c

To a stirred solution of **11c** (20.0 g, 69.1 mmol) in glacial acetic acid (40 mL) was added HBr in acetic acid (40%, 20 mL) and the resulting solution was stirred at room temperature for 2 hours. Upon completion, the reaction mixture was diluted with toluene (300 mL). The ensuing oil was decanted and triturated with ethanol (120 mL). The solid was filtered and washed with ethanol (3×70 mL), and dried under reduced pressure to afford **13c.**

M.p. 261-264°C, a beige powder, 6.9 g (42.6% yield). ^{1}H NMR (400 MHz, DMSO-d_6) δ 12.06 (s, 1H), 11.96 (s, 1H), 9.13 (br.s, 2H), 3.99 (s, 2H), 3.36-3.34 (m, 2H), 2.65-2.61 (m, 2H). ^{13}C NMR (126 MHz, DMSO-d_6) δ 161.0, 120.0, 116.0, 40.6, 38.2, 17.8. HRMS (ESI/TOF-Q) *m/z*: $[M]^+$ calcd for $C_6H_9N_3S$, 155.0517; found 155.0516.

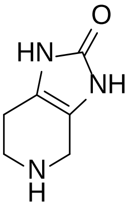

4,5,6,7-Tetrahydro-1H-imidazo[4,5-c]pyridin-2(3H)-one 14c

The compound was prepared according to the method for **8.**

M.p. 298-301°C, a white powder, 38.7 g (95.7% yield). ^{1}H NMR (400 MHz, DMSO-d$_6$) δ 9.48 (s, 1H), 9.42 (s, 1H), 3.31 (s, 2H), 2.84-2.82 (m, 2H), 2.23 (br.s, 1H), 2.15-2.11 (m, 2H). ^{13}C NMR (150 MHz, DMSO-d$_6$) δ 154.8, 115.4, 114.2, 43.0, 40.6, 22.1. HRMS (ESI/TOF-Q) *m/z*: [M]$^+$ calcd for $C_6H_9N_3O$, 139.0746; found 139.0744.

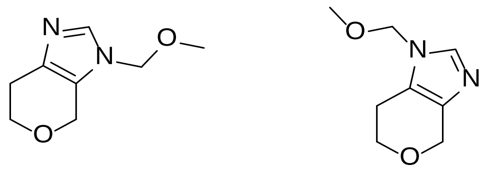

3-(Methoxymethyl)-3,4,6,7-tetrahydropyrano[3,4-d]imidazole 15 and 1-(Methoxymethyl)-1,4,6,7-tetrahydropyrano[3,4-d]imidazole mixture 15'

To a stirred suspension of **11a** (20 g, 0.16 mol, 1 eq.) in CH_3CN (150 mL) was added DIPEA (43.9 g, 0.34 mol, 2.1 eq.). To the reaction mixture was added MOM-Cl (13.7 g, 0.17 mol, 1.05 eq.) dropwise at 5°C. The resulting solution was stirred at room temperature for 24 hours. Upon completion, volatiles were removed under a reduced pressure and the resulting oil was extracted with MTBE (5×70 mL). The combined organics were concentrated under a reduce pressure to give a mixture **15** and **15'**.

A yellow oil, 11.8 g (43.6% yield). ^{1}H NMR (400 MHz, DMSO-d$_6$) δ 7.65, 7.63 (s, 1H), 5.22, 5.17 (br.s, 2H), 4.61, 4.47 (br.s, 2H), 3.83-3.81 (m, 2H), 3.16, 3.14 (s, 3H), 2.63-2.62, 2.55-2.54 (m, 2H). ^{13}C NMR (126 MHz, DMSO-d$_6$) δ 136.9, 136.8, 134.8, 133.7, 123.6, 122.9, 75.3, 75.3, 74.8, 65.0, 64.9, 64.0, 61.6, 55.2, 25.2, 21.5. LCMS (ES-API) m/z = 169.2 (M+H)$^+$, 170.2 (M+H)$^+$.

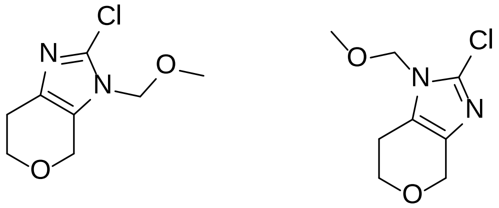

2-Chloro-3-(methoxymethyl)-3,4,6,7-tetrahydropyrano[3,4-d]imidazole 16 and **2-Chloro-1-(methoxymethyl)-1,4,6,7-tetrahydropyrano[3,4-d]imidazole 16'**

The flame-dried flask was charged with a mixture of **15** and **15'** (5.0 g, 29.7 mmol, 1 eq.) and THF (75 mL). The reaction mixture was cooled to -78°C under argon atmosphere; BuLi (12.5 mL 2.5 M in hexane, 1.05 eq.) was added. The reaction mixture was kept stirring for 30 minutes at -78 °C. At the same temperature C_2Cl_6 (7.4 g, 31.2 mmol, 1.05 eq.) in THF (50 mL) was added. The reaction mixture was allowed to warm to rt and was kept stirring for 12 hours. The reaction mixture was diluted with water (100 mL) and extracted with EtOAc (3×50 mL). The combined organic washings were dried over Na_2SO_4 and evaporated under a reduced pressure to afford a crude mixture of **16** and **16'** that was used in the next step without any purification.

A yellow oil, 4.6 g (76.6% yield). ^{1}H NMR (400 MHz, DMSO-d$_6$) δ 5.21, 5.16 (br.s, 2H), 4.60, 4.43 (br.s, 2H), 3.87-3.86, 3.85-3.84 (m, 2H), 3.23, 3.21 (s, 3H), 2.67-2.64, 2.53-2.50 (m, 2H). ^{13}C NMR (126 MHz, DMSO-d$_6$) δ 133.7, 132.4, 130.0, 129.9, 126.3, 125.7, 79.2, 74.6, 74.1, 64.9, 64.1, 63.9, 61.3, 55.8, 24.6, 21.6. MS-EI: m/z 45 (100%), 202 (10%), 172 (10%), 142 (7%).

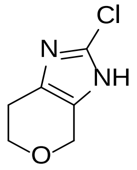

2-Chloro-3,4,6,7-tetrahydropyrano[3,4-d]imidazole 17

The mixture **16** and **16'** (4.6 g, 22.7 mmol) was dissolved in 4 N HCl (25 mL) and the resulting solution was heated at 45˚C for 24 hours. Upon completion, the reaction mixture was allowed to cool to room temperature and evaporated till

dryness under a reduced pressure. The residue was dissolved in DCM (150 mL) and washed with K_2CO_3 solution (20% in water, 3×50 mL). The organic layer was dried over Na_2SO_4 and evaporated under a reduced pressure to afford an oil. The crude product was purified by flash chromatography (MTBE/MeOH, SiO_2) to give **17.**

A white powder, 1.7 g (57% yield). ^{1}H NMR (400 MHz, $CDCl_3$) δ 4.62 (br.s, 2H), 3.96 (t, J = 5.4 Hz, 2H), 2.70 (t, J = 5.4 Hz, 2H). ^{13}C NMR (126 MHz, $CDCl_3$) δ 64.8, 64.2, 23.1. HRMS (ESI/TOF-Q) *m/z*: $[M]^+$ calcd for $C_6H_7ClN_2O$, 158.0247; found 158.0245.

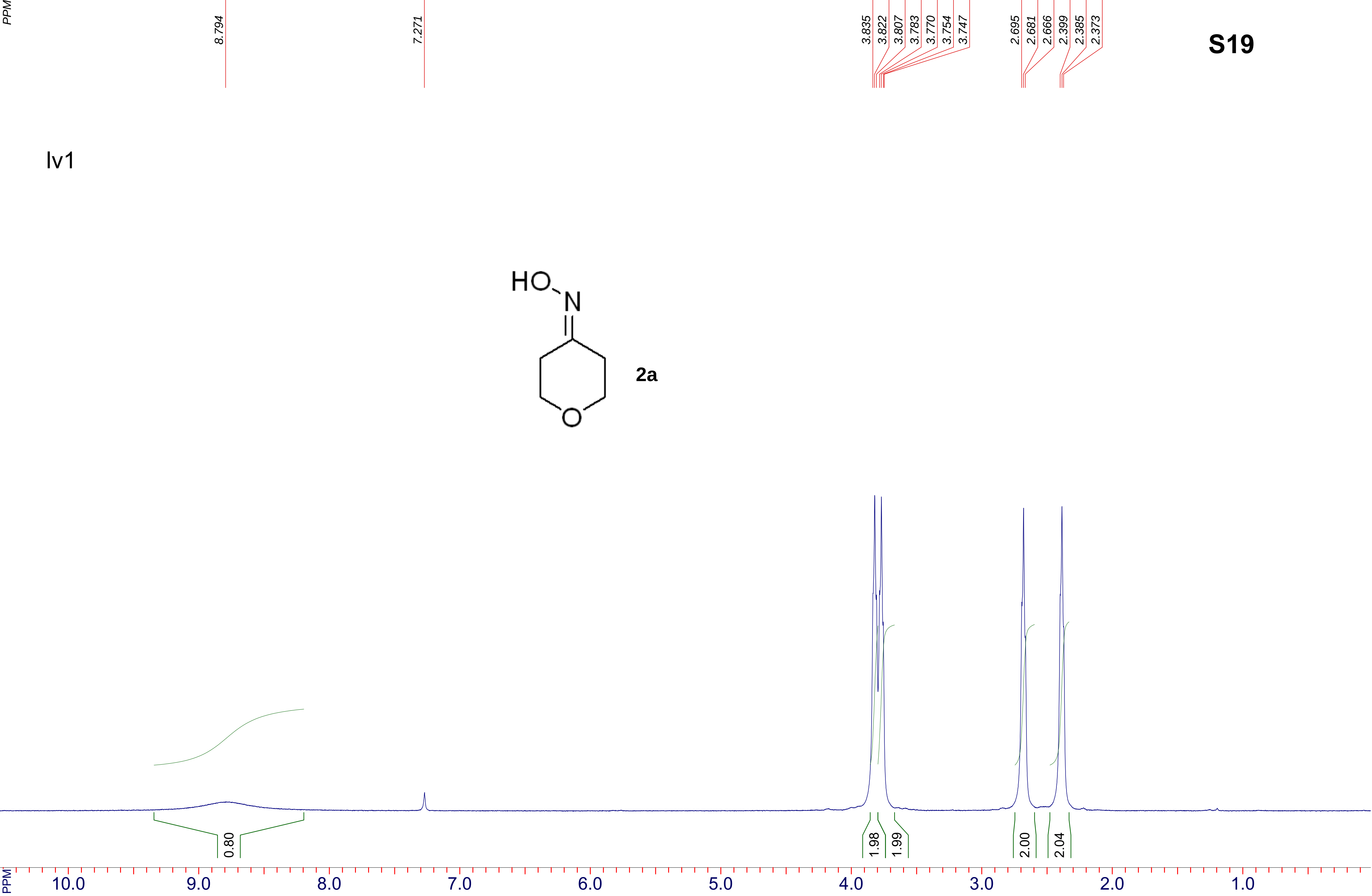

**File name: lv1**	Operator: nmr	SF: 400.1300 MHz	NSC: 1	PW: 0.00 usec, RG: 25	SI: 32768
Date: 19-Nov-2022	Solvent: CDCl3	SW: 8224 Hz	TE: 300 K	AQ: 1.99 sec, RD: 0.00 sec	

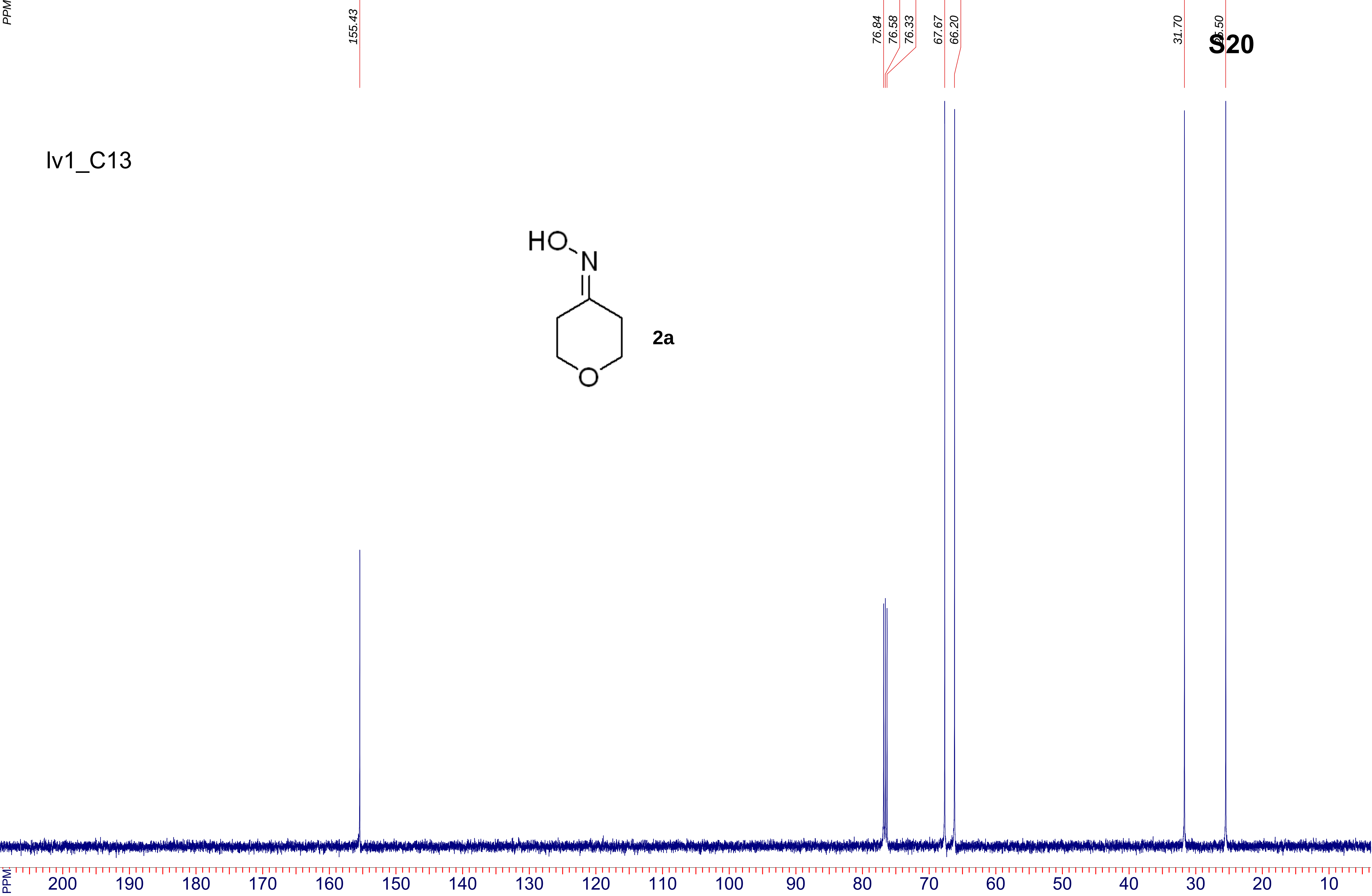

**File name: lv1_C13**	Operator: root	SF: 125.6429 MHz	NSC: 228	PW: 0.00 usec, RG: 51200	SI: 131072
Date: 23-Nov-2022	Solvent: CDCl3	SW: 32680 Hz	TE: 683 K	AQ: 0.78 sec, RD: 0.00 sec	

Target Compound Screening Report

**Data File**	1.d	**Sample Name**	1
**Sample Type**	Sample	**Position**	P1-A1
**Instrument Name**	Instrument 1	**User Name**	Denis V.Bylina
**Acq Method**	Fast_Gradient_HRMS_pos_Lock_08272019.m	**Acquired Time**	12/19/2022 12:50:33 PM (UTC+02:00)
**IRM Calibration Status**	Success	**DA Method**	1.m
**Comment**	Lysenko		
**Sample Group**		**Info.**	Agilent 6224 TOF LC/MS
**MFC**	C5H9NO2	**Stream Name**	LC 1
**Acquisition Time (Local)**	12/19/2022 12:50:33 PM (UTC+02:00)	**Acquisition SW Version**	6200 series TOF/6500 series Q-TOF B.08.00 (B8058.0)
**TOF Driver Version**	8.00.00	**TOF Firmware Version**	8.643
**Tune Mass Range Max.**	1700		

Compound Table

Label	Tgt Score	Mass Error (ppm)	Tgt Formula	Obs. RT	Ref. Mass	Obs. Mass
Cpd 1: C5 H9 N O2; 1.161	95.84	-0.37	C5 H9 N O2	1.161	115.0633	115.0633

Obs. *m/z*	Obs. RT	Obs. Mass	Tgt Formula	Tgt Mass	Tgt Mass Error (ppm)	RT Diff.	Find Cpds Algorithm
116.0704	1.161	115.0633	C5 H9 N O2	115.0633	-0.37	Find By Formula	

Compound Chromatograms

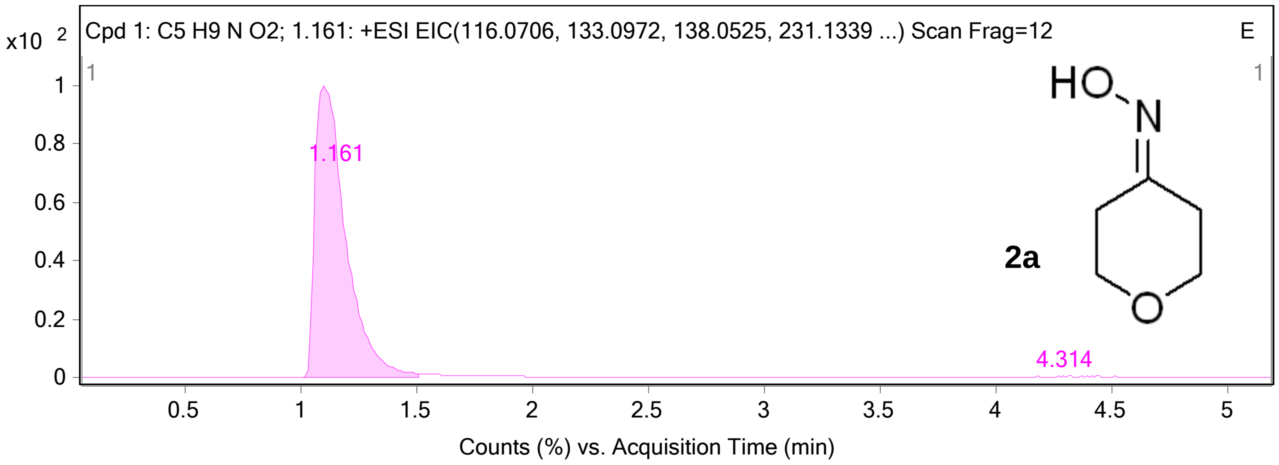

MS Zoomed Spectrum

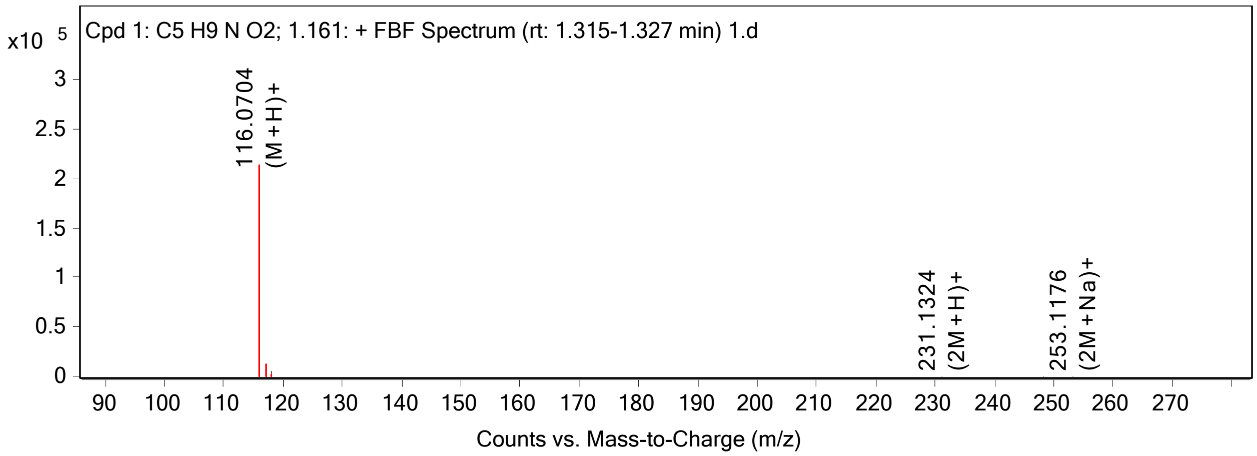

Target Compound Screening Report

MS Spectrum Peak List

Obs. *m/z*	Charge	Abund	Ion/Isotope
116.0704	1	211638.81	(M+H)+
117.0735	1	12021.54	(M+H)+
118.0844	1	4598.26	(M+H)+
231.1324	1	118.58	(2M+H)+
248.1674	1	82.35	(2M+NH4)+
253.1176	1	152.18	(2M+Na)+

MS Zoomed Spectrum

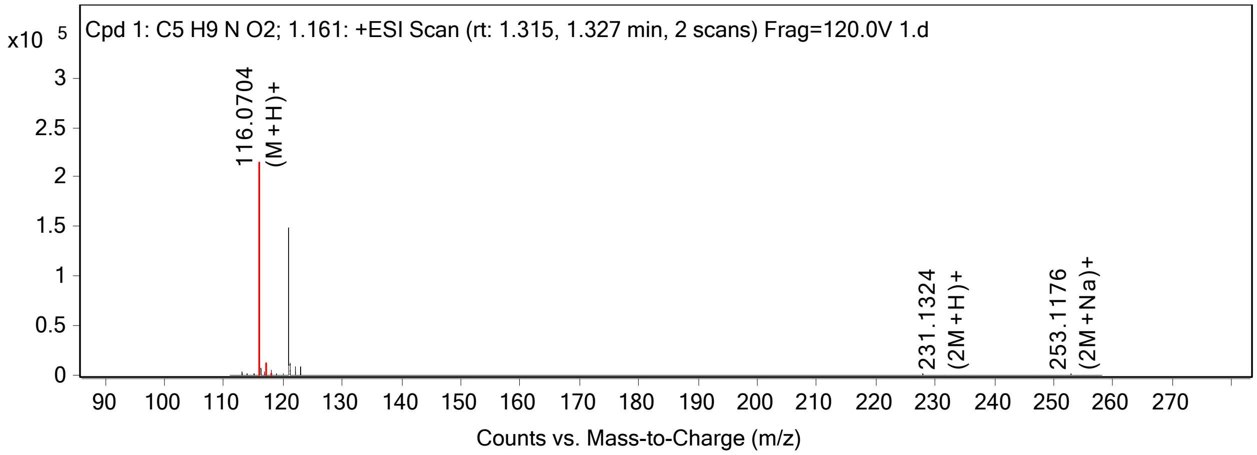

MS Spectrum Peak List

Obs. *m/z*	Charge	Abund	Ion/Isotope	Tgt Mass Error (ppm)
116.0704	1	211638.81	(M+H)+	2.06
117.0735	1	12021.54	(M+H)+	1.22
118.0844	1	4598.26	(M+H)+	-77.25
231.1324	1	118.58	(2M+H)+	6.64
248.1674	1	82.35	(2M+NH4)+	-27.74
253.1176	1	152.18	(2M+Na)+	-6.74

--- End Of Report ---

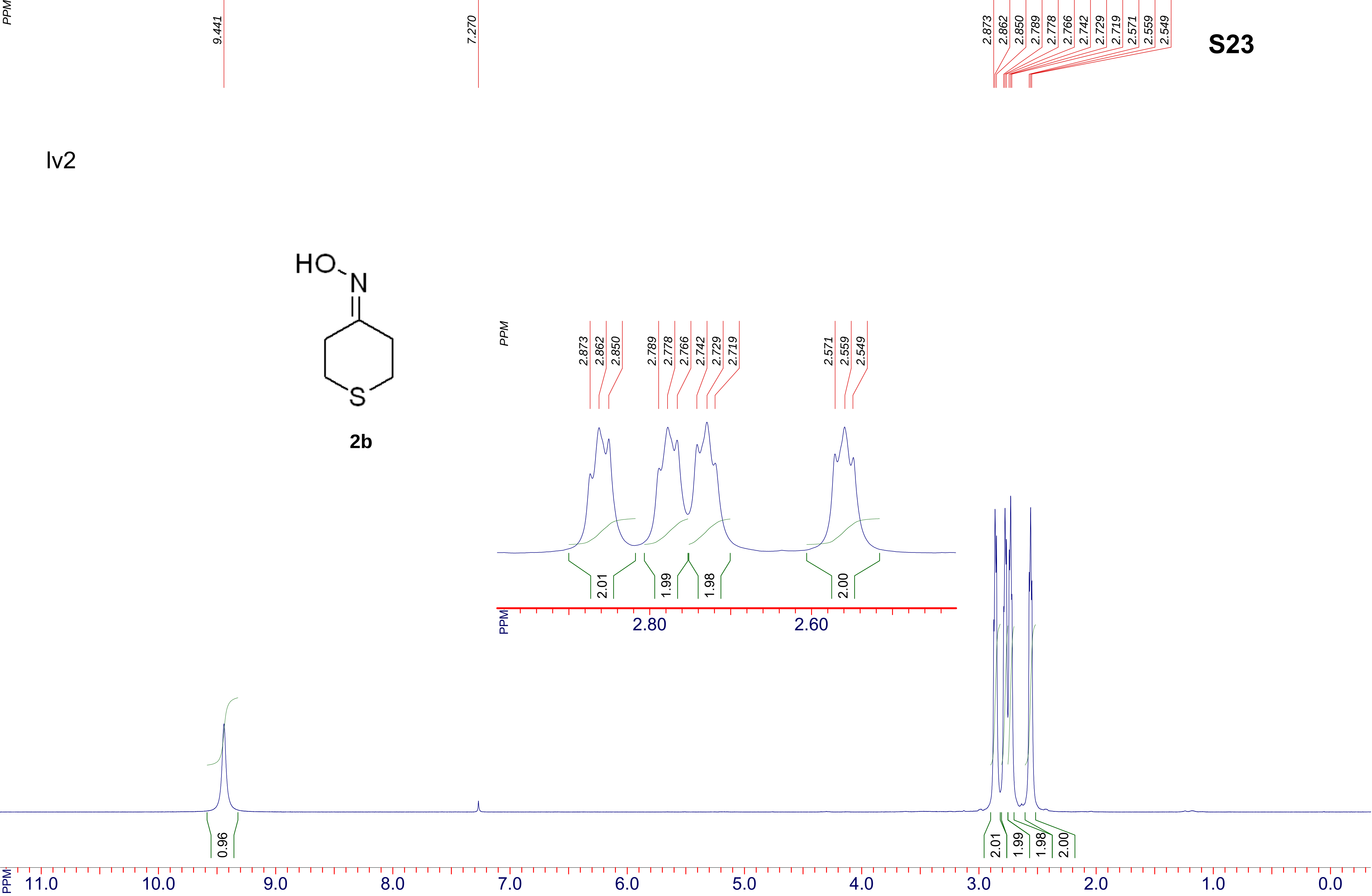

**File name: lv2**	Operator: root	SF: 499.6730 MHz	NSC: 1	PW: 0.00 usec, RG: 32	SI: 32768
Date: 06-Dec-2022	Solvent: CDCl3	SW: 8993 Hz	TE: 683 K	AQ: 1.82 sec, RD: 0.00 sec	

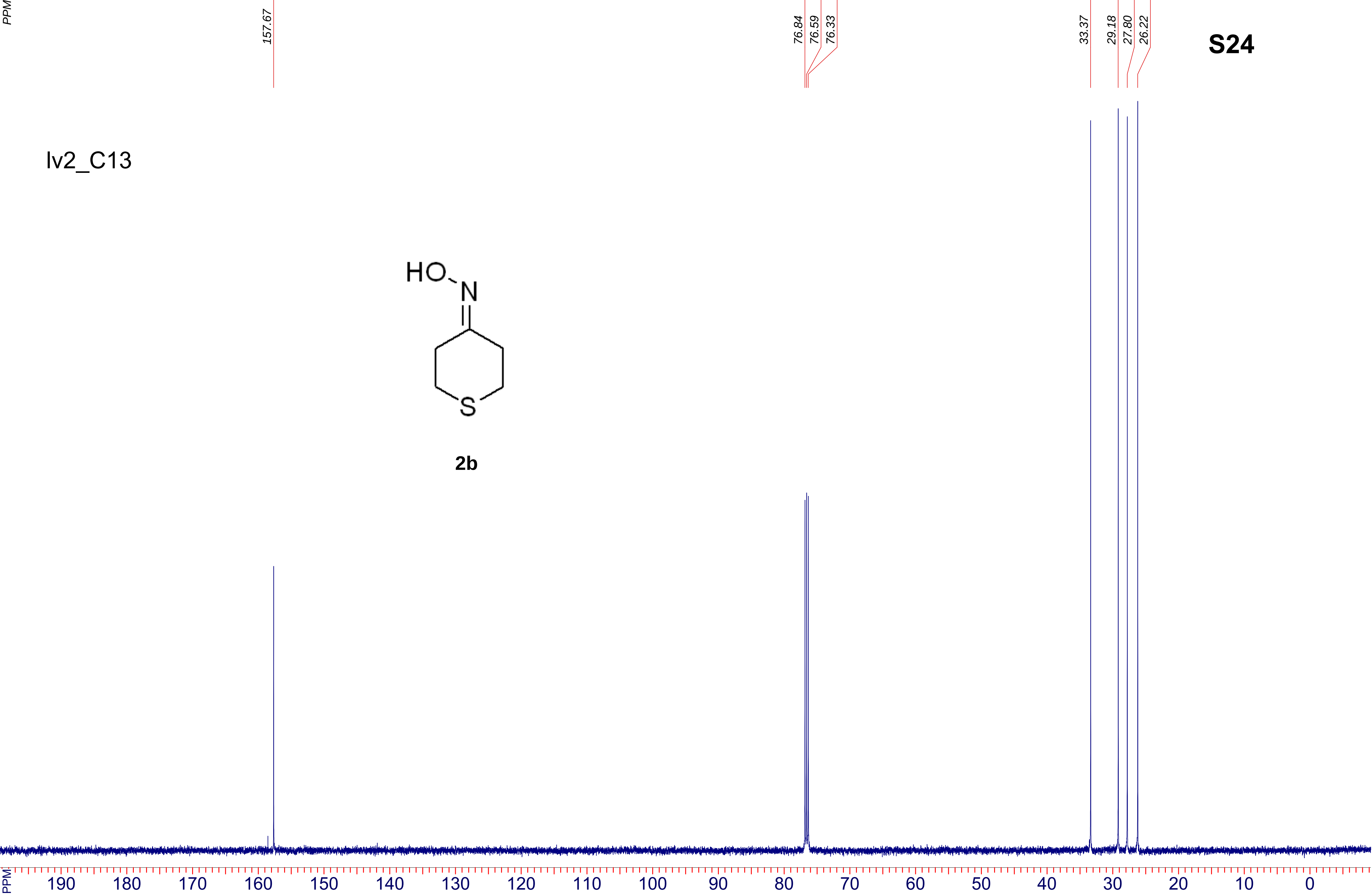

**File name: lv2_C13**	Operator: root	SF: 125.6429 MHz	NSC: 400	PW: 0.00 usec, RG: 51200	SI: 131072
Date: 06-Dec-2022	Solvent: CDCl3	SW: 32680 Hz	TE: 683 K	AQ: 0.78 sec, RD: 0.00 sec	

Target Compound Screening Report

**Data File**	2.d	**Sample Name**	2
**Sample Type**	Sample	**Position**	P1-A2
**Instrument Name**	Instrument 1	**User Name**	Denis V.Bylina
**Acq Method**	Fast_Gradient_HRMS_pos_Lock_08272019.m	**Acquired Time**	12/19/2022 12:56:27 PM (UTC+02:00)
**IRM Calibration Status**	Success	**DA Method**	1.m
**Comment**	Lysenko		
**Sample Group**		**Info.**	Agilent 6224 TOF LC/MS
**MFC**	C5H9NOS	**Stream Name**	LC 1
**Acquisition Time (Local)**	12/19/2022 12:56:27 PM (UTC+02:00)	**Acquisition SW Version**	6200 series TOF/6500 series Q-TOF B.08.00 (B8058.0)
**TOF Driver Version**	8.00.00	**TOF Firmware Version**	8.643
**Tune Mass Range Max.**	1700		

Compound Table

Label	Tgt Score	Mass Error (ppm)	Tgt Formula	Obs. RT	Ref. Mass	Obs. Mass
Cpd 1: C5 H9 N O S; 1.973	99.44	-2.43	C5 H9 N O S	1.973	131.0405	131.0402

Obs. *m/z*	Obs. RT	Obs. Mass	Tgt Formula	Tgt Mass	Tgt Mass Error (ppm)	RT Diff.	Find Cpds Algorithm
132.0475	1.973	131.0402	C5 H9 N O S	131.0405	-2.43	Find By Formula	

Compound Chromatograms

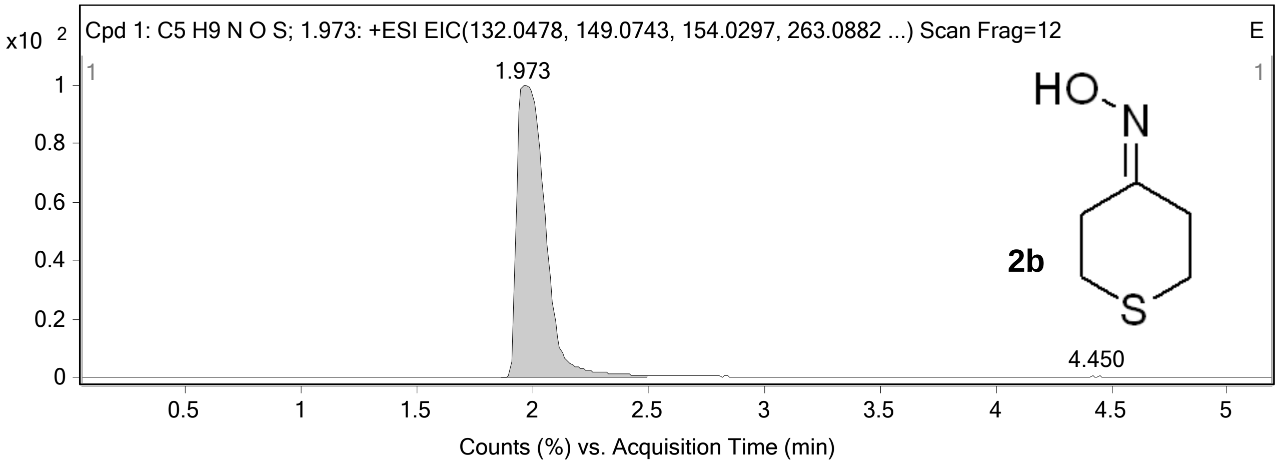

MS Zoomed Spectrum

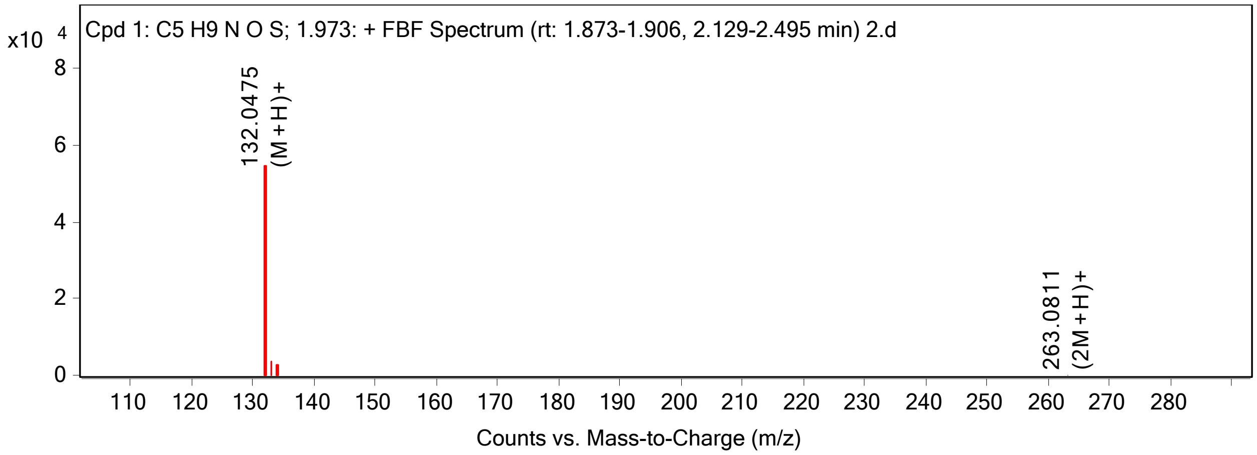

MS Spectrum Peak List

Obs. *m/z*	Charge	Abund	Ion/Isotope
132.0475	1	54615.63	(M+H)+
133.0504	1	3485.98	(M+H)+
134.0438	1	2521.09	(M+H)+
263.0811	1	198.86	(2M+H)+

MS Zoomed Spectrum

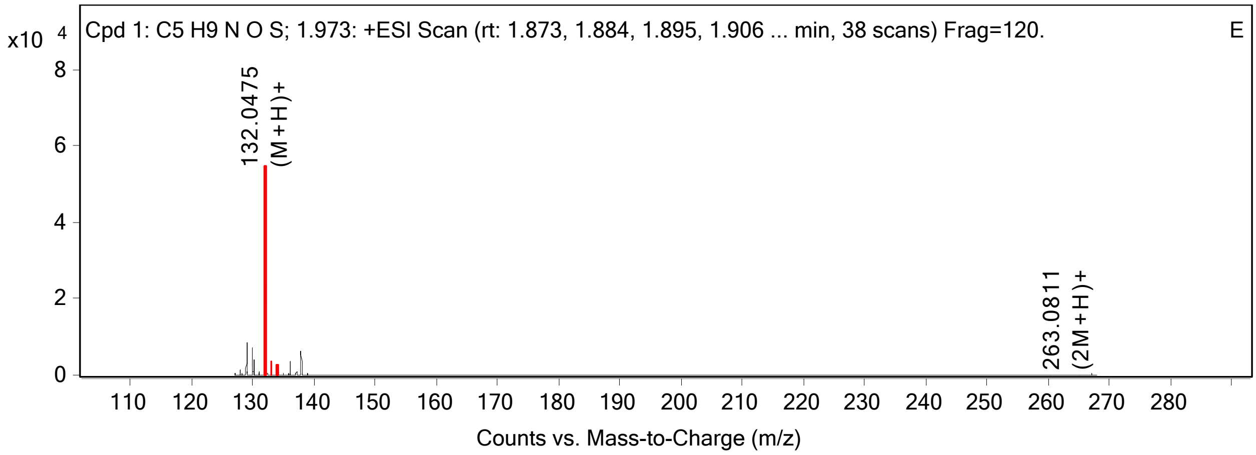

MS Spectrum Peak List

Obs. *m/z*	Charge	Abund	Ion/Isotope	Tgt Mass Error (ppm)
132.0475	1	54615.62	(M+H)+	2.35
133.0504	1	3485.98	(M+H)+	-0.41
134.0438	1	2521.09	(M+H)+	3.52
263.0811	1	198.86	(2M+H)+	27.14

--- End Of Report ---

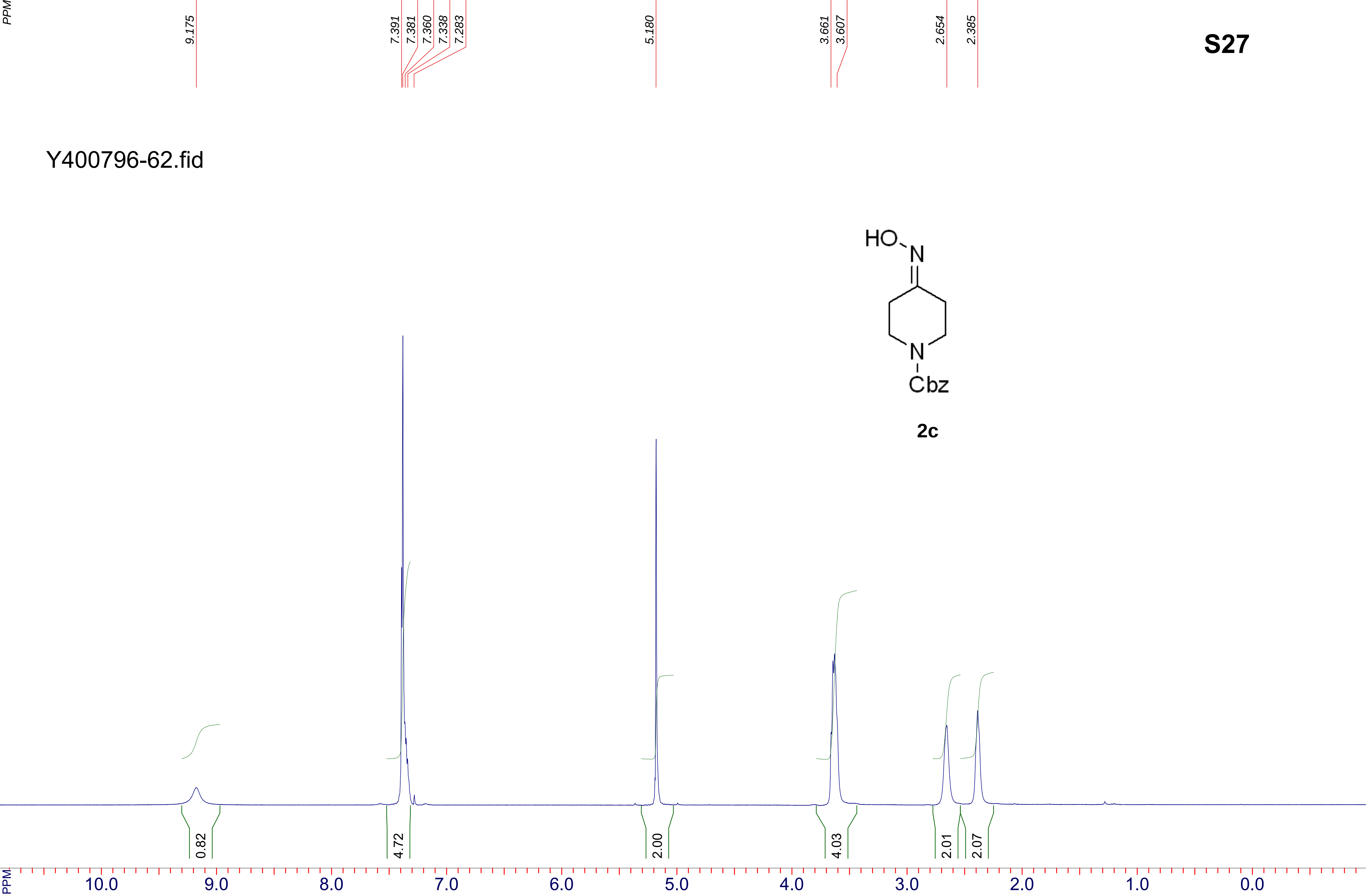

**File name: Y400796-62.fid**	Operator:	SF: 399.9734 MHz	NSC: 0	PW: 10.90 usec, RG: 24	SI: 32768
Date: 29-May-2023	Solvent: cdcl3	SW: 8000 Hz	TE: 298 K	AQ: 2.00 sec, RD: 0.00 sec	

S28

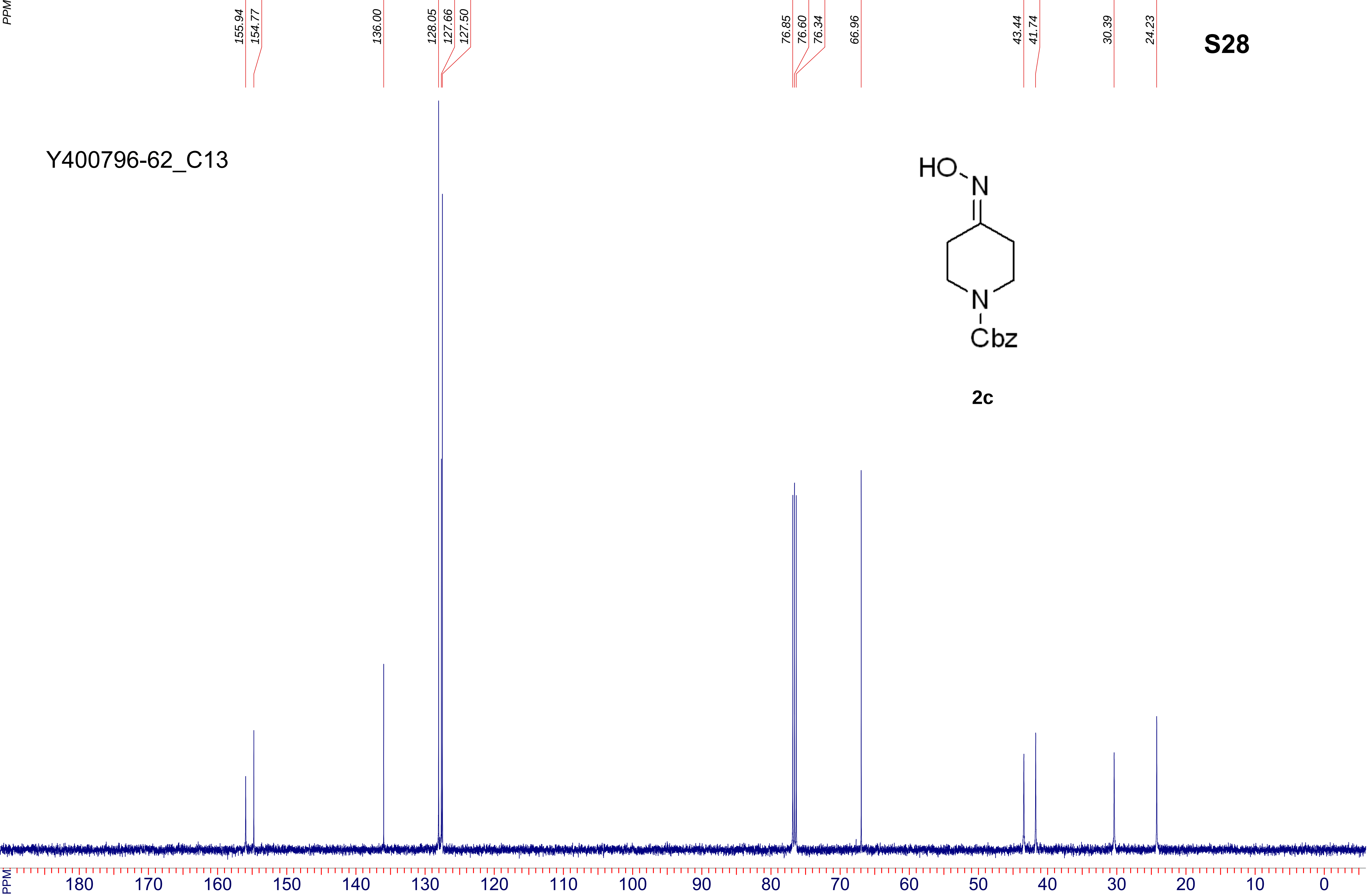

2c

**File name: Y400796-62_C13**	Operator: root	SF: 125.6429 MHz	NSC: 318	PW: 0.00 usec, RG: 51200	SI: 131072
Date: 29-May-2023	Solvent: CDCl3	SW: 32680 Hz	TE: 683 K	AQ: 0.78 sec, RD: 0.00 sec	

Target Compound Screening Report

**Data File**	10a.d	**Sample Name**	4
**Sample Type**	Sample	**Position**	P1-B1
**Instrument Name**	Instrument 1	**User Name**	Denis V.Bylina
**Acq Method**	Fast_Gradient_HRMS_pos_Lock_01312023.m	**Acquired Time**	7/10/2023 12:05:56 PM (UTC+03:00)
**IRM Calibration Status**	Success	**DA Method**	1.m
**Comment**	Lysenko		
**Sample Group**		**Info.**	Agilent 6224 TOF LC/MS
**MFC**	C13H16N2O3	**Stream Name**	LC 1
**Acquisition Time (Local)**	7/10/2023 12:05:56 PM (UTC+03:00)	**Acquisition SW Version**	6200 series TOF/6500 series Q-TOF B.08.00 (B8058.0)
**TOF Driver Version**	8.00.00	**TOF Firmware Version**	8.643
**Tune Mass Range Max.**	1700		

Compound Table

Label	Tgt Score	Mass Error (ppm)	Tgt Formula	Obs. RT	Ref. Mass	Obs. Mass
Cpd 3: C13 H16 N2 O3; 2.140	98.15	1.22	C13 H16 N2 O3	2.14	248.11609	248.11639

Obs. *m/z*	Obs. RT	Obs. Mass	Tgt Formula	Tgt Mass	Tgt Mass Error (ppm)	RT Diff.	Find Cpds Algorithm
249.12371	2.14	248.11639	C13 H16 N2 O3	248.11609	1.22	Find By Formula	

Compound Chromatograms

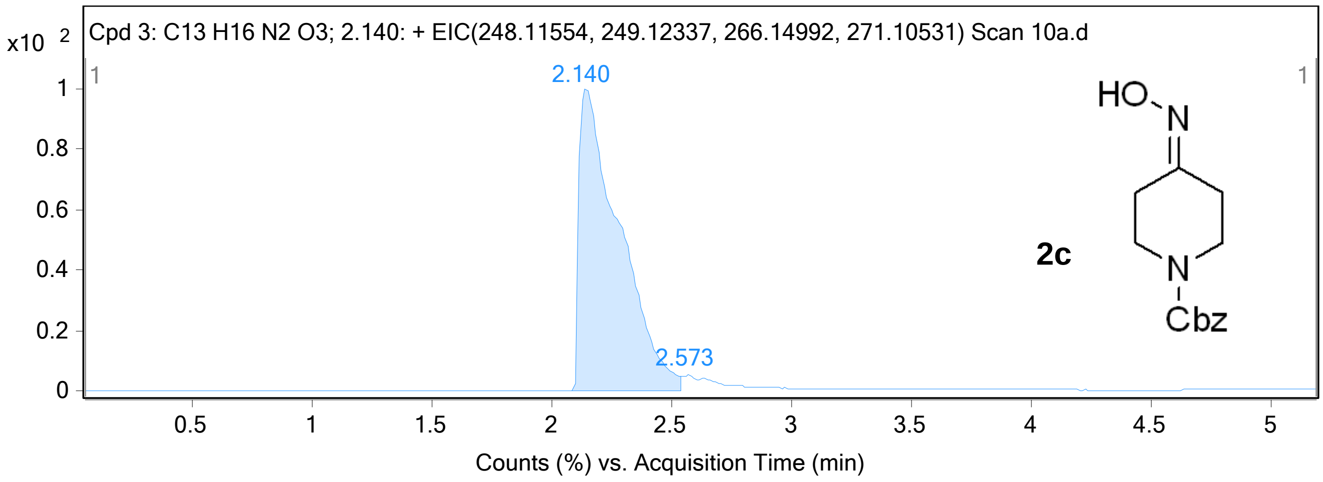

MS Zoomed Spectrum

Target Compound Screening Report

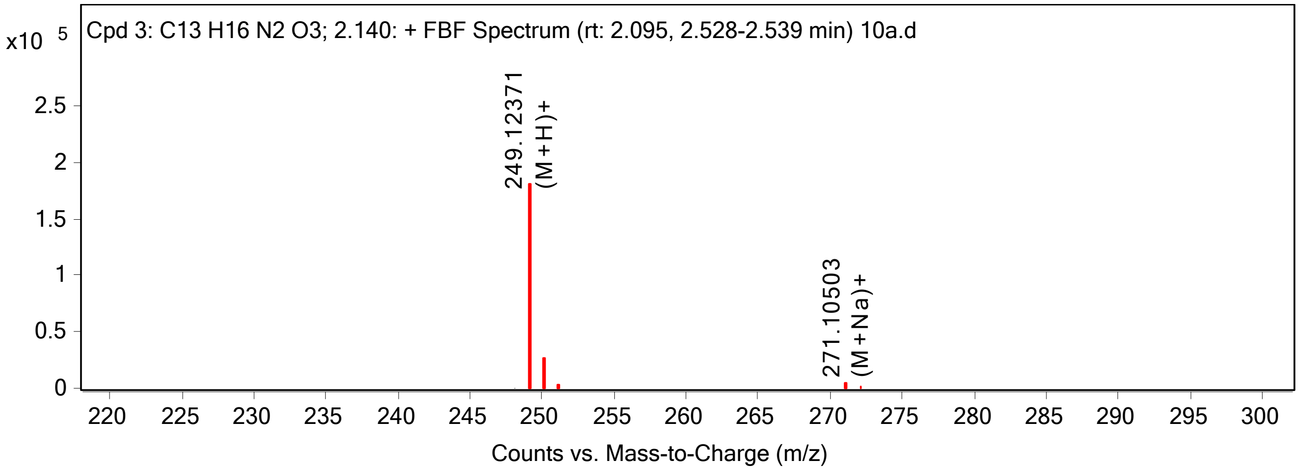

MS Spectrum Peak List

Obs. *m/z*	Charge	Abund	Ion/Isotope
248.11531	1	391.49	M+
249.12371	1	181241.59	(M+H)+
250.12612	1	26749.33	(M+H)+
251.13238	1	4009.57	(M+H)+
271.10503	1	5279.14	(M+Na)+
272.10852	1	874.71	(M+Na)+

MS Zoomed Spectrum

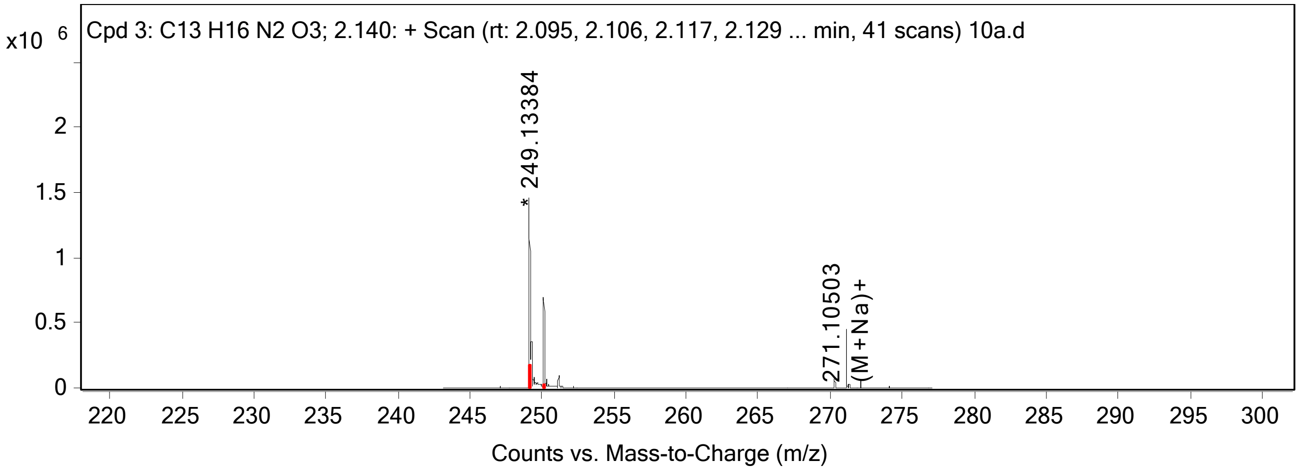

MS Spectrum Peak List

Obs. *m/z*	Charge	Abund	Ion/Isotope	Tgt Mass Error (ppm)
248.11531	1	391.49	M+	0.93
249.12371	1	181241.59	(M+H)+	-1.39
249.13384		1464650.49		
250.12612	1	26749.33	(M+H)+	1.37
251.13238	1	4009.57	(M+H)+	-14.12
271.10503	1	5279.14	(M+Na)+	1.04
272.10852	1	874.71	(M+Na)+	-0.43

--- End Of Report ---

lv4

2d

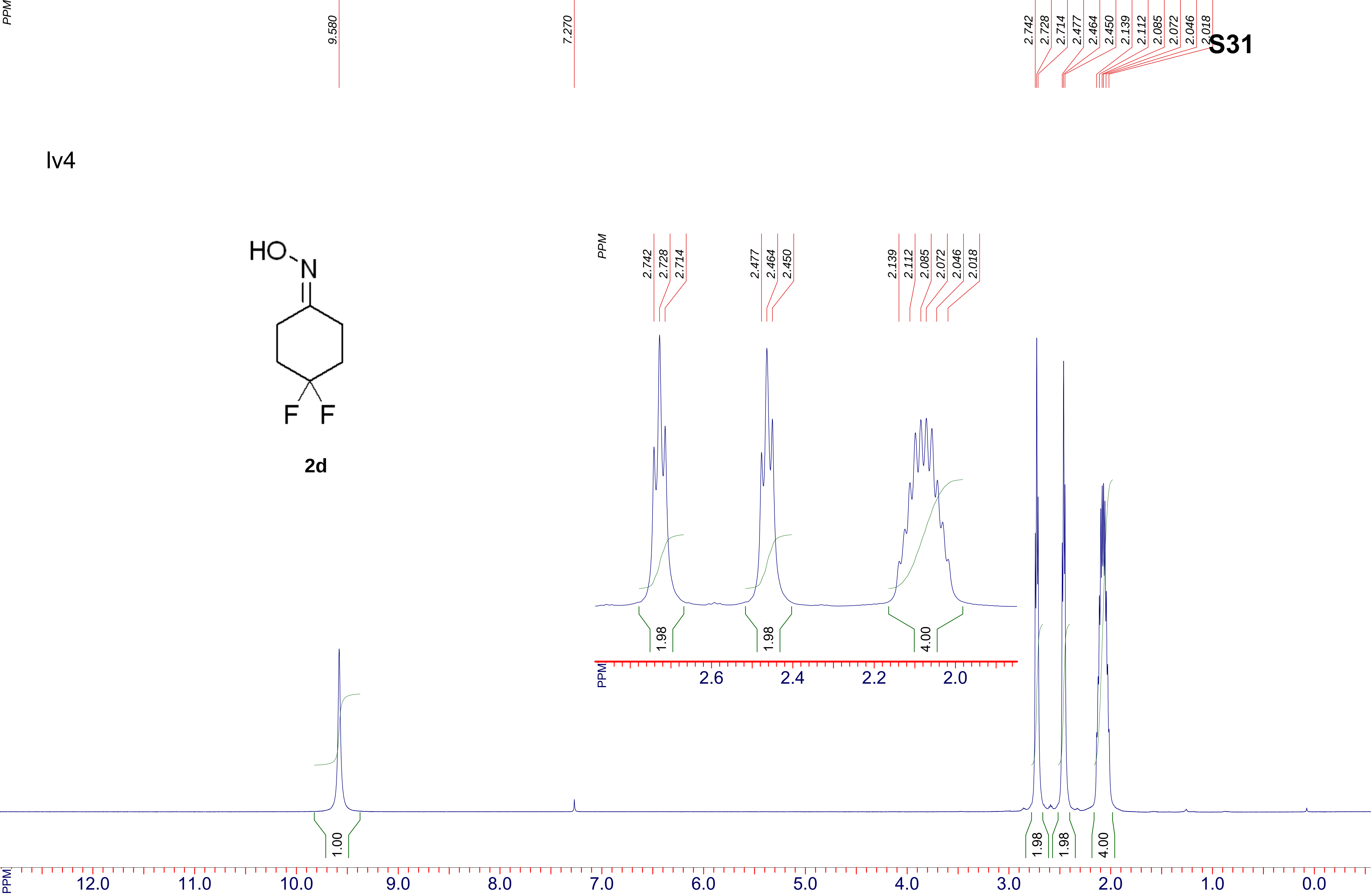

File name: lv4	Operator: root	SF: 499.6730 MHz	NSC: 1	PW: 0.00 usec, RG: 32	SI: 32768
Date: 06-Dec-2022	Solvent: CDCl3	SW: 8993 Hz	TE: 683 K	AQ: 1.82 sec, RD: 0.00 sec	

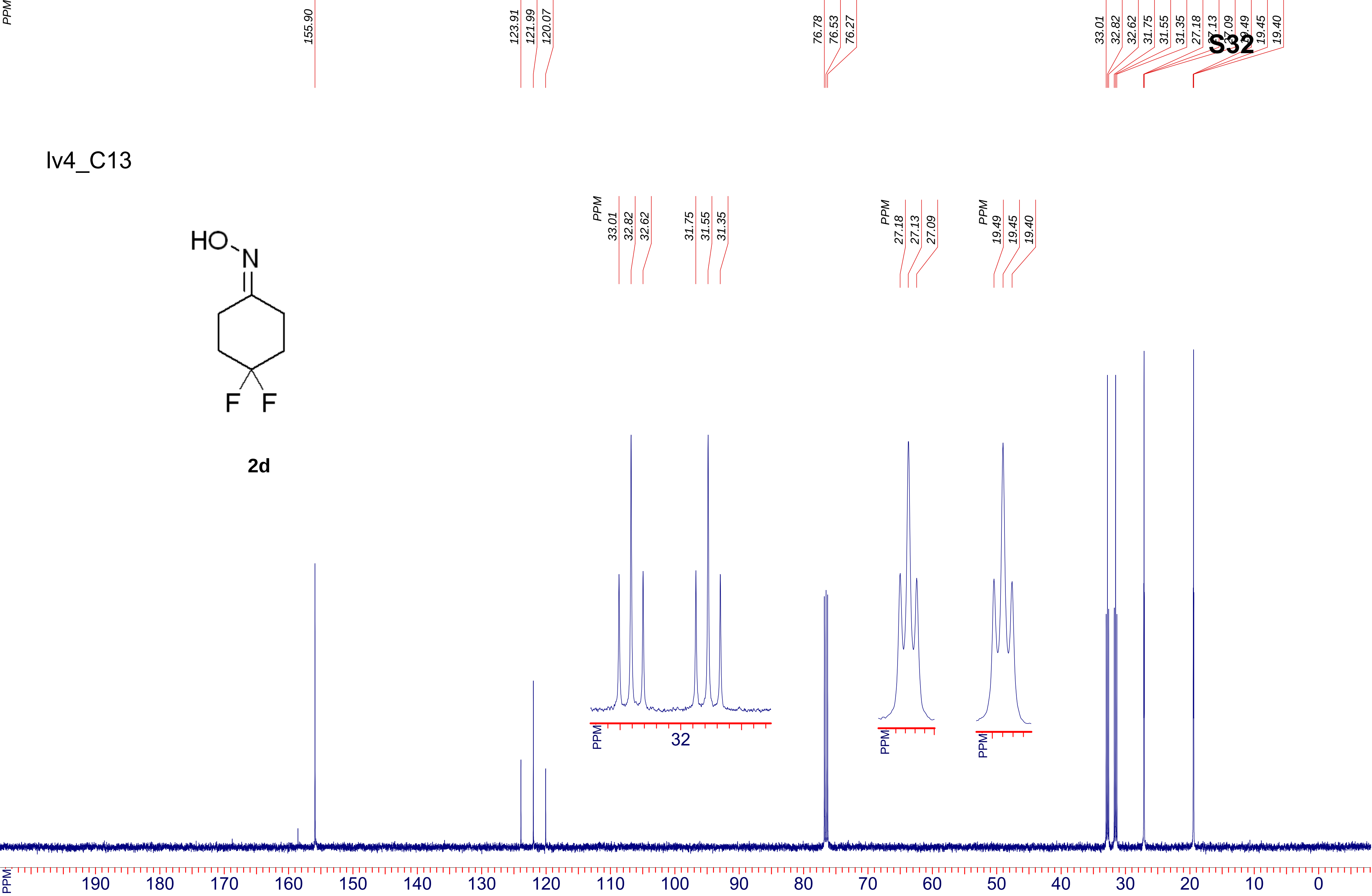

2d

**File name: lv4_C13**	Operator: root	SF: 125.6429 MHz	NSC: 400	PW: 0.00 usec, RG: 51200	SI: 131072
Date: 06-Dec-2022	Solvent: CDCl3	SW: 32680 Hz	TE: 683 K	AQ: 0.78 sec, RD: 0.00 sec	

lv4_F19{H}

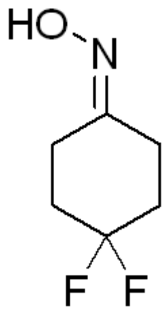

2d

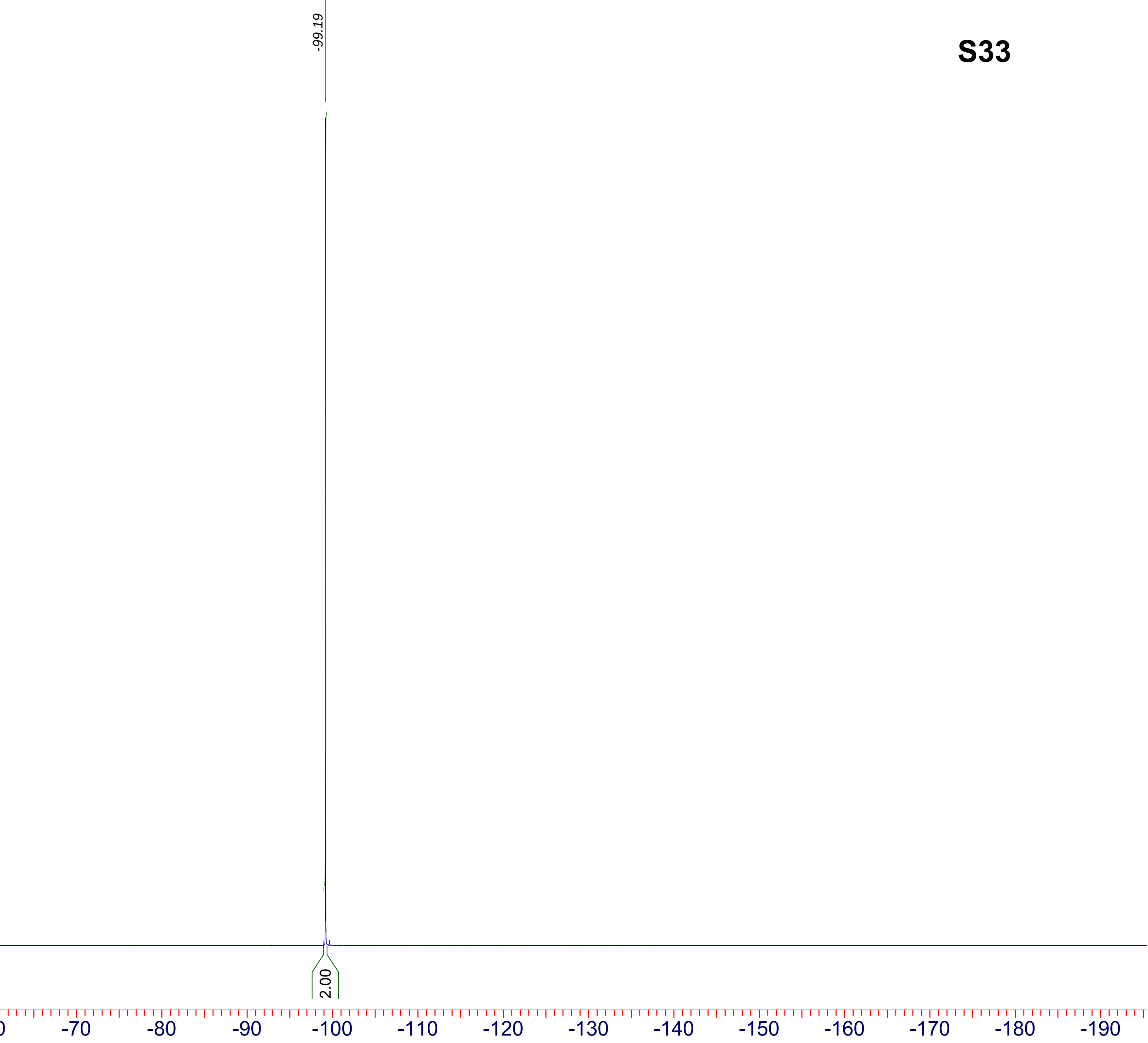

File name: lv4_F19{H}	Operator: nmr	SF: 376.4986 MHz	NSC: 1	PW: 0.00 usec, RG: 912	SI: 262144
Date: 06-Dec-2022	Solvent: CDCl3	SW: 138889 Hz	TE: 300 K	AQ: 0.94 sec, RD: 0.00 sec	Parameter file, TOPSPINVersion 2.1

Target Compound Screening Report

**Data File**	3.d	**Sample Name**	4
**Sample Type**	Sample	**Position**	P1-A3
**Instrument Name**	Instrument 1	**User Name**	Denis V.Bylina
**Acq Method**	Fast_Gradient_HRMS_pos_Lock_08272019.m	**Acquired Time**	12/19/2022 1:02:23 PM (UTC+02:00)
**IRM Calibration Status**	Success	**DA Method**	1.m
**Comment**	Lysenko		
**Sample Group**		**Info.**	Agilent 6224 TOF LC/MS
**MFC**	C6H9F2NO	**Stream Name**	LC 1
**Acquisition Time (Local)**	12/19/2022 1:02:23 PM (UTC+02:00)	**Acquisition SW Version**	6200 series TOF/6500 series Q-TOF B.08.00 (B8058.0)
**TOF Driver Version**	8.00.00	**TOF Firmware Version**	8.643
**Tune Mass Range Max.**	1700		

Compound Table

Label	Tgt Score	Mass Error (ppm)	Tgt Formula	Obs. RT	Ref. Mass	Obs. Mass
Cpd 1: C6 H9 F2 N O; 2.271	99.37	-1.41	C6 H9 F2 N O	2.271	149.0652	149.065

Obs. *m/z*	Obs. RT	Obs. Mass	Tgt Formula	Tgt Mass	Tgt Mass Error (ppm)	RT Diff.	Find Cpds Algorithm
150.0723	2.271	149.065	C6 H9 F2 N O	149.0652	-1.41	Find By Formula	

Compound Chromatograms

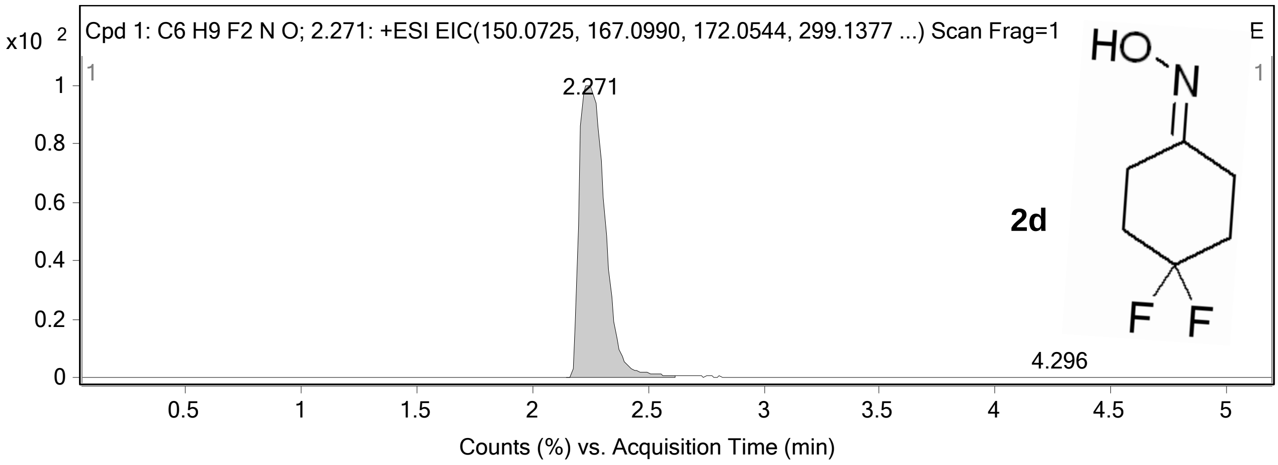

MS Zoomed Spectrum

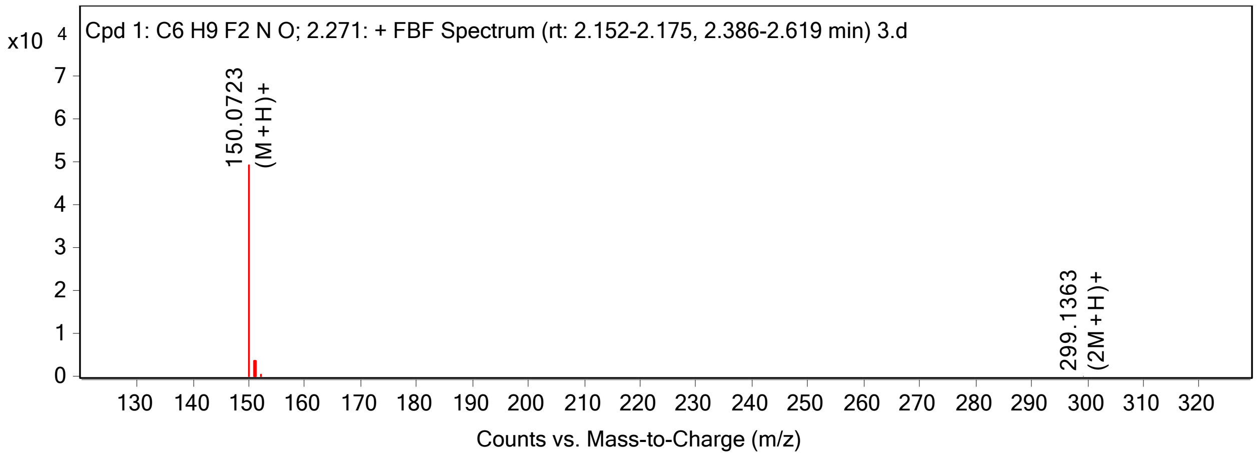

Target Compound Screening Report

MS Spectrum Peak List

Obs. *m/z*	Charge	Abund	Ion/Isotope
150.0723	1	48800.07	(M+H)+
151.076	1	3489.12	(M+H)+
152.0768	1	486.17	(M+H)+
299.1363	1	139.02	(2M+H)+

MS Zoomed Spectrum

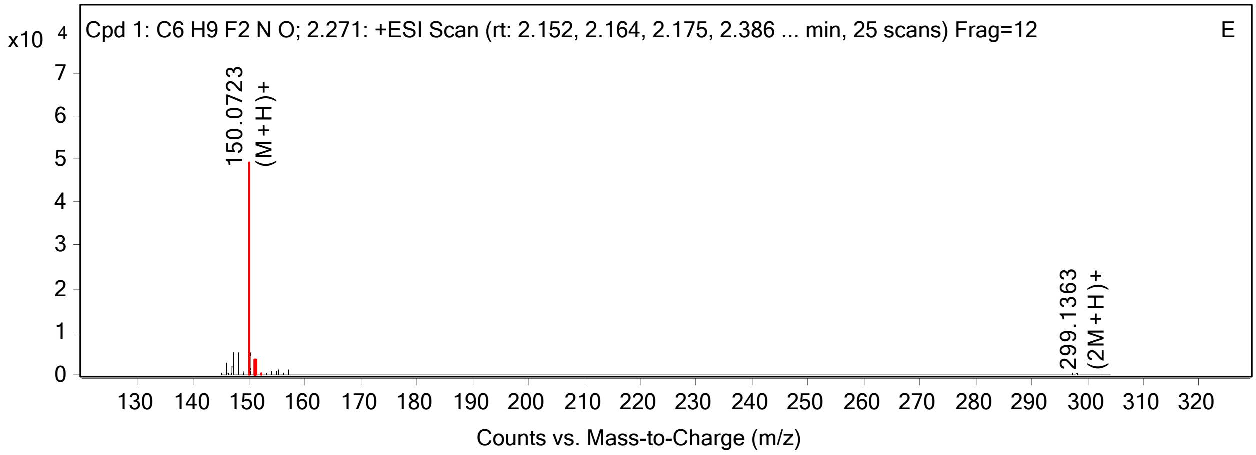

MS Spectrum Peak List

Obs. *m/z*	Charge	Abund	Ion/Isotope	Tgt Mass Error (ppm)
150.0723	1	48800.06	(M+H)+	1.62
151.076	1	3489.12	(M+H)+	-2.49
152.0768	1	486.17	(M+H)+	5.65
299.1363	1	139.02	(2M+H)+	4.78

--- End Of Report ---

S36

lv5

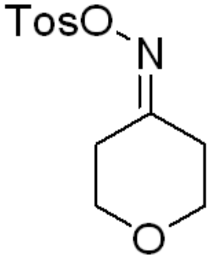

3a

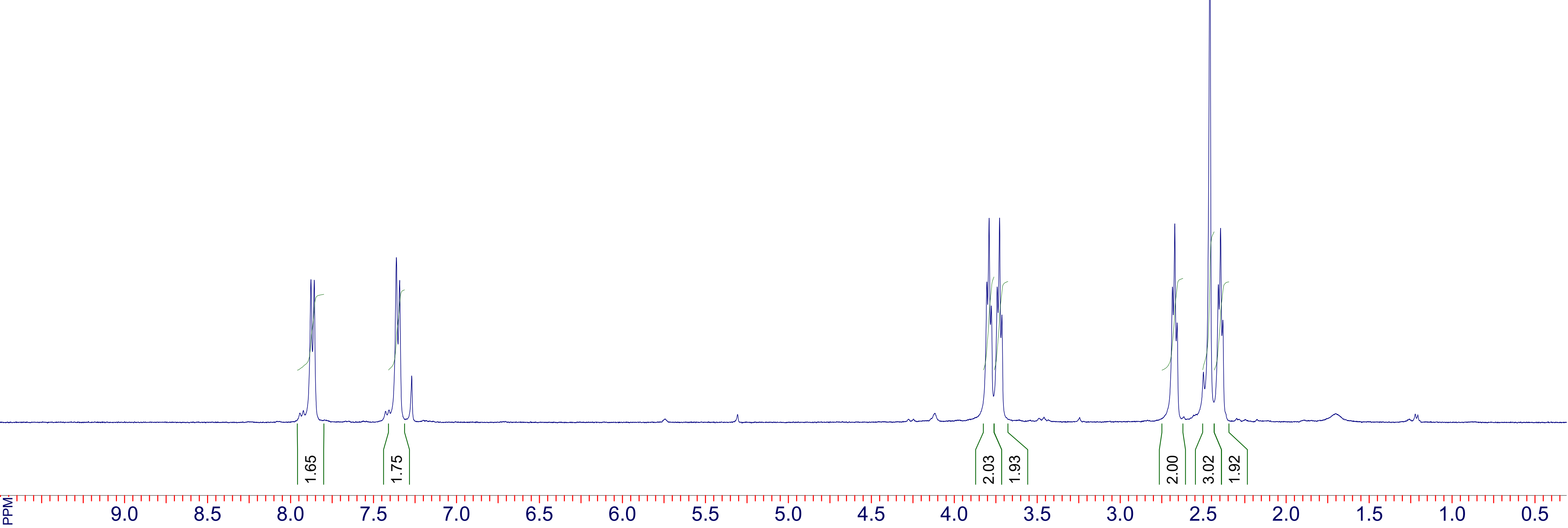

**File name: lv5**	Operator: nmr	SF: 400.1300 MHz	NSC: 1	PW: 0.00 usec, RG: 25	SI: 32768
Date: 19-Nov-2022	Solvent: CDCl3	SW: 8224 Hz	TE: 300 K	AQ: 1.99 sec, RD: 0.00 sec	

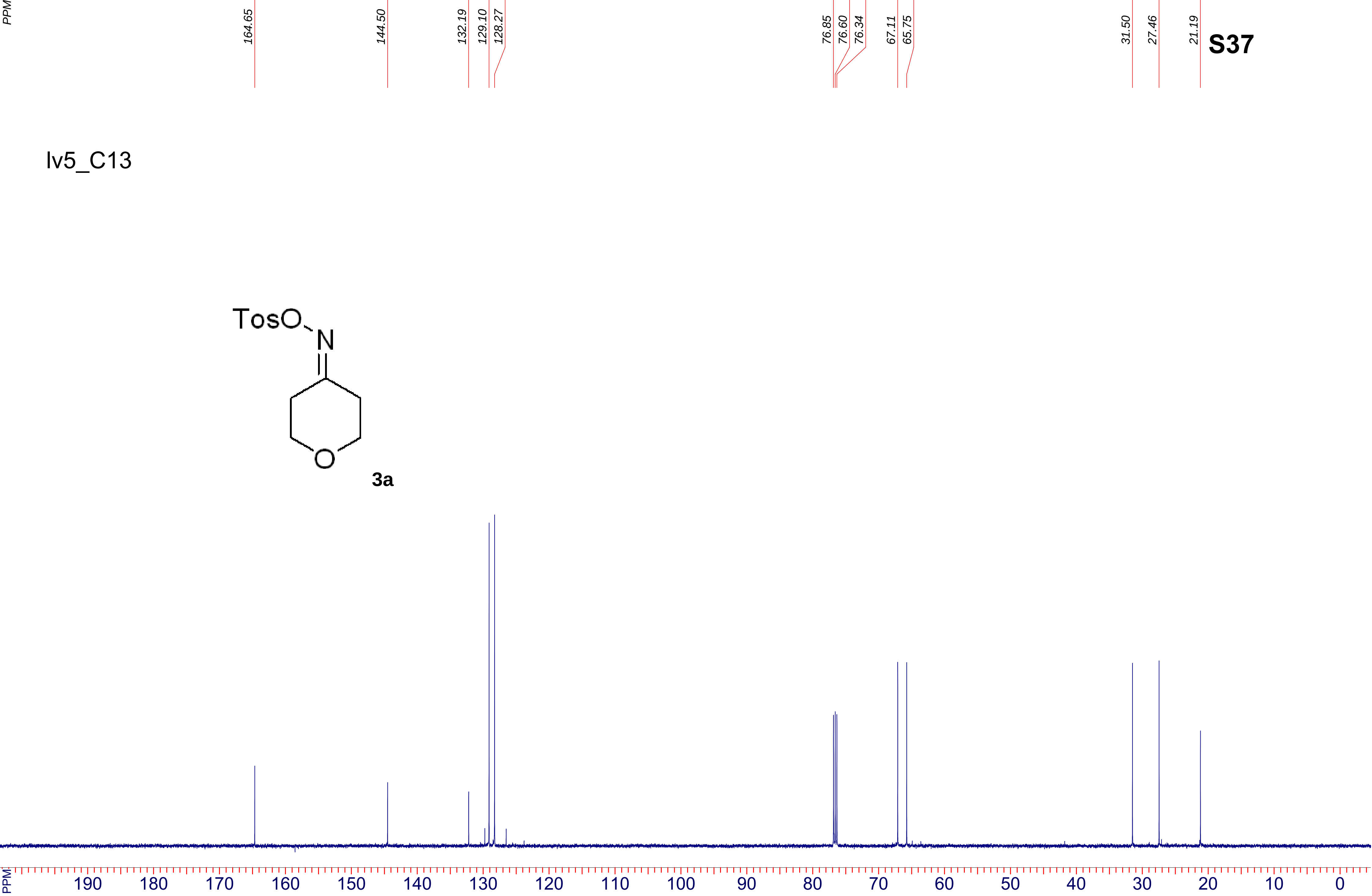

**File name: lv5_C13**	Operator: root	SF: 125.6429 MHz	NSC: 400	PW: 0.00 usec, RG: 51200	SI: 131072
Date: 23-Nov-2022	Solvent: CDCl3	SW: 32680 Hz	TE: 683 K	AQ: 0.78 sec, RD: 0.00 sec	

Target Compound Screening Report

**Data File**	4.d	**Sample Name**	5
**Sample Type**	Sample	**Position**	P1-A4
**Instrument Name**	Instrument 1	**User Name**	Denis V.Bylina
**Acq Method**	Fast_Gradient_HRMS_pos_Lock_08272019.m	**Acquired Time**	12/19/2022 1:08:20 PM (UTC+02:00)
**IRM Calibration Status**	Success	**DA Method**	1.m
**Comment**	Lysenko		
**Sample Group**		**Info.**	Agilent 6224 TOF LC/MS
**MFC**	C12H15NO4S	**Stream Name**	LC 1
**Acquisition Time (Local)**	12/19/2022 1:08:20 PM (UTC+02:00)	**Acquisition SW Version**	6200 series TOF/6500 series Q-TOF B.08.00 (B8058.0)
**TOF Driver Version**	8.00.00	**TOF Firmware Version**	8.643
**Tune Mass Range Max.**	1700		

Compound Table

Label	Tgt Score	Mass Error (ppm)	Tgt Formula	Obs. RT	Ref. Mass	Obs. Mass
Cpd 1: C12 H15 N O4 S; 2.957	98.18	-1.86	C12 H15 N O4 S	2.957	269.0722	269.0717

Obs. *m/z*	Obs. RT	Obs. Mass	Tgt Formula	Tgt Mass	Tgt Mass Error (ppm)	RT Diff.	Find Cpds Algorithm
270.079	2.957	269.0717	C12 H15 N O4 S	269.0722	-1.86	Find By Formula	

Compound Chromatograms

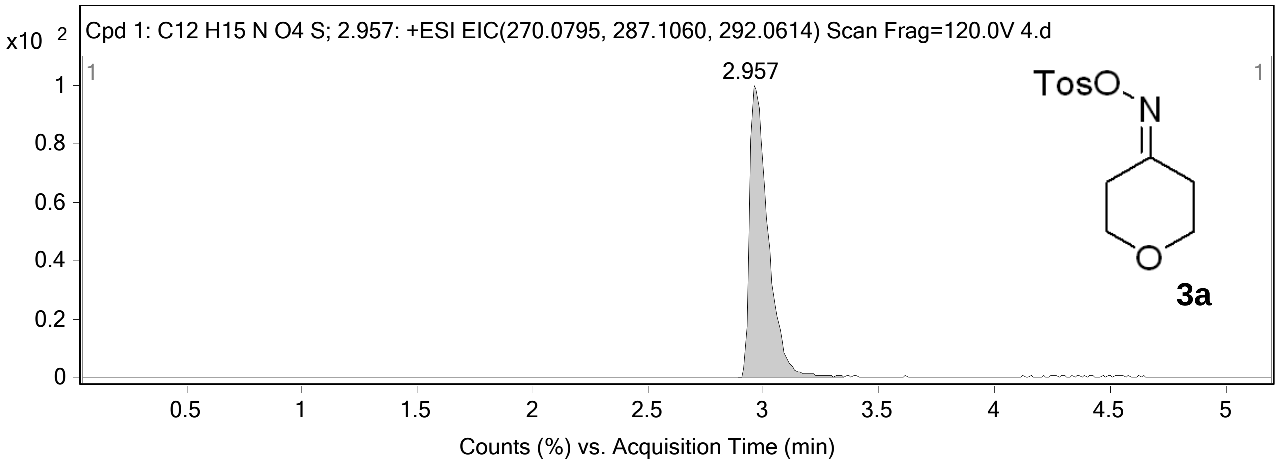

MS Zoomed Spectrum

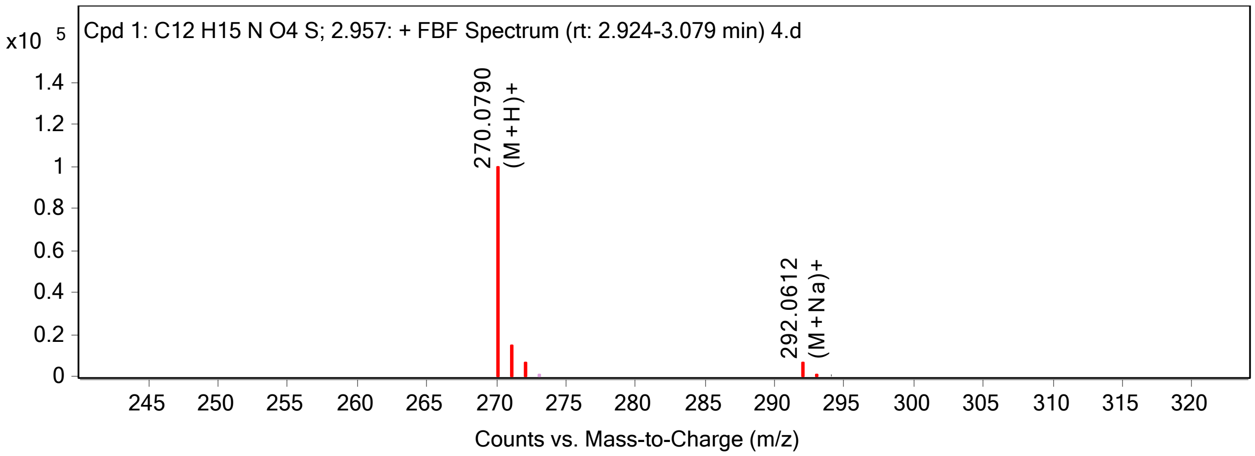

MS Spectrum Peak List

Obs. *m/z*	Charge	Abund	Ion/Isotope
270.079	1	99719.44	(M+H)+
271.0819	1	13317.24	(M+H)+
272.0772	1	5117.59	(M+H)+
292.0612	1	6215.6	(M+Na)+
293.0632	1	1095.24	(M+Na)+
294.0569	1	504.36	(M+Na)+

MS Zoomed Spectrum

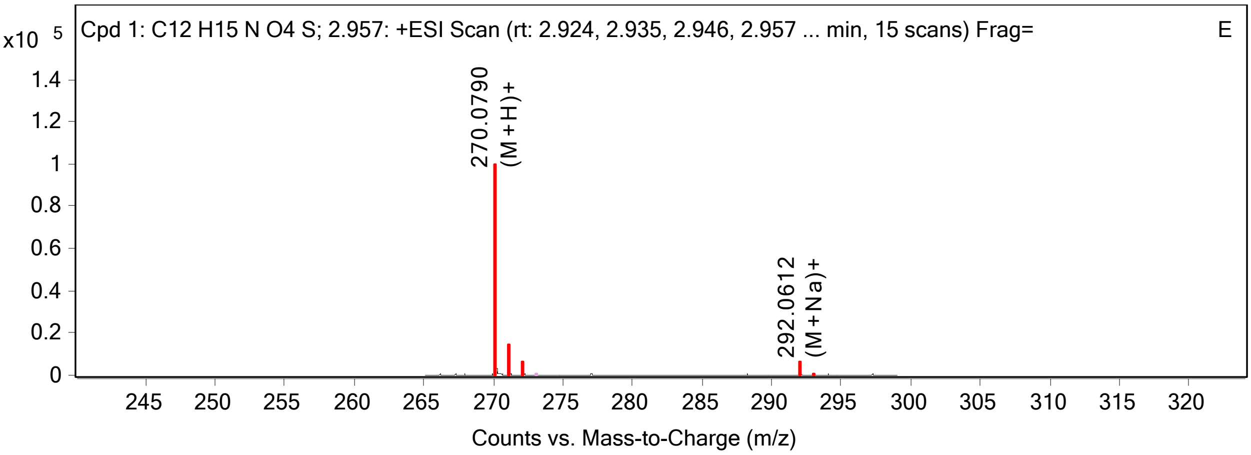

MS Spectrum Peak List

Obs. *m/z*	Charge	Abund	Ion/Isotope	Tgt Mass Error (ppm)
270.079	1	99719.44	(M+H)+	1.76
271.0819	1	13317.24	(M+H)+	2.14
272.0772	1	5117.59	(M+H)+	2.67
292.0612	1	6215.6	(M+Na)+	0.79
293.0632	1	1095.24	(M+Na)+	4.19
294.0569	1	504.36	(M+Na)+	10.05

--- End Of Report ---

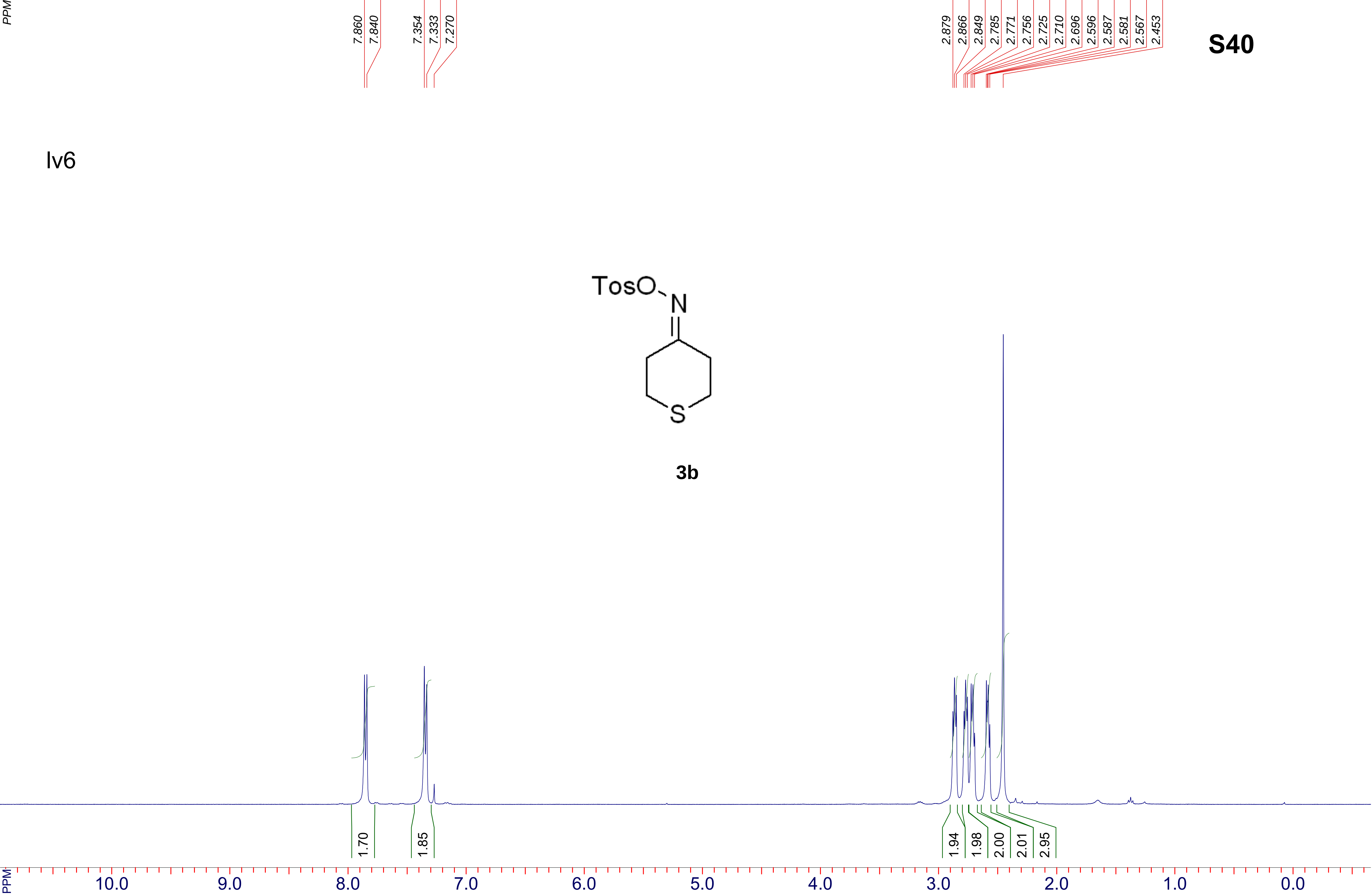

**File name: Iv6**	Operator: nmr	SF: 400.1300 MHz	NSC: 1	PW: 0.00 usec, RG: 25	SI: 32768
Date: 18-Nov-2022	Solvent: CDCl3	SW: 8224 Hz	TE: 300 K	AQ: 1.99 sec, RD: 0.00 sec	

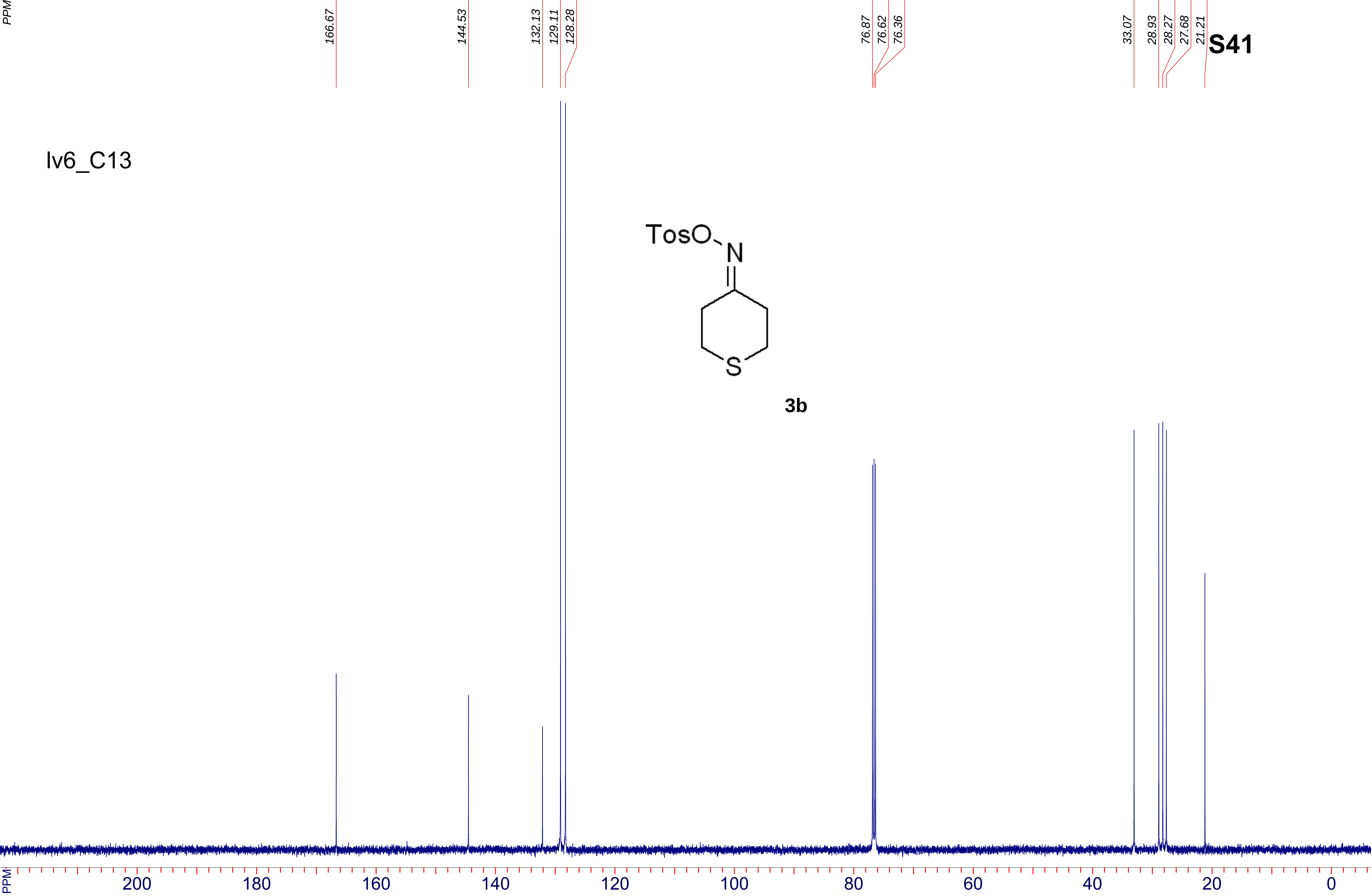

**File name: lv6_C13**	Operator: root	SF: 125.6429 MHz	NSC: 500	PW: 0.00 usec, RG: 51200	SI: 131072
Date: 19-Nov-2022	Solvent: CDCl3	SW: 32680 Hz	TE: 683 K	AQ: 0.78 sec, RD: 0.00 sec	

Target Compound Screening Report

**Data File**	5.d	**Sample Name**	6
**Sample Type**	Sample	**Position**	P1-A5
**Instrument Name**	Instrument 1	**User Name**	Denis V.Bylina
**Acq Method**	Fast_Gradient_HRMS_pos_Lock_08272019.m	**Acquired Time**	12/19/2022 1:20:22 PM (UTC+02:00)
**IRM Calibration Status**	Success	**DA Method**	1.m
**Comment**	Lysenko		
**Sample Group**		**Info.**	Agilent 6224 TOF LC/MS
**MFC**	C12H15NO3S2	**Stream Name**	LC 1
**Acquisition Time (Local)**	12/19/2022 1:20:22 PM (UTC+02:00)	**Acquisition SW Version**	6200 series TOF/6500 series Q-TOF B.08.00 (B8058.0)
**TOF Driver Version**	8.00.00	**TOF Firmware Version**	8.643
**Tune Mass Range Max.**	1700		

Compound Table

Label	Tgt Score	Mass Error (ppm)	Tgt Formula	Obs. RT	Ref. Mass	Obs. Mass
Cpd 1: C12 H15 N O3 S2; 3.212	94.24	-4.85	C12 H15 N O3 S2	3.212	285.0493	285.048

Obs. *m/z*	Obs. RT	Obs. Mass	Tgt Formula	Tgt Mass	Tgt Mass Error (ppm)	RT Diff.	Find Cpds Algorithm
286.0551	3.212	285.048	C12 H15 N O3 S2	285.0493	-4.85	Find By Formula	

Compound Chromatograms

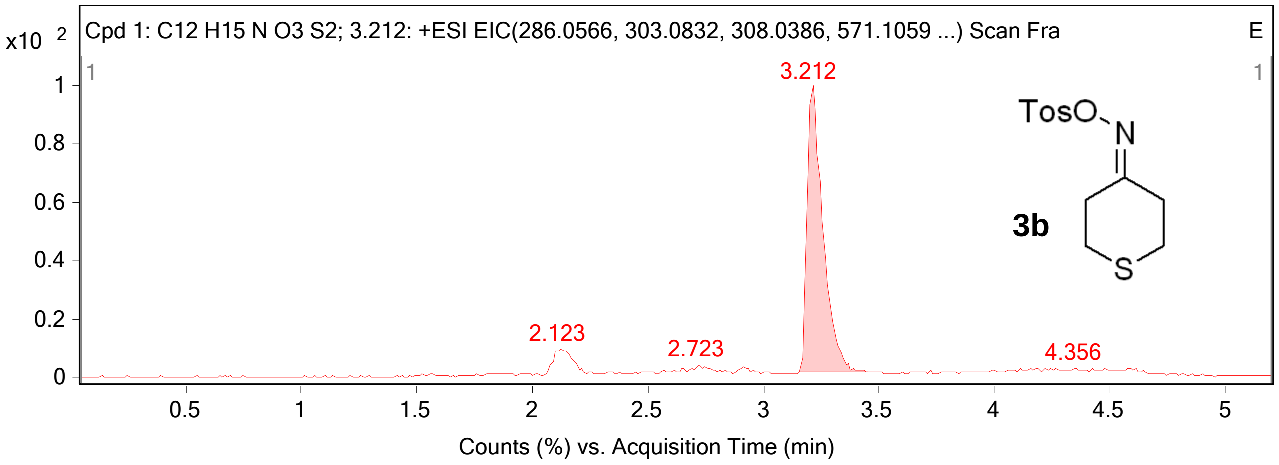

MS Zoomed Spectrum

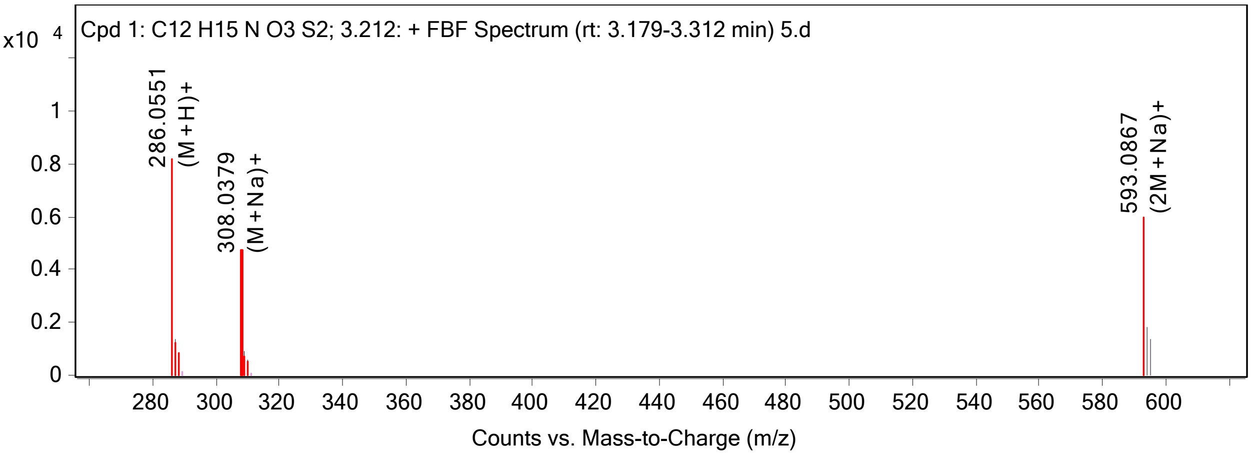

MS Spectrum Peak List

Obs. *m/z*	Charge	Abund	Ion/Isotope
286.0551	1	8088.92	(M+H)+
287.0583	1	1354.34	(M+H)+
288.0539	1	837.55	(M+H)+
308.0379	1	4477.54	(M+Na)+
309.0383	1	944.91	(M+Na)+
310.0302	1	580.76	(M+Na)+
593.0867	1	5946.17	(2M+Na)+
594.0888	1	1842.2	(2M+Na)+
595.0847	1	1377.22	(2M+Na)+

MS Zoomed Spectrum

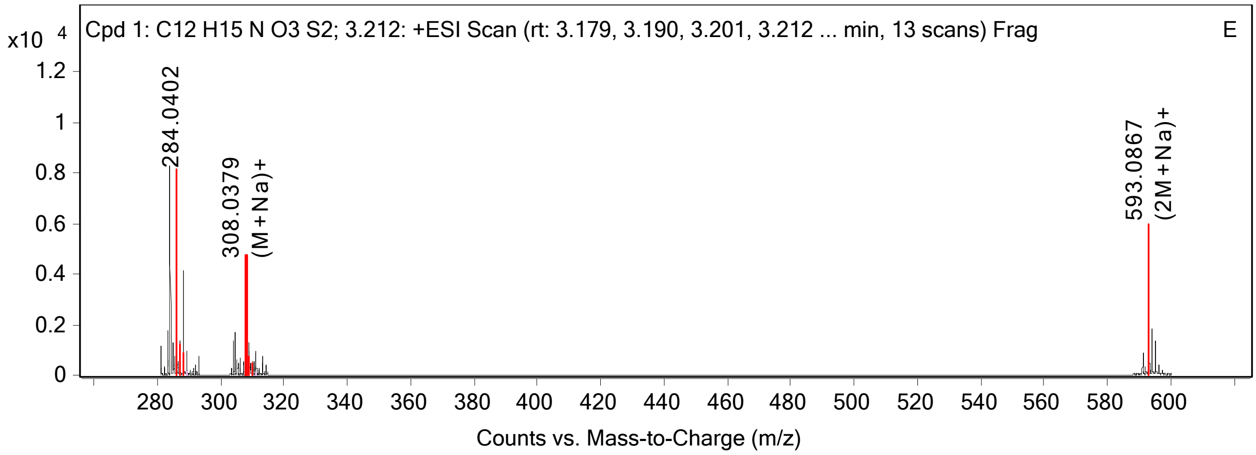

MS Spectrum Peak List

Obs. *m/z*	Charge	Abund	Ion/Isotope	Tgt Mass Error (ppm)
284.0402		8375.01		
286.0551	1	8088.92	(M+H)+	5.26
287.0583	1	1354.34	(M+H)+	3.85
288.0539	1	837.55	(M+H)+	-0.12
308.0379	1	4477.54	(M+Na)+	2.21
309.0383	1	944.91	(M+Na)+	9.94
310.0302	1	580.76	(M+Na)+	18.25
593.0867	1	5946.17	(2M+Na)+	2.01
594.0888	1	1842.2	(2M+Na)+	3.16
595.0847	1	1377.22	(2M+Na)+	2.12

--- End Of Report ---

lv590

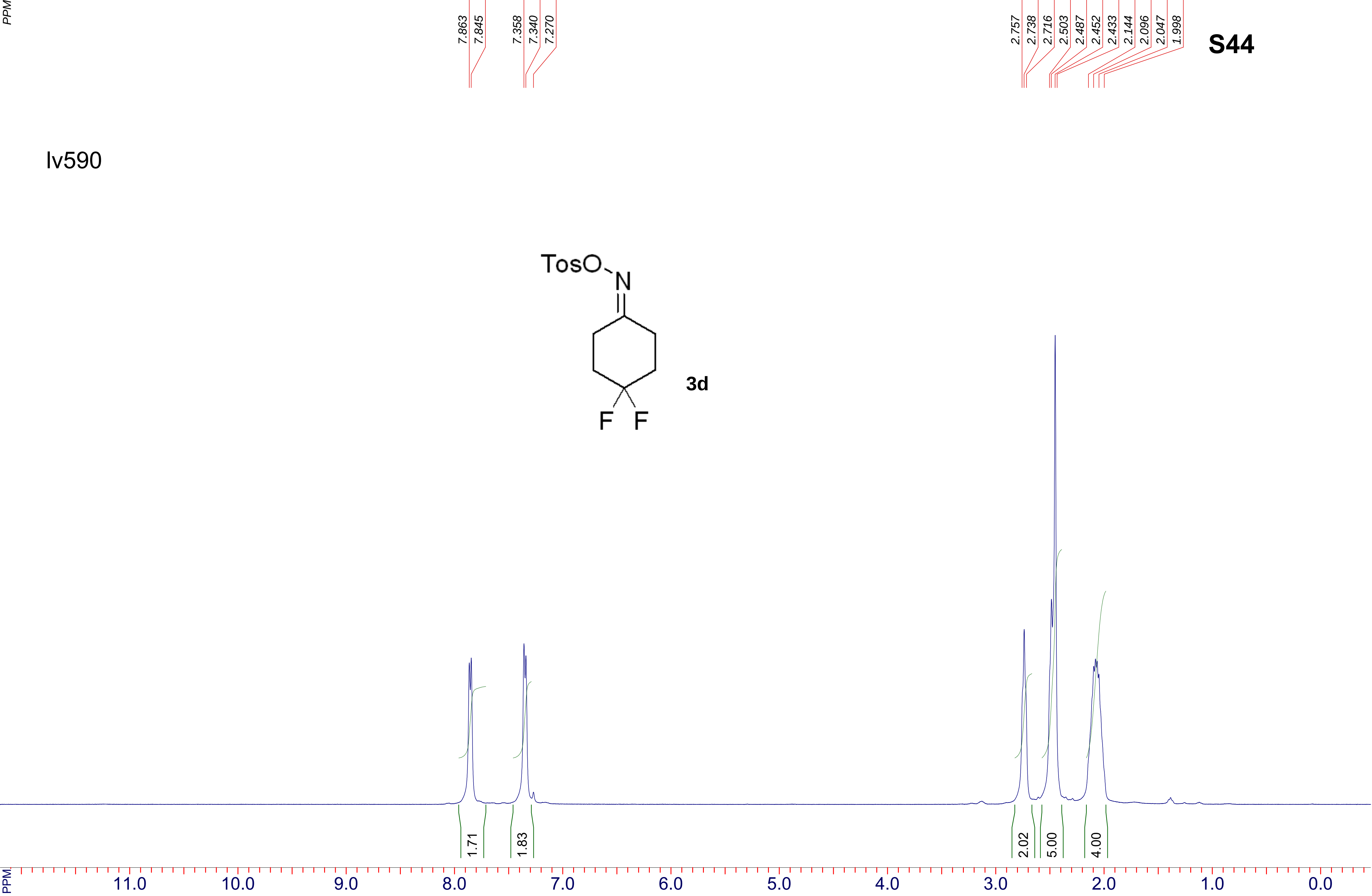

File name: lv590	Operator: nmr	SF: 400.1300 MHz	NSC: 1	PW: 0.00 usec, RG: 25	SI: 32768
Date: 31-Oct-2022	Solvent: CDCl3	SW: 8224 Hz	TE: 300 K	AQ: 1.99 sec, RD: 0.00 sec	

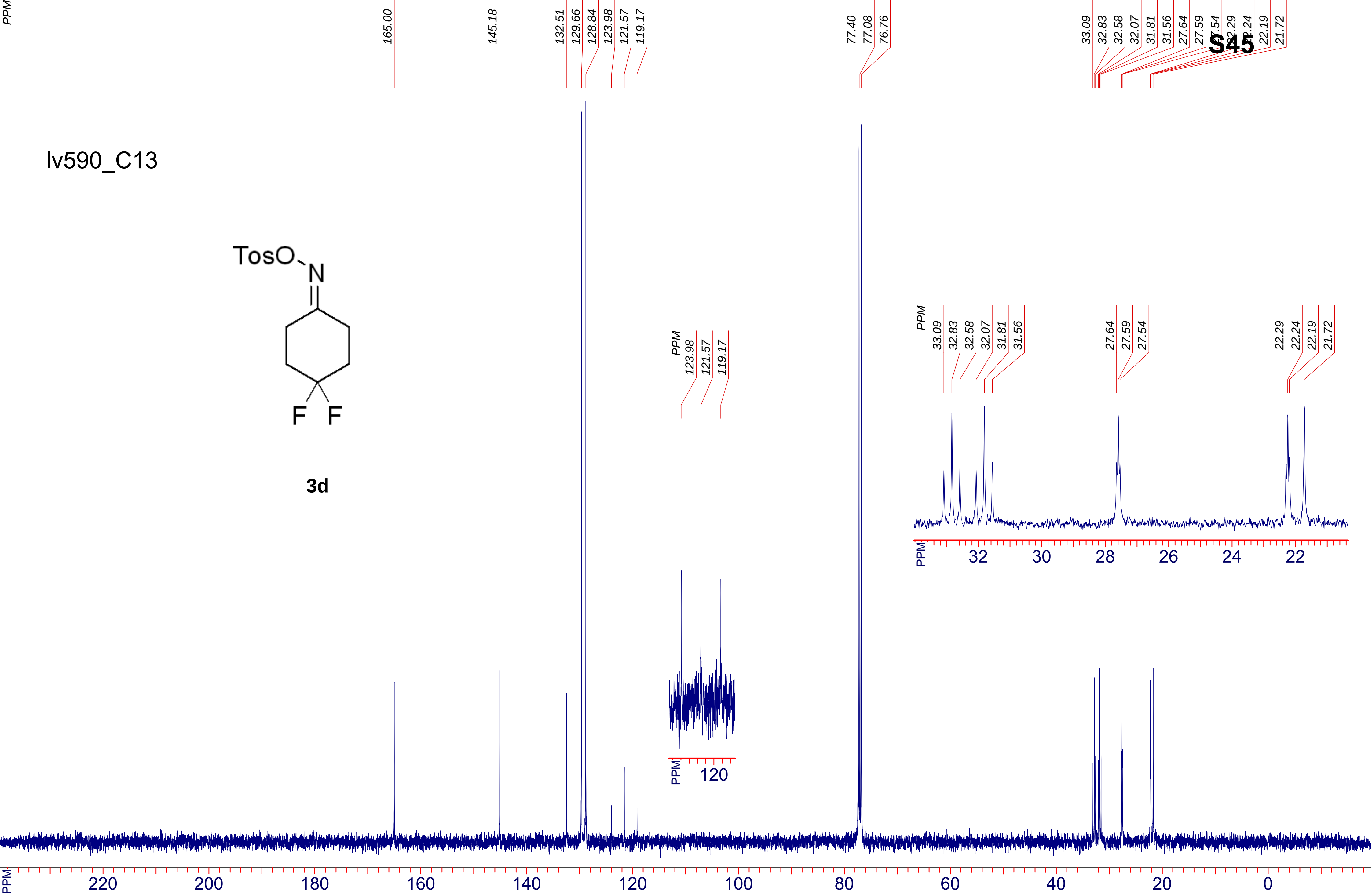

**File name: lv590_C13**	Operator: nmr	SF: 100.6128 MHz	NSC: 517	PW: 0.00 usec, RG: 2050	SI: 32768
Date: 31-Oct-2022	Solvent: CDCl3	SW: 26042 Hz	TE: 300 K	AQ: 1.26 sec, RD: 0.00 sec	

lv590_F19{H}

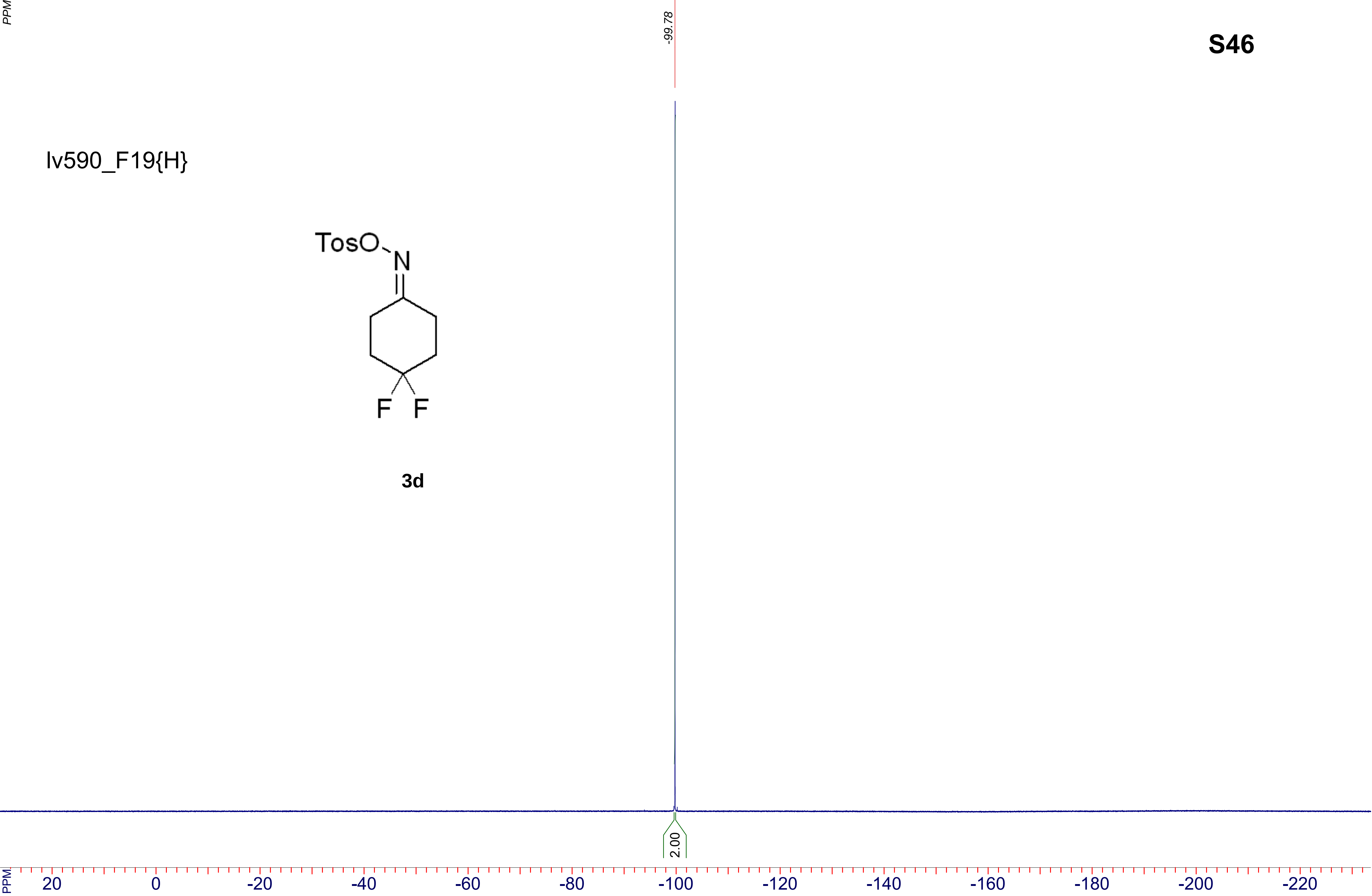

**File name: lv590_F19{H}**	Operator: nmr	SF: 376.4986 MHz	NSC: 1	PW: 0.00 usec, RG: 812	SI: 262144
Date: 31-Oct-2022	Solvent: CDCl3	SW: 138889 Hz	TE: 300 K	AQ: 0.94 sec, RD: 0.00 sec	19F-{1H}

Target Compound Screening Report

**Data File**	6.d	**Sample Name**	8
**Sample Type**	Sample	**Position**	P1-A6
**Instrument Name**	Instrument 1	**User Name**	Denis V.Bylina
**Acq Method**	Fast_Gradient_HRMS_pos_Lock_08272019.m	**Acquired Time**	12/19/2022 1:26:18 PM (UTC+02:00)
**IRM Calibration Status**	Success	**DA Method**	1.m
**Comment**	Lysenko		
**Sample Group**		**Info.**	Agilent 6224 TOF LC/MS
**MFC**	C13H15F2NO3S	**Stream Name**	LC 1
**Acquisition Time (Local)**	12/19/2022 1:26:18 PM (UTC+02:00)	**Acquisition SW Version**	6200 series TOF/6500 series Q-TOF B.08.00 (B8058.0)
**TOF Driver Version**	8.00.00	**TOF Firmware Version**	8.643
**Tune Mass Range Max.**	1700		

Compound Table

Label	Tgt Score	Mass Error (ppm)	Tgt Formula	Obs. RT	Ref. Mass	Obs. Mass
Cpd 1: C13 H15 F2 N O3 S; 3.230	98.67	-1.67	C13 H15 F2 N O3 S	3.23	303.0741	303.0736

Obs. *m/z*	Obs. RT	Obs. Mass	Tgt Formula	Tgt Mass	Tgt Mass Error (ppm)	RT Diff.	Find Cpds Algorithm
304.0807	3.23	303.0736	C13 H15 F2 N O3 S	303.0741	-1.67	Find By Formula	

Compound Chromatograms

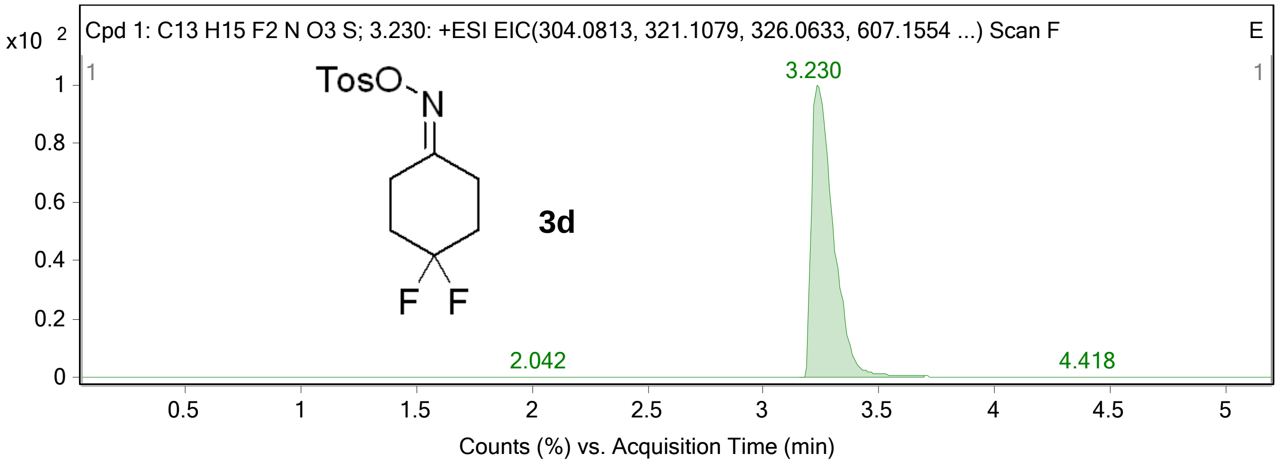

3d

MS Zoomed Spectrum

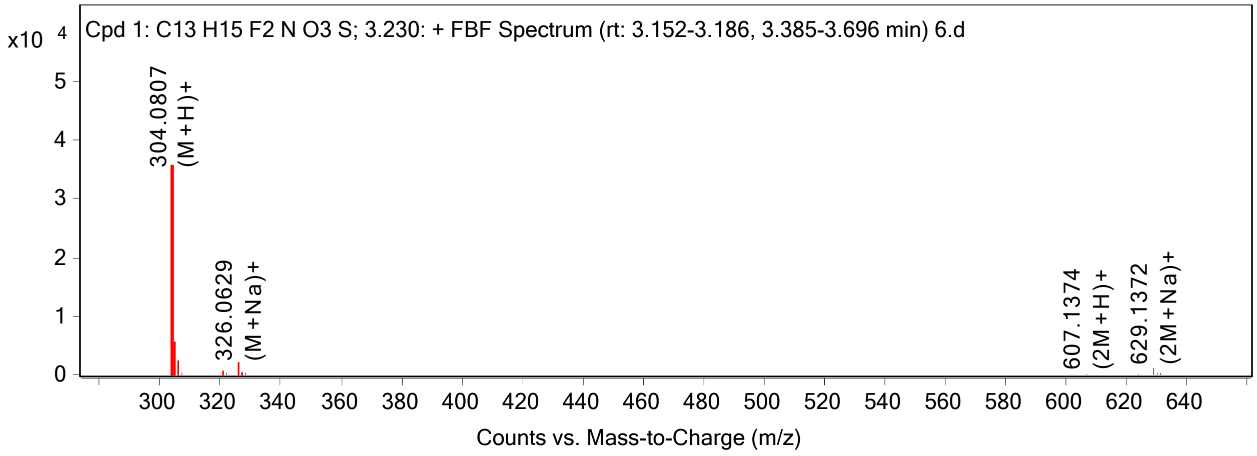

Agilent Technologies

MS Spectrum Peak List

Obs. *m/z*	Charge	Abund	Ion/Isotope
304.0807	1	35639.16	(M+H)+
305.0842	1	5191.17	(M+H)+
306.0793	1	2023.62	(M+H)+
321.1106	1	433.45	(M+NH4)+
326.0629	1	1653	(M+Na)+
327.0692	1	461.21	(M+Na)+
328.0608	1	233.34	(M+Na)+
629.1372	1	1140.27	(2M+Na)+
630.1465	1	389.5	(2M+Na)+
631.1379	1	212.14	(2M+Na)+

MS Zoomed Spectrum

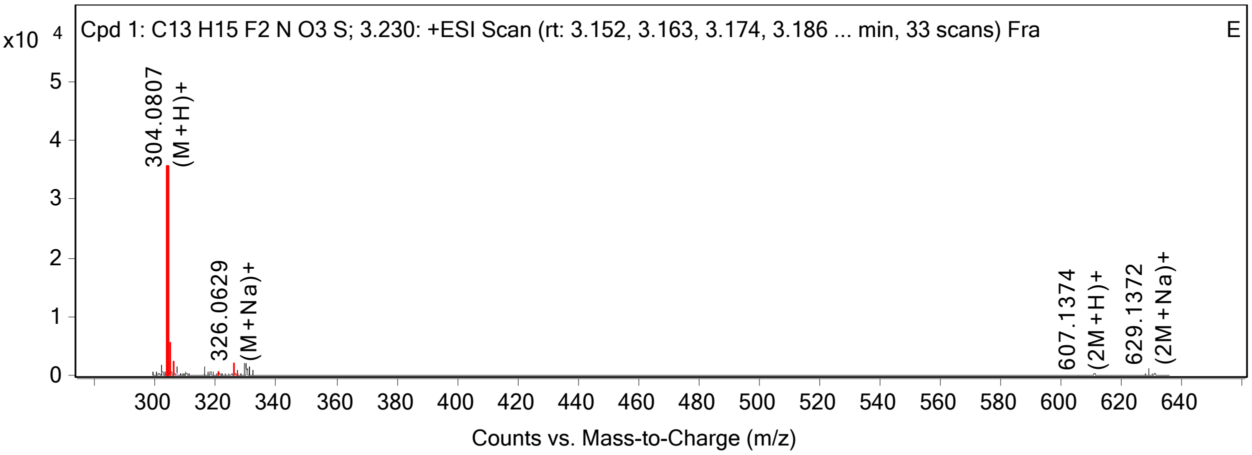

MS Spectrum Peak List

Obs. *m/z*	Charge	Abund	Ion/Isotope	Tgt Mass Error (ppm)
304.0807	1	35639.16	(M+H)+	2.28
305.0842	1	5191.17	(M+H)+	0.76
306.0793	1	2023.62	(M+H)+	1.63
321.1106	1	433.45	(M+NH4)+	-8.37
326.0629	1	1653	(M+Na)+	1.06
327.0692	1	461.21	(M+Na)+	-8.81
328.0608	1	233.34	(M+Na)+	2.86
629.1372	1	1140.27	(2M+Na)+	0.19
630.1465	1	389.5	(2M+Na)+	-9.69
631.1379	1	212.14	(2M+Na)+	-1.33

--- End Of Report ---

PPM 8.665 4.448 4.430 4.405 4.255 4.228 4.208 4.175 4.149 4.133 3.606 3.575 3.544 3.518 2.914 2.883 2.860 2.829 2.499 2.424 2.388

R730837.fid

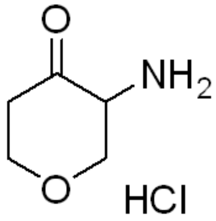

4a

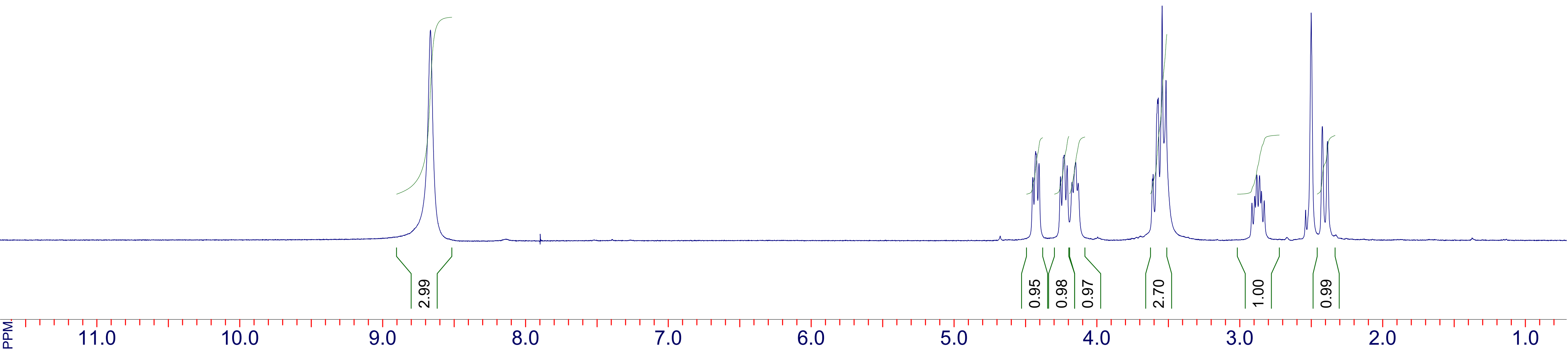

File name: R730837.fid	Operator:	SF: 399.9831 MHz	NSC: 0	PW: 7.50 usec, RG: 34	SI: 16384
Date: 14-Sep-2016	Solvent: dmso_d6	SW: 7639 Hz	TE: 293 K	AQ: 1.05 sec, RD: 0.00 sec	

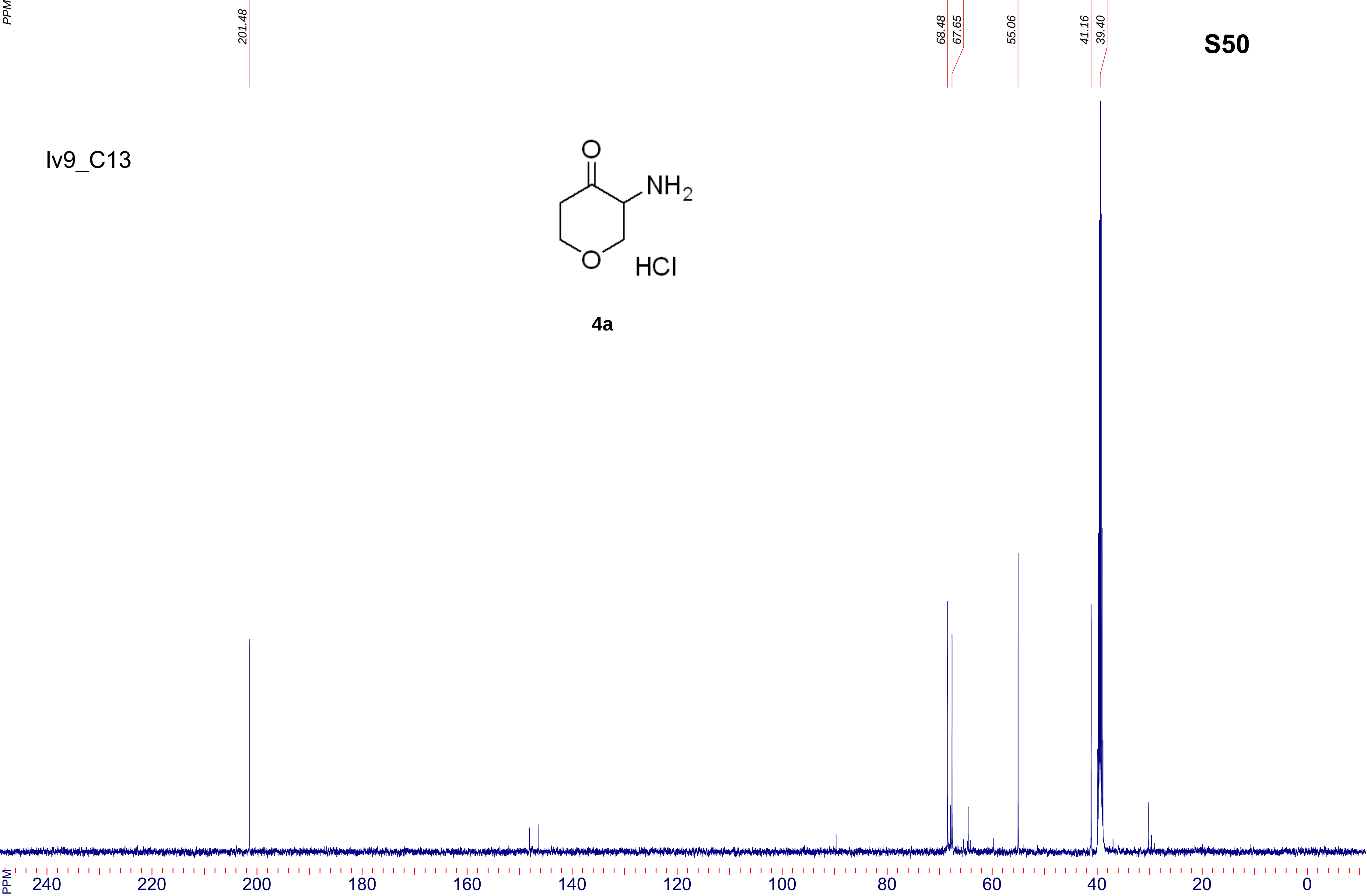

File name: lv9_C13	Operator: root	SF: 125.6429 MHz	NSC: 350	PW: 0.00 usec, RG: 51200	SI: 131072
Date: 11-Mar-2023	Solvent: DMSO	SW: 32680 Hz	TE: 683 K	AQ: 1.00 sec, RD: 0.00 sec	

Target Compound Screening Report

**Data File**	18d.d	**Sample Name**	9
**Sample Type**	Sample	**Position**	P1-B9
**Instrument Name**	Instrument 1	**User Name**	Denis V.Bylina
**Acq Method**	Fast_Gradient_HRMS_pos_Lock_01312023.m	**Acquired Time**	7/10/2023 12:45:27 PM (UTC+03:00)
**IRM Calibration Status**	Success	**DA Method**	1.m
**Comment**	Lysenko		
**Sample Group**		**Info.**	Agilent 6224 TOF LC/MS
**MFC**	C5H9NO2	**Stream Name**	LC 1
**Acquisition Time (Local)**	7/10/2023 12:45:27 PM (UTC+03:00)	**Acquisition SW Version**	6200 series TOF/6500 series Q-TOF B.08.00 (B8058.0)
**TOF Driver Version**	8.00.00	**TOF Firmware Version**	8.643
**Tune Mass Range Max.**	1700		

Compound Table

Label	Tgt Score	Mass Error (ppm)	Tgt Formula	Obs. RT	Ref. Mass	Obs. Mass
Cpd 2: C5 H9 N O2; 0.416	95.89	4.52	C5 H9 N O2	0.416	115.06333	115.06385

Obs. *m/z*	Obs. RT	Obs. Mass	Tgt Formula	Tgt Mass	Tgt Mass Error (ppm)	RT Diff.	Find Cpds Algorithm
116.07102	0.416	115.06385	C5 H9 N O2	115.06333	4.52	Find By Formula	

Compound Chromatograms

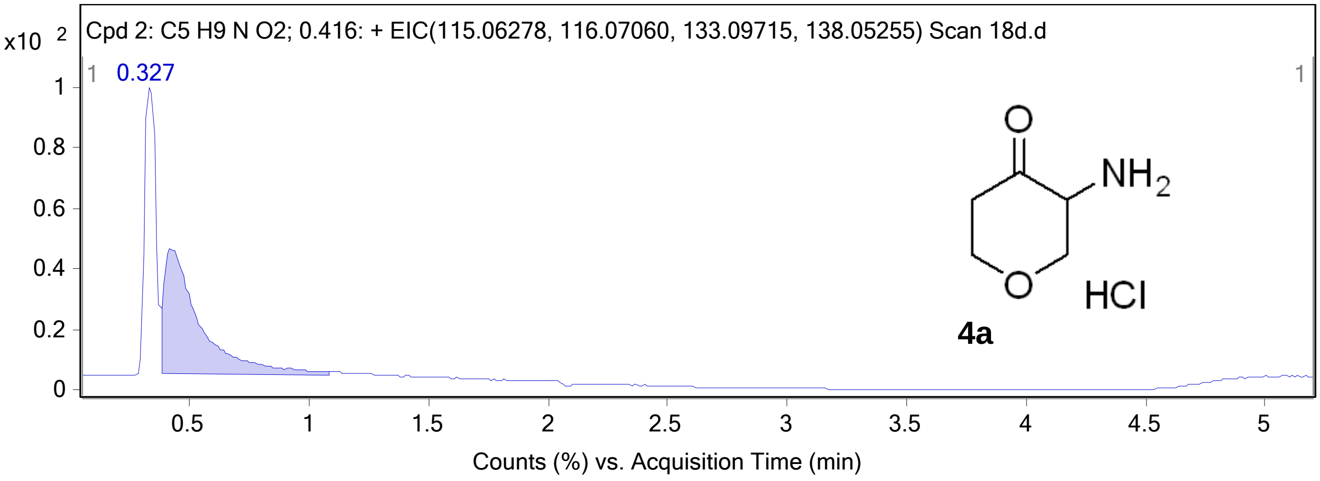

MS Zoomed Spectrum

Target Compound Screening Report

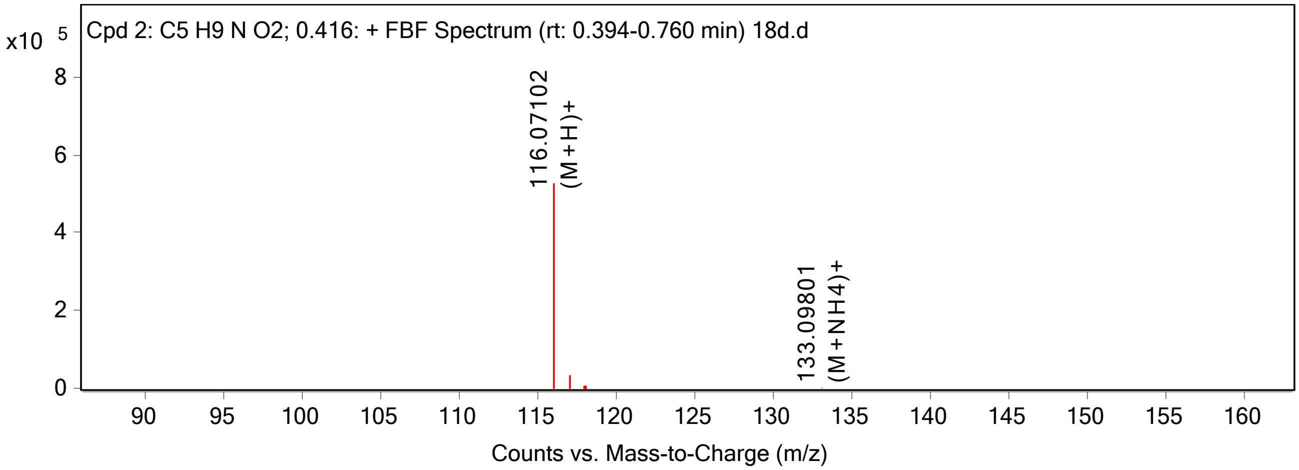

MS Spectrum Peak List

Obs. *m/z*	Charge	Abund	Ion/Isotope
116.07102	1	526392.69	(M+H)+
117.07397	1	27148.16	(M+H)+
118.08453	1	7066.56	(M+H)+
133.09801	1	1934.45	(M+NH4)+

MS Zoomed Spectrum

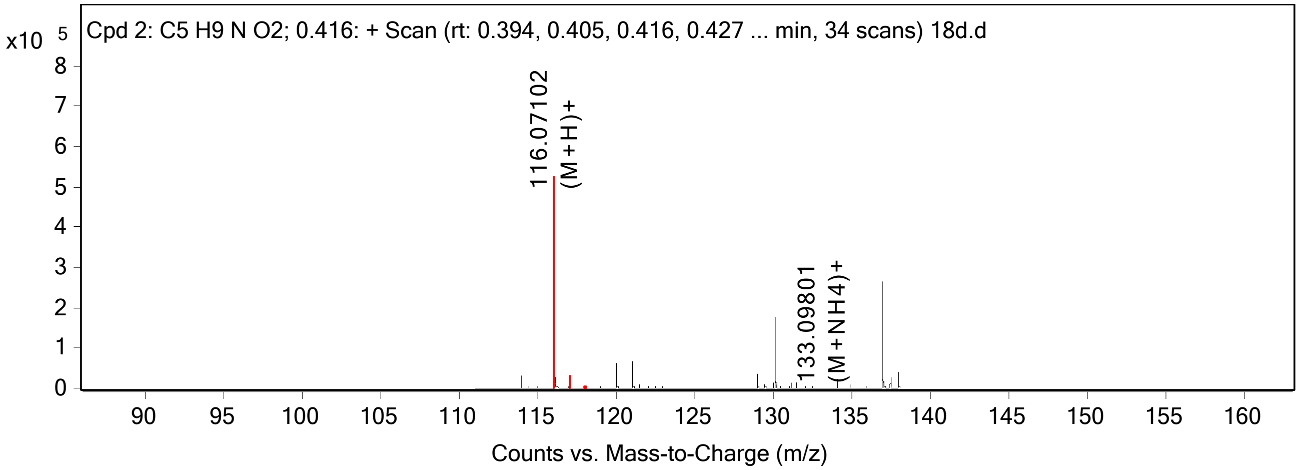

MS Spectrum Peak List

Obs. *m/z*	Charge	Abund	Ion/Isotope	Tgt Mass Error (ppm)
116.07102	1	526392.66	(M+H)+	-3.55
117.07397	1	27148.16	(M+H)+	-2.81
118.08453	1	7066.56	(M+H)+	-78.05
133.09801	1	1934.45	(M+NH4)+	-6.46

--- End Of Report ---

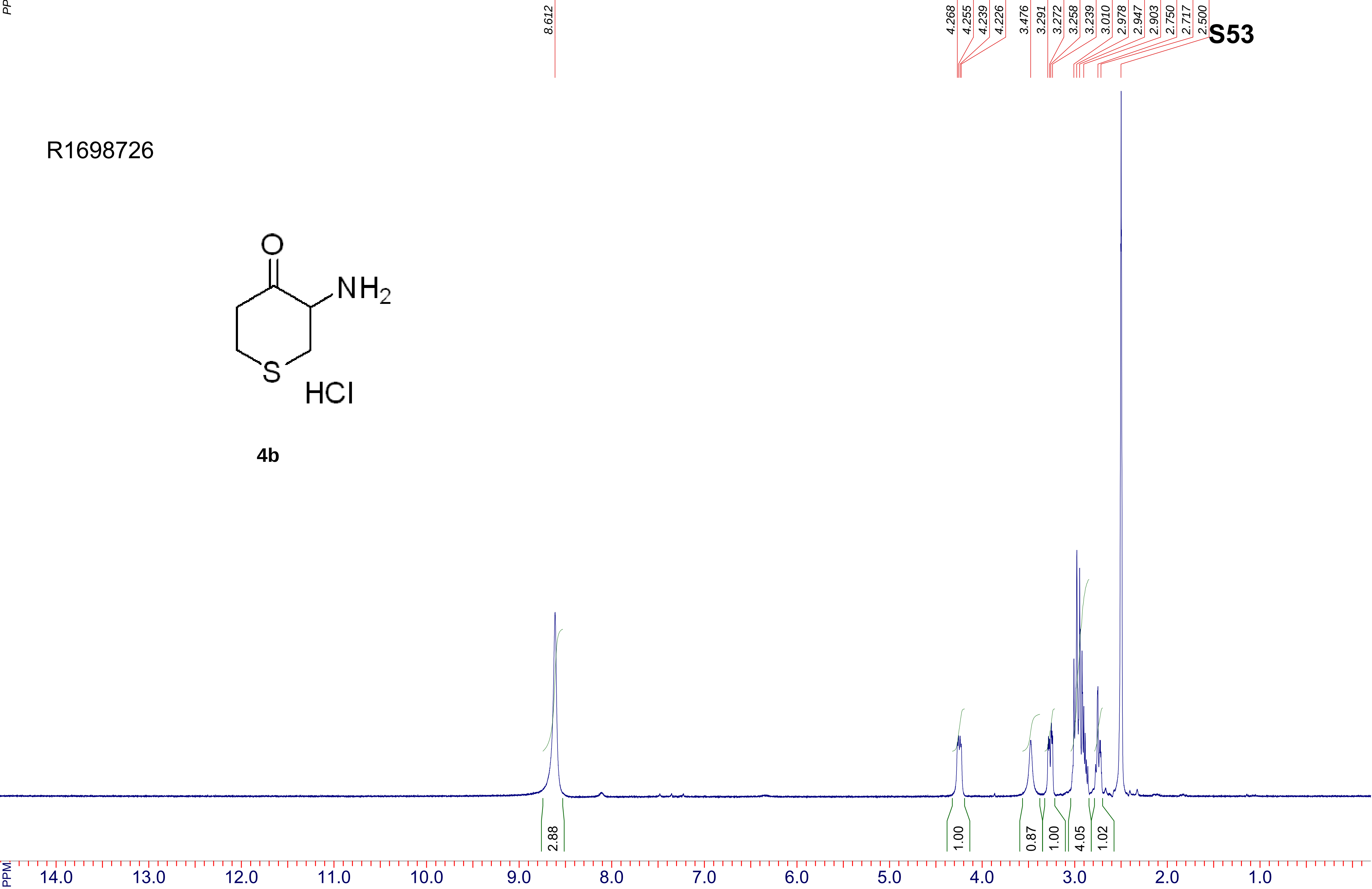

R1698726

4b

**File name: R1698726**	Operator:	SF: 399.9800 MHz	NSC: 0	PW: 11.30 usec, RG: 40	SI: 32768
Date: 16-Mar-2020	Solvent: dmso	SW: 7599 Hz	TE: 294 K	AQ: 2.16 sec, RD: 0.00 sec	

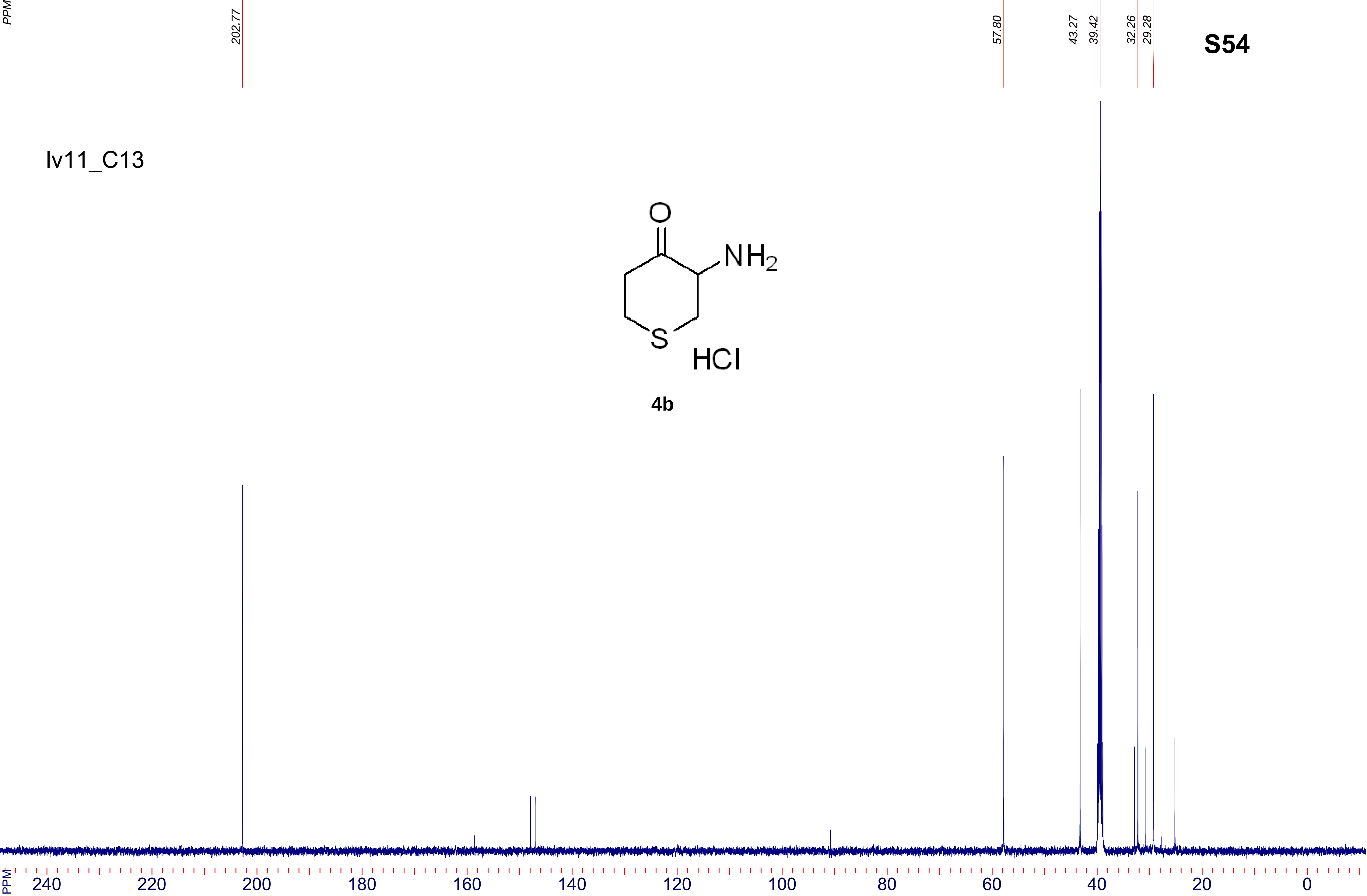

**File name: lv11_C13**	Operator: root	SF: 125.6429 MHz	NSC: 612	PW: 0.00 usec, RG: 51200	SI: 131072
Date: 12-Mar-2023	Solvent: DMSO	SW: 32680 Hz	TE: 683 K	AQ: 0.78 sec, RD: 0.00 sec	

Target Compound Screening Report

**Data File**	17.d	**Sample Name**	R1698726
**Sample Type**	Sample	**Position**	P1-F1
**Instrument Name**	Instrument 1	**User Name**	Denis V.Bylina
**Acq Method**	Fast_Gradient_HRMS_pos_Lock_01312023.m	**Acquired Time**	6/5/2023 5:27:53 PM (UTC+03:00)
**IRM Calibration Status**	Success	**DA Method**	1.m
**Comment**	H000108		
**Sample Group**		**Info.**	Agilent 6224 TOF LC/MS
**MFC**	C5H9NOS	**Stream Name**	LC 1
**Acquisition Time (Local)**	6/5/2023 5:27:53 PM (UTC+03:00)	**Acquisition SW Version**	6200 series TOF/6500 series Q-TOF B.08.00 (B8058.0)
**TOF Driver Version**	8.00.00	**TOF Firmware Version**	8.643
**Tune Mass Range Max.**	1700		

Compound Table

Label	Tgt Score	Mass Error (ppm)	Tgt Formula	Obs. RT	Ref. Mass	Obs. Mass
Cpd 1: C5 H9 N O S; 0.355	98.91	0.35	C5 H9 N O S	0.355	131.04048	131.04053

Obs. *m/z*	Obs. RT	Obs. Mass	Tgt Formula	Tgt Mass	Tgt Mass Error (ppm)	RT Diff.	Find Cpds Algorithm
132.04787	0.355	131.04053	C5 H9 N O S	131.04048	0.35	Find By Formula	

Compound Chromatograms

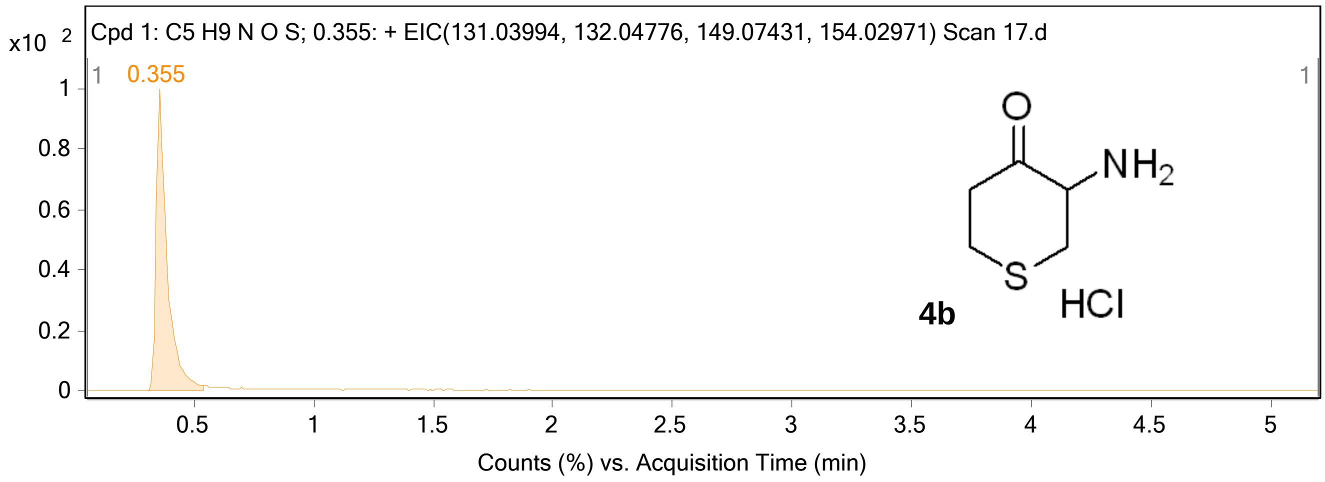

MS Zoomed Spectrum

Target Compound Screening Report

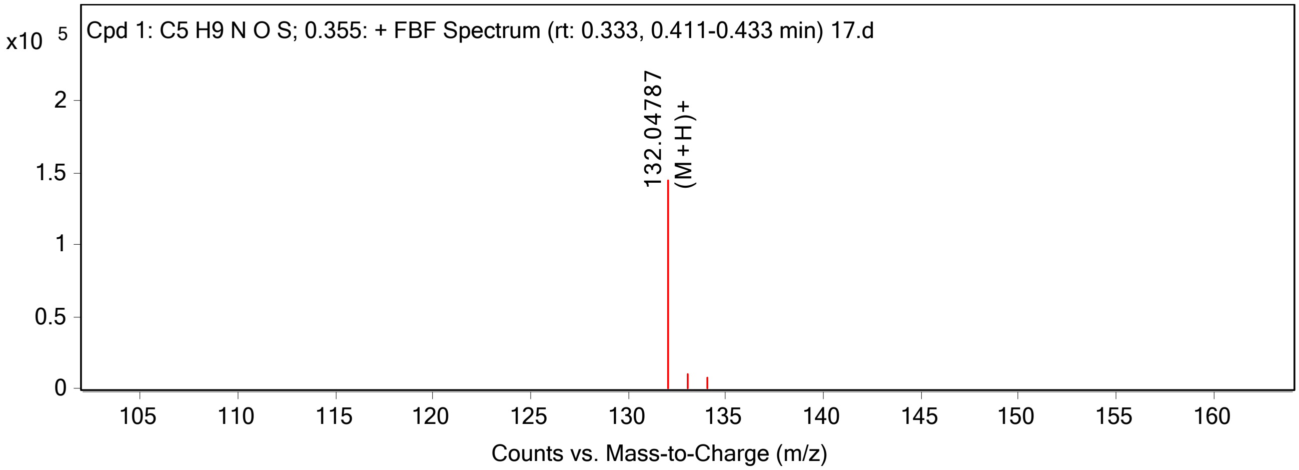

MS Spectrum Peak List

Obs. *m/z*	Charge	Abund	Ion/Isotope
132.04787	1	144863.63	(M+H)+
133.04952	1	9625.54	(M+H)+
134.0443	1	6756.2	(M+H)+

MS Zoomed Spectrum

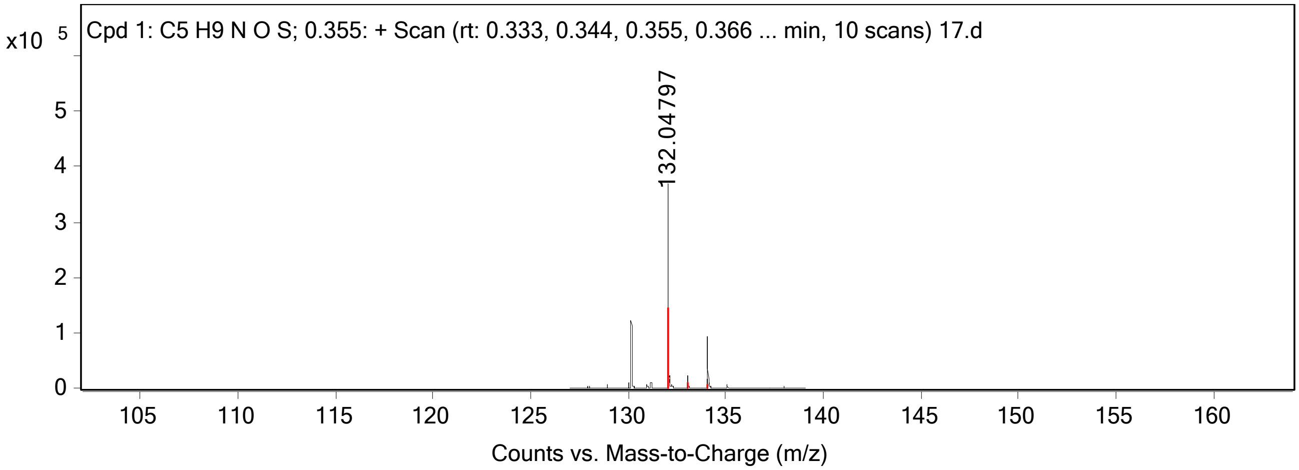

MS Spectrum Peak List

Obs. *m/z*	Charge	Abund	Ion/Isotope	Tgt Mass Error (ppm)
132.04787	1	144863.63	(M+H)+	-0.81
132.04797		373150.78		
133.04952	1	9625.54	(M+H)+	6.32
134.0443	1	6756.2	(M+H)+	-0.1

--- End Of Report ---

NCBZ aminoketone 1H

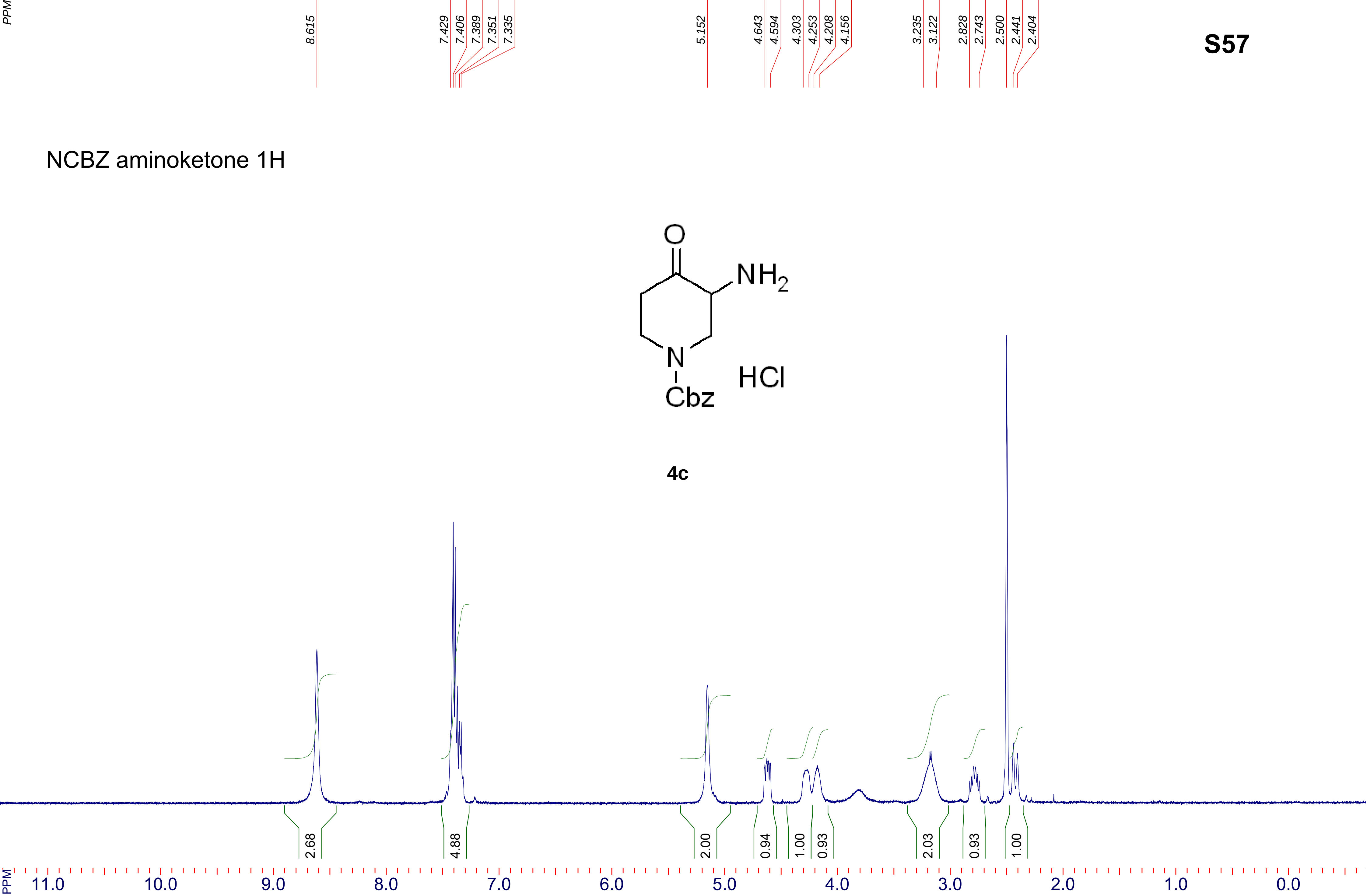

4c

File name: NCBZ aminoketone 1H	Operator:	SF: 399.9800 MHz	NSC: 0	PW: 11.30 usec, RG: 30	SI: 32768
Date: 03-Mar-2020	Solvent: dmso	SW: 7599 Hz	TE: 294 K	AQ: 2.16 sec, RD: 0.00 sec	

lv12_C13

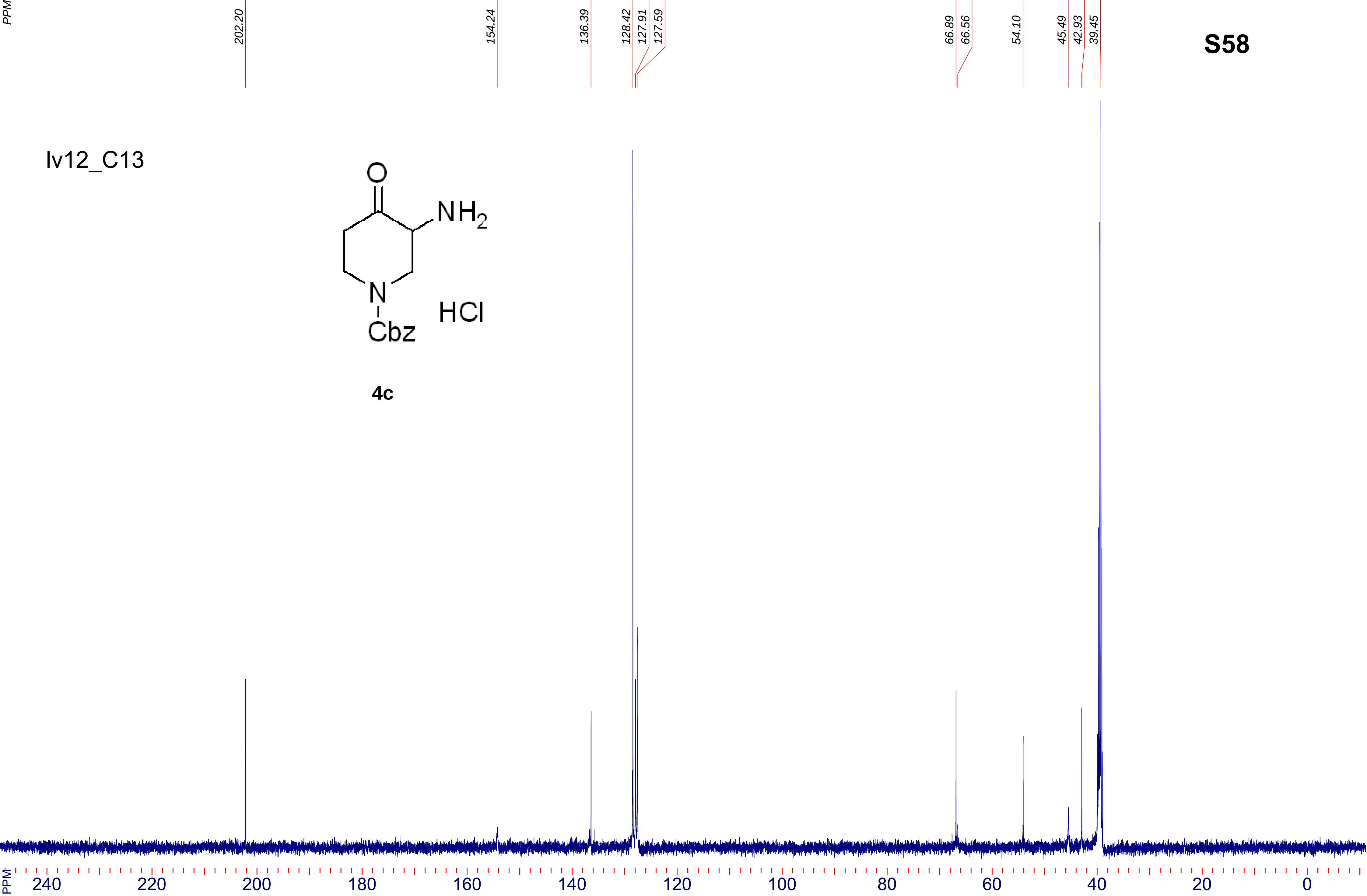

4c

**File name: lv12_C13**	Operator: root	SF: 125.6429 MHz	NSC: 270	PW: 0.00 usec, RG: 51200	SI: 131072
Date: 12-Mar-2023	Solvent: DMSO	SW: 32680 Hz	TE: 683 K	AQ: 0.78 sec, RD: 0.00 sec	

Target Compound Screening Report

**Data File**	19.d	**Sample Name**	R1686331
**Sample Type**	Sample	**Position**	P1-F3
**Instrument Name**	Instrument 1	**User Name**	Denis V.Bylina
**Acq Method**	Fast_Gradient_HRMS_pos_Lock_01312023.m	**Acquired Time**	6/5/2023 5:39:56 PM (UTC+03:00)
**IRM Calibration Status**	Success	**DA Method**	1.m
**Comment**	H000108		
**Sample Group**		**Info.**	Agilent 6224 TOF LC/MS
**MFC**	C13H16N2O3	**Stream Name**	LC 1
**Acquisition Time (Local)**	6/5/2023 5:39:56 PM (UTC+03:00)	**Acquisition SW Version**	6200 series TOF/6500 series Q-TOF B.08.00 (B8058.0)
**TOF Driver Version**	8.00.00	**TOF Firmware Version**	8.643
**Tune Mass Range Max.**	1700		

Compound Table

Label	Tgt Score	Mass Error (ppm)	Tgt Formula	Obs. RT	Ref. Mass	Obs. Mass
Cpd 1: C13 H16 N2 O3; 1.396	98.22	1.21	C13 H16 N2 O3	1.396	248.11609	248.11639

Obs. *m/z*	Obs. RT	Obs. Mass	Tgt Formula	Tgt Mass	Tgt Mass Error (ppm)	RT Diff.	Find Cpds Algorithm
249.12377	1.396	248.11639	C13 H16 N2 O3	248.11609	1.21	Find By Formula	

Compound Chromatograms

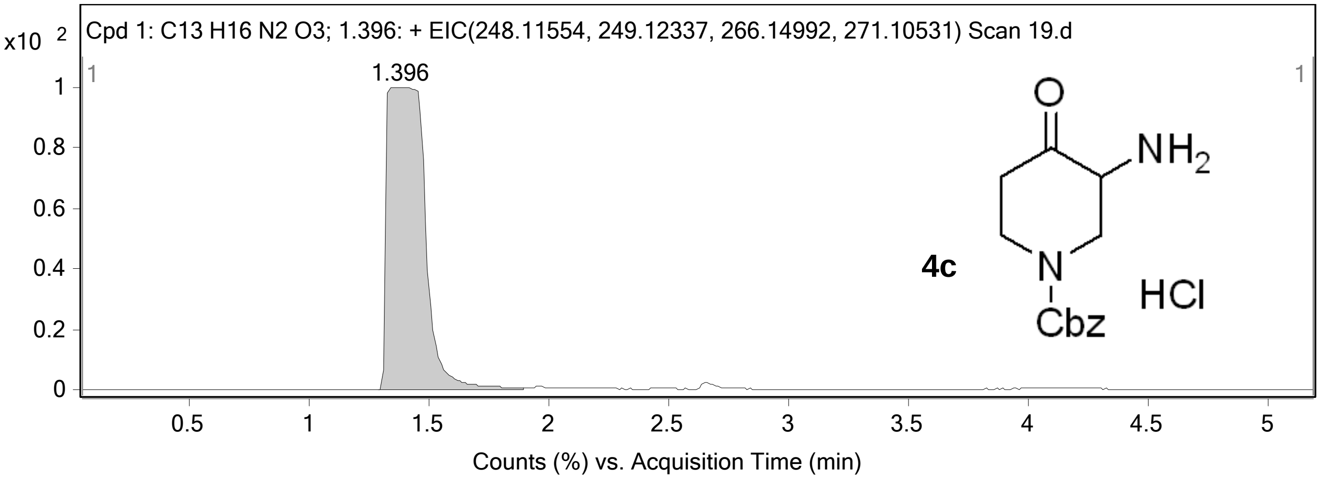

MS Zoomed Spectrum

Target Compound Screening Report

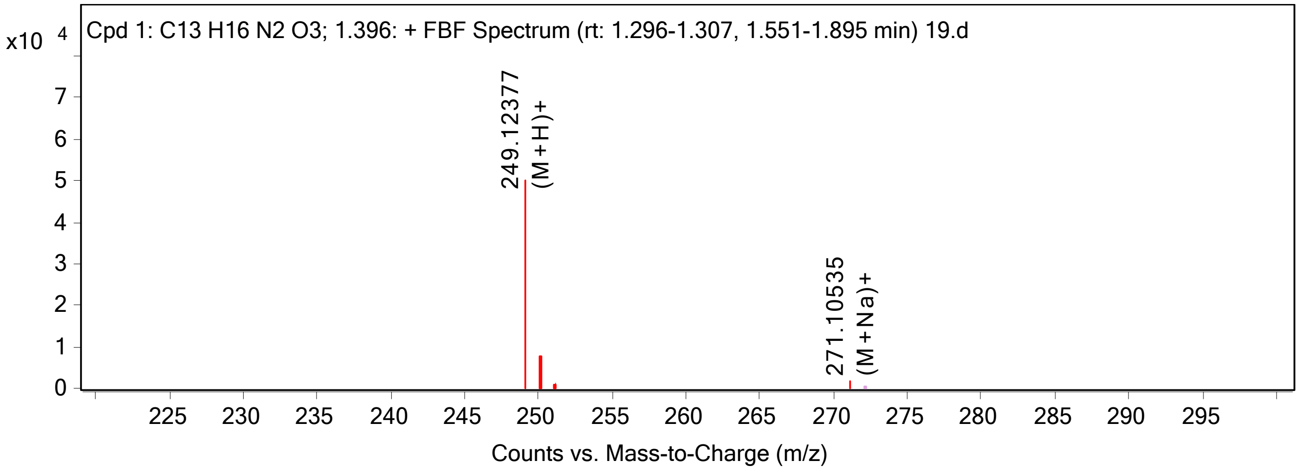

MS Spectrum Peak List

Obs. *m/z*	Charge	Abund	Ion/Isotope
249.12377	1	49834.08	(M+H)+
250.12647	1	7164.95	(M+H)+
251.12737	1	1470.44	(M+H)+
271.10535	1	1873.94	(M+Na)+

MS Zoomed Spectrum

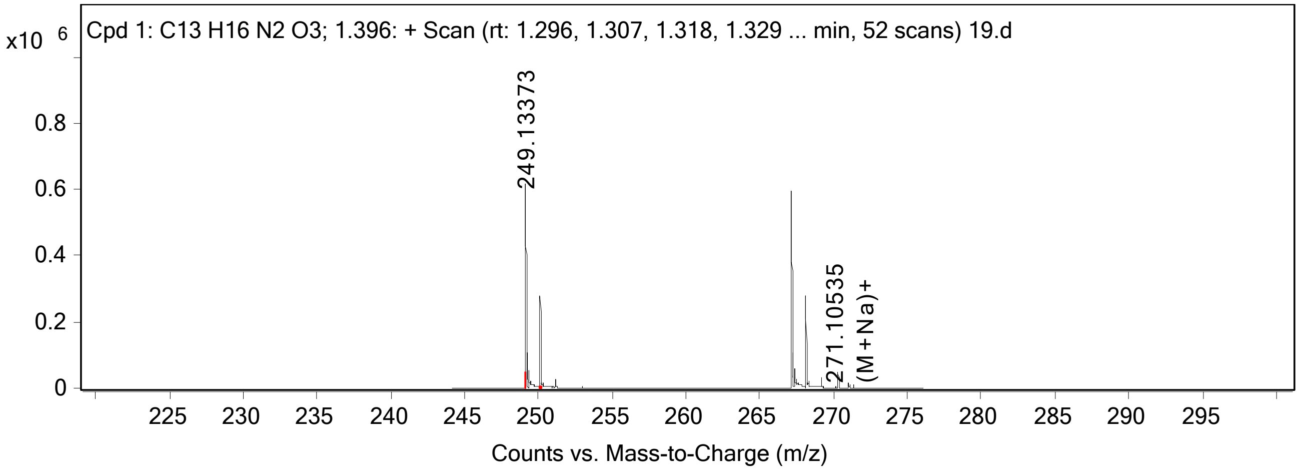

MS Spectrum Peak List

Obs. *m/z*	Charge	Abund	Ion/Isotope	Tgt Mass Error (ppm)
249.12377	1	49834.08	(M+H)+	-1.63
249.13373		620797.21		
250.12647	1	7164.95	(M+H)+	-0.02
251.12737	1	1470.44	(M+H)+	5.82
271.10535	1	1873.94	(M+Na)+	-0.14

--- End Of Report ---

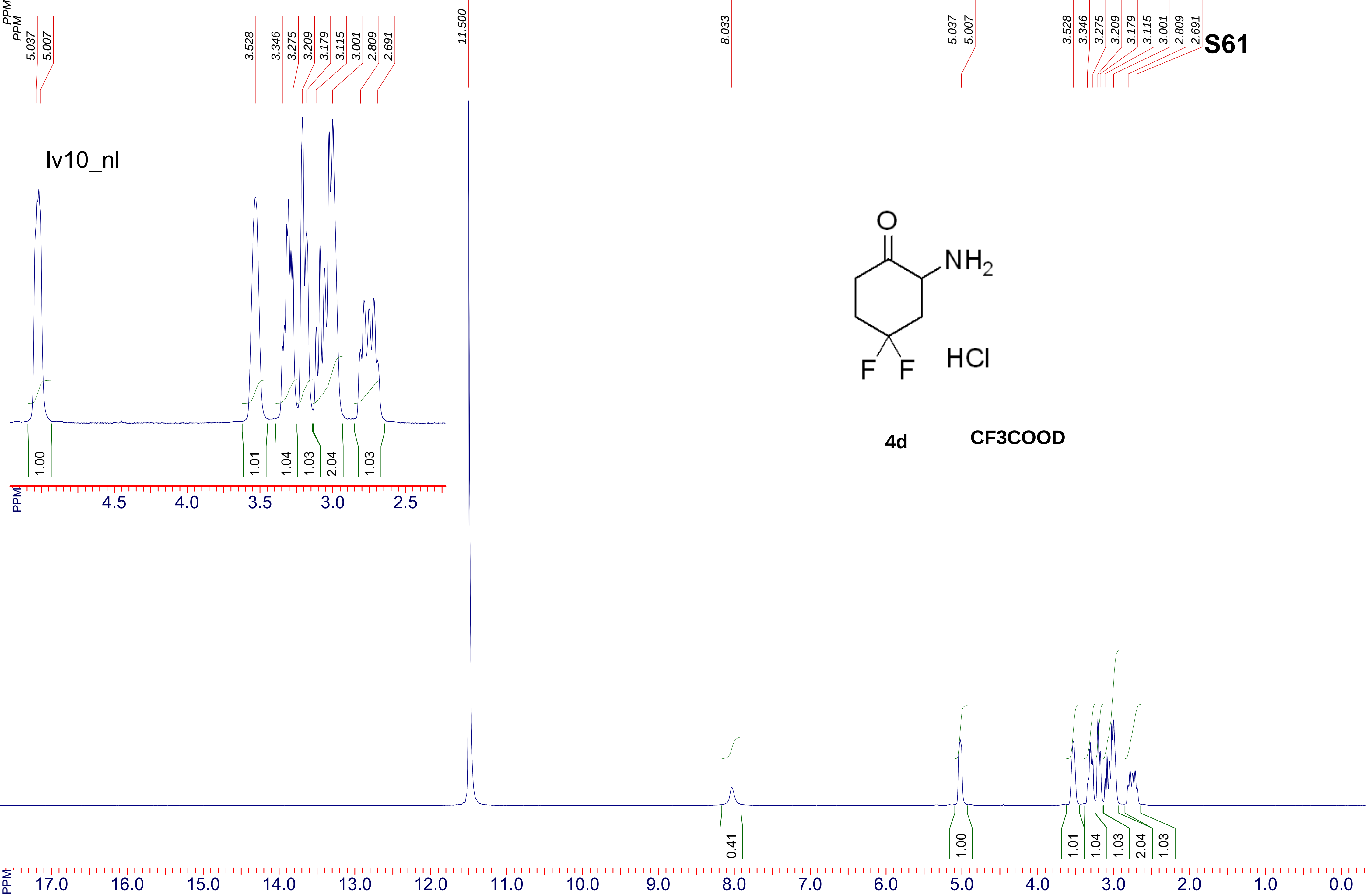

File name: lv10_nl	Operator: root	SF: 499.6730 MHz	NSC: 1	PW: 0.00 usec, RG: 32	SI: 32768
Date: 31-Mar-2023	Solvent: CDCl3	SW: 8993 Hz	TE: 683 K	AQ: 1.82 sec, RD: 0.00 sec	

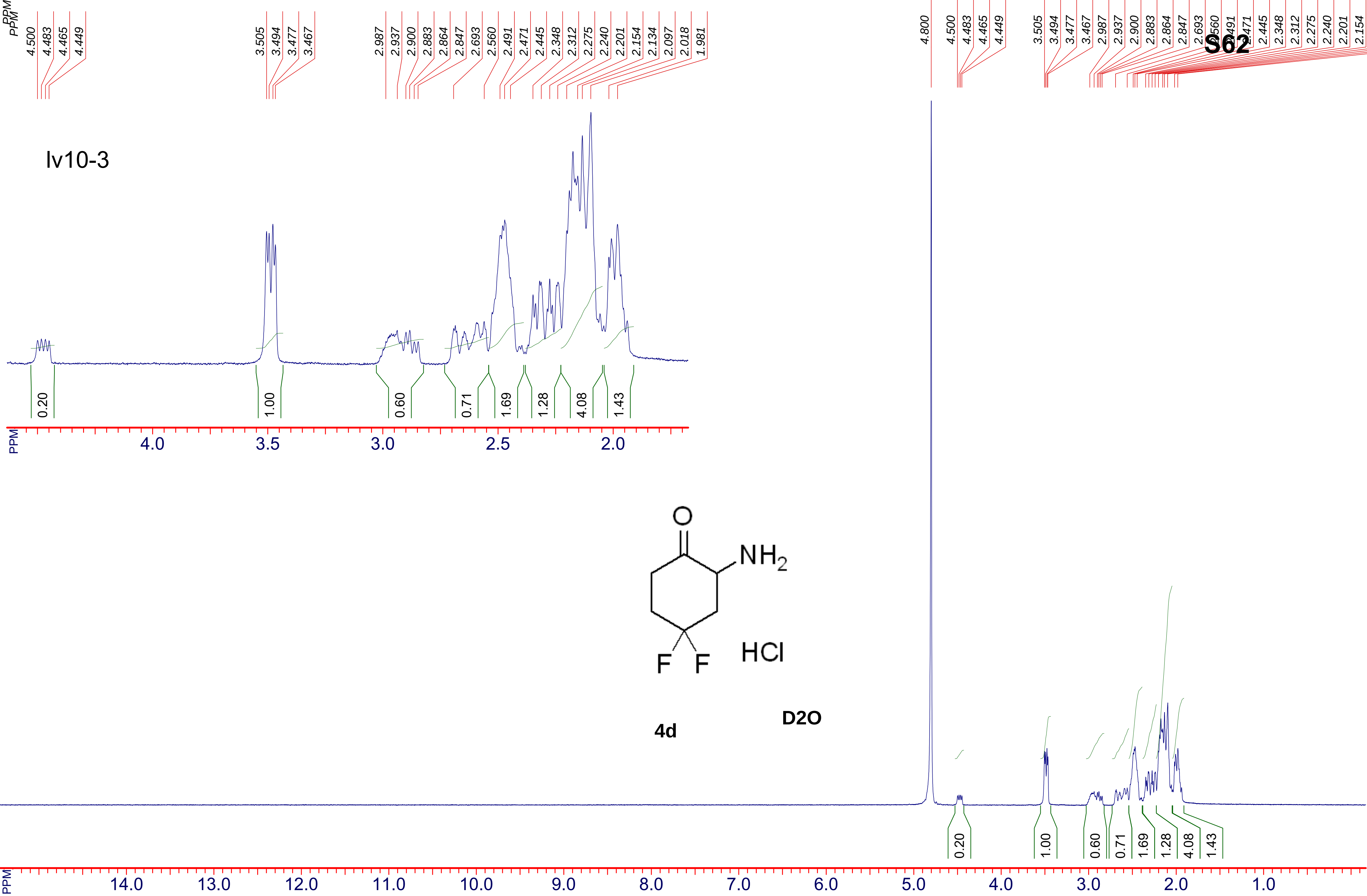

File name: lv10-3	Operator: nmr	SF: 400.1300 MHz	NSC: 1	PW: 0.00 usec, RG: 25	SI: 32768
Date: 04-Apr-2023	Solvent: CDCl3	SW: 8224 Hz	TE: 300 K	AQ: 1.99 sec, RD: 0.00 sec	

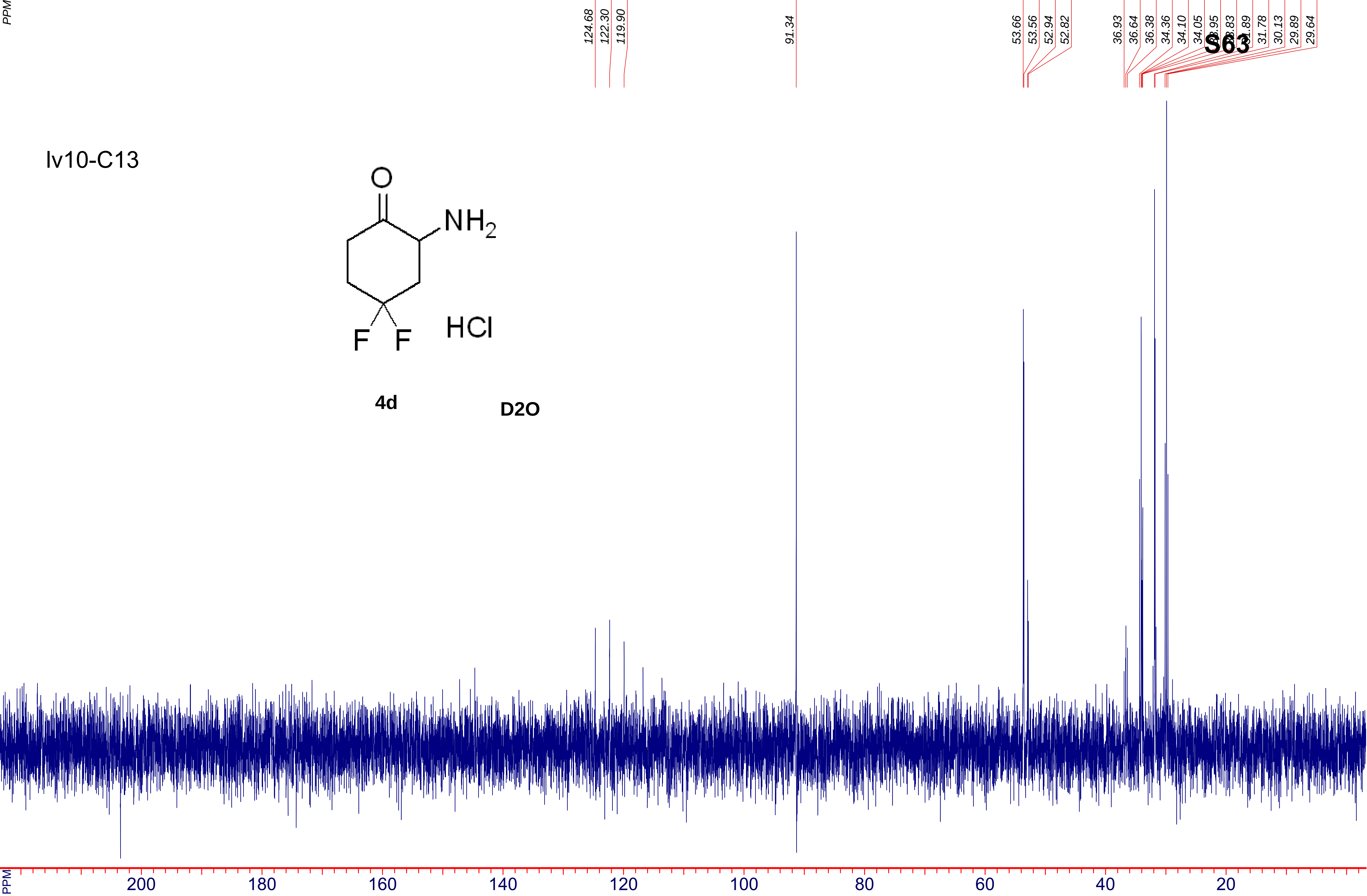

File name: lv10-C13	Operator: nmr	SF: 100.6128 MHz	NSC: 134	PW: 0.00 usec, RG: 2050	SI: 32768
Date: 03-Apr-2023	Solvent: D2O	SW: 26042 Hz	TE: 300 K	AQ: 0.98 sec, RD: 0.00 sec	

lv10_F19{H}

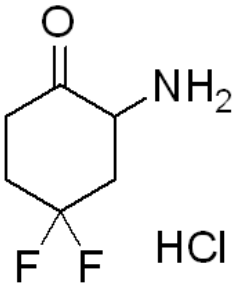

4d

D2O

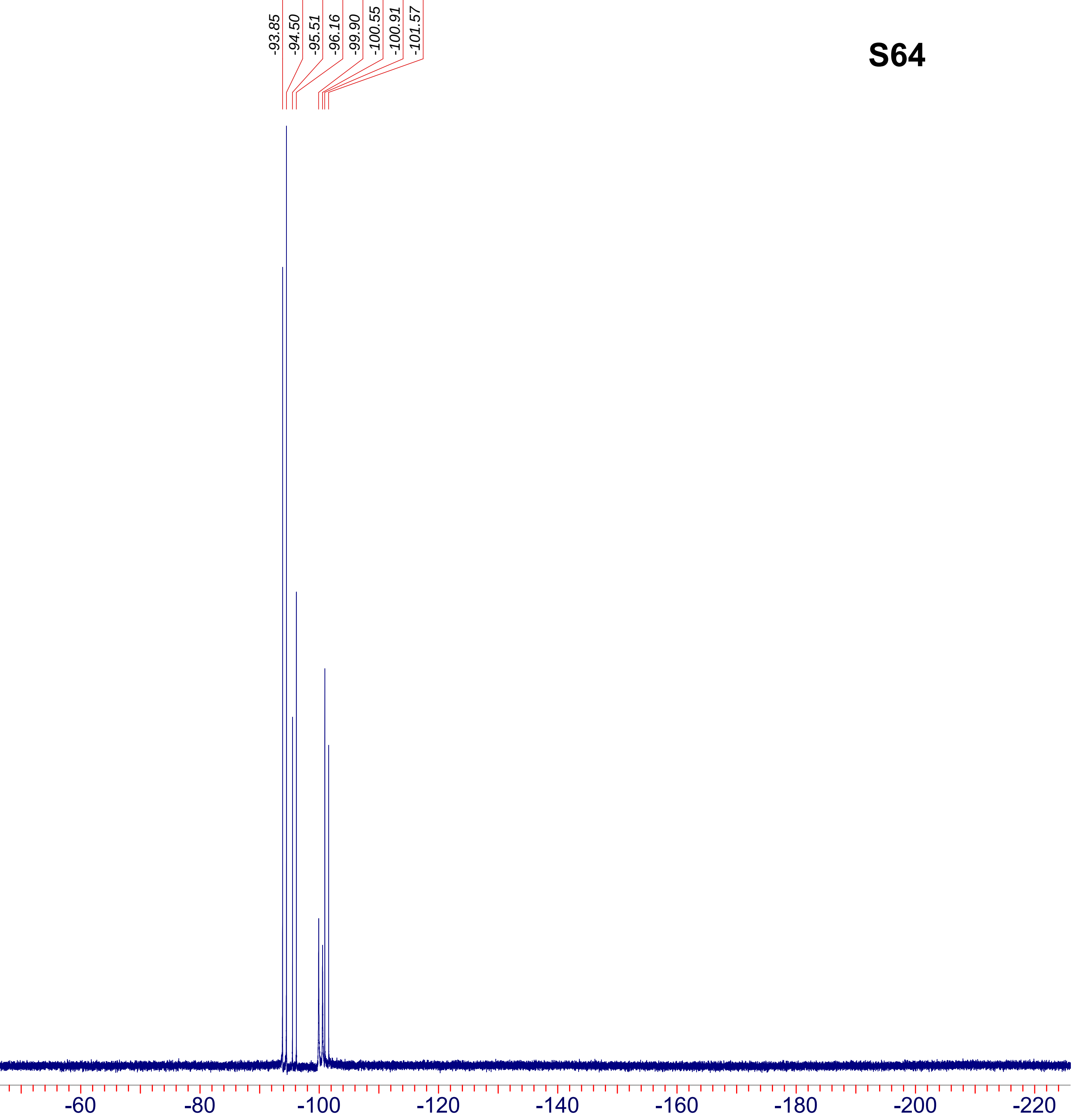

File name: lv10_F19{H}	Operator: nmr	SF: 376.4986 MHz	NSC: 1	PW: 0.00 usec, RG: 912	SI: 262144
Date: 03-Apr-2023	Solvent: D2O	SW: 138889 Hz	TE: 300 K	AQ: 0.94 sec, RD: 0.00 sec	

Target Compound Screening Report

**Data File**	17c.d	**Sample Name**	10
**Sample Type**	Sample	**Position**	P1-B8
**Instrument Name**	Instrument 1	**User Name**	Denis V.Bylina
**Acq Method**	Fast_Gradient_HRMS_pos_Lock_01312023.m	**Acquired Time**	4/7/2023 4:21:13 PM (UTC+03:00)
**IRM Calibration Status**	Success	**DA Method**	1.m
**Comment**	Lysenko		
**Sample Group**		**Info.**	Agilent 6224 TOF LC/MS
**MFC**	C6H9F2NO	**Stream Name**	LC 1
**Acquisition Time (Local)**	4/7/2023 4:21:13 PM (UTC+03:00)	**Acquisition SW Version**	6200 series TOF/6500 series Q-TOF B.08.00 (B8058.0)
**TOF Driver Version**	8.00.00	**TOF Firmware Version**	8.643
**Tune Mass Range Max.**	1700		

Compound Table

Label	Tgt Score	Mass Error (ppm)	Tgt Formula	Obs. RT	Ref. Mass	Obs. Mass
Cpd 2: C6 H9 F2 N O; 1.483	99.19	0.81	C6 H9 F2 N O	1.483	149.0652	149.0653

Obs. *m/z*	Obs. RT	Obs. Mass	Tgt Formula	Tgt Mass	Tgt Mass Error (ppm)	RT Diff.	Find Cpds Algorithm
150.0726	1.483	149.0653	C6 H9 F2 N O	149.0652	0.81	Find By Formula	

Compound Chromatograms

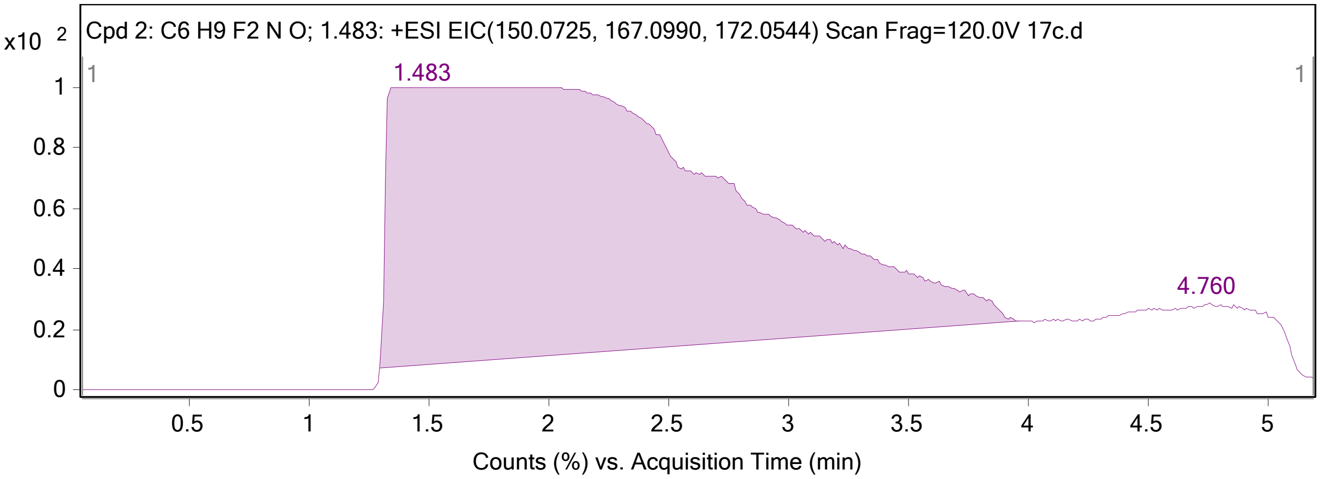

MS Zoomed Spectrum

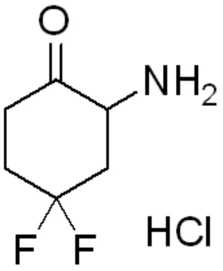

4d

Target Compound Screening Report

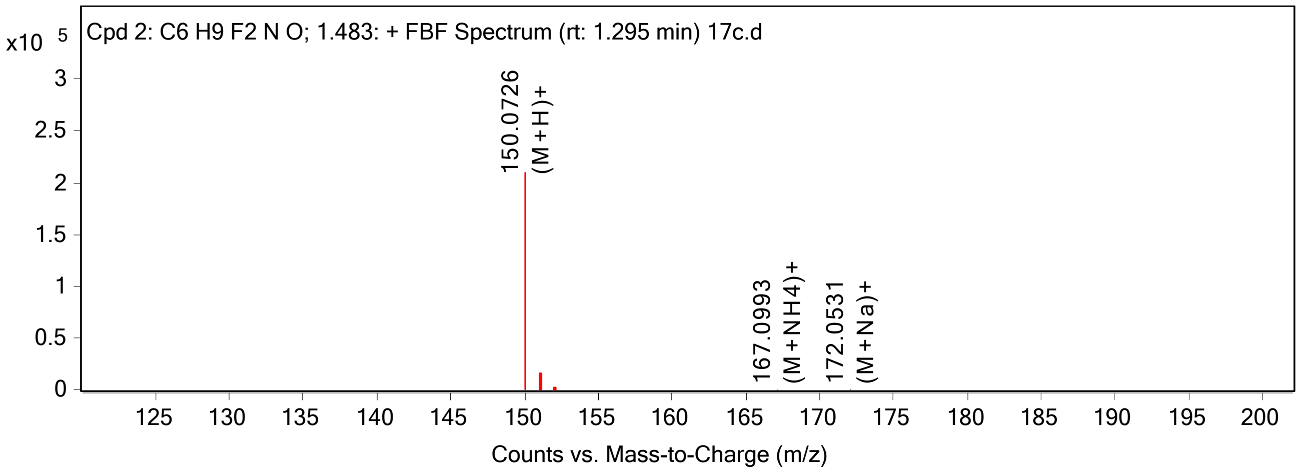

MS Spectrum Peak List

Obs. *m/z*	Charge	Abund	Ion/Isotope
150.0726	1	209241.02	(M+H)+
151.0759	1	11925.3	(M+H)+
152.0759	1	1091.99	(M+H)+
167.0993	1	143.17	(M+NH4)+
172.0531	1	374.35	(M+Na)+

MS Zoomed Spectrum

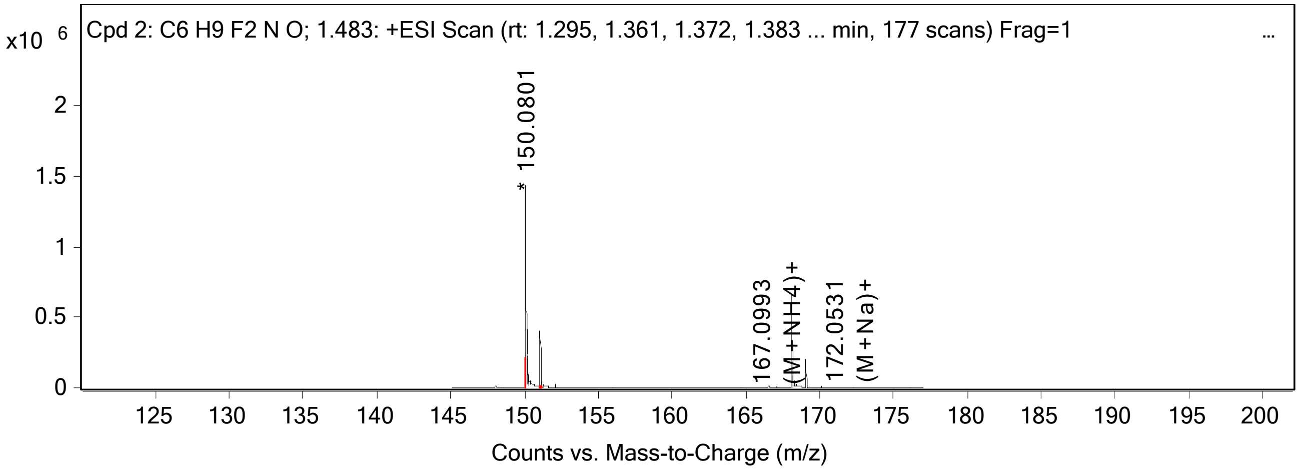

MS Spectrum Peak List

Obs. *m/z*	Charge	Abund	Ion/Isotope	Tgt Mass Error (ppm)
150.0726	1	209241.02	(M+H)+	-0.82
150.0801		1442317.36		
151.0759	1	11925.3	(M+H)+	-1.95
152.0759	1	1091.99	(M+H)+	11.66
167.0993	1	143.17	(M+NH4)+	-1.76
172.0531	1	374.35	(M+Na)+	8.07

--- End Of Report ---

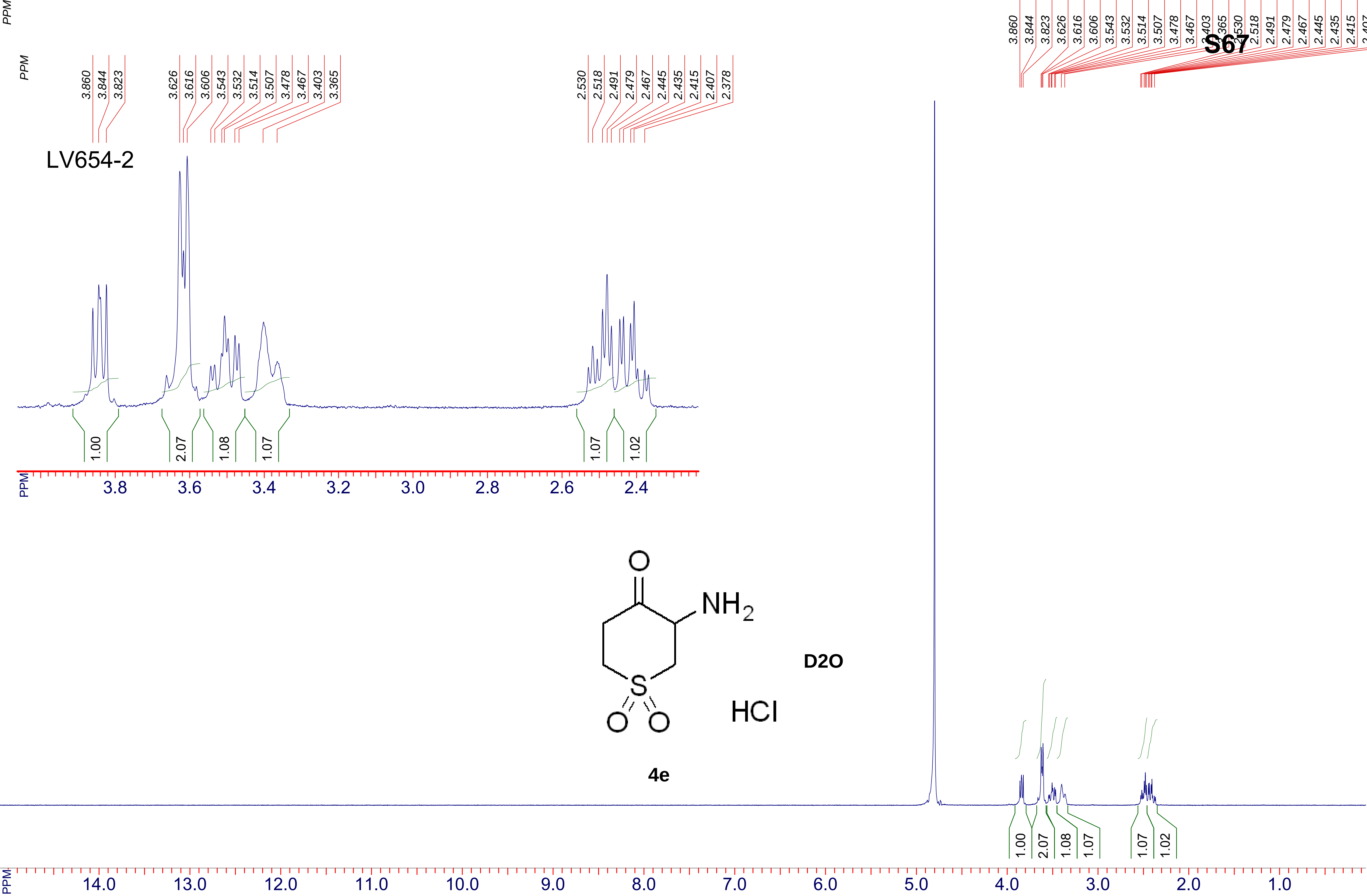

**File name: LV654-2**	Operator: nmr	SF: 400.1300 MHz	NSC: 1	PW: 0.00 usec, RG: 32	SI: 32768
Date: 29-Nov-2022	Solvent: CDCl3	SW: 8224 Hz	TE: 300 K	AQ: 1.99 sec, RD: 0.00 sec	

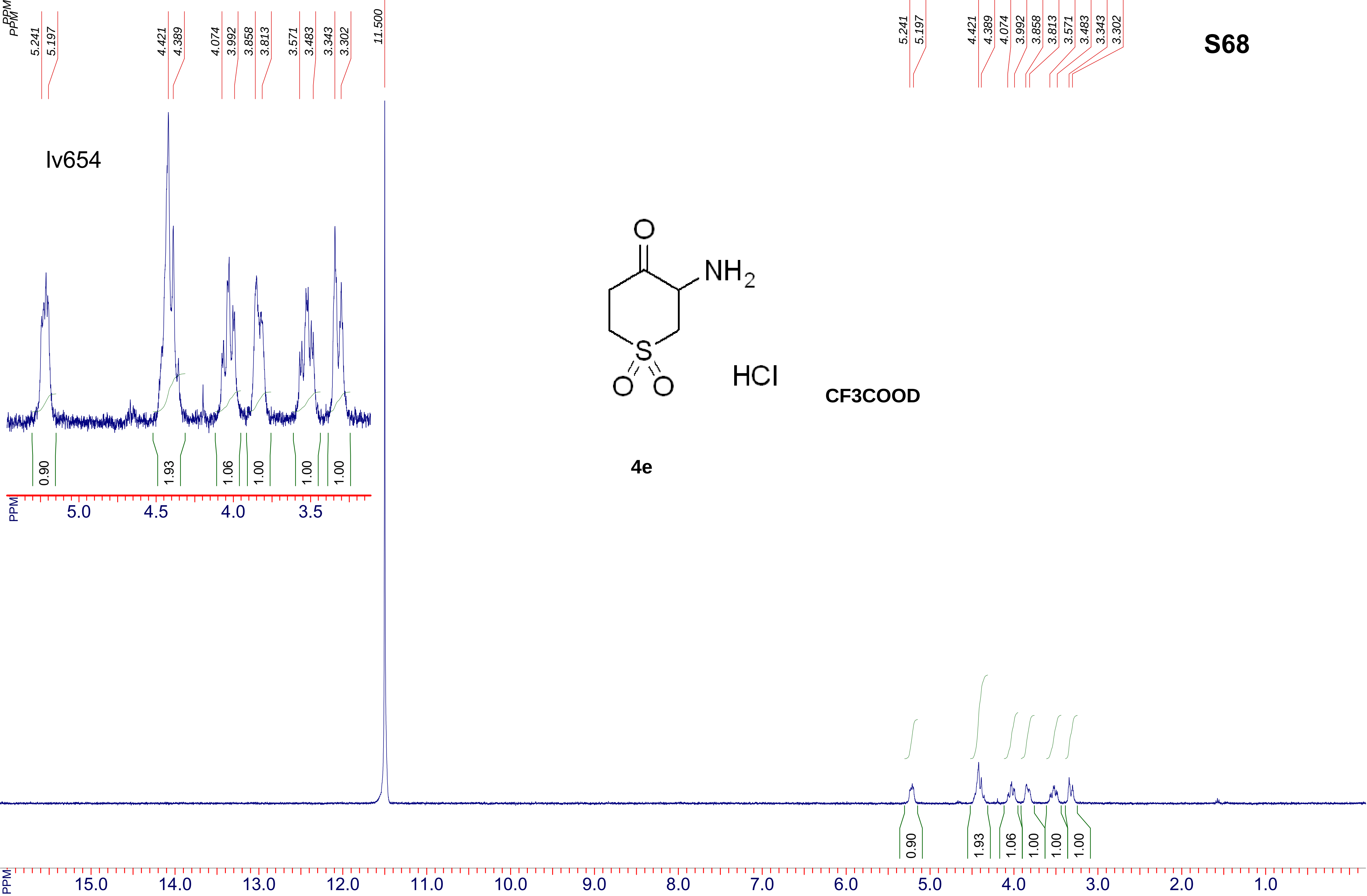

**File name: lv654**	Operator: nmr	SF: 400.1300 MHz	NSC: 1	PW: 0.00 usec, RG: 2	SI: 32768
Date: 14-Dec-2022	Solvent: DMSO	SW: 8224 Hz	TE: 300 K	AQ: 1.99 sec, RD: 0.00 sec	

lv654

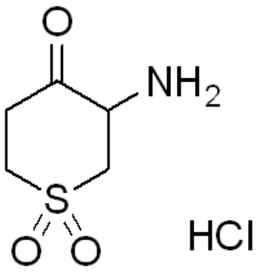

DMSO-d6

4e

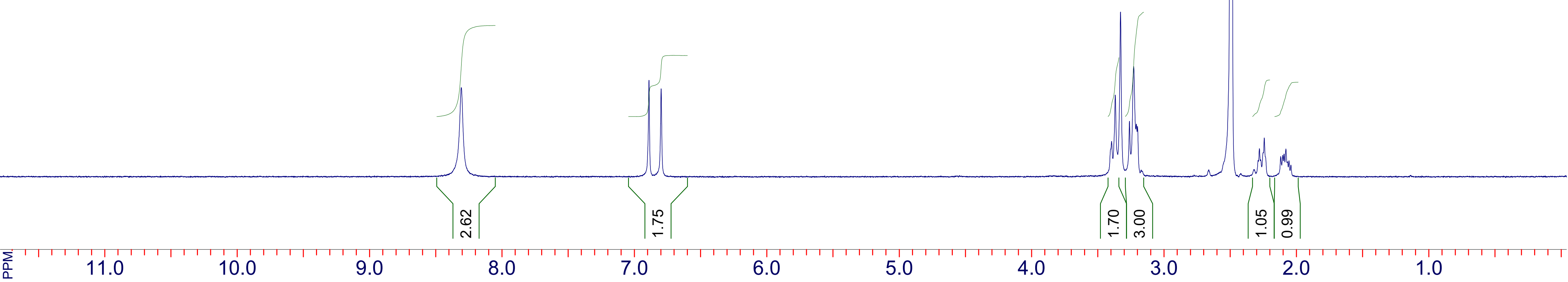

**File name: lv654**	Operator: nmr	SF: 400.1300 MHz	NSC: 1	PW: 0.00 usec, RG: 25	SI: 32768
Date: 30-Nov-2022	Solvent: DMSO	SW: 8224 Hz	TE: 300 K	AQ: 1.99 sec, RD: 0.00 sec	

lv33-C13

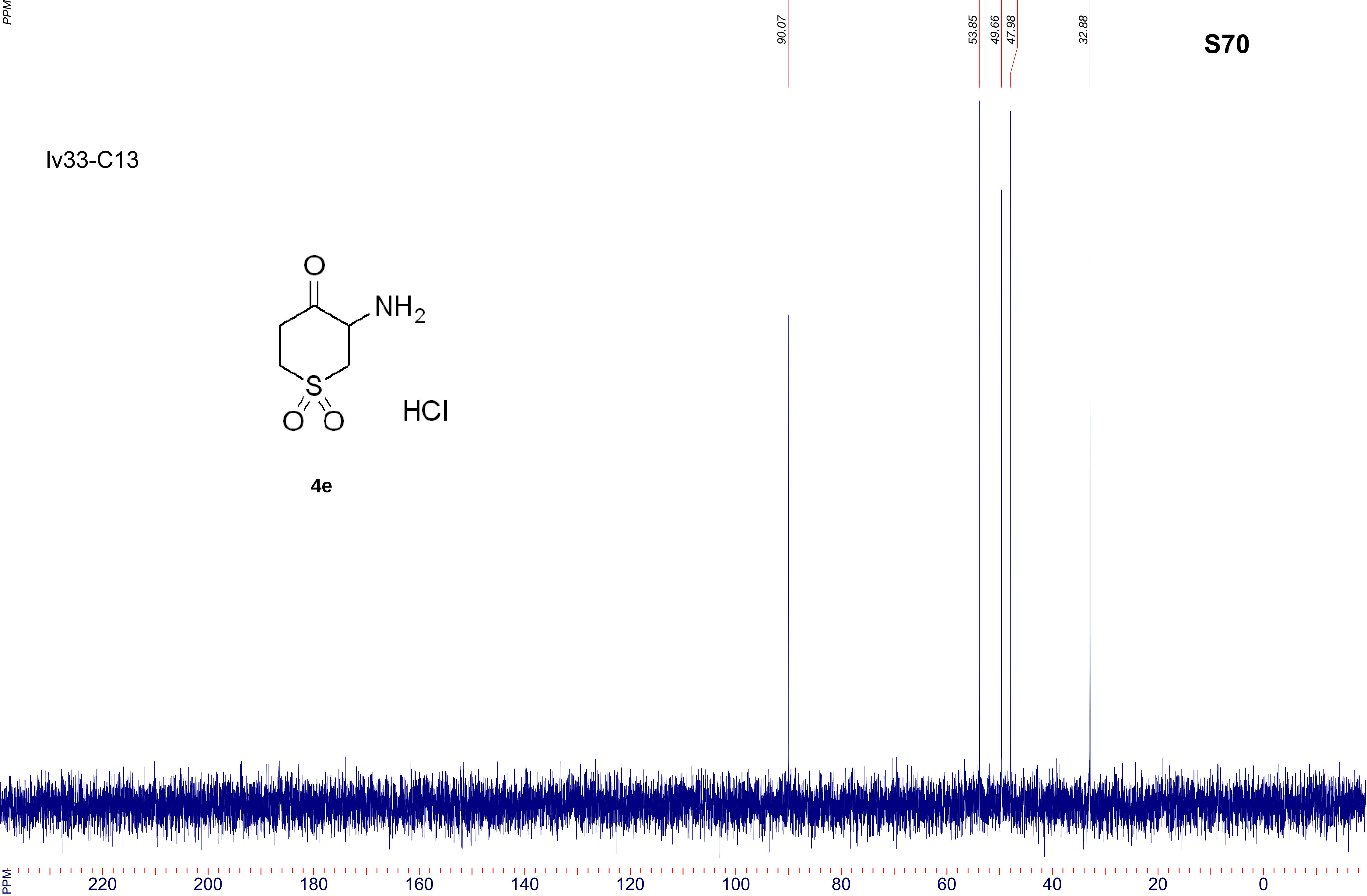

4e

**File name: lv33-C13**	Operator: nmr	SF: 100.6128 MHz	NSC: 65	PW: 0.00 usec, RG: 2050	SI: 32768
Date: 03-Apr-2023	Solvent: D2O	SW: 26042 Hz	TE: 300 K	AQ: 0.98 sec, RD: 0.00 sec	

Target Compound Screening Report

**Data File**	28c2.d	**Sample Name**	33
**Sample Type**	Sample	**Position**	P1-D1
**Instrument Name**	Instrument 1	**User Name**	Denis V.Bylina
**Acq Method**	Fast_Gradient_HRMS_pos_Lock_01312023.m	**Acquired Time**	4/7/2023 5:48:42 PM (UTC+03:00)
**IRM Calibration Status**	Success	**DA Method**	1.m
**Comment**	Lysenko		
**Sample Group**		**Info.**	Agilent 6224 TOF LC/MS
**MFC**	C5H9NO3S	**Stream Name**	LC 1
**Acquisition Time (Local)**	4/7/2023 5:48:42 PM (UTC+03:00)	**Acquisition SW Version**	6200 series TOF/6500 series Q-TOF B.08.00 (B8058.0)
**TOF Driver Version**	8.00.00	**TOF Firmware Version**	8.643
**Tune Mass Range Max.**	1700		

Compound Table

Label	Tgt Score	Mass Error (ppm)	Tgt Formula	Obs. RT	Ref. Mass	Obs. Mass
Cpd 3: C5 H9 N O3 S; 1.203	99.51	-0.66	C5 H9 N O3 S	1.203	163.0303	163.0302

Obs. *m/z*	Obs. RT	Obs. Mass	Tgt Formula	Tgt Mass	Tgt Mass Error (ppm)	RT Diff.	Find Cpds Algorithm
164.0375	1.203	163.0302	C5 H9 N O3 S	163.0303	-0.66	Find By Formula	

Compound Chromatograms

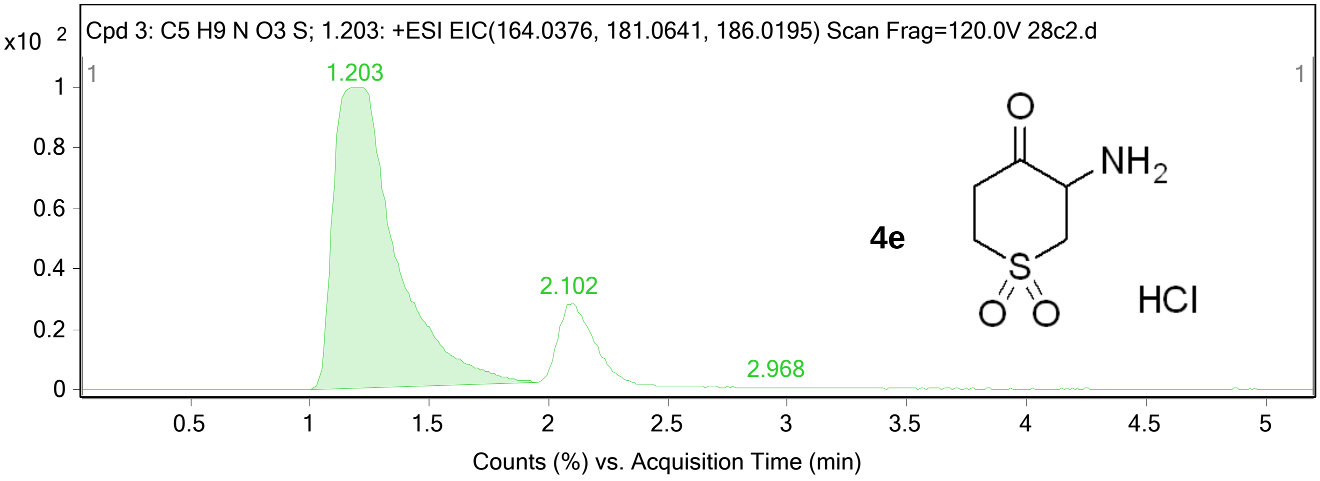

MS Zoomed Spectrum

Target Compound Screening Report

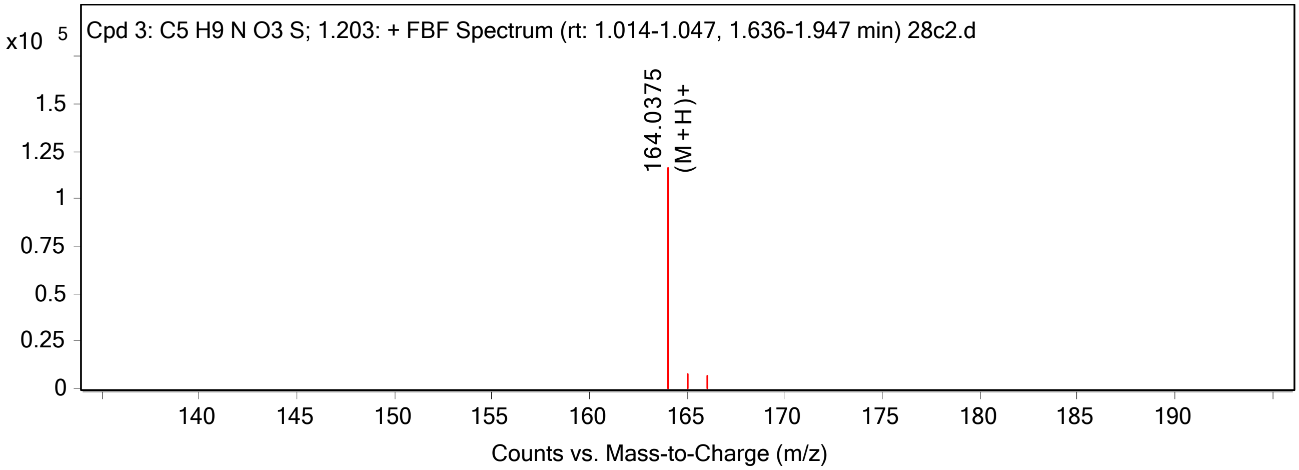

MS Spectrum Peak List

Obs. *m/z*	Charge	Abund	Ion/Isotope
164.0375	1	115803.93	(M+H)+
165.0403	1	7128.2	(M+H)+
166.0344	1	5229.91	(M+H)+

MS Zoomed Spectrum

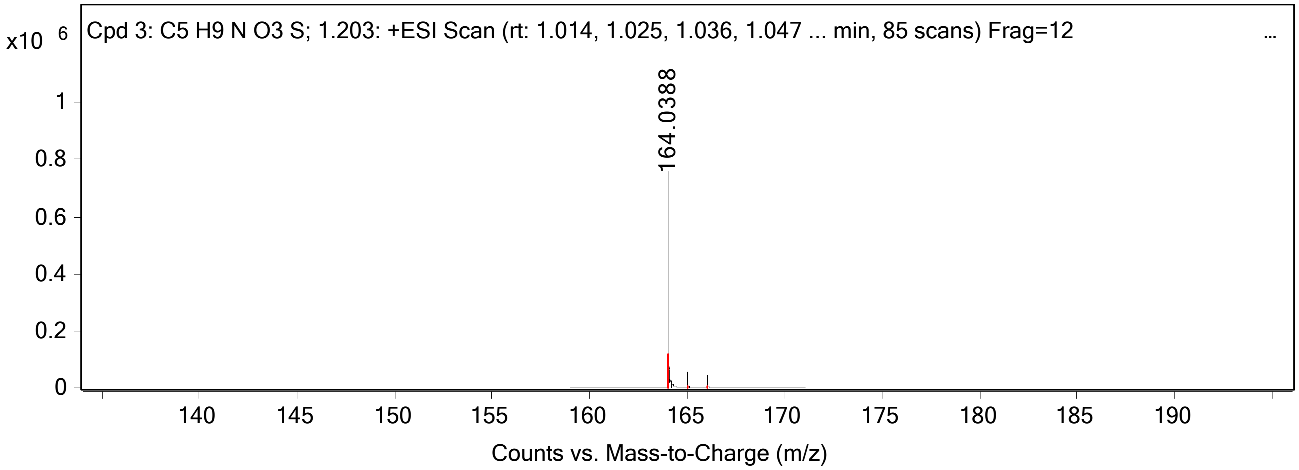

MS Spectrum Peak List

Obs. *m/z*	Charge	Abund	Ion/Isotope	Tgt Mass Error (ppm)
164.0375	1	115803.93	(M+H)+	0.7
164.0388		765441.62		
165.0403	1	7128.2	(M+H)+	-0.85
166.0344	1	5229.91	(M+H)+	1.68

--- End Of Report ---

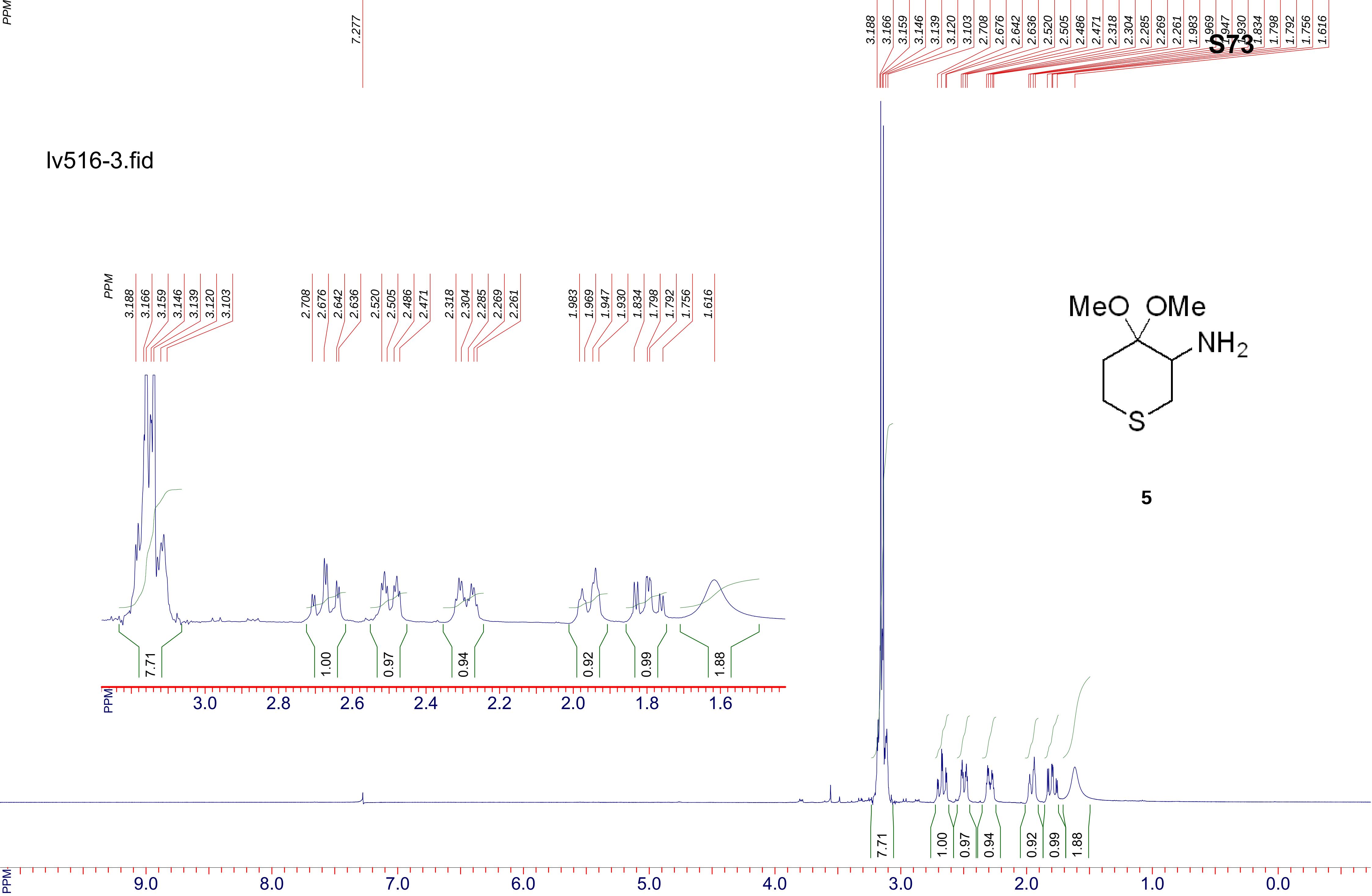

5

File name: lv516-3.fid	Operator:	SF: 399.9714 MHz	NSC: 0	PW: 10.90 usec, RG: 24	SI: 32768
Date: 15-Nov-2022	Solvent: cdcl3	SW: 8000 Hz	TE: 298 K	AQ: 2.00 sec, RD: 0.00 sec	

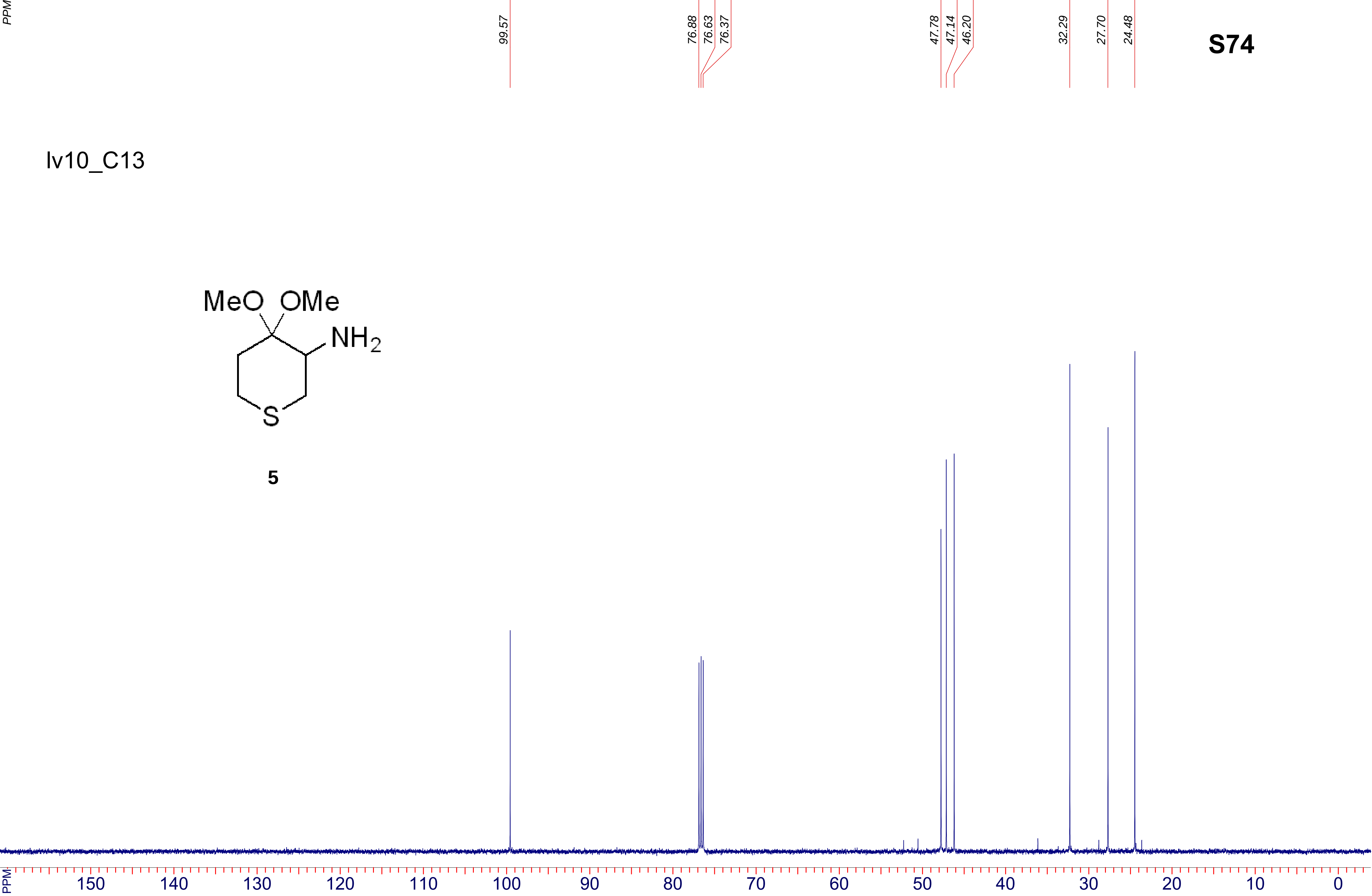

5

**File name: lv10_C13**	Operator: root	SF: 125.6429 MHz	NSC: 279	PW: 0.00 usec, RG: 51200	SI: 131072
Date: 17-Nov-2022	Solvent: CDCl3	SW: 32680 Hz	TE: 683 K	AQ: 1.57 sec, RD: 0.00 sec	

Target Compound Screening Report

**Data File**	7.d	**Sample Name**	10
**Sample Type**	Sample	**Position**	P1-A7
**Instrument Name**	Instrument 1	**User Name**	Denis V.Bylina
**Acq Method**	Fast_Gradient_HRMS_pos_Lock_08272019.m	**Acquired Time**	12/19/2022 1:32:16 PM (UTC+02:00)
**IRM Calibration Status**	Success	**DA Method**	1.m
**Comment**	Lysenko		
**Sample Group**		**Info.**	Agilent 6224 TOF LC/MS
**MFC**	C7H15NO2S	**Stream Name**	LC 1
**Acquisition Time (Local)**	12/19/2022 1:32:16 PM (UTC+02:00)	**Acquisition SW Version**	6200 series TOF/6500 series Q-TOF B.08.00 (B8058.0)
**TOF Driver Version**	8.00.00	**TOF Firmware Version**	8.643
**Tune Mass Range Max.**	1700		

Compound Table

Label	Tgt Score	Mass Error (ppm)	Tgt Formula	Obs. RT	Ref. Mass	Obs. Mass
Cpd 1: C7 H15 N O2 S; 1.037	98.52	-3.28	C7 H15 N O2 S	1.037	177.0823	177.0818

Obs. *m/z*	Obs. RT	Obs. Mass	Tgt Formula	Tgt Mass	Tgt Mass Error (ppm)	RT Diff.	Find Cpds Algorithm
178.089	1.037	177.0818	C7 H15 N O2 S	177.0823	-3.28	Find By Formula	

Compound Chromatograms

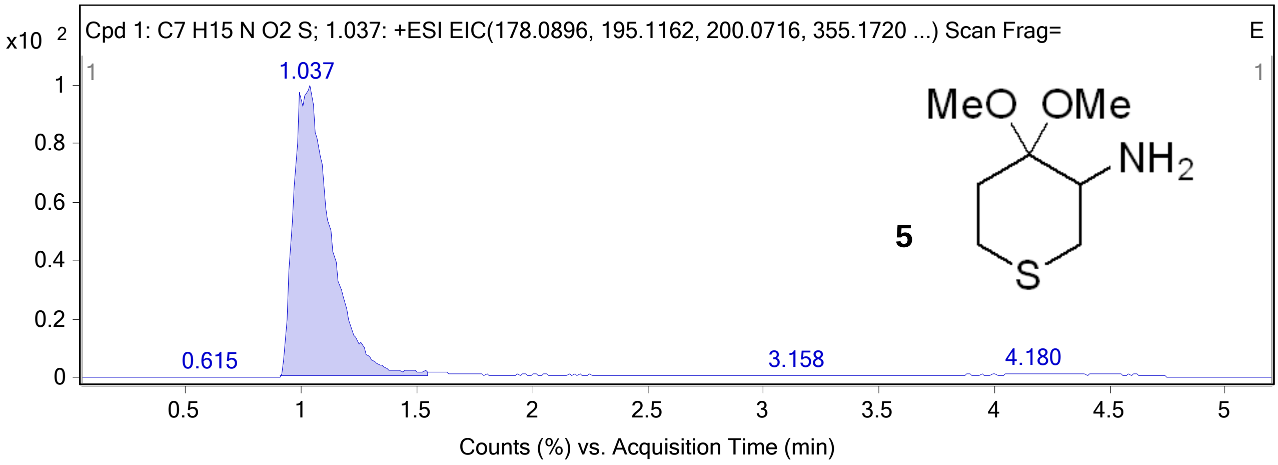

MS Zoomed Spectrum

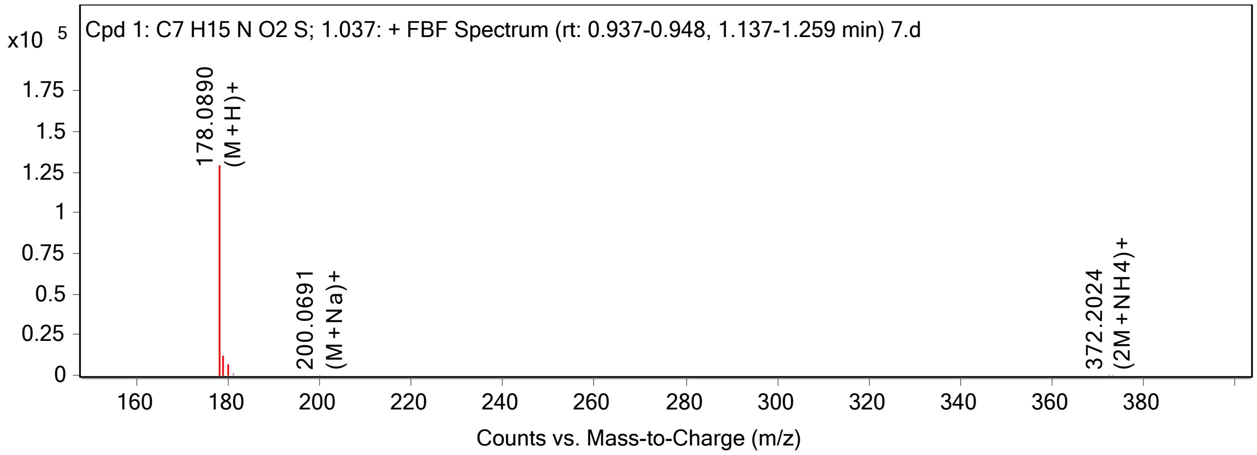

MS Spectrum Peak List

Obs. *m/z*	Charge	Abund	Ion/Isotope
178.089	1	128588.73	(M+H)+
179.092	1	10820.73	(M+H)+
180.0861	1	5726.59	(M+H)+
200.0691	1	401.93	(M+Na)+
372.2024	1	148.31	(2M+NH4)+
373.1987	1	69.93	(2M+NH4)+

MS Zoomed Spectrum

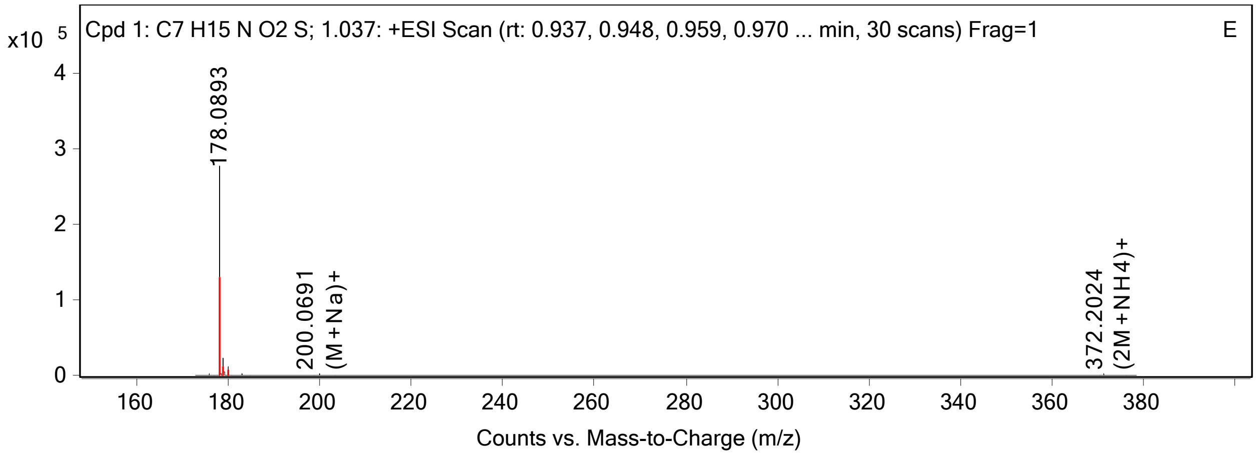

MS Spectrum Peak List

Obs. *m/z*	Charge	Abund	Ion/Isotope	Tgt Mass Error (ppm)
178.089	1	128588.72	(M+H)+	3.3
178.0893		286445.56		
179.092	1	10820.73	(M+H)+	2.24
180.0861	1	5726.59	(M+H)+	3.91
200.0691	1	401.93	(M+Na)+	12.23
372.2024	1	148.31	(2M+NH4)+	-10.43
373.1987	1	69.93	(2M+NH4)+	6.79

--- End Of Report ---

lv13.fid

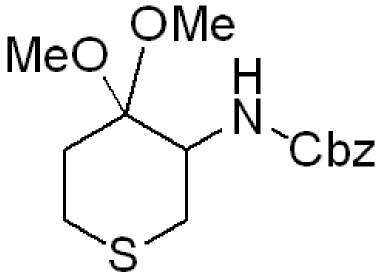

6

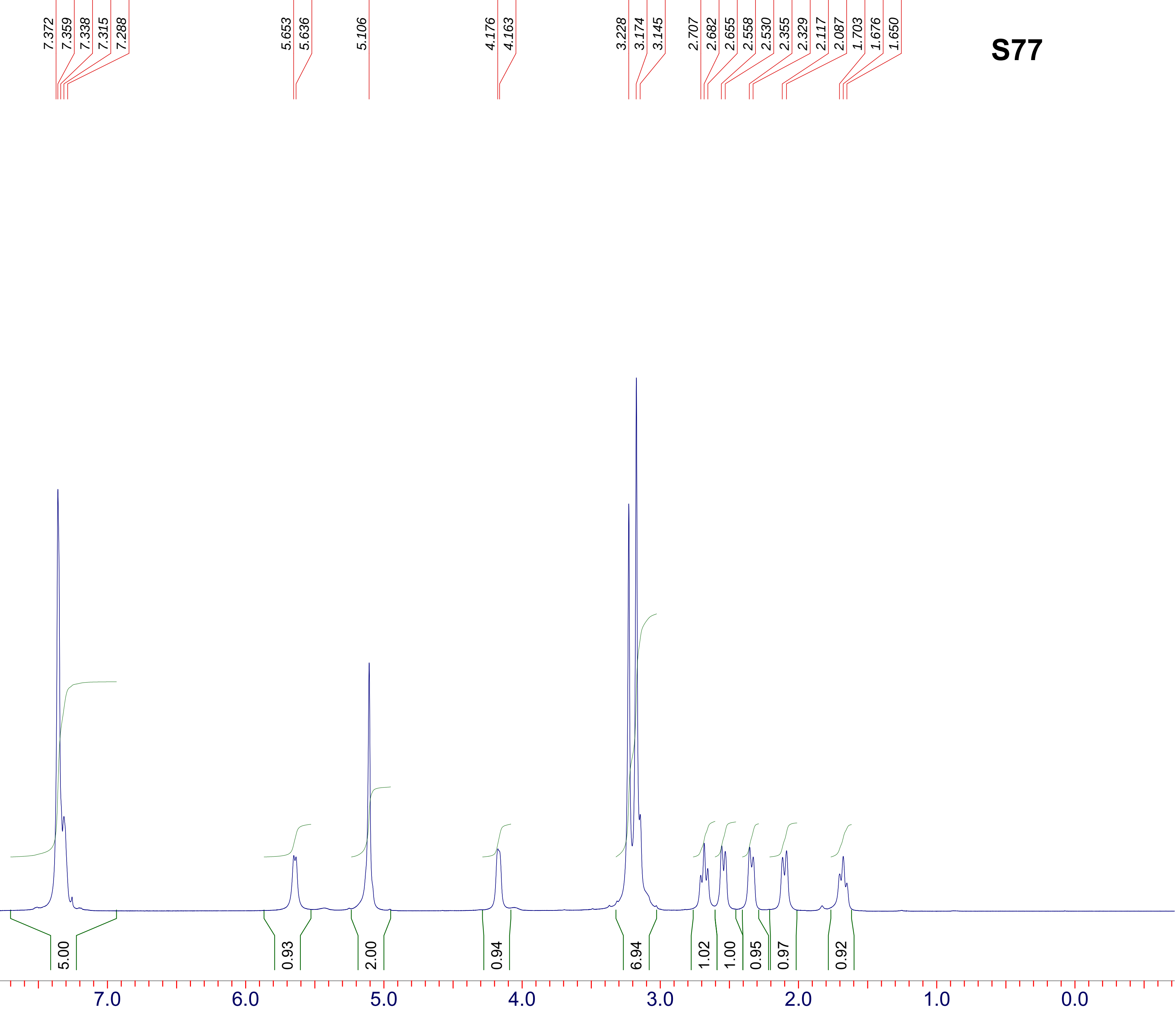

**File name: lv13.fid**	Operator:	SF: 499.8180 MHz	NSC: 0	PW: 13.60 usec, RG: 12	SI: 32768
Date: 08-Dec-2022	Solvent: cdcl3	SW: 9328 Hz	TE: 298 K	AQ: 1.72 sec, RD: 0.00 sec	

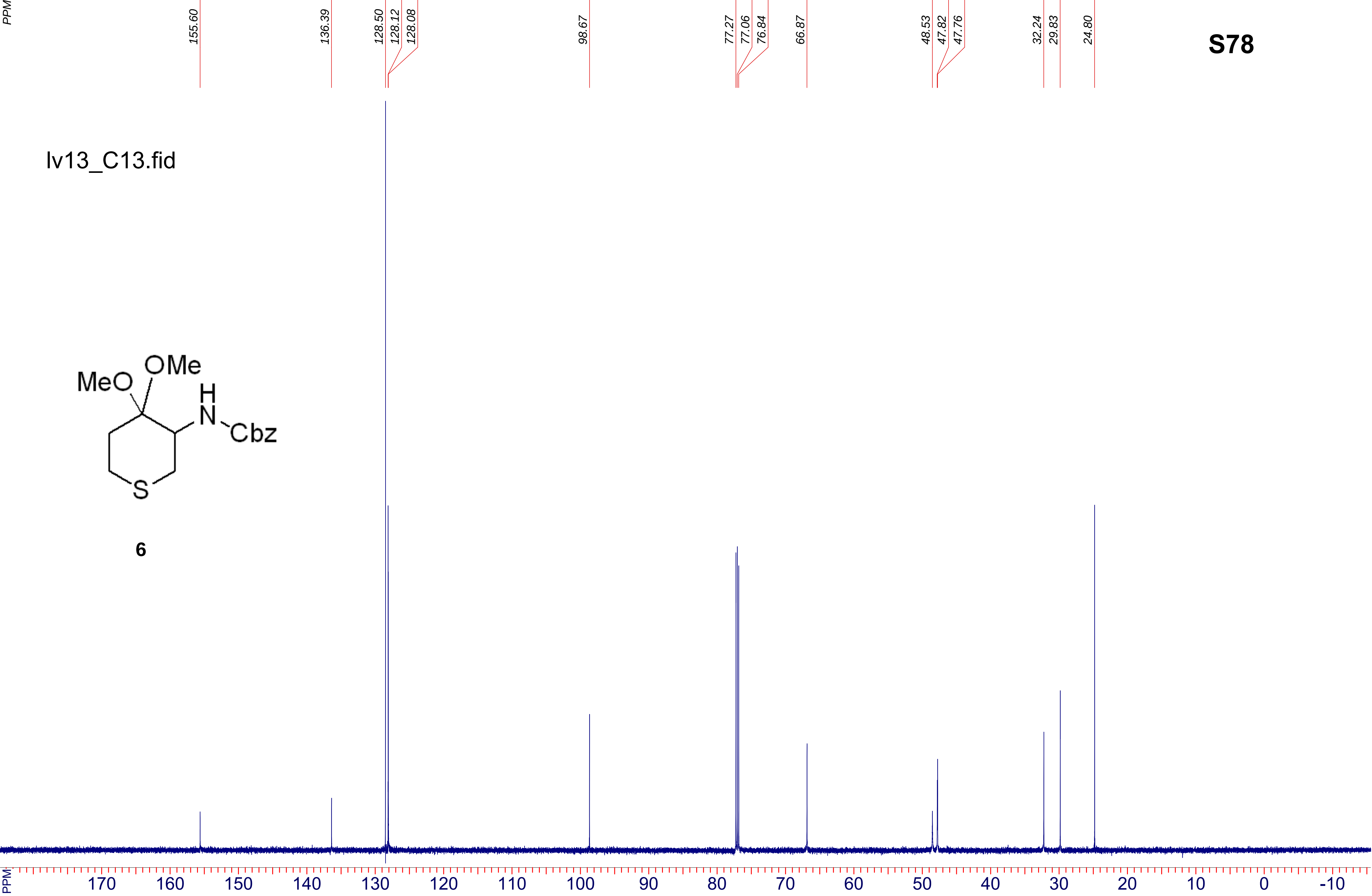

File name: lv13_C13.fid	Operator:	SF: 150.8297 MHz	NSC: 0	PW: 3.09 usec, RG: 60	SI: 65536
Date: 09-Dec-2022	Solvent: cdcl3	SW: 37879 Hz	TE: 298 K	AQ: 0.87 sec, RD: 0.00 sec	

Target Compound Screening Report

**Data File**	9.d	**Sample Name**	13
**Sample Type**	Sample	**Position**	P1-A9
**Instrument Name**	Instrument 1	**User Name**	Denis V.Bylina
**Acq Method**	Fast_Gradient_HRMS_pos_Lock_08272019.m	**Acquired Time**	12/19/2022 1:44:09 PM (UTC+02:00)
**IRM Calibration Status**	Success	**DA Method**	1.m
**Comment**	Lysenko		
**Sample Group**		**Info.**	Agilent 6224 TOF LC/MS
**MFC**	C15H21NO4S	**Stream Name**	LC 1
**Acquisition Time (Local)**	12/19/2022 1:44:09 PM (UTC+02:00)	**Acquisition SW Version**	6200 series TOF/6500 series Q-TOF B.08.00 (B8058.0)
**TOF Driver Version**	8.00.00	**TOF Firmware Version**	8.643
**Tune Mass Range Max.**	1700		

Compound Table

Label	Tgt Score	Mass Error (ppm)	Tgt Formula	Obs. RT	Ref. Mass	Obs. Mass
Cpd 1: C15 H21 N O4 S; 3.197	98.1	-3.15	C15 H21 N O4 S	3.197	311.1191	311.1182

Obs. *m/z*	Obs. RT	Obs. Mass	Tgt Formula	Tgt Mass	Tgt Mass Error (ppm)	RT Diff.	Find Cpds Algorithm
334.1077	3.197	311.1182	C15 H21 N O4 S	311.1191	-3.15	Find By Formula	

Compound Chromatograms

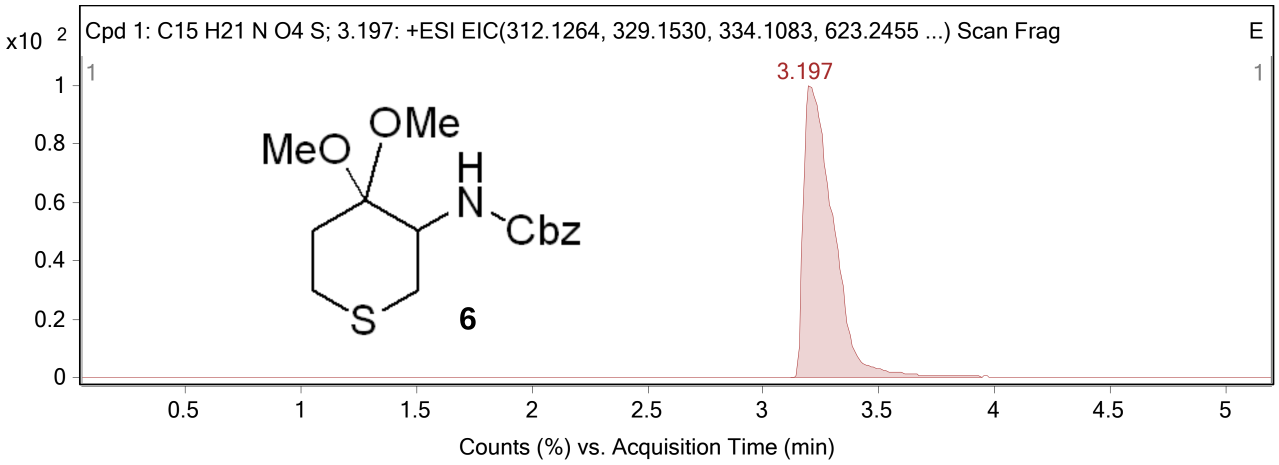

MS Zoomed Spectrum

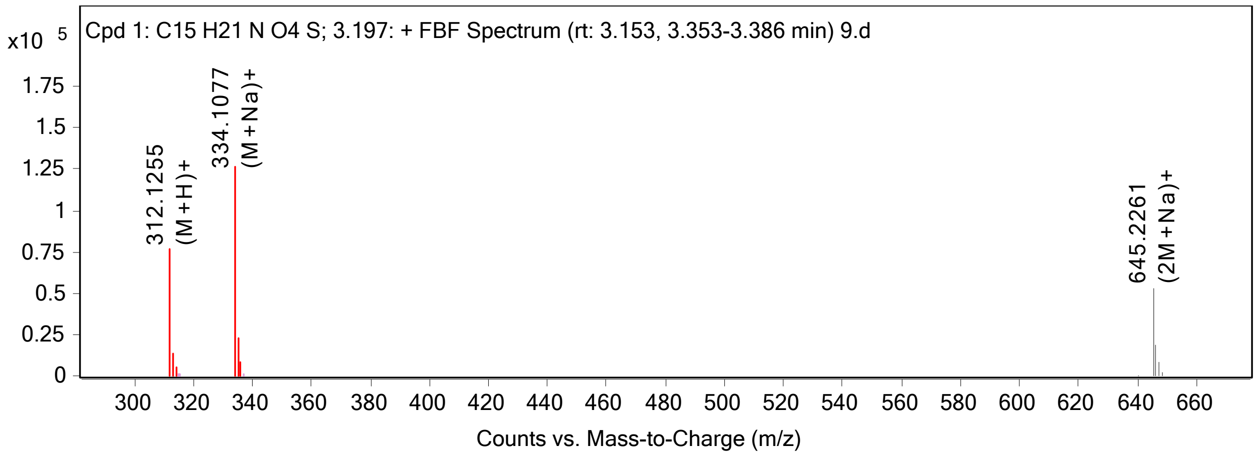

MS Spectrum Peak List

Obs. *m/z*	Charge	Abund	Ion/Isotope
312.1255	1	76666.3	(M+H)+
313.1284	1	12758.85	(M+H)+
314.1236	1	4698.27	(M+H)+
334.1077	1	126314.96	(M+Na)+
335.1105	1	20341.03	(M+Na)+
336.1059	1	7676.93	(M+Na)+
645.2261	1	53257.19	(2M+Na)+
646.229	1	18750.79	(2M+Na)+
647.2261	1	8239.29	(2M+Na)+
648.2261	1	1975.52	(2M+Na)+

MS Zoomed Spectrum

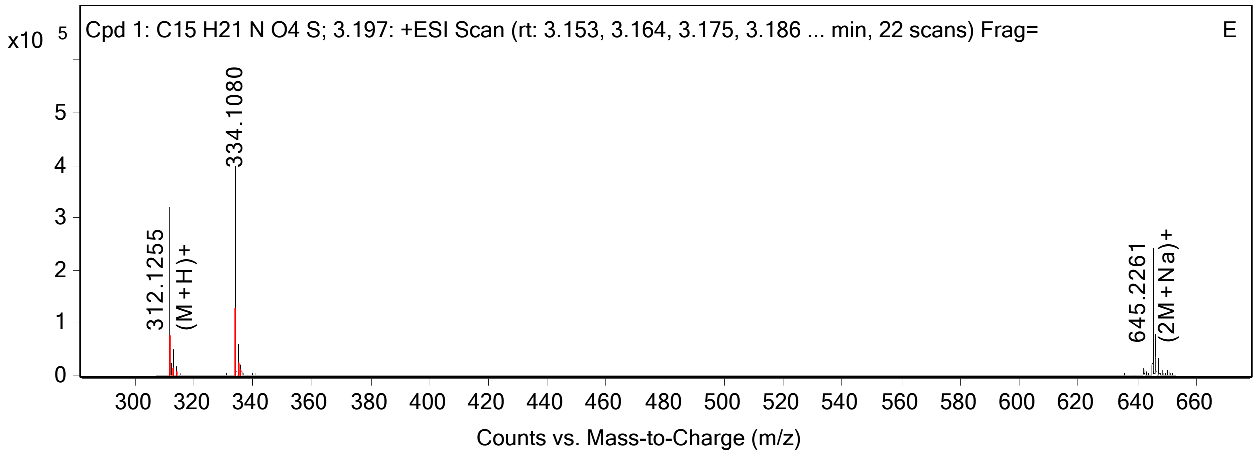

MS Spectrum Peak List

Obs. *m/z*	Charge	Abund	Ion/Isotope	Tgt Mass Error (ppm)
312.1255	1	76666.29	(M+H)+	2.88
313.1284	1	12758.85	(M+H)+	3.47
314.1236	1	4698.27	(M+H)+	6.01
334.1077	1	126314.96	(M+Na)+	1.92
335.1105	1	20341.03	(M+Na)+	2.78
336.1059	1	7676.93	(M+Na)+	4.63
645.2261	1	53257.19	(2M+Na)+	2.06
646.229	1	18750.79	(2M+Na)+	2.42
647.2261	1	8239.29	(2M+Na)+	2.73
648.2261	1	1975.52	(2M+Na)+	4.71

--- End Of Report ---

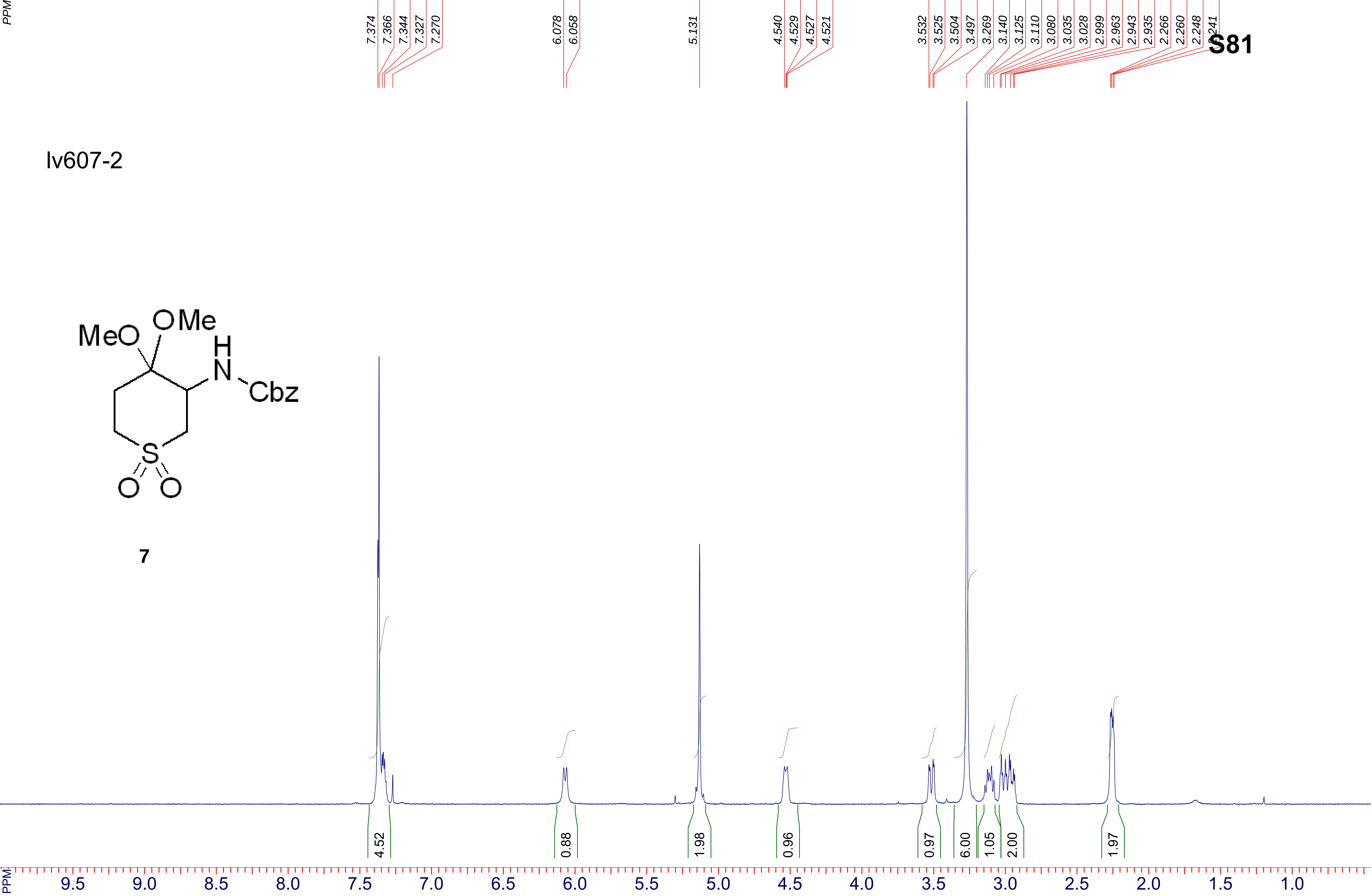

**File name: lv607-2**	Operator: root	SF: 499.6730 MHz	NSC: 1	PW: 0.00 usec, RG: 32	SI: 32768
Date: 18-Nov-2022	Solvent: CDCl3	SW: 8993 Hz	TE: 683 K	AQ: 1.82 sec, RD: 0.00 sec	

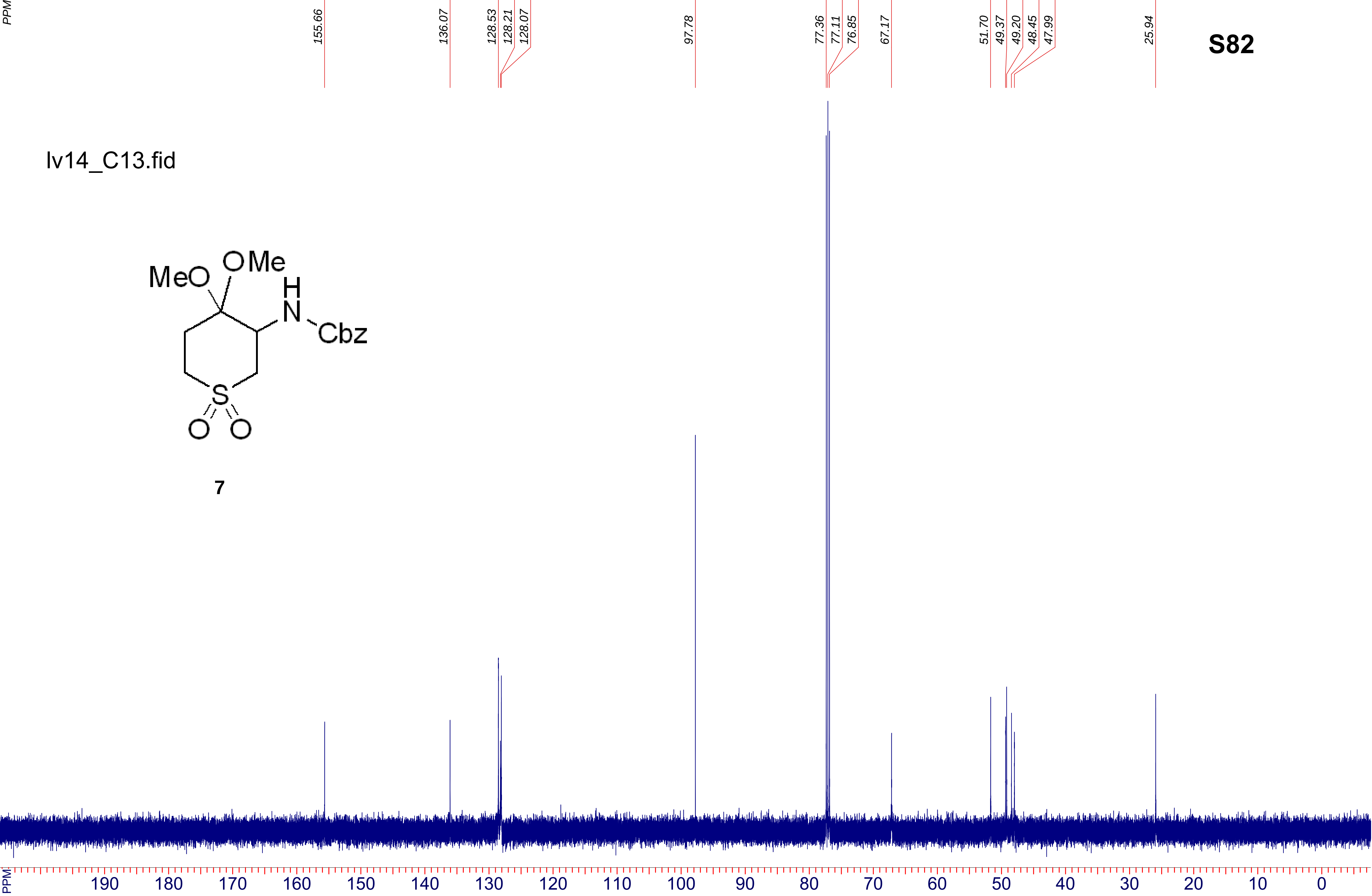

File name: lv14_C13.fid	Operator:	SF: 125.6920 MHz	NSC: 0	PW: 3.33 usec, RG: 60	SI: 131072
Date: 19-Nov-2022	Solvent: cdcl3	SW: 31250 Hz	TE: 298 K	AQ: 2.05 sec, RD: 0.00 sec	

Target Compound Screening Report

**Data File**	10.d	**Sample Name**	14
**Sample Type**	Sample	**Position**	P1-B1
**Instrument Name**	Instrument 1	**User Name**	Denis V.Bylina
**Acq Method**	Fast_Gradient_HRMS_pos_Lock_08272019.m	**Acquired Time**	12/19/2022 1:50:06 PM (UTC+02:00)
**IRM Calibration Status**	Success	**DA Method**	1.m
**Comment**	Lysenko		
**Sample Group**		**Info.**	Agilent 6224 TOF LC/MS
**MFC**	C15H21NO6S	**Stream Name**	LC 1
**Acquisition Time (Local)**	12/19/2022 1:50:06 PM (UTC+02:00)	**Acquisition SW Version**	6200 series TOF/6500 series Q-TOF B.08.00 (B8058.0)
**TOF Driver Version**	8.00.00	**TOF Firmware Version**	8.643
**Tune Mass Range Max.**	1700		

Compound Table

Label	Tgt Score	Mass Error (ppm)	Tgt Formula	Obs. RT	Ref. Mass	Obs. Mass
Cpd 1: C15 H21 N O6 S; 2.832	98.33	-2.07	C15 H21 N O6 S	2.832	343.109	343.1082

Obs. *m/z*	Obs. RT	Obs. Mass	Tgt Formula	Tgt Mass	Tgt Mass Error (ppm)	RT Diff.	Find Cpds Algorithm
361.1423	2.832	343.1082	C15 H21 N O6 S	343.109	-2.07	Find By Formula	

Compound Chromatograms

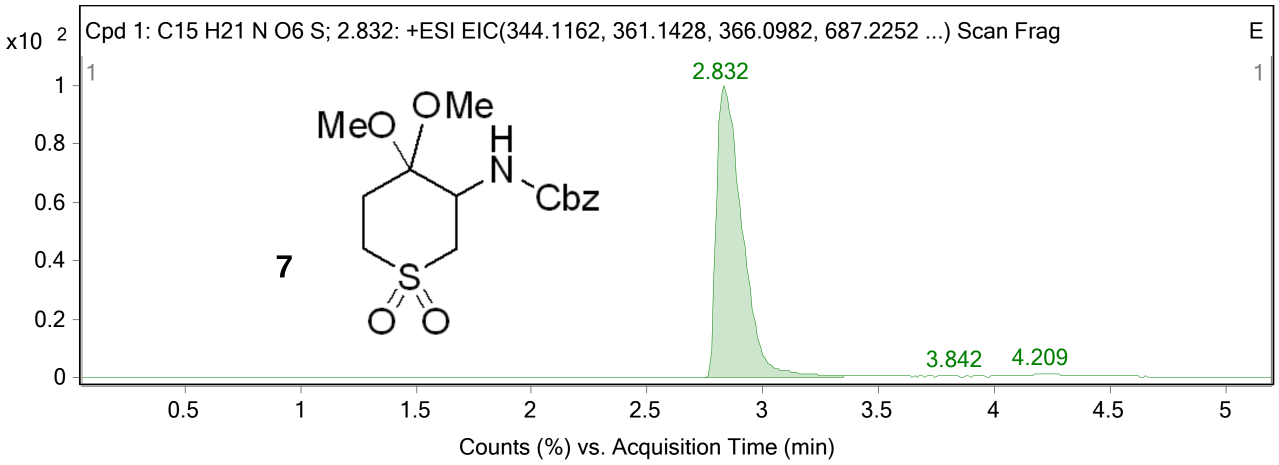

MS Zoomed Spectrum

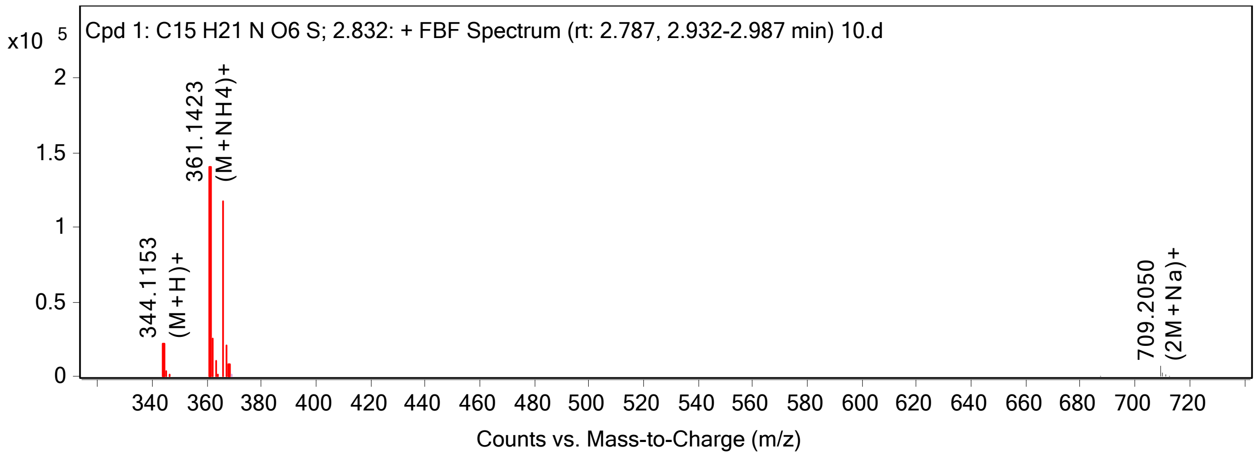

MS Spectrum Peak List

Obs. *m/z*	Charge	Abund	Ion/Isotope
344.1153	1	22222.03	(M+H)+
345.1187	1	3829.82	(M+H)+
361.1423	1	140299.92	(M+NH4)+
362.145	1	22792.53	(M+NH4)+
363.1406	1	8903.46	(M+NH4)+
366.0976	1	117579.51	(M+Na)+
367.1005	1	18165.14	(M+Na)+
368.0958	1	7200.63	(M+Na)+
709.205	1	7080.06	(2M+Na)+
710.2074	1	2719.08	(2M+Na)+

MS Zoomed Spectrum

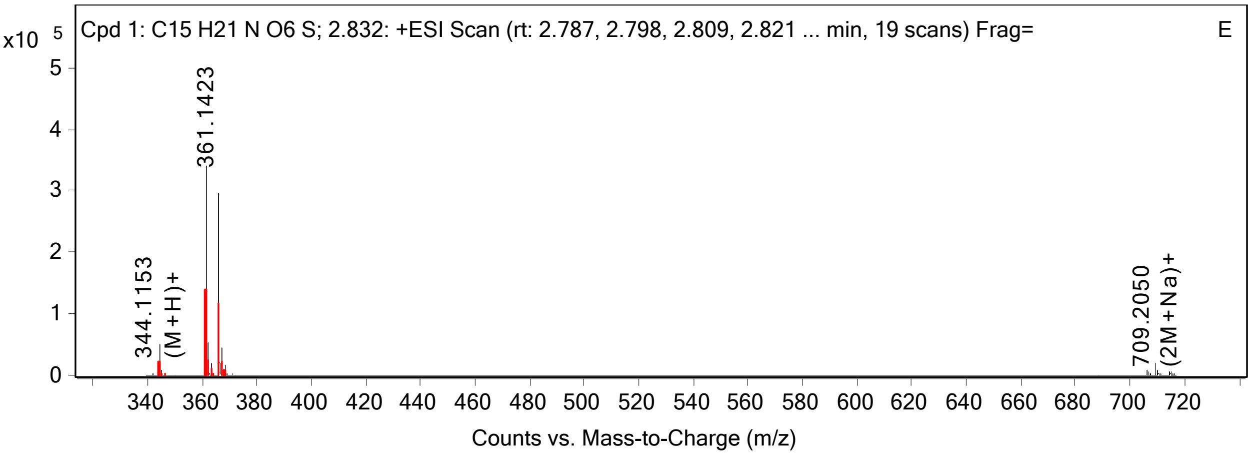

MS Spectrum Peak List

Obs. *m/z*	Charge	Abund	Ion/Isotope	Tgt Mass Error (ppm)
344.1153	1	22222.03	(M+H)+	2.77
345.1187	1	3829.82	(M+H)+	1.75
361.1423	1	140299.92	(M+NH4)+	1.35
362.145	1	22792.53	(M+NH4)+	2.07
363.1406	1	8903.46	(M+NH4)+	4.44
366.0976	1	117579.51	(M+Na)+	1.65
367.1005	1	18165.15	(M+Na)+	2.05
368.0958	1	7200.63	(M+Na)+	4.89
709.205	1	7080.06	(2M+Na)+	3.06
710.2074	1	2719.08	(2M+Na)+	4.03

--- End Of Report ---

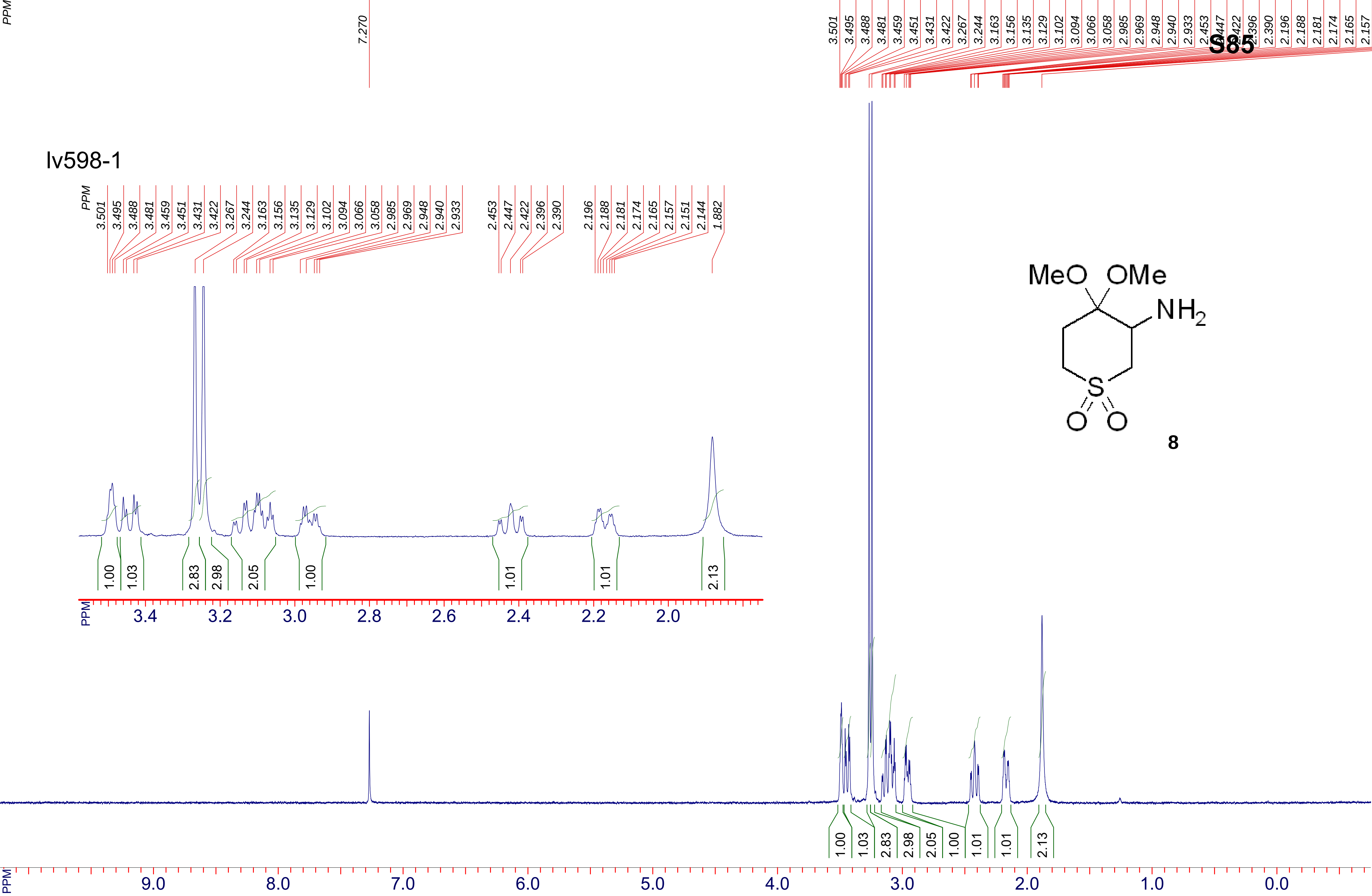

File name: lv598-1	Operator: root	SF: 499.6730 MHz	NSC: 1	PW: 0.00 usec, RG: 32	SI: 32768
Date: 21-Nov-2022	Solvent: CDCl3	SW: 8993 Hz	TE: 683 K	AQ: 1.82 sec, RD: 0.00 sec	

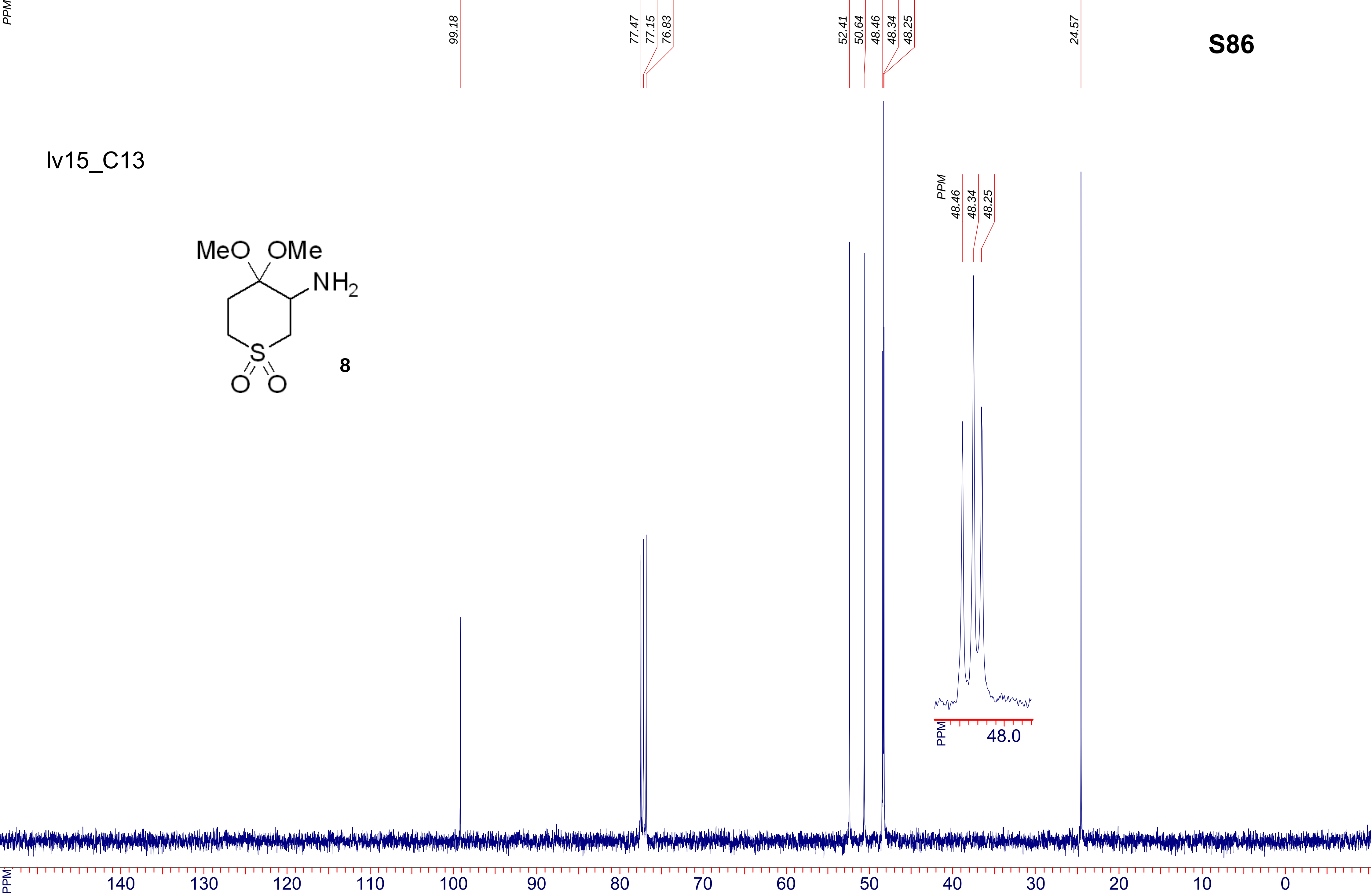

**File name: lv15_C13**	Operator: nmr	SF: 100.6128 MHz	NSC: 163	PW: 0.00 usec, RG: 2050	SI: 32768
Date: 06-Dec-2022	Solvent: CDCl3	SW: 26042 Hz	TE: 300 K	AQ: 0.98 sec, RD: 0.00 sec	Parameter file, TOPSPINVersion 2.1

Target Compound Screening Report

**Data File**	11.d	**Sample Name**	15
**Sample Type**	Sample	**Position**	P1-B2
**Instrument Name**	Instrument 1	**User Name**	Denis V.Bylina
**Acq Method**	Fast_Gradient_HRMS_pos_Lock_08272019.m	**Acquired Time**	12/19/2022 1:56:04 PM (UTC+02:00)
**IRM Calibration Status**	Success	**DA Method**	1.m
**Comment**	Lysenko		
**Sample Group**		**Info.**	Agilent 6224 TOF LC/MS
**MFC**	C7H15NO4S	**Stream Name**	LC 1
**Acquisition Time (Local)**	12/19/2022 1:56:04 PM (UTC+02:00)	**Acquisition SW Version**	6200 series TOF/6500 series Q-TOF B.08.00 (B8058.0)
**TOF Driver Version**	8.00.00	**TOF Firmware Version**	8.643
**Tune Mass Range Max.**	1700		

Compound Table

Label	Tgt Score	Mass Error (ppm)	Tgt Formula	Obs. RT	Ref. Mass	Obs. Mass
Cpd 1: C7 H15 N O4 S; 0.641	98.94	-2.35	C7 H15 N O4 S	0.641	209.0722	209.0717

Obs. *m/z*	Obs. RT	Obs. Mass	Tgt Formula	Tgt Mass	Tgt Mass Error (ppm)	RT Diff.	Find Cpds Algorithm
210.0789	0.641	209.0717	C7 H15 N O4 S	209.0722	-2.35	Find By Formula	

Compound Chromatograms

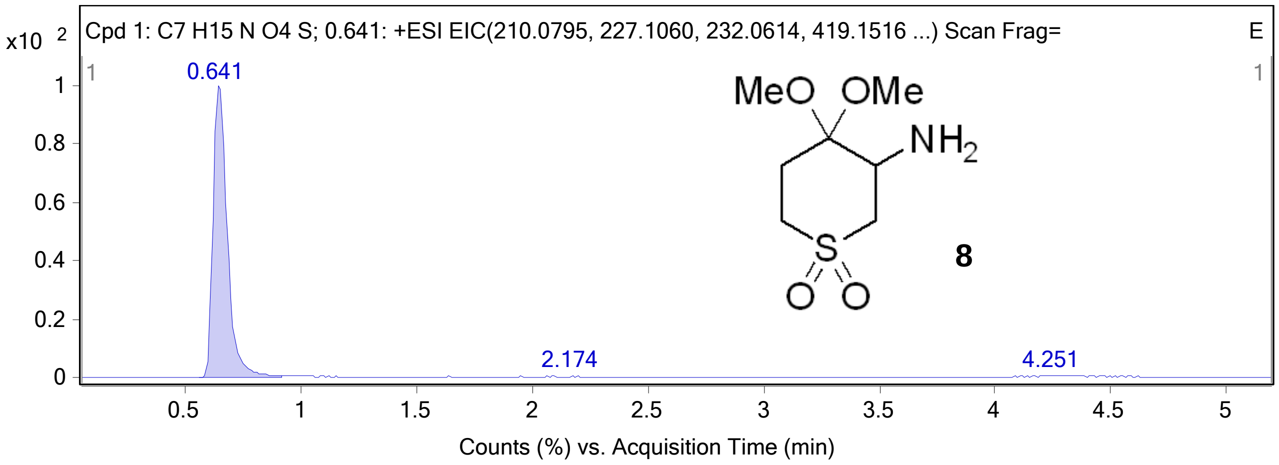

MS Zoomed Spectrum

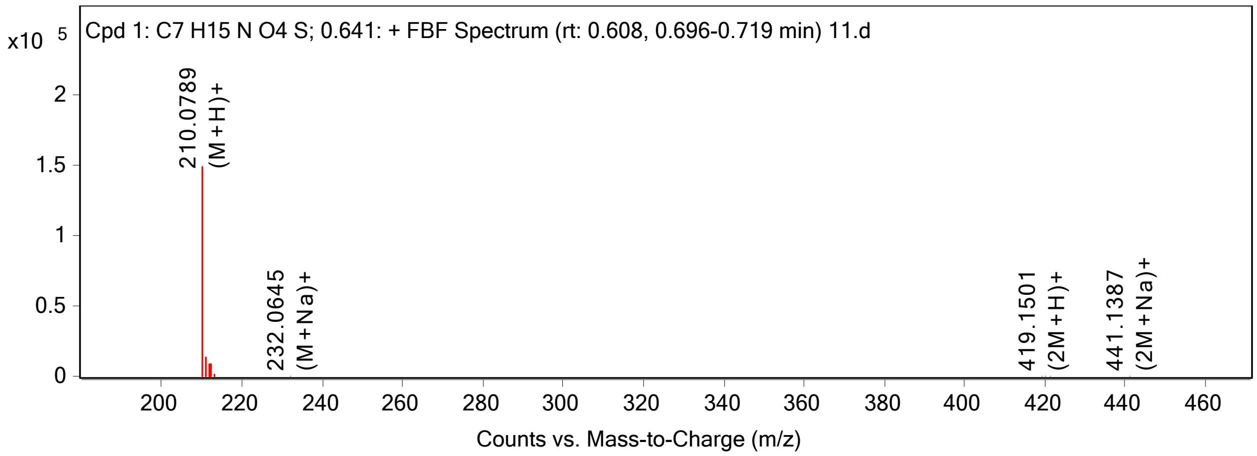

MS Spectrum Peak List

Obs. *m/z*	Charge	Abund	Ion/Isotope
210.0789	1	148792.86	(M+H)+
211.082	1	12206.11	(M+H)+
212.0765	1	7408.76	(M+H)+
213.0809	1	571.85	(M+H)+
232.0645	1	137.27	(M+Na)+
419.1501	1	448.79	(2M+H)+
420.1516	1	107.39	(2M+H)+
421.1487	1	83.17	(2M+H)+
441.1387	1	90.51	(2M+Na)+

MS Zoomed Spectrum

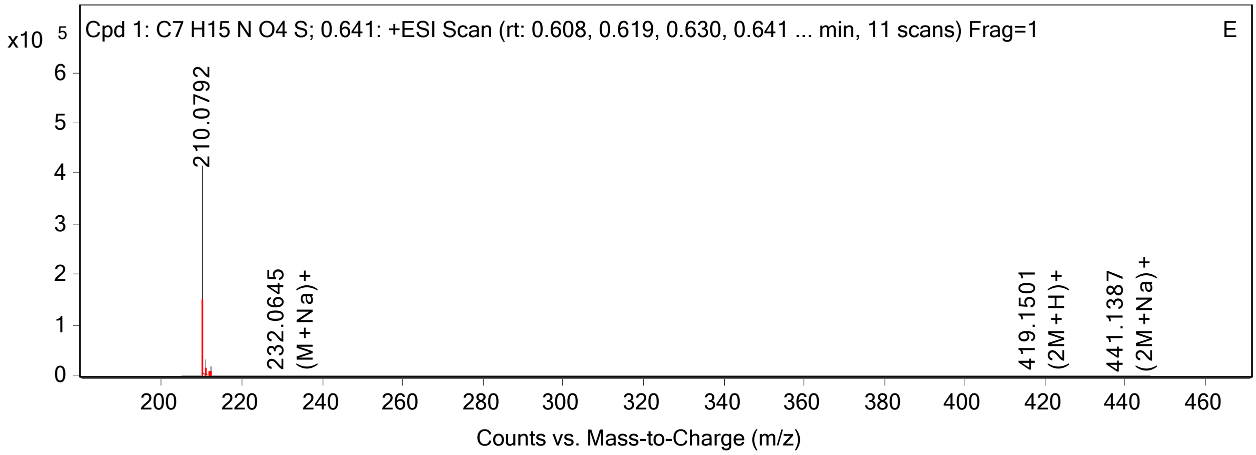

MS Spectrum Peak List

Obs. *m/z*	Charge	Abund	Ion/Isotope	Tgt Mass Error (ppm)
210.0789	1	148792.85	(M+H)+	2.41
210.0792		428030.08		
211.082	1	12206.11	(M+H)+	1.52
212.0765	1	7408.76	(M+H)+	2.88
213.0809	1	571.85	(M+H)+	-4.53
232.0645	1	137.27	(M+Na)+	-13.21
419.1501	1	448.79	(2M+H)+	3.66
420.1516	1	107.39	(2M+H)+	6.82
421.1487	1	83.17	(2M+H)+	2.58
441.1387	1	90.51	(2M+Na)+	-11.58

--- End Of Report ---

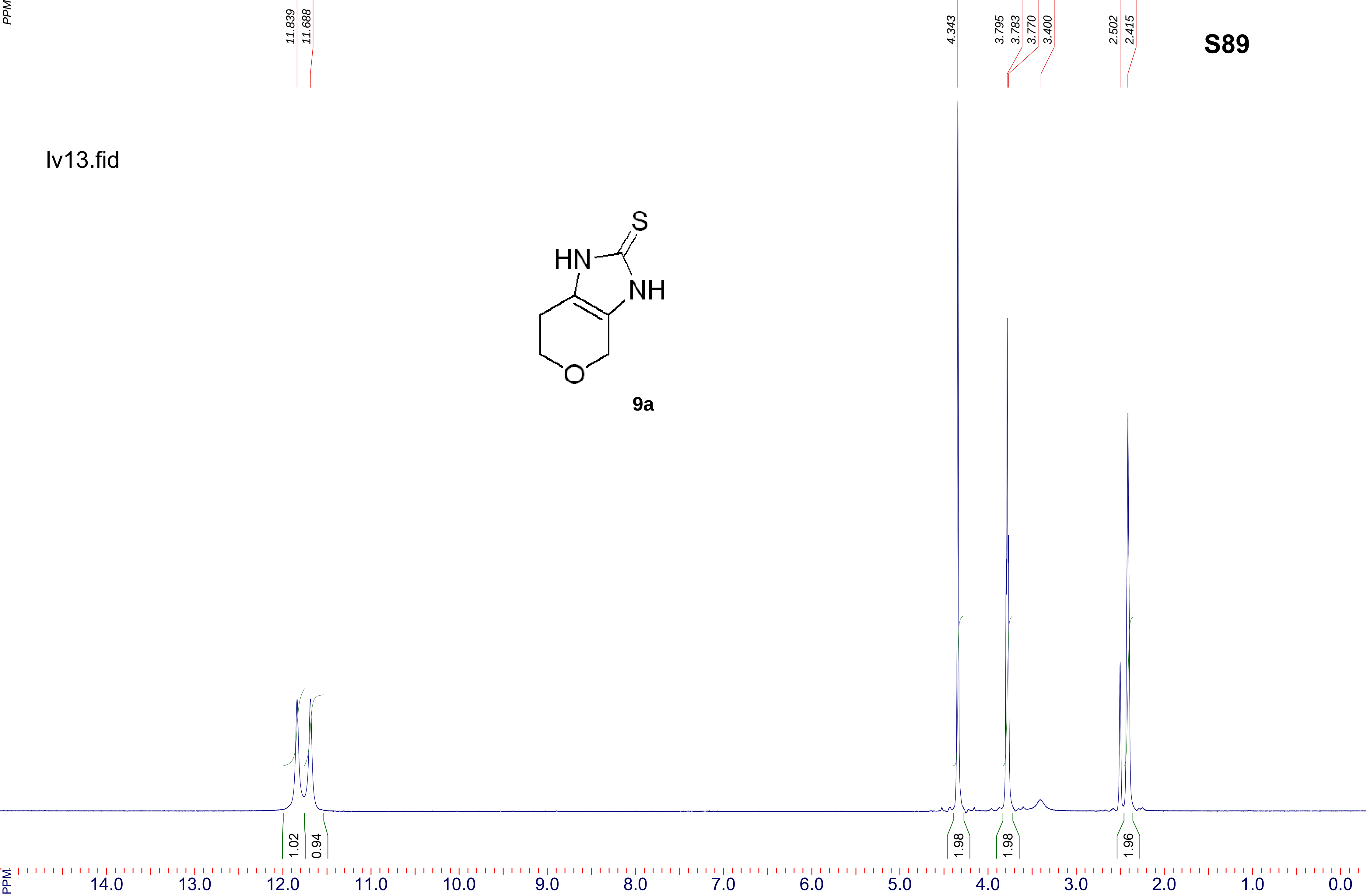

File name: lv13.fid	Operator:	SF: 399.9733 MHz	NSC: 0	PW: 10.90 usec, RG: 24	SI: 32768
Date: 10-Mar-2023	Solvent: dmso	SW: 8000 Hz	TE: 298 K	AQ: 2.00 sec, RD: 0.00 sec	

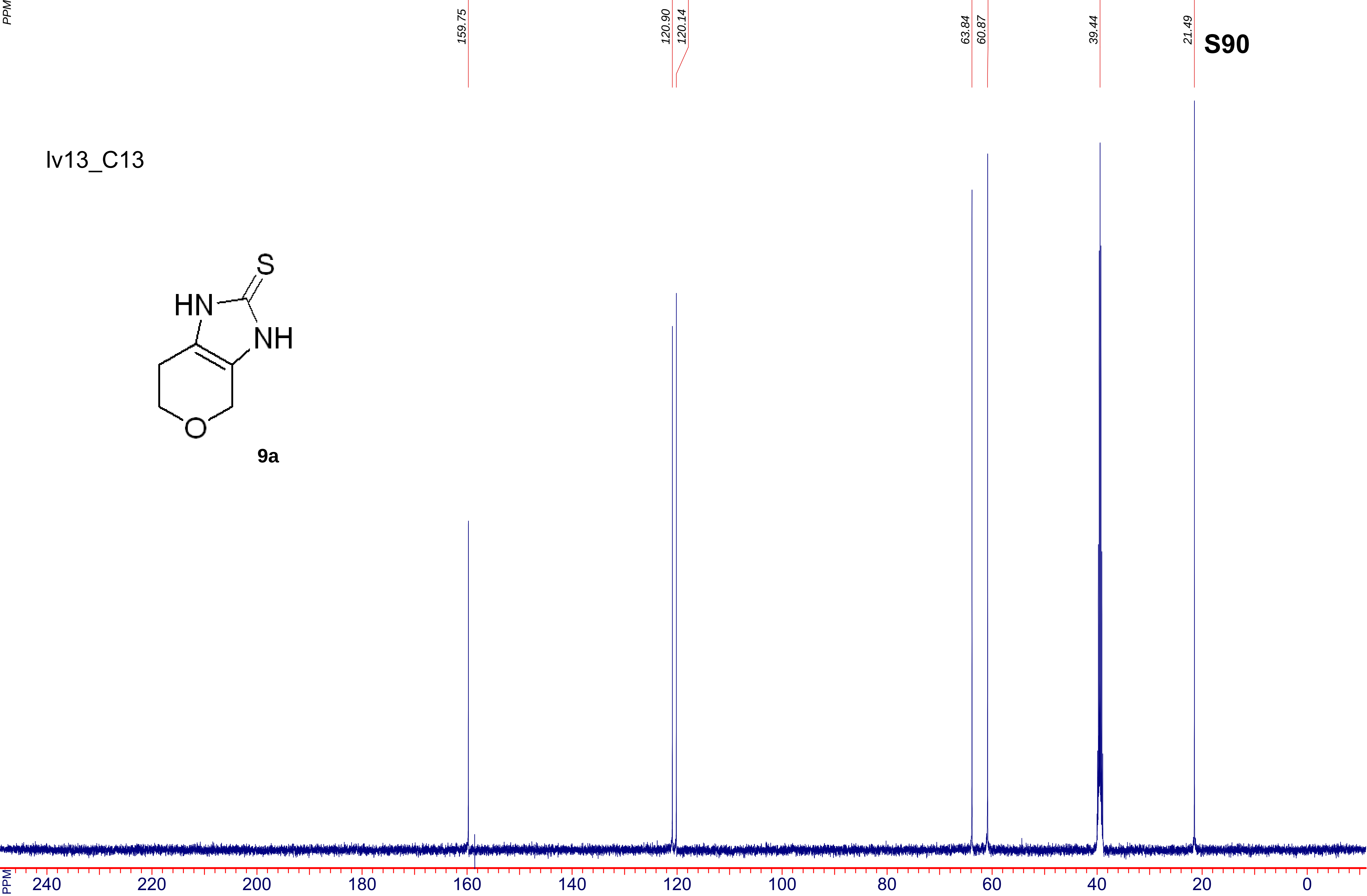

**File name: lv13_C13**	Operator: root	SF: 125.6429 MHz	NSC: 404	PW: 0.00 usec, RG: 51200	SI: 131072
Date: 12-Mar-2023	Solvent: DMSO	SW: 32680 Hz	TE: 683 K	AQ: 0.78 sec, RD: 0.00 sec	

Target Compound Screening Report

**Data File**	18c.d	**Sample Name**	13
**Sample Type**	Sample	**Position**	P1-B9
**Instrument Name**	Instrument 1	**User Name**	Denis V.Bylina
**Acq Method**	Fast_Gradient_HRMS_pos_Lock_01312023.m	**Acquired Time**	4/7/2023 4:27:14 PM (UTC+03:00)
**IRM Calibration Status**	Success	**DA Method**	1.m
**Comment**	Lysenko		
**Sample Group**		**Info.**	Agilent 6224 TOF LC/MS
**MFC**	C6H8N2OS	**Stream Name**	LC 1
**Acquisition Time (Local)**	4/7/2023 4:27:14 PM (UTC+03:00)	**Acquisition SW Version**	6200 series TOF/6500 series Q-TOF B.08.00 (B8058.0)
**TOF Driver Version**	8.00.00	**TOF Firmware Version**	8.643
**Tune Mass Range Max.**	1700		

Compound Table

Label	Tgt Score	Mass Error (ppm)	Tgt Formula	Obs. RT	Ref. Mass	Obs. Mass
Cpd 2: C6 H8 N2 O S; 2.257	94.85	-2.43	C6 H8 N2 O S	2.257	156.0357	156.0354

Obs. *m/z*	Obs. RT	Obs. Mass	Tgt Formula	Tgt Mass	Tgt Mass Error (ppm)	RT Diff.	Find Cpds Algorithm
179.0247	2.257	156.0354	C6 H8 N2 O S	156.0357	-2.43	Find By Formula	

Compound Chromatograms

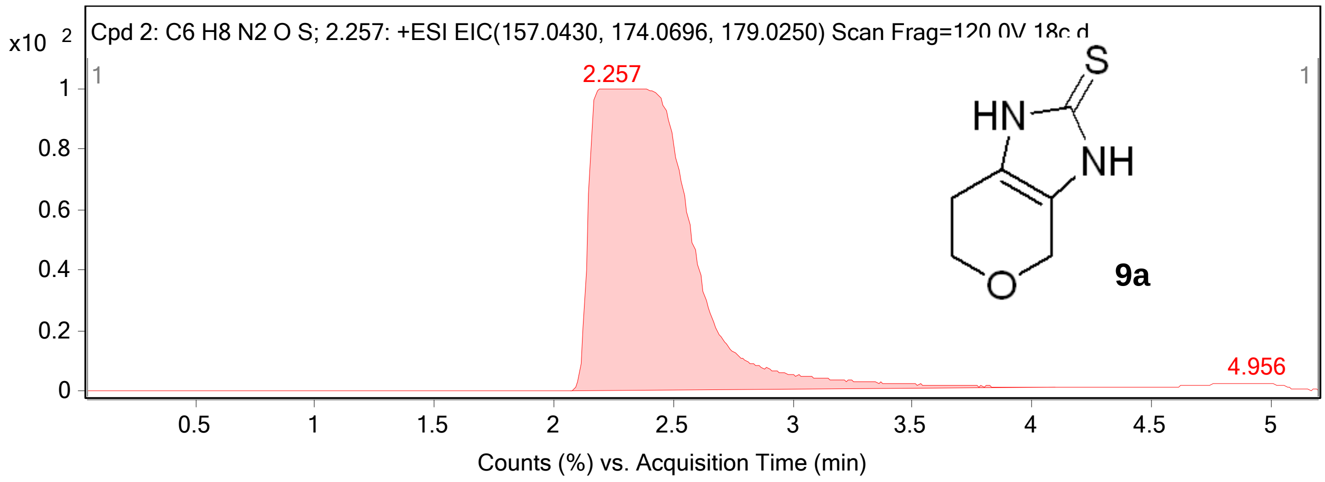

MS Zoomed Spectrum

Target Compound Screening Report

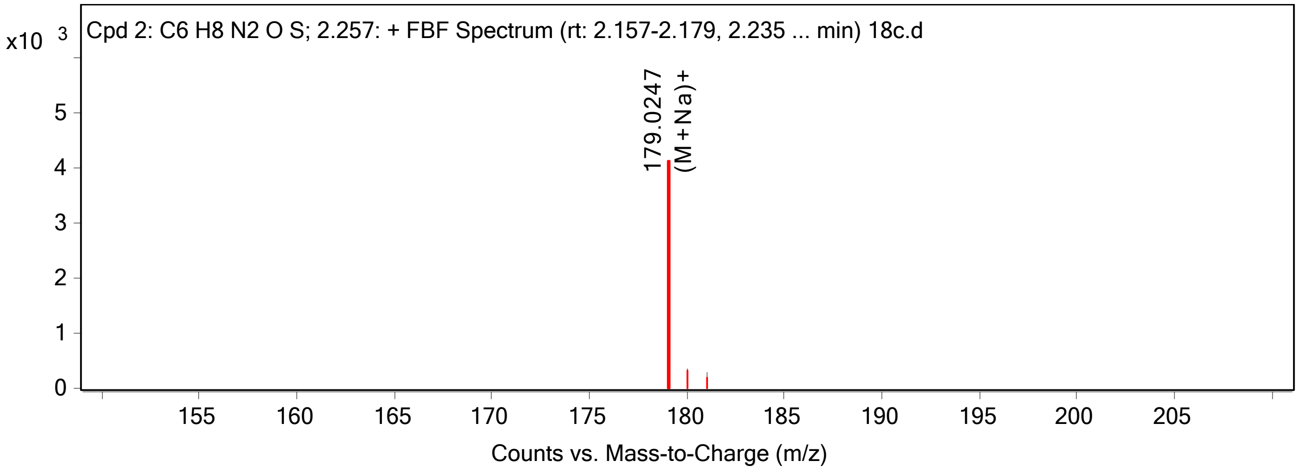

MS Spectrum Peak List

Obs. *m/z*	Charge	Abund	Ion/Isotope
179.0247	1	4013.44	(M+Na)+
180.0267	1	371.83	(M+Na)+
181.0194	1	289.9	(M+Na)+

MS Zoomed Spectrum

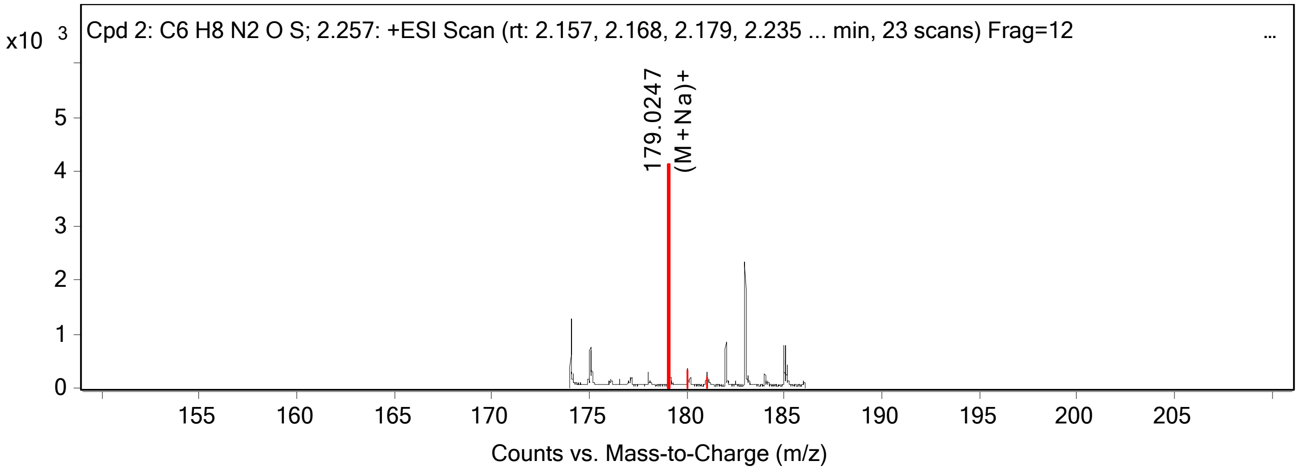

MS Spectrum Peak List

Obs. *m/z*	Charge	Abund	Ion/Isotope	Tgt Mass Error (ppm)
179.0247	1	4013.44	(M+Na)+	1.21
179.0248		4013.44		
180.0267	1	371.83	(M+Na)+	3.78
181.0194	1	289.9	(M+Na)+	12.33

--- End Of Report ---

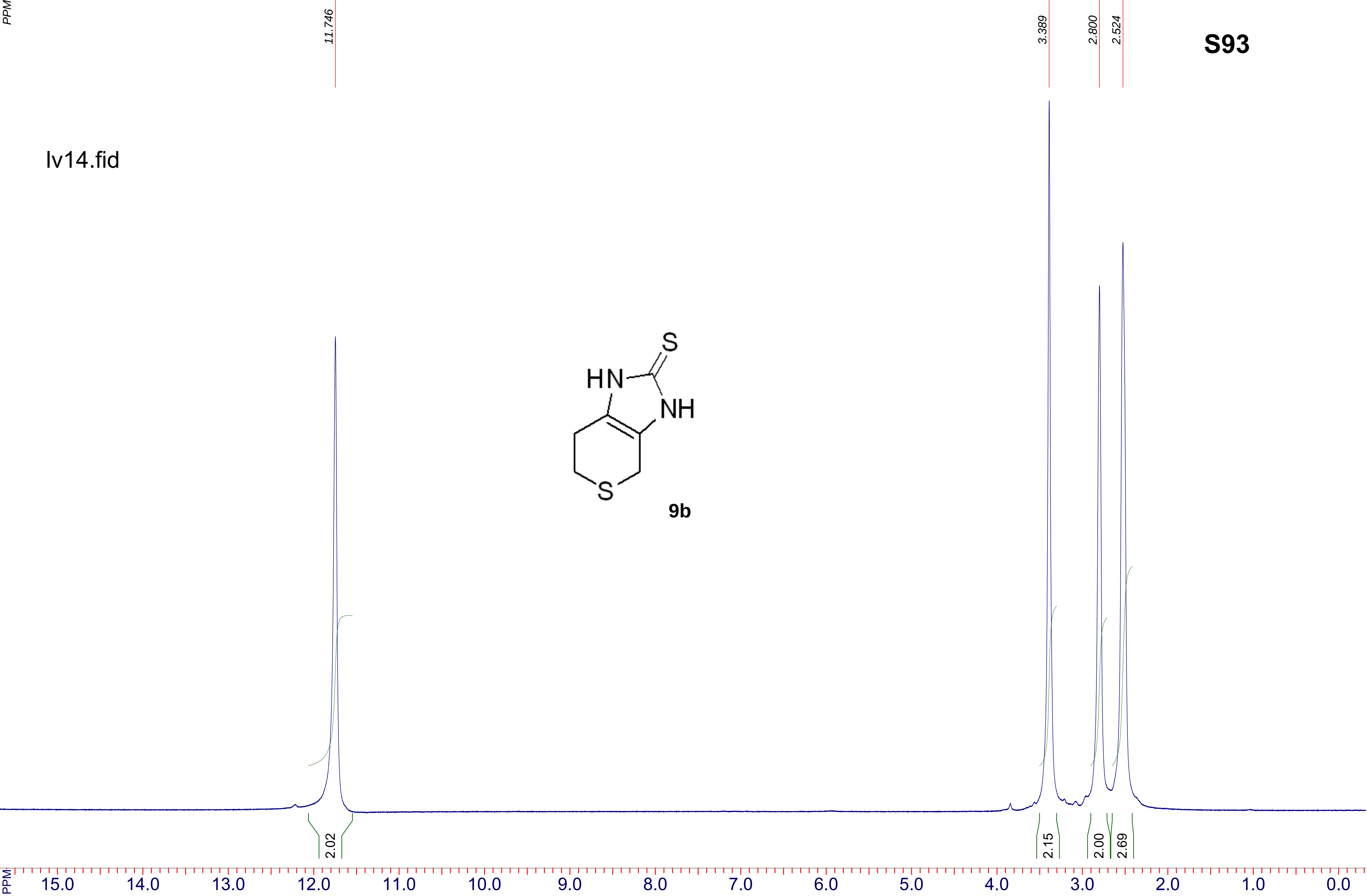

**File name: lv14.fid**	Operator:	SF: 399.9733 MHz	NSC: 0	PW: 10.90 usec, RG: 24	SI: 32768
Date: 09-Mar-2023	Solvent: dmso	SW: 8000 Hz	TE: 298 K	AQ: 2.00 sec, RD: 0.00 sec	

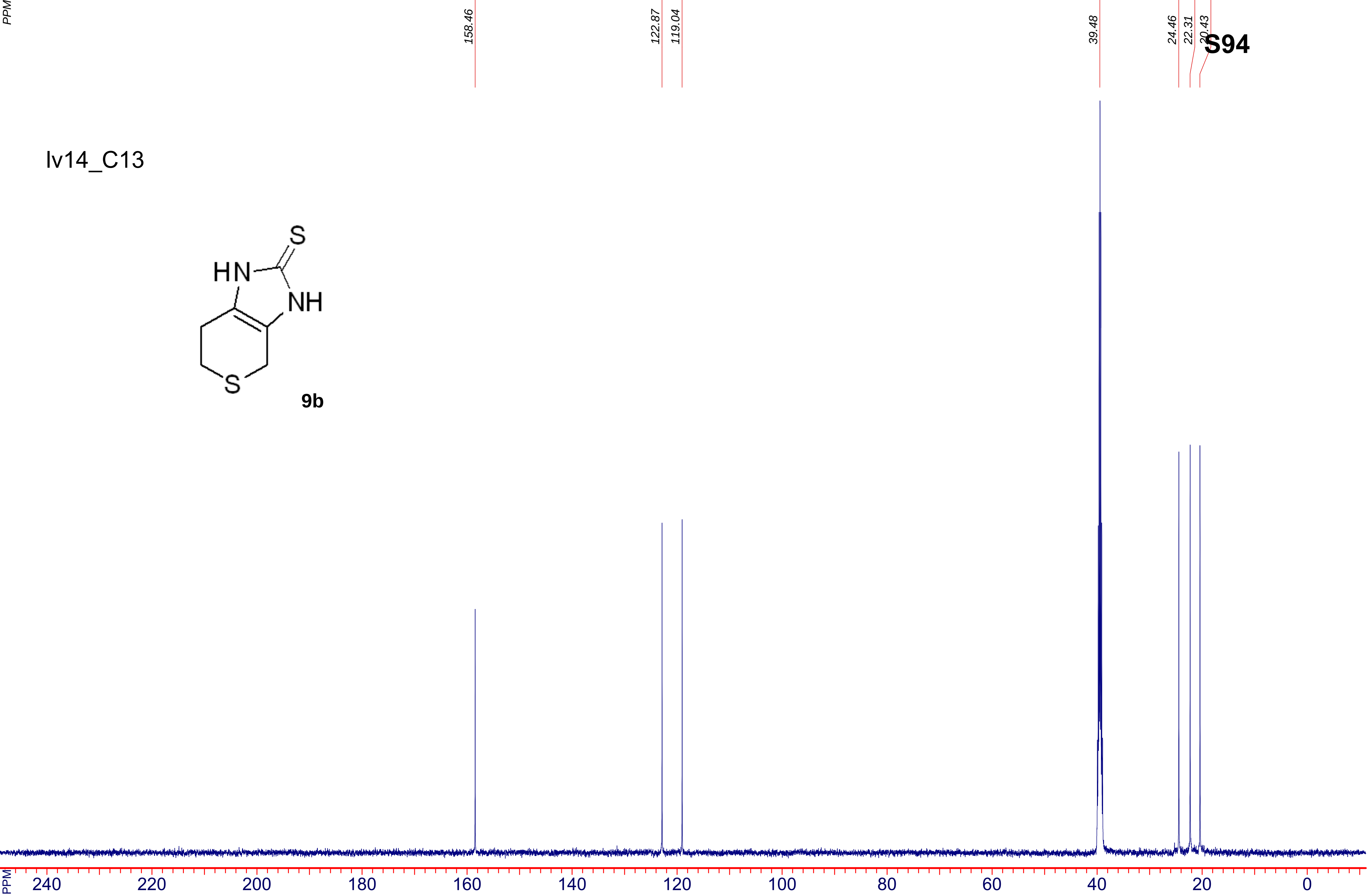

9b

File name: lv14_C13	Operator: root	SF: 125.6429 MHz	NSC: 350	PW: 0.00 usec, RG: 51200	SI: 131072
Date: 11-Mar-2023	Solvent: DMSO	SW: 32680 Hz	TE: 683 K	AQ: 1.00 sec, RD: 0.00 sec	

Target Compound Screening Report

**Data File**	19c.d	**Sample Name**	14
**Sample Type**	Sample	**Position**	P1-C1
**Instrument Name**	Instrument 1	**User Name**	Denis V.Bylina
**Acq Method**	Fast_Gradient_HRMS_pos_Lock_01312023.m	**Acquired Time**	4/7/2023 4:33:10 PM (UTC+03:00)
**IRM Calibration Status**	Success	**DA Method**	1.m
**Comment**	Lysenko		
**Sample Group**		**Info.**	Agilent 6224 TOF LC/MS
**MFC**	C6H8N2S2	**Stream Name**	LC 1
**Acquisition Time (Local)**	4/7/2023 4:33:10 PM (UTC+03:00)	**Acquisition SW Version**	6200 series TOF/6500 series Q-TOF B.08.00 (B8058.0)
**TOF Driver Version**	8.00.00	**TOF Firmware Version**	8.643
**Tune Mass Range Max.**	1700		

Compound Table

Label	Tgt Score	Mass Error (ppm)	Tgt Formula	Obs. RT	Ref. Mass	Obs. Mass
Cpd 3: C6 H8 N2 S2; 2.811	98.76	-1	C6 H8 N2 S2	2.811	172.0129	172.0127

Obs. *m/z*	Obs. RT	Obs. Mass	Tgt Formula	Tgt Mass	Tgt Mass Error (ppm)	RT Diff.	Find Cpds Algorithm
173.02	2.811	172.0127	C6 H8 N2 S2	172.0129	-1	Find By Formula	

Compound Chromatograms

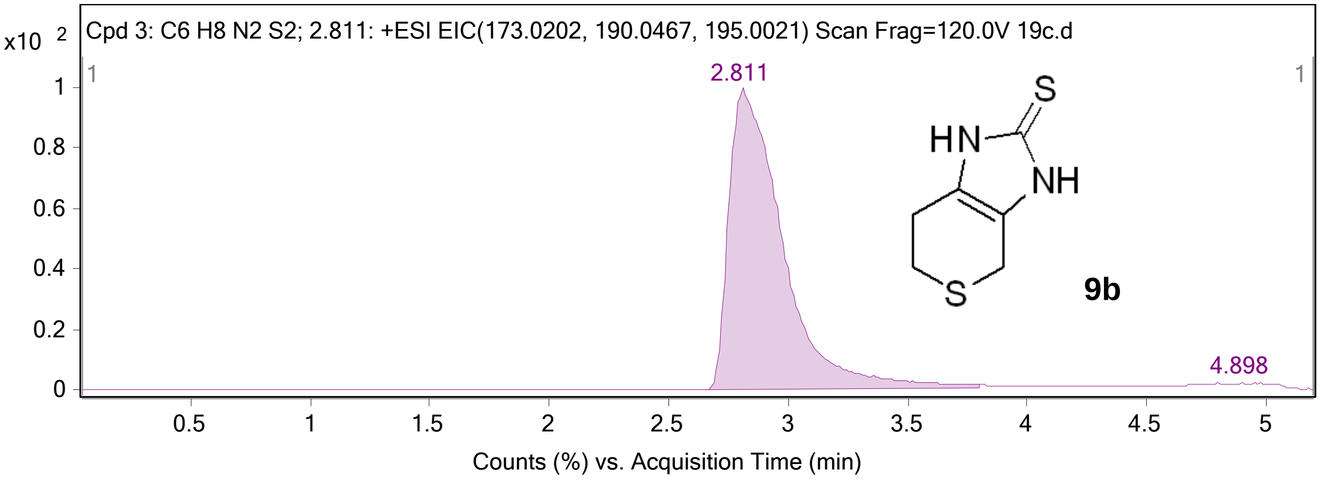

MS Zoomed Spectrum

Target Compound Screening Report

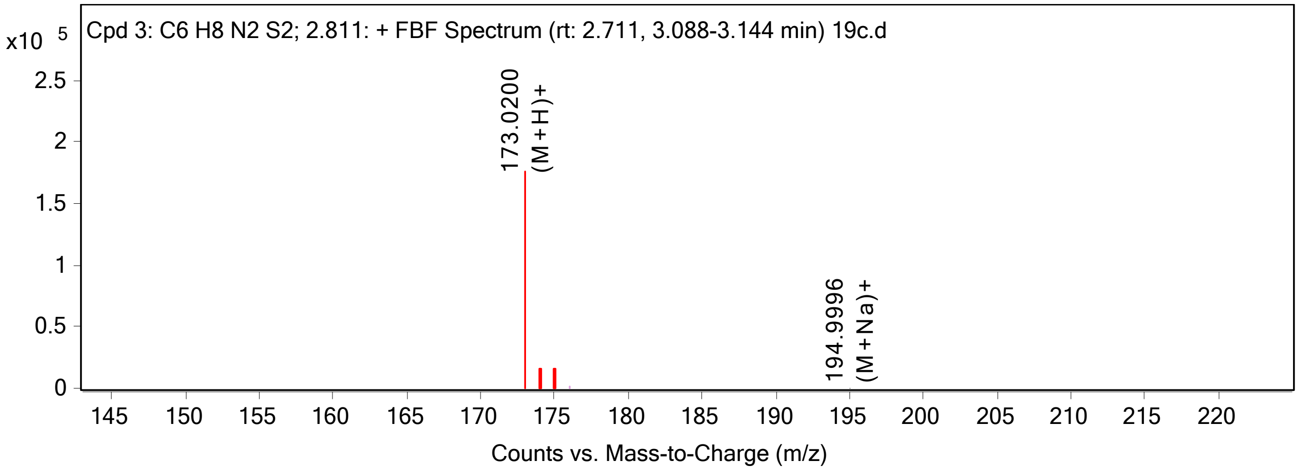

MS Spectrum Peak List

Obs. *m/z*	Charge	Abund	Ion/Isotope
173.02	1	175769.8	(M+H)+
174.0219	1	13750.35	(M+H)+
175.016	1	13882.02	(M+H)+
194.9996	1	249.51	(M+Na)+

MS Zoomed Spectrum

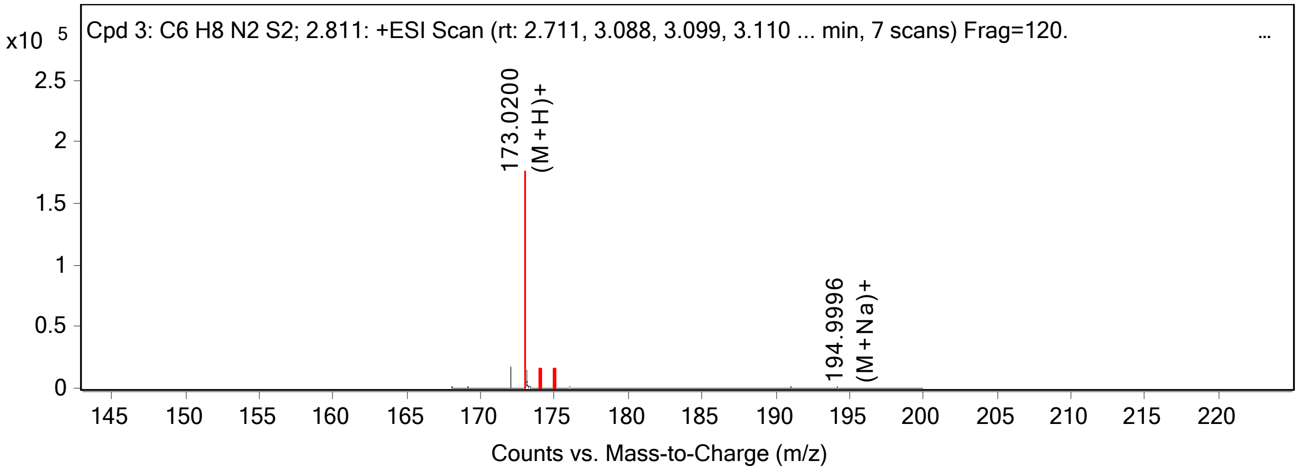

MS Spectrum Peak List

Obs. *m/z*	Charge	Abund	Ion/Isotope	Tgt Mass Error (ppm)
173.02	1	175769.8	(M+H)+	0.82
173.02		175769.8		
174.0219	1	13750.35	(M+H)+	2.48
175.016	1	13882.02	(M+H)+	1.5
194.9996	1	249.51	(M+Na)+	12.71

--- End Of Report ---

lv16.fid

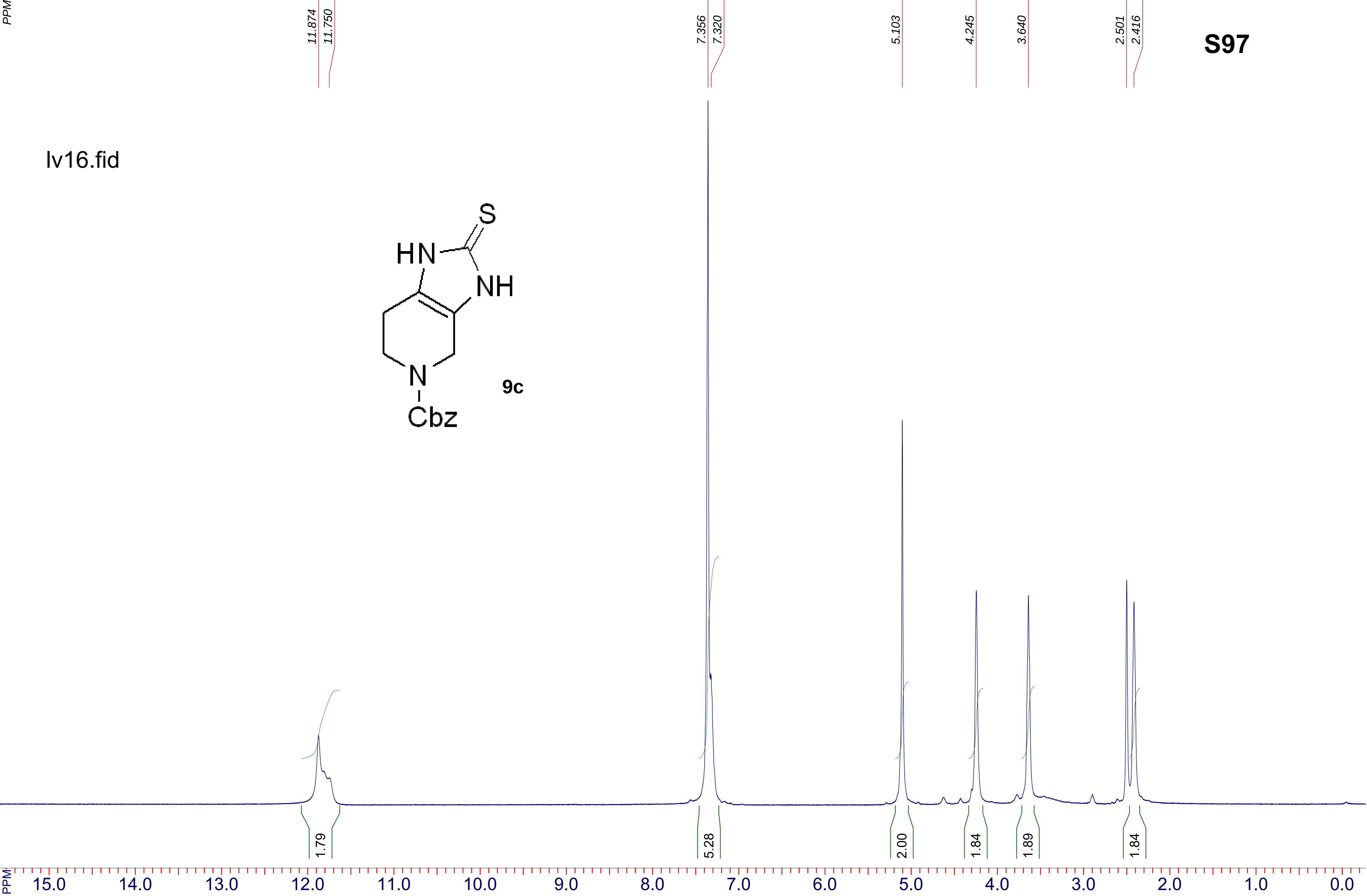

9c

**File name: lv16.fid**	Operator:	SF: 399.9733 MHz	NSC: 0	PW: 10.90 usec, RG: 24	SI: 32768
Date: 09-Mar-2023	Solvent: dmso	SW: 8000 Hz	TE: 298 K	AQ: 2.00 sec, RD: 0.00 sec	

S98

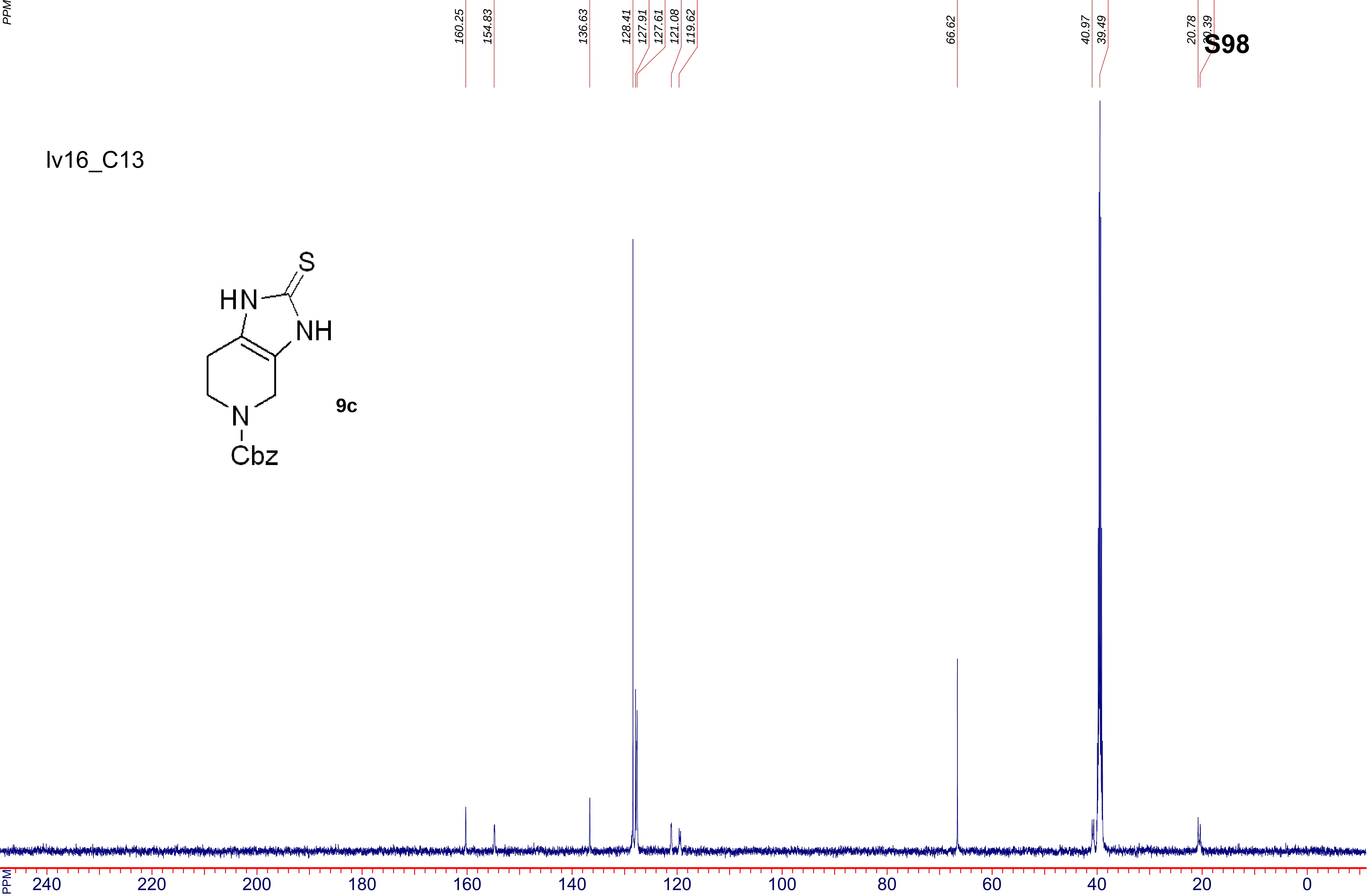

**File name: lv16_C13**	Operator: root	SF: 125.6429 MHz	NSC: 350	PW: 0.00 usec, RG: 51200	SI: 131072
Date: 11-Mar-2023	Solvent: DMSO	SW: 32680 Hz	TE: 683 K	AQ: 1.00 sec, RD: 0.00 sec	

Target Compound Screening Report

**Data File**	21c.d	**Sample Name**	16
**Sample Type**	Sample	**Position**	P1-C3
**Instrument Name**	Instrument 1	**User Name**	Denis V.Bylina
**Acq Method**	Fast_Gradient_HRMS_pos_Lock_01312023.m	**Acquired Time**	4/7/2023 4:45:05 PM (UTC+03:00)
**IRM Calibration Status**	Success	**DA Method**	1.m
**Comment**	Lysenko		
**Sample Group**		**Info.**	Agilent 6224 TOF LC/MS
**MFC**	C14H15N3O2S	**Stream Name**	LC 1
**Acquisition Time (Local)**	4/7/2023 4:45:05 PM (UTC+03:00)	**Acquisition SW Version**	6200 series TOF/6500 series Q-TOF B.08.00 (B8058.0)
**TOF Driver Version**	8.00.00	**TOF Firmware Version**	8.643
**Tune Mass Range Max.**	1700		

Compound Table

Label	Tgt Score	Mass Error (ppm)	Tgt Formula	Obs. RT	Ref. Mass	Obs. Mass
Cpd 3: C14 H15 N3 O2 S; 3.403	98.86	-0.44	C14 H15 N3 O2 S	3.403	289.0885	289.0884

Obs. *m/z*	Obs. RT	Obs. Mass	Tgt Formula	Tgt Mass	Tgt Mass Error (ppm)	RT Diff.	Find Cpds Algorithm
290.0957	3.403	289.0884	C14 H15 N3 O2 S	289.0885	-0.44	Find By Formula	

Compound Chromatograms

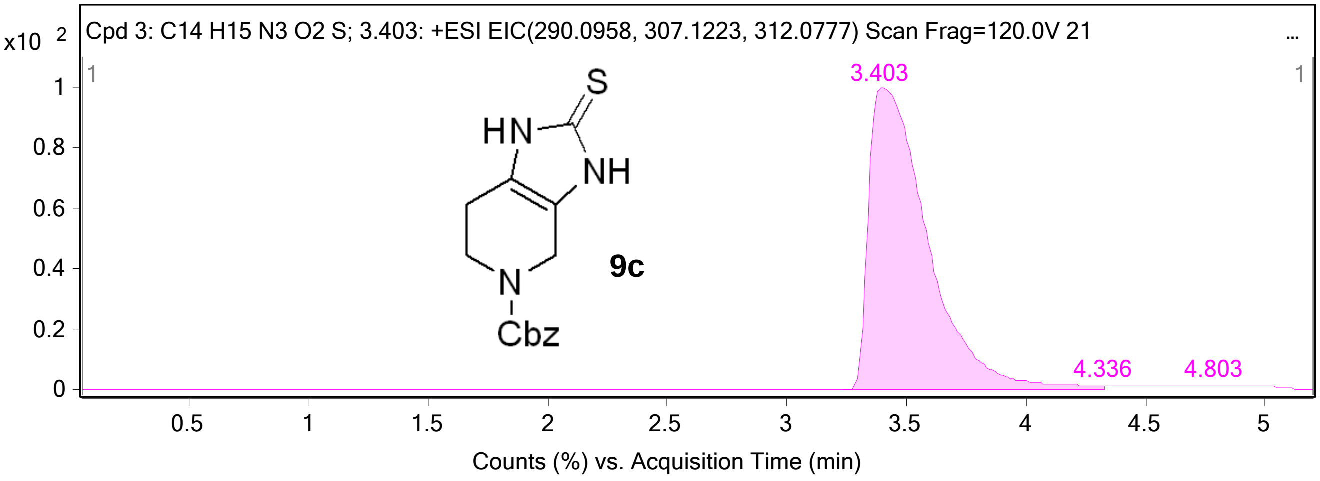

MS Zoomed Spectrum

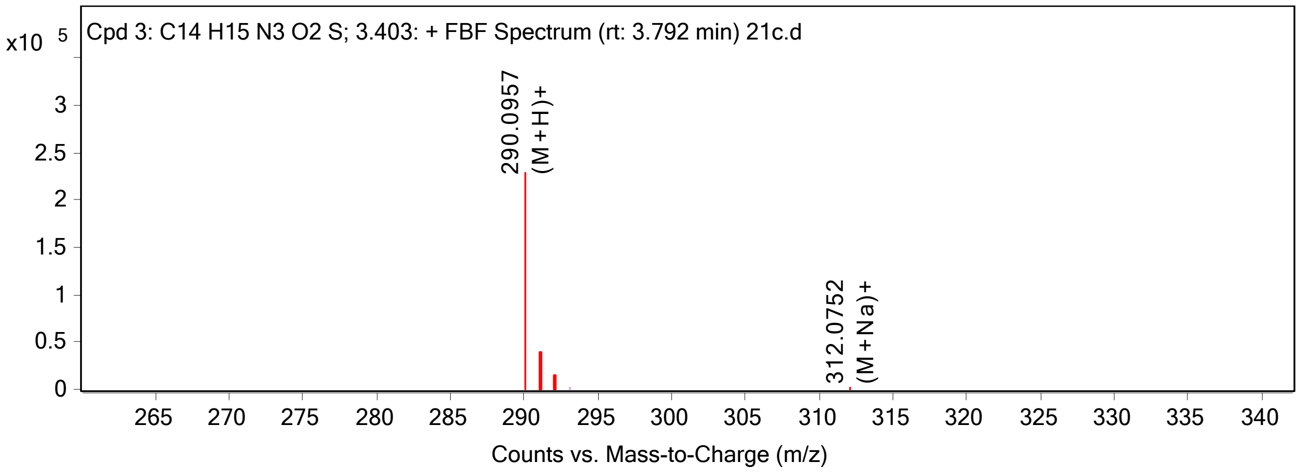

MS Spectrum Peak List

Obs. *m/z*	Charge	Abund	Ion/Isotope
290.0957	1	228762.97	(M+H)+
291.0984	1	37495.89	(M+H)+
292.0932	1	11089.01	(M+H)+
312.0752	1	1624.41	(M+Na)+

MS Zoomed Spectrum

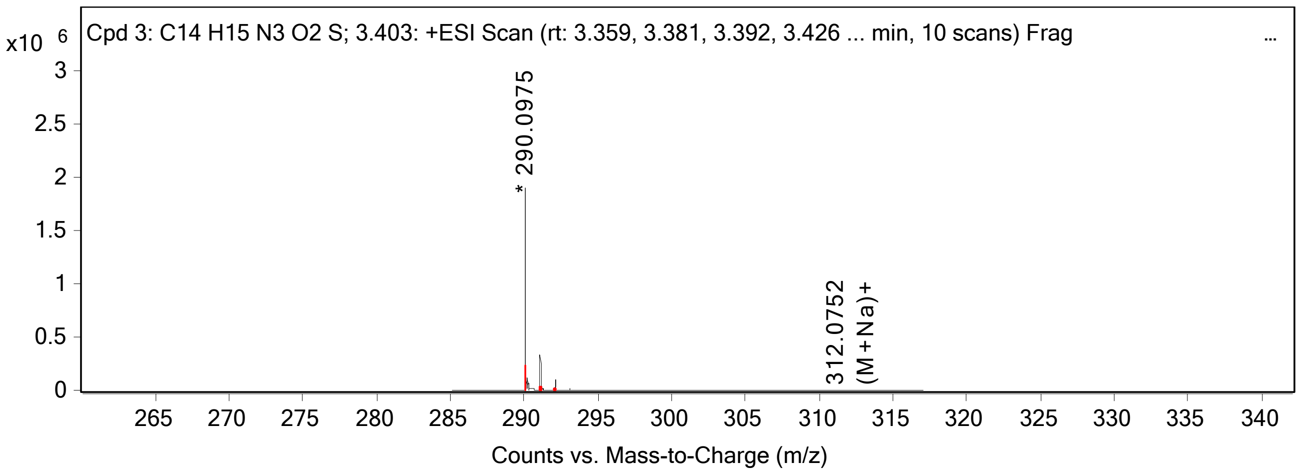

MS Spectrum Peak List

Obs. *m/z*	Charge	Abund	Ion/Isotope	Tgt Mass Error (ppm)
290.0957	1	228762.96	(M+H)+	0.19
290.0975		1924372.75		
291.0984	1	37495.9	(M+H)+	0.65
292.0932	1	11089.01	(M+H)+	3.68
312.0752	1	1624.41	(M+Na)+	8.18

--- End Of Report ---

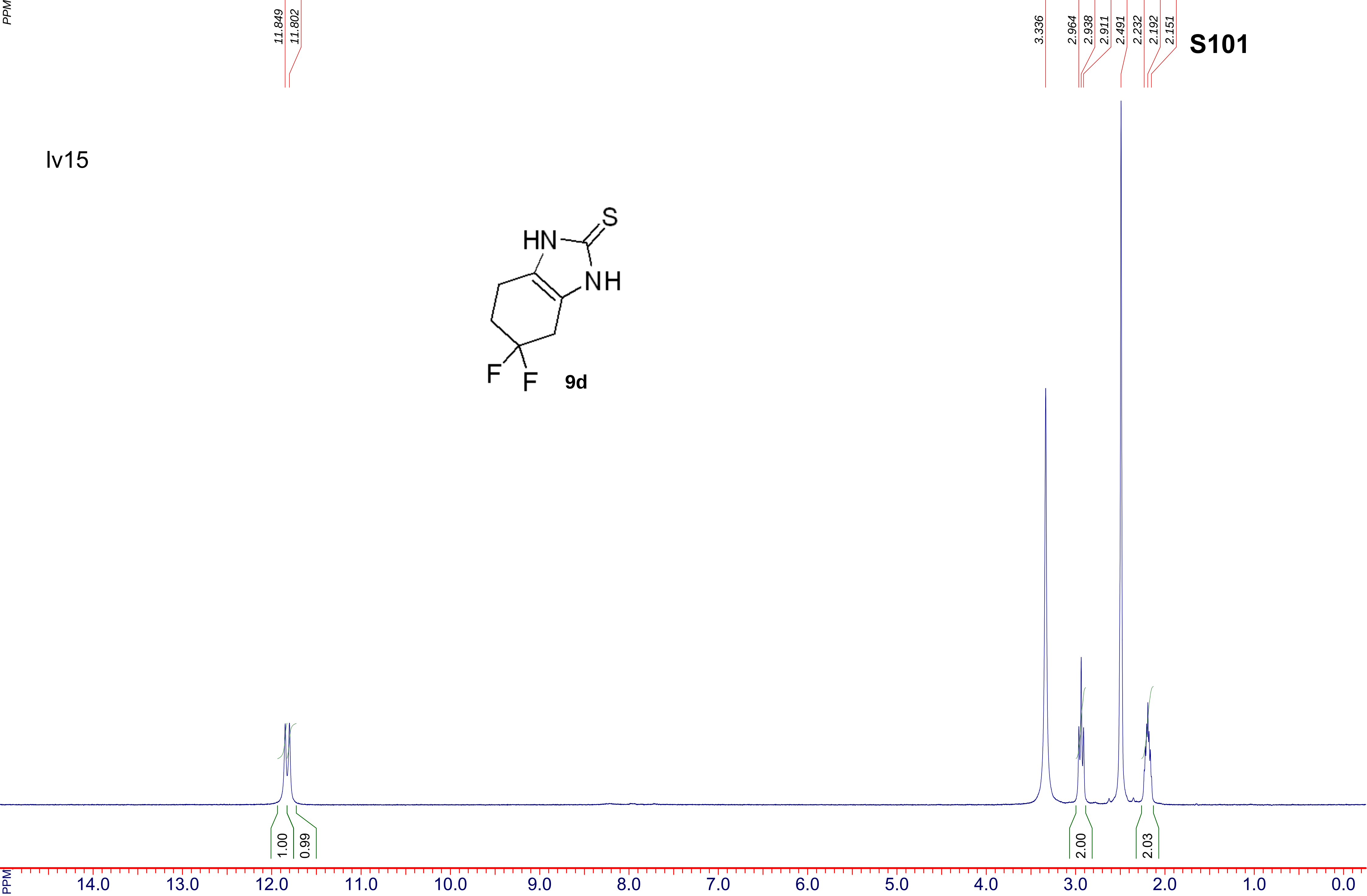

**File name: lv15**	Operator: root	SF: 499.6730 MHz	NSC: 1	PW: 0.00 usec, RG: 32	SI: 32768
Date: 23-Mar-2023	Solvent: DMSO	SW: 8993 Hz	TE: 683 K	AQ: 1.82 sec, RD: 0.00 sec	

lv15_C13

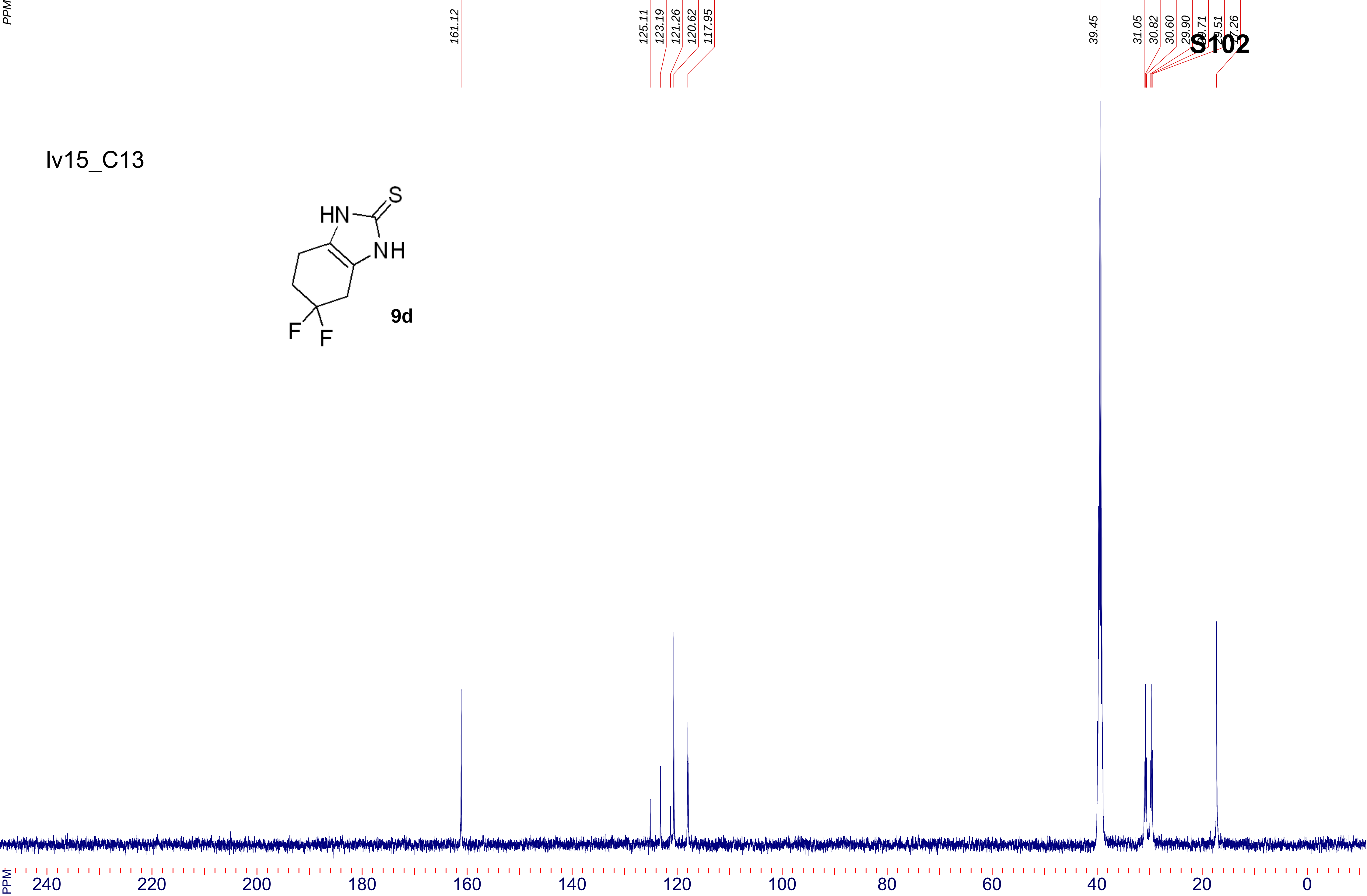

File name: lv15_C13	Operator: root	SF: 125.6429 MHz	NSC: 350	PW: 0.00 usec, RG: 51200	SI: 131072
Date: 11-Mar-2023	Solvent: DMSO	SW: 32680 Hz	TE: 683 K	AQ: 1.00 sec, RD: 0.00 sec	

BB500610-75_F19{H}

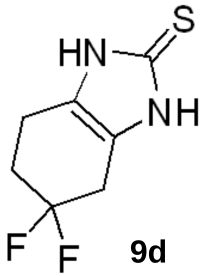

9d

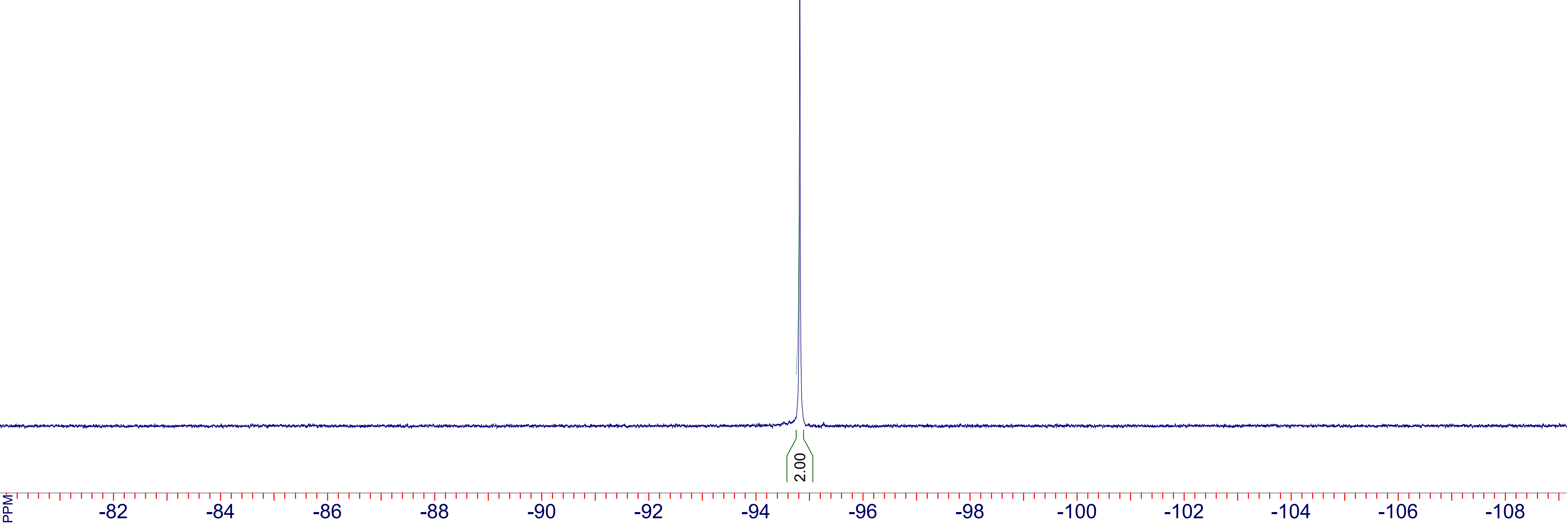

**File name: BB500610-75_F19{H}**	Operator: nmr	SF: 376.4986 MHz	NSC: 1	PW: 0.00 usec, RG: 456	SI: 262144
Date: 22-Jun-2023	Solvent: DMSO	SW: 138889 Hz	TE: 300 K	AQ: 0.94 sec, RD: 0.00 sec	

Target Compound Screening Report

**Data File**	20c.d	**Sample Name**	15
**Sample Type**	Sample	**Position**	P1-C2
**Instrument Name**	Instrument 1	**User Name**	Denis V.Bylina
**Acq Method**	Fast_Gradient_HRMS_pos_Lock_01312023.m	**Acquired Time**	4/7/2023 4:39:08 PM (UTC+03:00)
**IRM Calibration Status**	Success	**DA Method**	1.m
**Comment**	Lysenko		
**Sample Group**		**Info.**	Agilent 6224 TOF LC/MS
**MFC**	C7H8F2N2S	**Stream Name**	LC 1
**Acquisition Time (Local)**	4/7/2023 4:39:08 PM (UTC+03:00)	**Acquisition SW Version**	6200 series TOF/6500 series Q-TOF B.08.00 (B8058.0)
**TOF Driver Version**	8.00.00	**TOF Firmware Version**	8.643
**Tune Mass Range Max.**	1700		

Compound Table

Label	Tgt Score	Mass Error (ppm)	Tgt Formula	Obs. RT	Ref. Mass	Obs. Mass
Cpd 2: C7 H8 F2 N2 S; 2.973	99.36	-0.89	C7 H8 F2 N2 S	2.973	190.0376	190.0375

Obs. *m/z*	Obs. RT	Obs. Mass	Tgt Formula	Tgt Mass	Tgt Mass Error (ppm)	RT Diff.	Find Cpds Algorithm
191.0447	2.973	190.0375	C7 H8 F2 N2 S	190.0376	-0.89	Find By Formula	

Compound Chromatograms

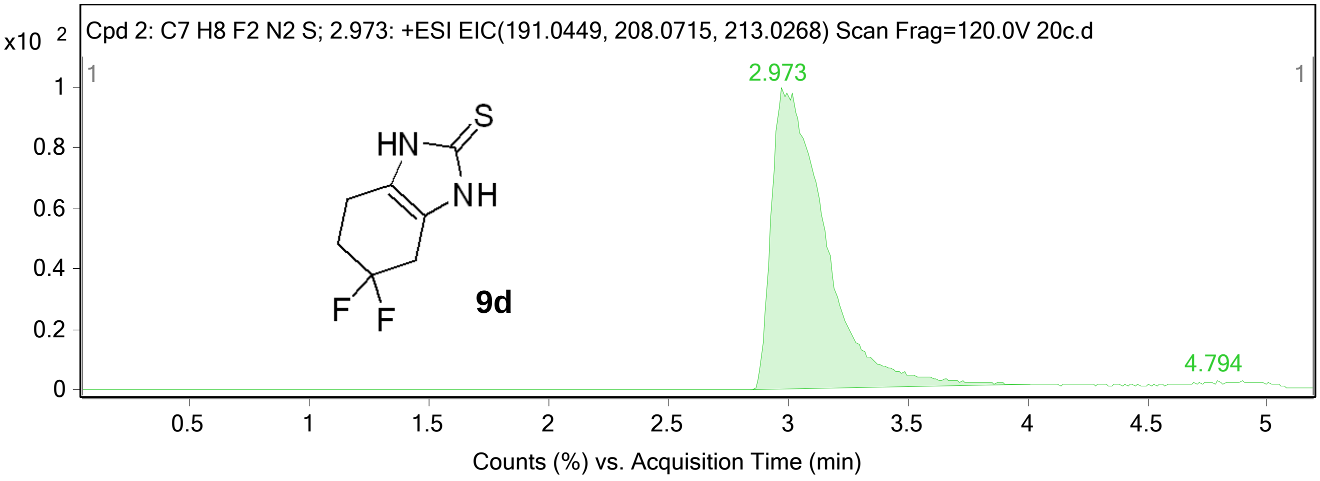

9d

MS Zoomed Spectrum

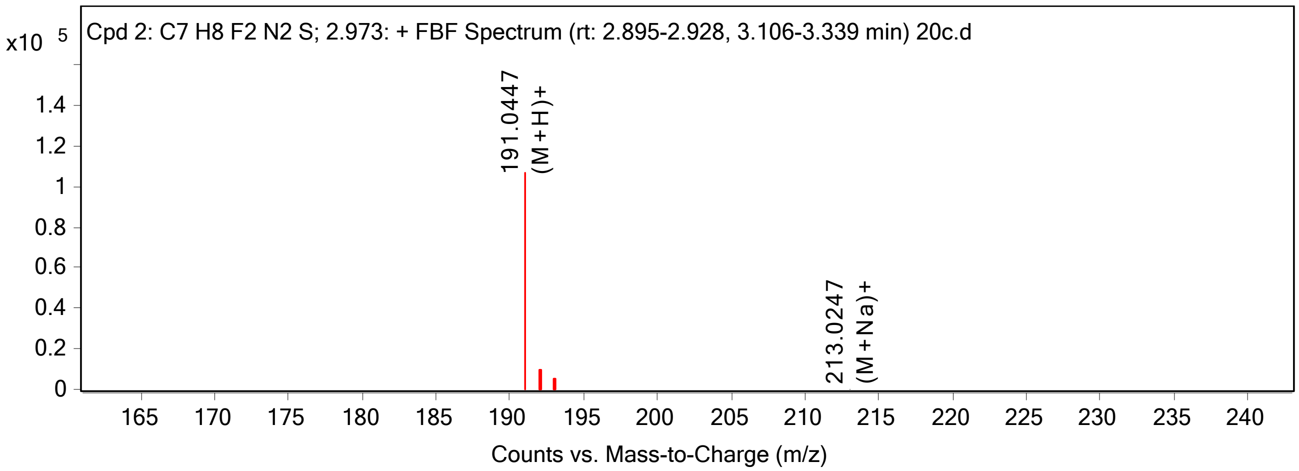

MS Spectrum Peak List

Obs. *m/z*	Charge	Abund	Ion/Isotope
191.0447	1	106640.52	(M+H)+
192.0473	1	8811.7	(M+H)+
193.0413	1	4331.7	(M+H)+
213.0247	1	192.85	(M+Na)+

MS Zoomed Spectrum

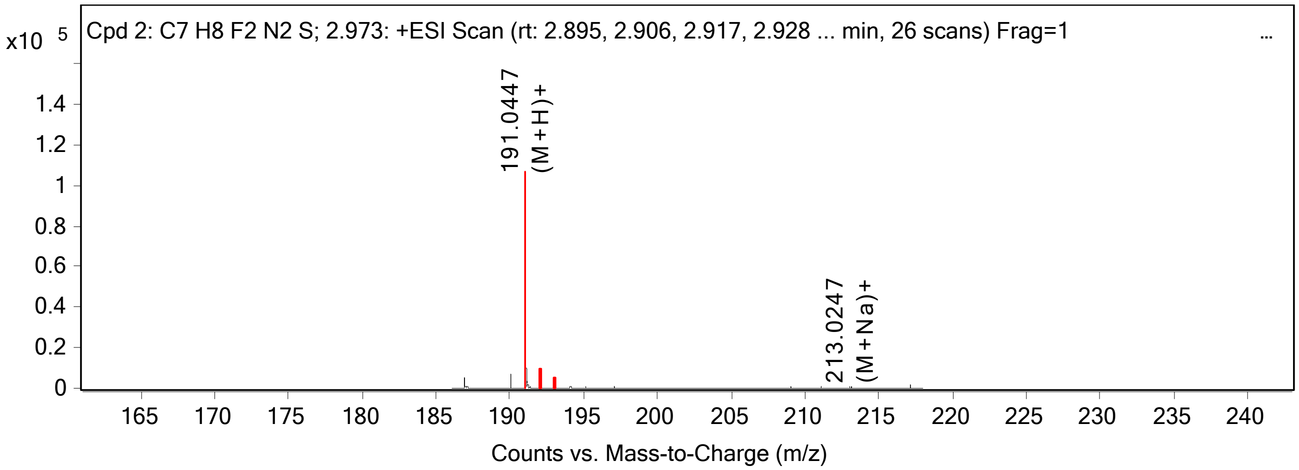

MS Spectrum Peak List

Obs. *m/z*	Charge	Abund	Ion/Isotope	Tgt Mass Error (ppm)
191.0447	1	106640.51	(M+H)+	0.89
191.0447		106640.51		
192.0473	1	8811.7	(M+H)+	0.89
193.0413	1	4331.7	(M+H)+	0.42
213.0247	1	192.85	(M+Na)+	10.04

--- End Of Report ---

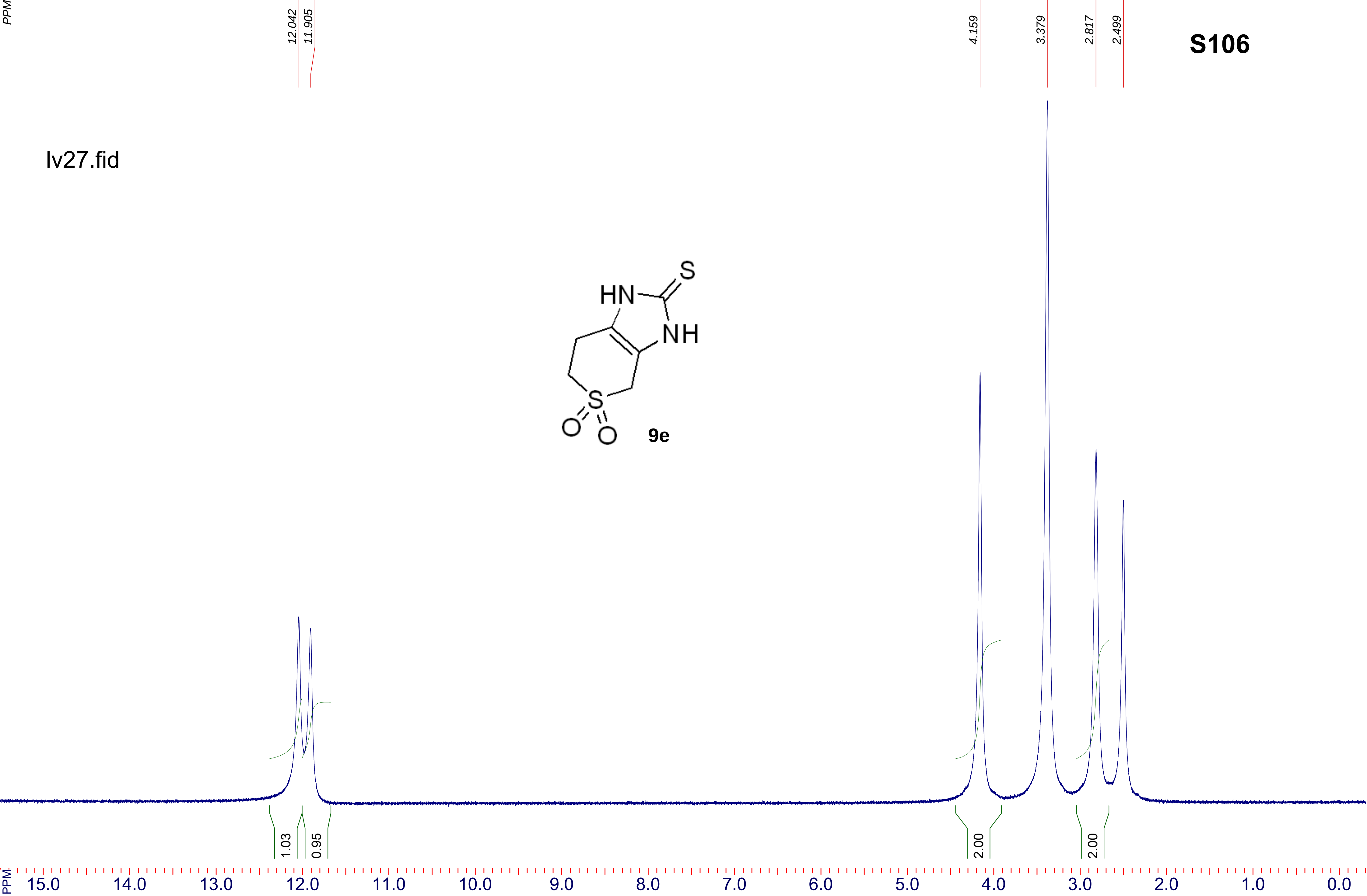

**File name: lv27.fid**	Operator:	SF: 399.9733 MHz	NSC: 0	PW: 10.90 usec, RG: 24	SI: 32768
Date: 23-Mar-2023	Solvent: dmso	SW: 8000 Hz	TE: 298 K	AQ: 2.00 sec, RD: 0.00 sec	

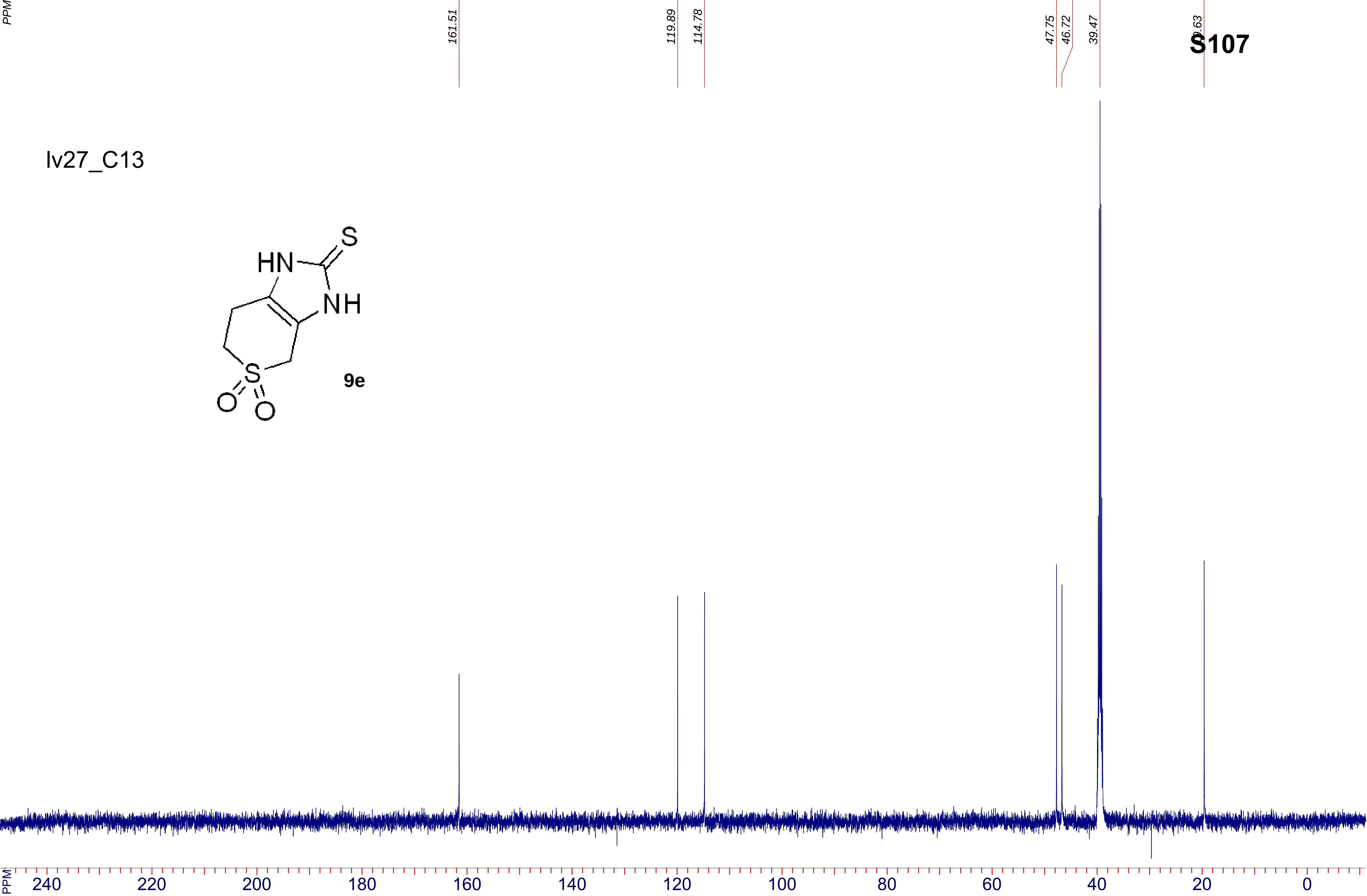

9e

**File name: lv27_C13**	Operator: root	SF: 125.6429 MHz	NSC: 146	PW: 0.00 usec, RG: 51200	SI: 131072
Date: 25-Mar-2023	Solvent: DMSO	SW: 32680 Hz	TE: 683 K	AQ: 1.00 sec, RD: 0.00 sec	

Target Compound Screening Report

**Data File**	27c.d	**Sample Name**	27
**Sample Type**	Sample	**Position**	P1-C9
**Instrument Name**	Instrument 1	**User Name**	Denis V.Bylina
**Acq Method**	Fast_Gradient_HRMS_pos_Lock_01312023.m	**Acquired Time**	4/7/2023 5:08:57 PM (UTC+03:00)
**IRM Calibration Status**	Success	**DA Method**	1.m
**Comment**	Lysenko		
**Sample Group**		**Info.**	Agilent 6224 TOF LC/MS
**MFC**	C6H8N2O2S2	**Stream Name**	LC 1
**Acquisition Time (Local)**	4/7/2023 5:08:57 PM (UTC+03:00)	**Acquisition SW Version**	6200 series TOF/6500 series Q-TOF B.08.00 (B8058.0)
**TOF Driver Version**	8.00.00	**TOF Firmware Version**	8.643
**Tune Mass Range Max.**	1700		

Compound Table

Label	Tgt Score	Mass Error (ppm)	Tgt Formula	Obs. RT	Ref. Mass	Obs. Mass
Cpd 1: C6 H8 N2 O2 S2; 1.249	99.42	-0.29	C6 H8 N2 O2 S2	1.249	204.0027	204.0027

Obs. *m/z*	Obs. RT	Obs. Mass	Tgt Formula	Tgt Mass	Tgt Mass Error (ppm)	RT Diff.	Find Cpds Algorithm
205.01	1.249	204.0027	C6 H8 N2 O2 S2	204.0027	-0.29	Find By Formula	

Compound Chromatograms

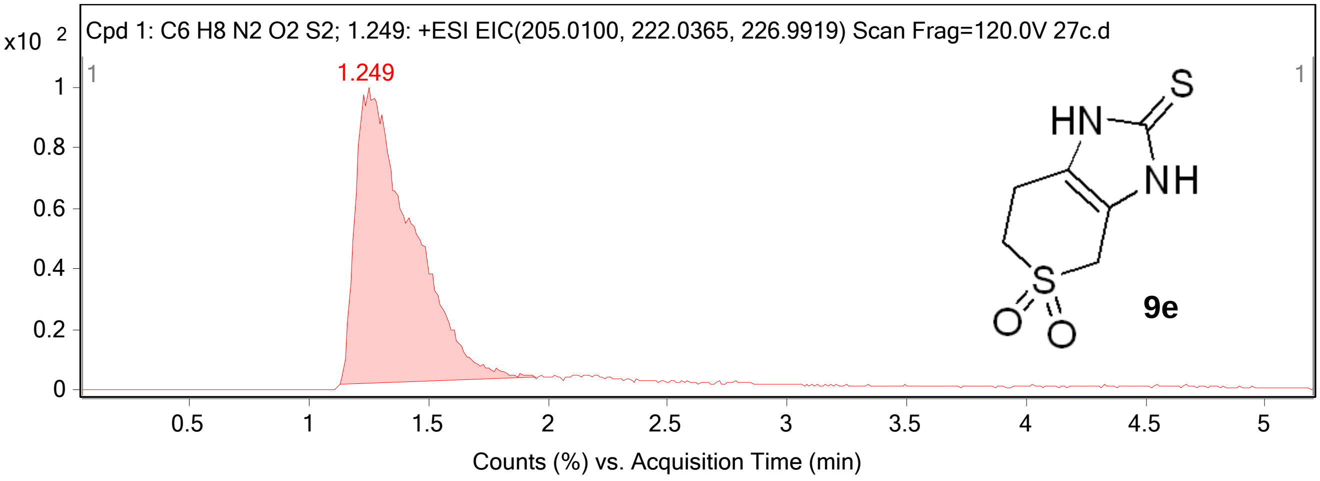

MS Zoomed Spectrum

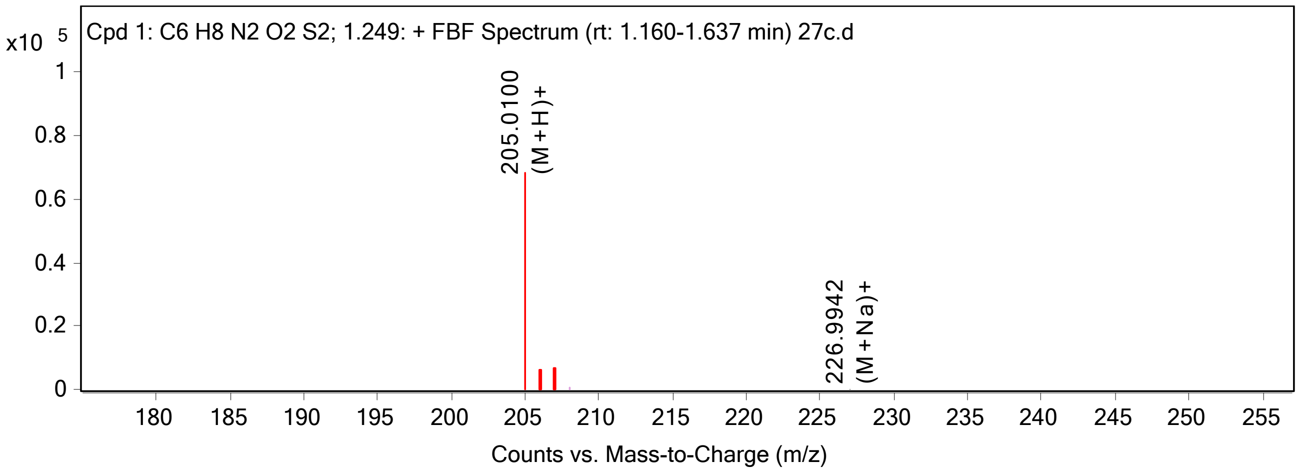

MS Spectrum Peak List

Obs. *m/z*	Charge	Abund	Ion/Isotope
205.01	1	68225.82	(M+H)+
206.0121	1	5695.92	(M+H)+
207.0062	1	5995.99	(M+H)+
226.9942	1	180.07	(M+Na)+

MS Zoomed Spectrum

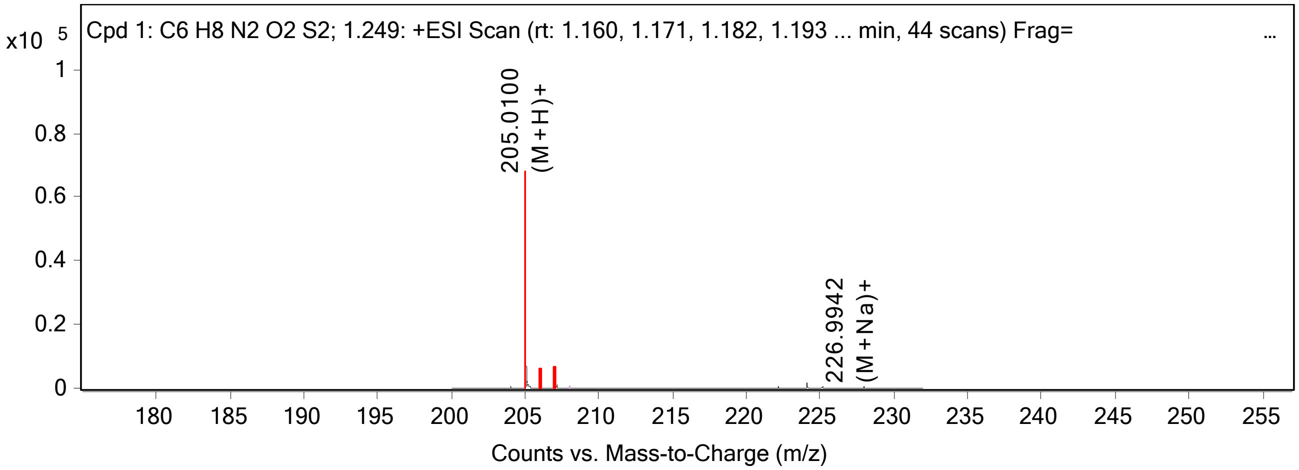

MS Spectrum Peak List

Obs. *m/z*	Charge	Abund	Ion/Isotope	Tgt Mass Error (ppm)
205.01	1	68225.82	(M+H)+	0.18
206.0121	1	5695.92	(M+H)+	0.62
207.0062	1	5995.99	(M+H)+	1.48
226.9942	1	180.07	(M+Na)+	-9.9

--- End Of Report ---

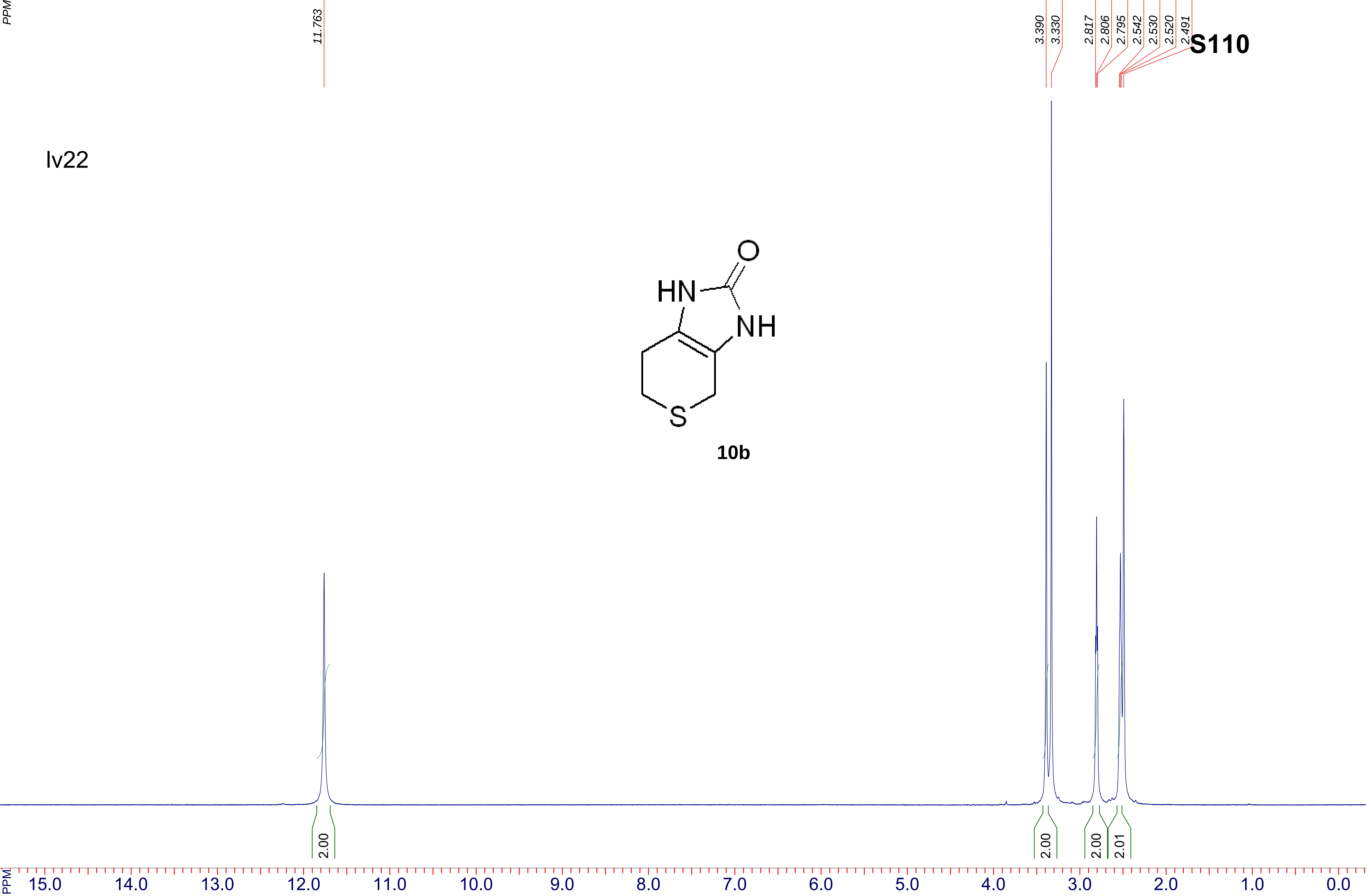

**File name: lv22**	Operator: root	SF: 499.6730 MHz	NSC: 1	PW: 0.00 usec, RG: 32	SI: 32768
Date: 23-Mar-2023	Solvent: DMSO	SW: 8993 Hz	TE: 683 K	AQ: 1.82 sec, RD: 0.00 sec	

S111

lv22_C13

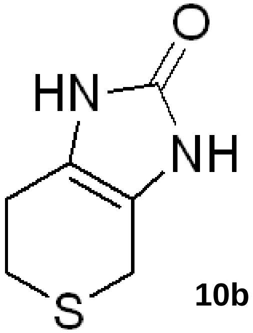

10b

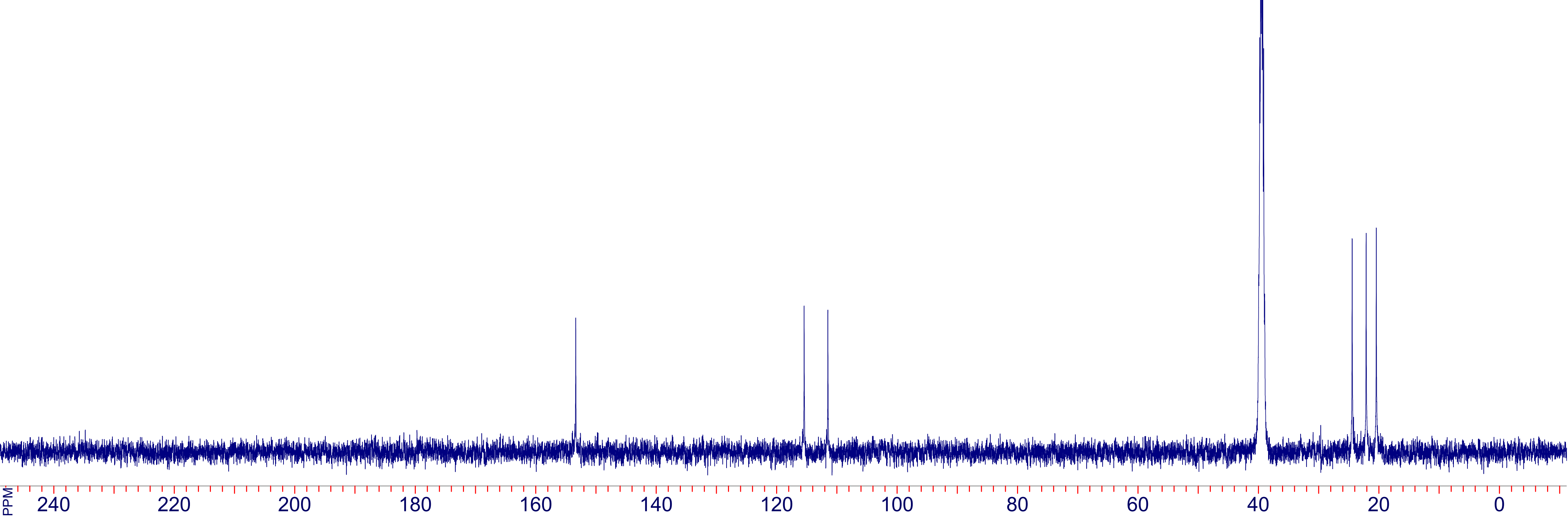

**File name: lv22_C13**	Operator: root	SF: 125.6429 MHz	NSC: 336	PW: 0.00 usec, RG: 51200	SI: 131072
Date: 11-Mar-2023	Solvent: DMSO	SW: 32680 Hz	TE: 683 K	AQ: 1.00 sec, RD: 0.00 sec	

Target Compound Screening Report

**Data File**	25a.d	**Sample Name**	22
**Sample Type**	Sample	**Position**	P1-C7
**Instrument Name**	Instrument 1	**User Name**	Denis V.Bylina
**Acq Method**	Fast_Gradient_HRMS_pos_Lock_08272019.m	**Acquired Time**	4/3/2023 7:20:47 PM (UTC+03:00)
**IRM Calibration Status**	Success	**DA Method**	1.m
**Comment**	Lysenko		
**Sample Group**		**Info.**	Agilent 6224 TOF LC/MS
**MFC**	C6H8N2OS	**Stream Name**	LC 1
**Acquisition Time (Local)**	4/3/2023 7:20:47 PM (UTC+03:00)	**Acquisition SW Version**	6200 series TOF/6500 series Q-TOF B.08.00 (B8058.0)
**TOF Driver Version**	8.00.00	**TOF Firmware Version**	8.643
**Tune Mass Range Max.**	1700		

Compound Table

Label	Tgt Score	Mass Error (ppm)	Tgt Formula	Obs. RT	Ref. Mass	Obs. Mass
Cpd 1: C6 H8 N2 O S; 2.327	95.16	1.59	C6 H8 N2 O S	2.327	156.0357	156.036

Obs. *m/z*	Obs. RT	Obs. Mass	Tgt Formula	Tgt Mass	Tgt Mass Error (ppm)	RT Diff.	Find Cpds Algorithm
335.0603	2.327	156.036	C6 H8 N2 O S	156.0357	1.59	Find By Formula	

Compound Chromatograms

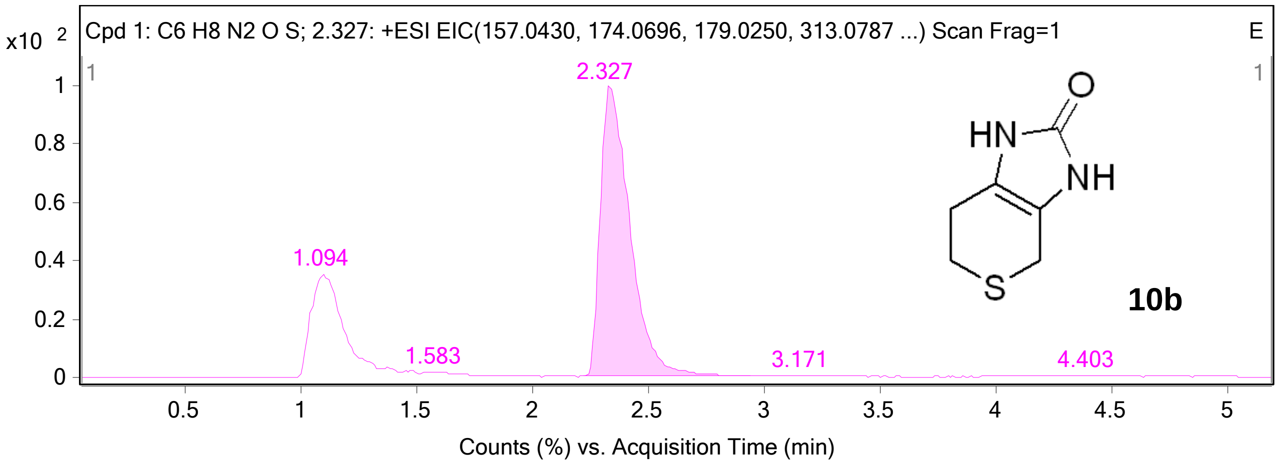

MS Zoomed Spectrum

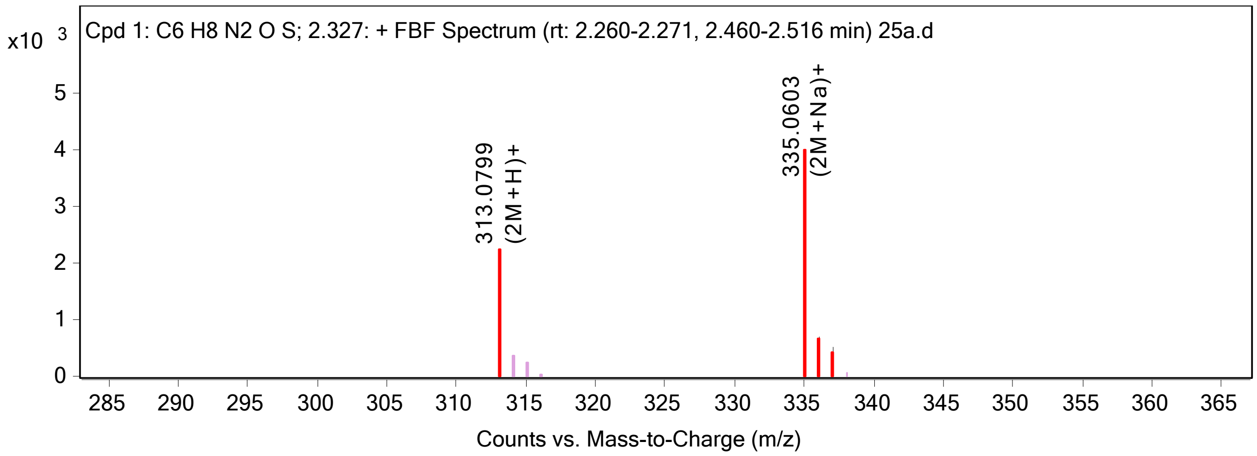

MS Spectrum Peak List

Obs. *m/z*	Charge	Abund	Ion/Isotope
313.0799	1	2251	(2M+H)+
335.0603	1	3846.67	(2M+Na)+
336.0627	1	711.21	(2M+Na)+
337.0599	1	515.39	(2M+Na)+

MS Zoomed Spectrum

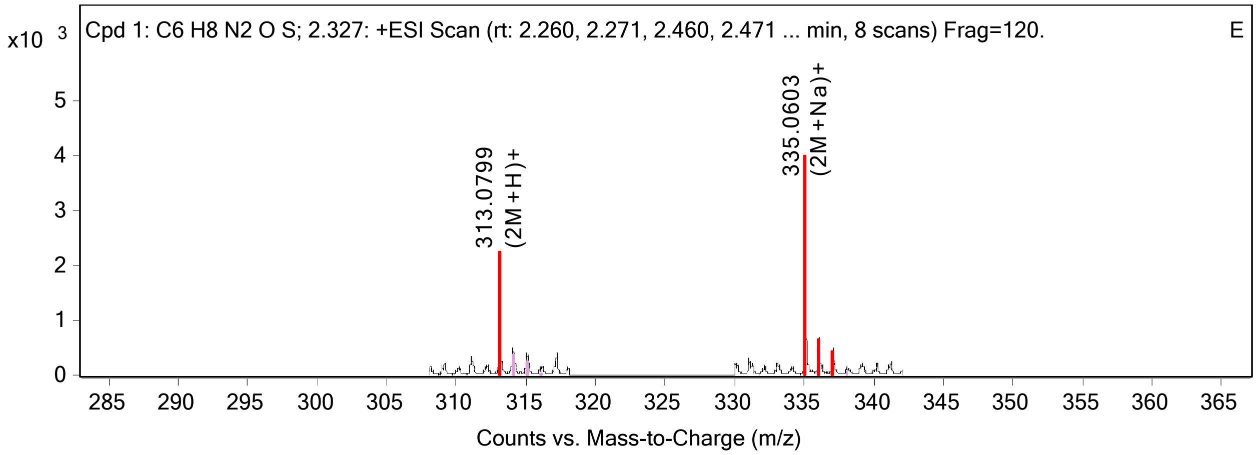

MS Spectrum Peak List

Obs. *m/z*	Charge	Abund	Ion/Isotope	Tgt Mass Error (ppm)
313.0799	1	2251	(2M+H)+	-3.57
335.0603	1	3846.67	(2M+Na)+	1.12
335.0603		3846.67		
336.0627	1	711.21	(2M+Na)+	1.18
337.0599	1	515.39	(2M+Na)+	-5.93

--- End Of Report ---

BB500610-59

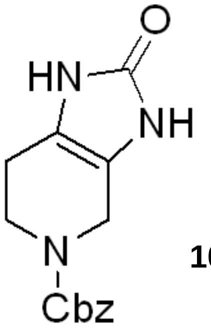

10c

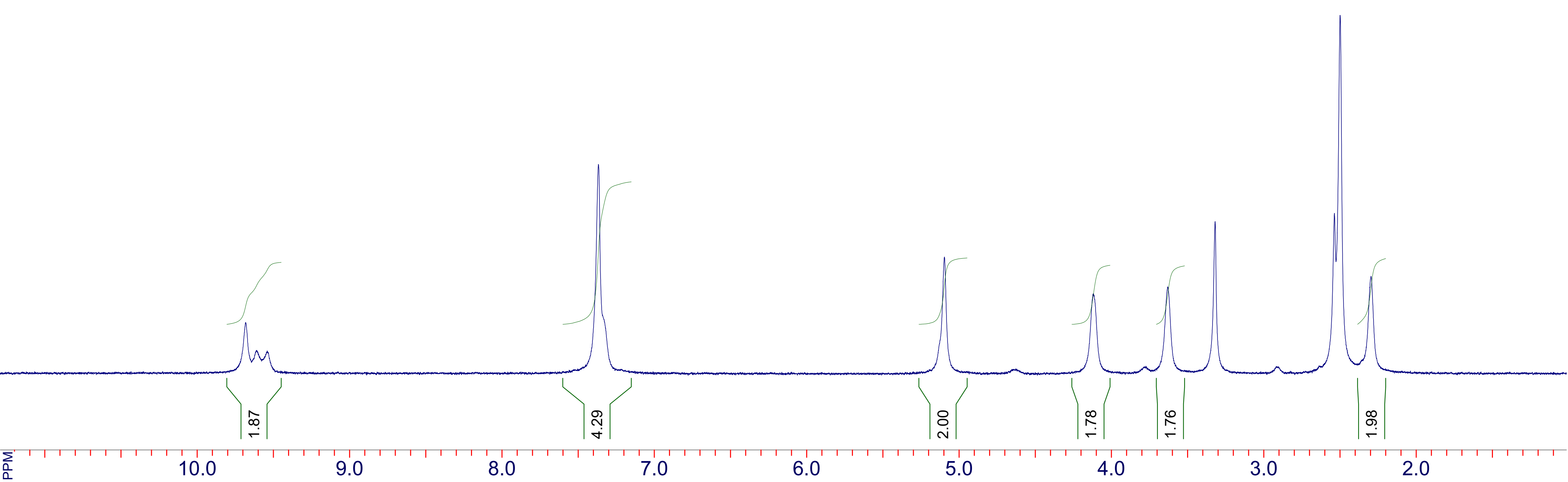

**File name: BB500610-59**	Operator: root	SF: 499.6730 MHz	NSC: 1	PW: 0.00 usec, RG: 32	SI: 32768
Date: 21-Jun-2023	Solvent: DMSO	SW: 8993 Hz	TE: 683 K	AQ: 1.82 sec, RD: 0.00 sec	

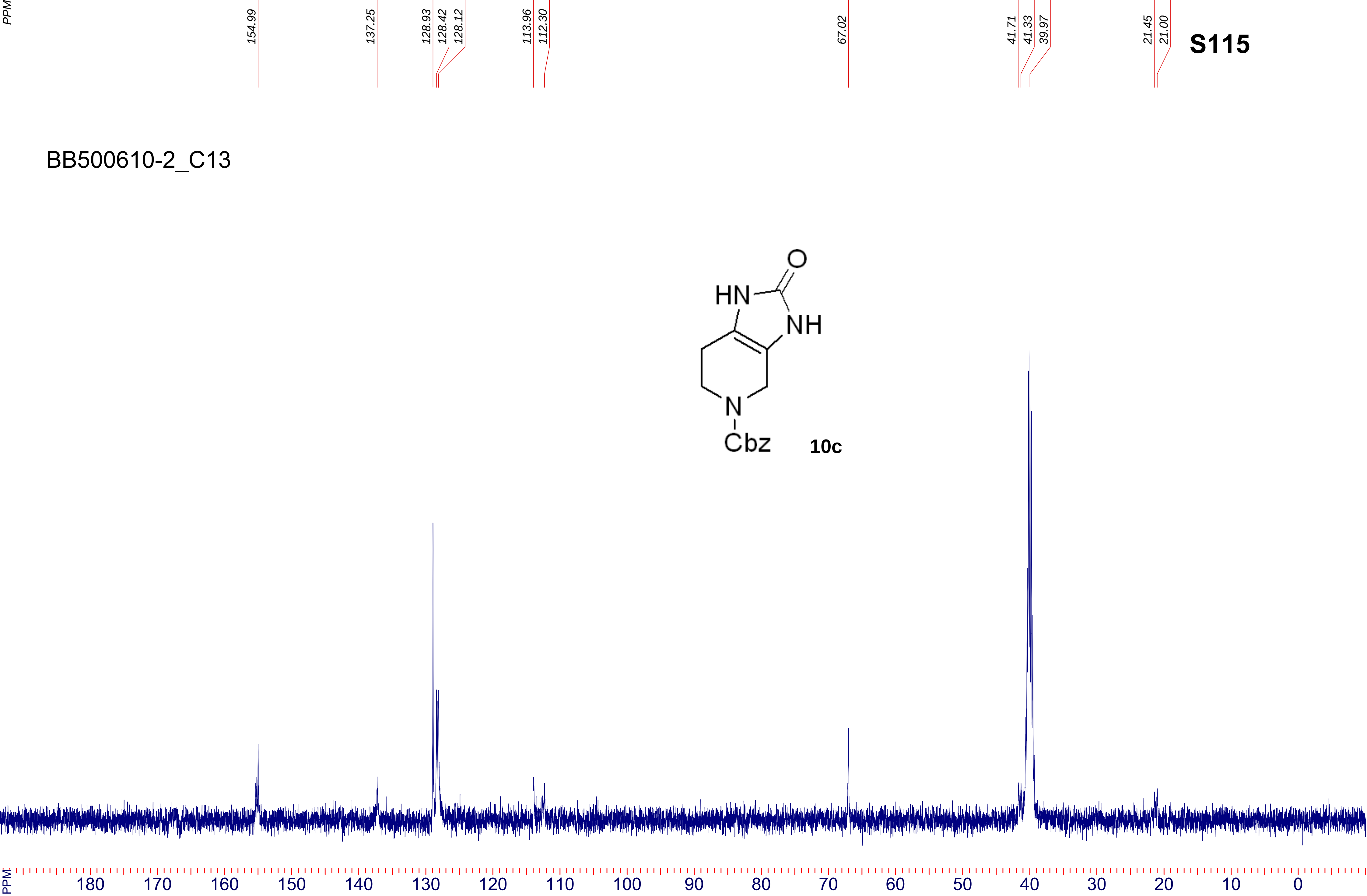

File name: BB500610-2_C13	Operator: nmr	SF: 100.6128 MHz	NSC: 167	PW: 0.00 usec, RG: 2050	SI: 32768
Date: 02-Jun-2023	Solvent: DMSO	SW: 26042 Hz	TE: 300 K	AQ: 0.98 sec, RD: 0.00 sec	

Target Compound Screening Report

**Data File**	11.d	**Sample Name**	24
**Sample Type**	Sample	**Position**	P1-B2
**Instrument Name**	Instrument 1	**User Name**	Denis V.Bylina
**Acq Method**	Fast_Gradient_HRMS_pos_Lock_01312023.m	**Acquired Time**	7/10/2023 10:47:30 AM (UTC+03:00)
**IRM Calibration Status**	Success	**DA Method**	1.m
**Comment**	Lysenko		
**Sample Group**		**Info.**	Agilent 6224 TOF LC/MS
**MFC**	C14H15N3O3	**Stream Name**	LC 1
**Acquisition Time (Local)**	7/10/2023 10:47:30 AM (UTC+03:00)	**Acquisition SW Version**	6200 series TOF/6500 series Q-TOF B.08.00 (B8058.0)
**TOF Driver Version**	8.00.00	**TOF Firmware Version**	8.643
**Tune Mass Range Max.**	1700		

Compound Table

Label	Tgt Score	Mass Error (ppm)	Tgt Formula	Obs. RT	Ref. Mass	Obs. Mass
Cpd 1: C14 H15 N3 O3; 1.980	99.46	0.68	C14 H15 N3 O3	1.98	273.11134	273.11153

Obs. *m/z*	Obs. RT	Obs. Mass	Tgt Formula	Tgt Mass	Tgt Mass Error (ppm)	RT Diff.	Find Cpds Algorithm
274.11881	1.98	273.11153	C14 H15 N3 O3	273.11134	0.68	Find By Formula	

Compound Chromatograms

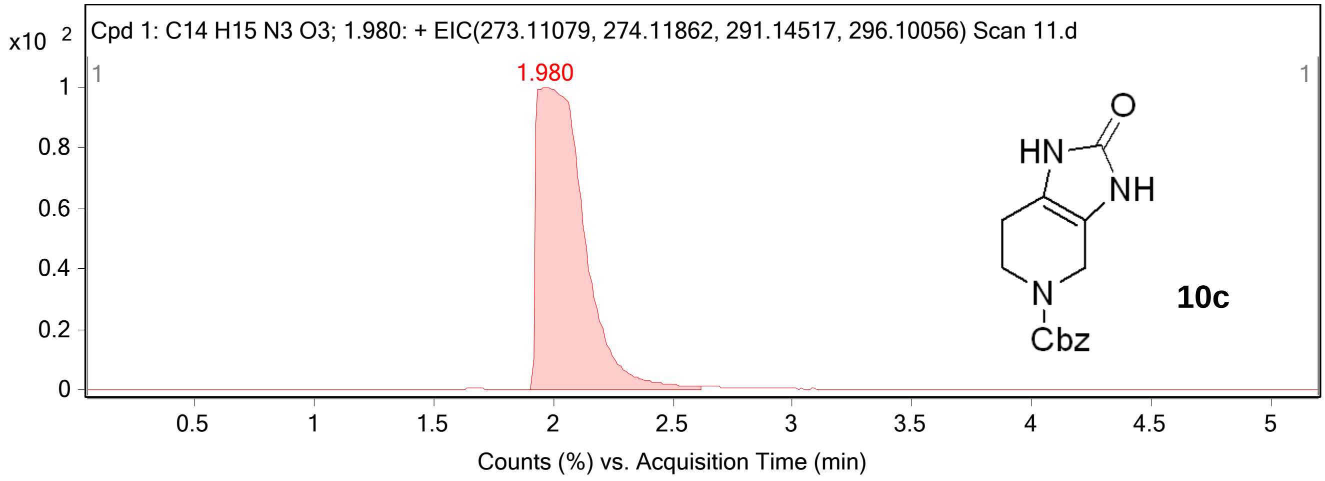

MS Zoomed Spectrum

Target Compound Screening Report

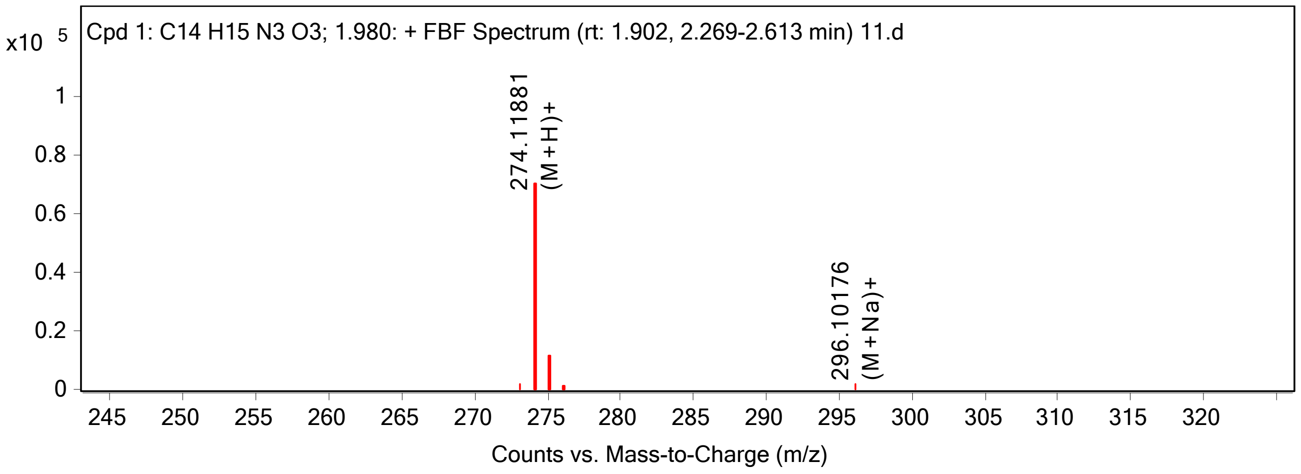

MS Spectrum Peak List

Obs. *m/z*	Charge	Abund	Ion/Isotope
273.10755	1	1665.33	M+
274.11881	1	70234.5	(M+H)+
275.12205	1	11025.8	(M+H)+
276.12451	1	1353.7	(M+H)+
296.10176	1	1776.78	(M+Na)+

MS Zoomed Spectrum

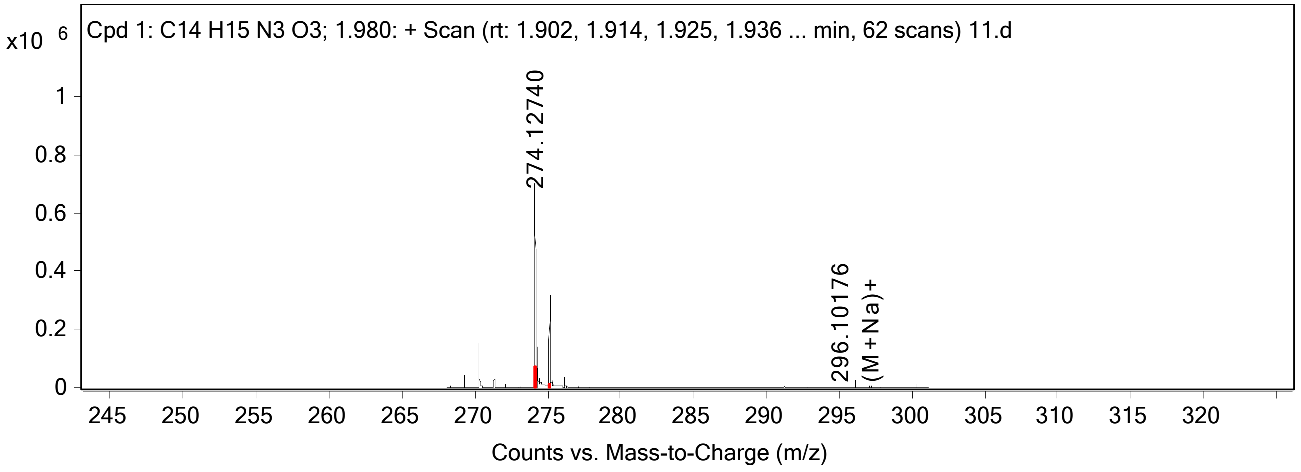

MS Spectrum Peak List

Obs. *m/z*	Charge	Abund	Ion/Isotope	Tgt Mass Error (ppm)
273.10755	1	1665.33	M+	11.87
274.11881	1	70234.5	(M+H)+	-0.7
274.1274		706989.39		
275.12205	1	11025.8	(M+H)+	-1.65
276.12451	1	1353.7	(M+H)+	-1.87
296.10176	1	1776.78	(M+Na)+	-4.04

--- End Of Report ---

BB500610-43.fid

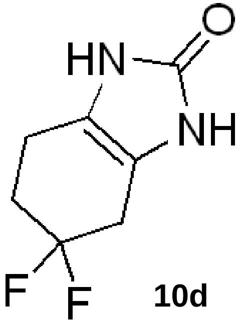

10d

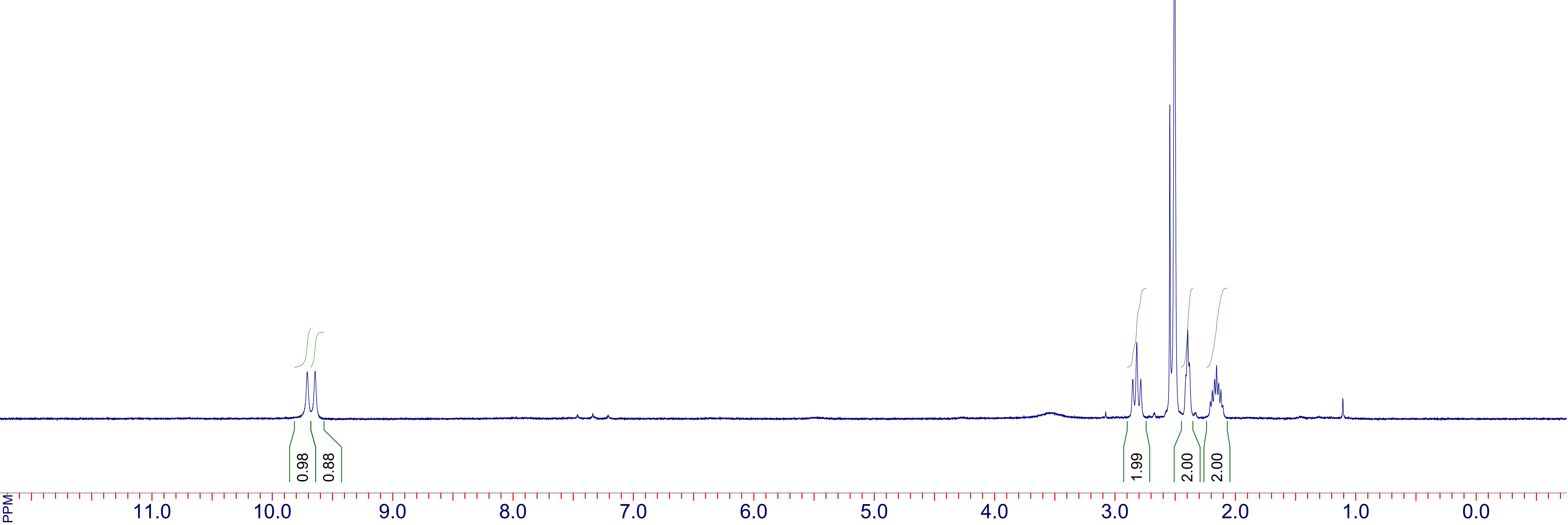

File name: BB500610-43.fid	Operator:	SF: 399.9753 MHz	NSC: 0	PW: 10.90 usec, RG: 20	SI: 32768
Date: 15-Jun-2023	Solvent: dmso	SW: 8000 Hz	TE: 298 K	AQ: 2.00 sec, RD: 0.00 sec	

23_C13

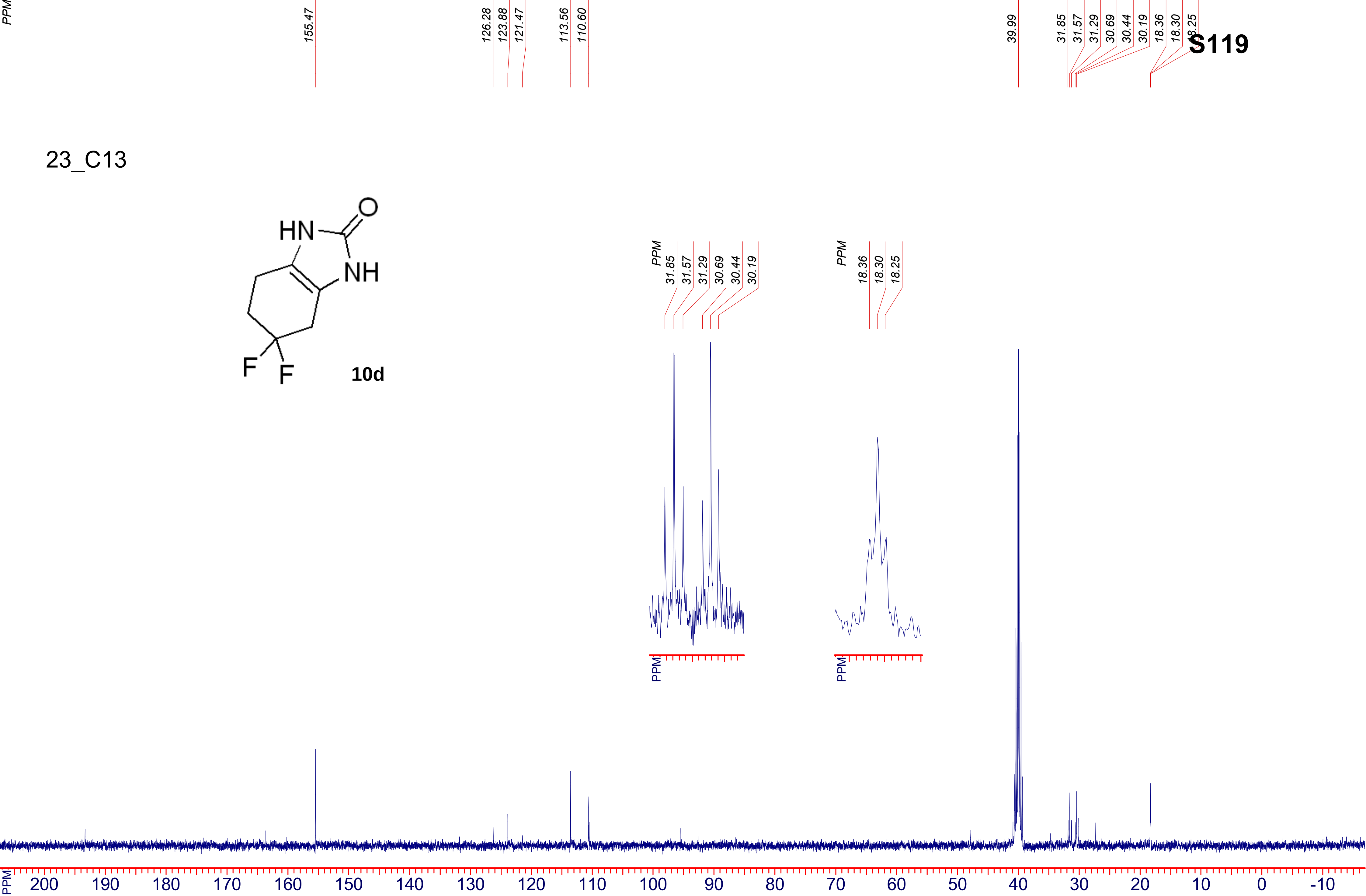

**File name: 23_C13**	Operator: nmr	SF: 100.6128 MHz	NSC: 420	PW: 0.00 usec, RG: 2050	SI: 32768
Date: 30-Jun-2023	Solvent: DMSO	SW: 26042 Hz	TE: 300 K	AQ: 0.98 sec, RD: 0.00 sec	

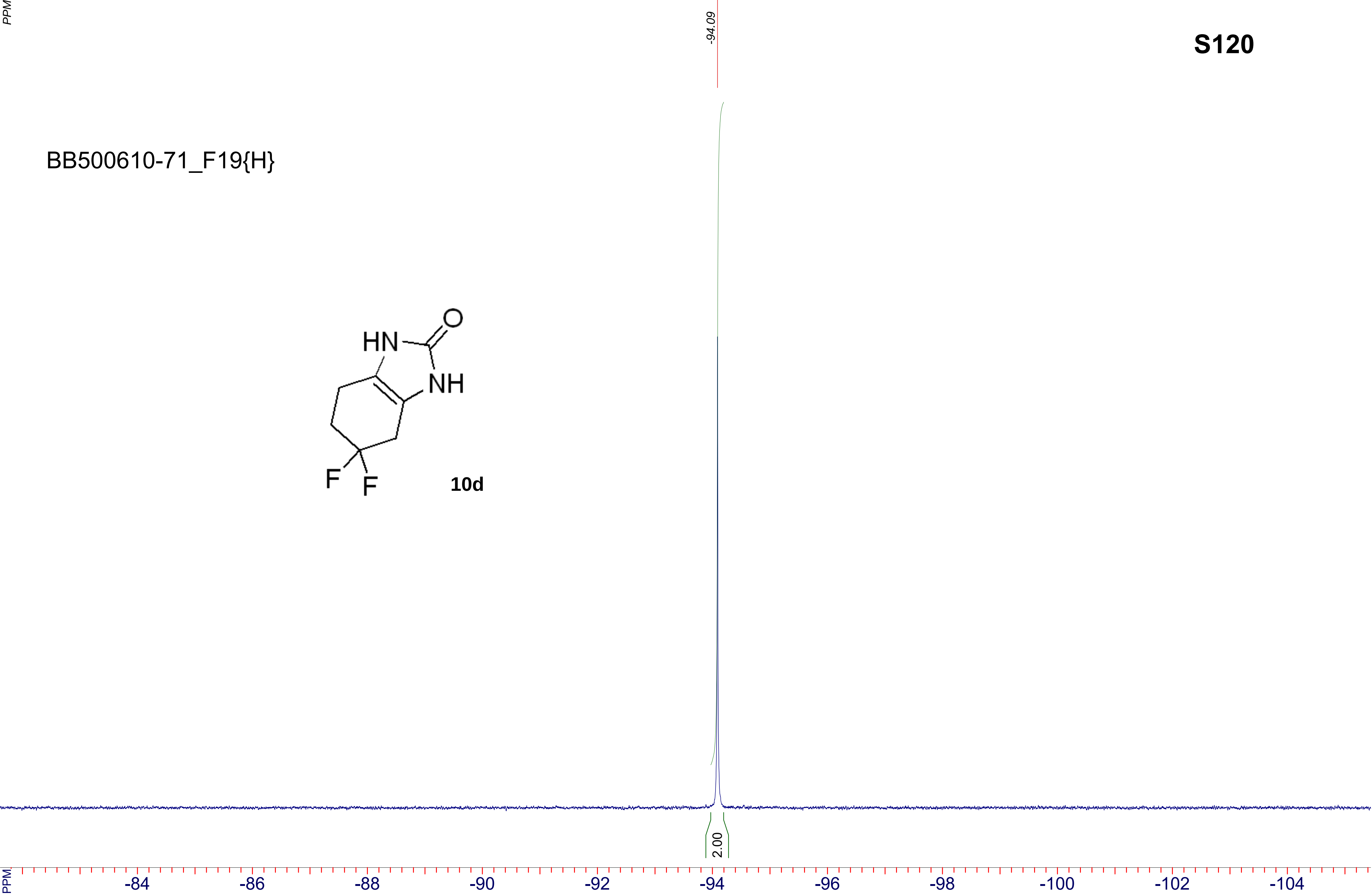

**File name: BB500610-71_F19{H}**	Operator: nmr	SF: 376.4986 MHz	NSC: 1	PW: 0.00 usec, RG: 456	SI: 262144
Date: 22-Jun-2023	Solvent: DMSO	SW: 138889 Hz	TE: 300 K	AQ: 0.94 sec, RD: 0.00 sec	

Target Compound Screening Report

**Data File**	15.d	**Sample Name**	23
**Sample Type**	Sample	**Position**	P1-B6
**Instrument Name**	Instrument 1	**User Name**	Denis V.Bylina
**Acq Method**	Fast_Gradient_HRMS_pos_Lock_01312023.m	**Acquired Time**	7/10/2023 11:11:41 AM (UTC+03:00)
**IRM Calibration Status**	Success	**DA Method**	1.m
**Comment**	Lysenko		
**Sample Group**		**Info.**	Agilent 6224 TOF LC/MS
**MFC**	C7H8F2N2O	**Stream Name**	LC 1
**Acquisition Time (Local)**	7/10/2023 11:11:41 AM (UTC+03:00)	**Acquisition SW Version**	6200 series TOF/6500 series Q-TOF B.08.00 (B8058.0)
**TOF Driver Version**	8.00.00	**TOF Firmware Version**	8.643
**Tune Mass Range Max.**	1700		

Compound Table

Label	Tgt Score	Mass Error (ppm)	Tgt Formula	Obs. RT	Ref. Mass	Obs. Mass
Cpd 1: C7 H8 F2 N2 O; 1.401	86.73	1.71	C7 H8 F2 N2 O	1.401	174.06047	174.06077

Obs. *m/z*	Obs. RT	Obs. Mass	Tgt Formula	Tgt Mass	Tgt Mass Error (ppm)	RT Diff.	Find Cpds Algorithm
175.06804	1.401	174.06077	C7 H8 F2 N2 O	174.06047	1.71	Find By Formula	

Compound Chromatograms

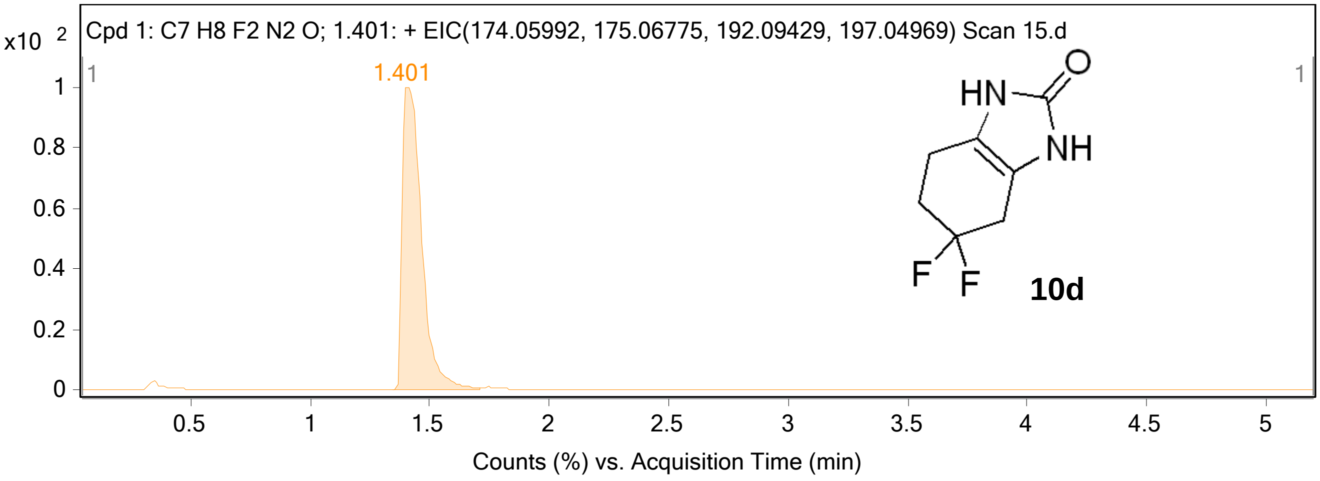

MS Zoomed Spectrum

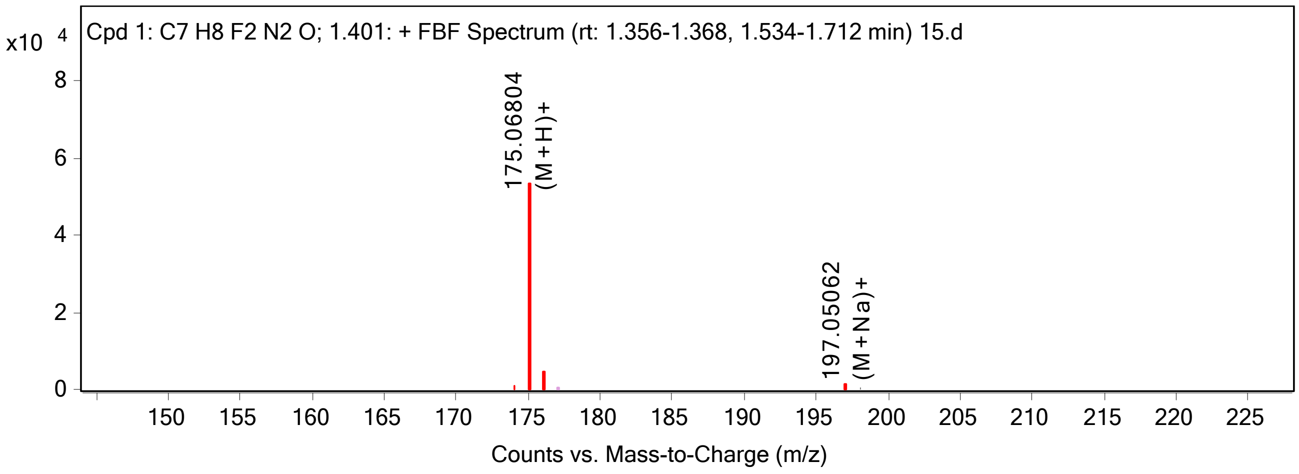

MS Spectrum Peak List

Obs. *m/z*	Charge	Abund	Ion/Isotope
174.05805	1	861.98	M+
175.06804	1	53077.68	(M+H)+
176.07141	1	4739.7	(M+H)+
197.05062	1	1085.39	(M+Na)+
198.0486	1	258.98	(M+Na)+

MS Zoomed Spectrum

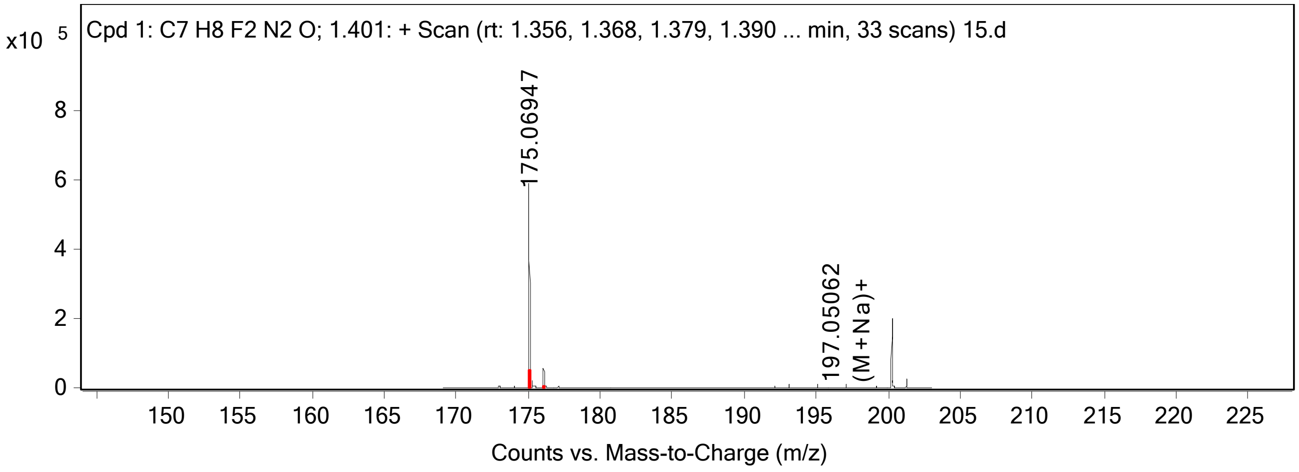

MS Spectrum Peak List

Obs. *m/z*	Charge	Abund	Ion/Isotope	Tgt Mass Error (ppm)
174.05805	1	861.98	M+	10.73
175.06804	1	53077.68	(M+H)+	-1.68
175.06947		590882.88		
176.07141	1	4739.7	(M+H)+	-4.64
197.05062	1	1085.39	(M+Na)+	-4.73
198.0486	1	258.98	(M+Na)+	19.85

--- End Of Report ---

S123

BB500610-79

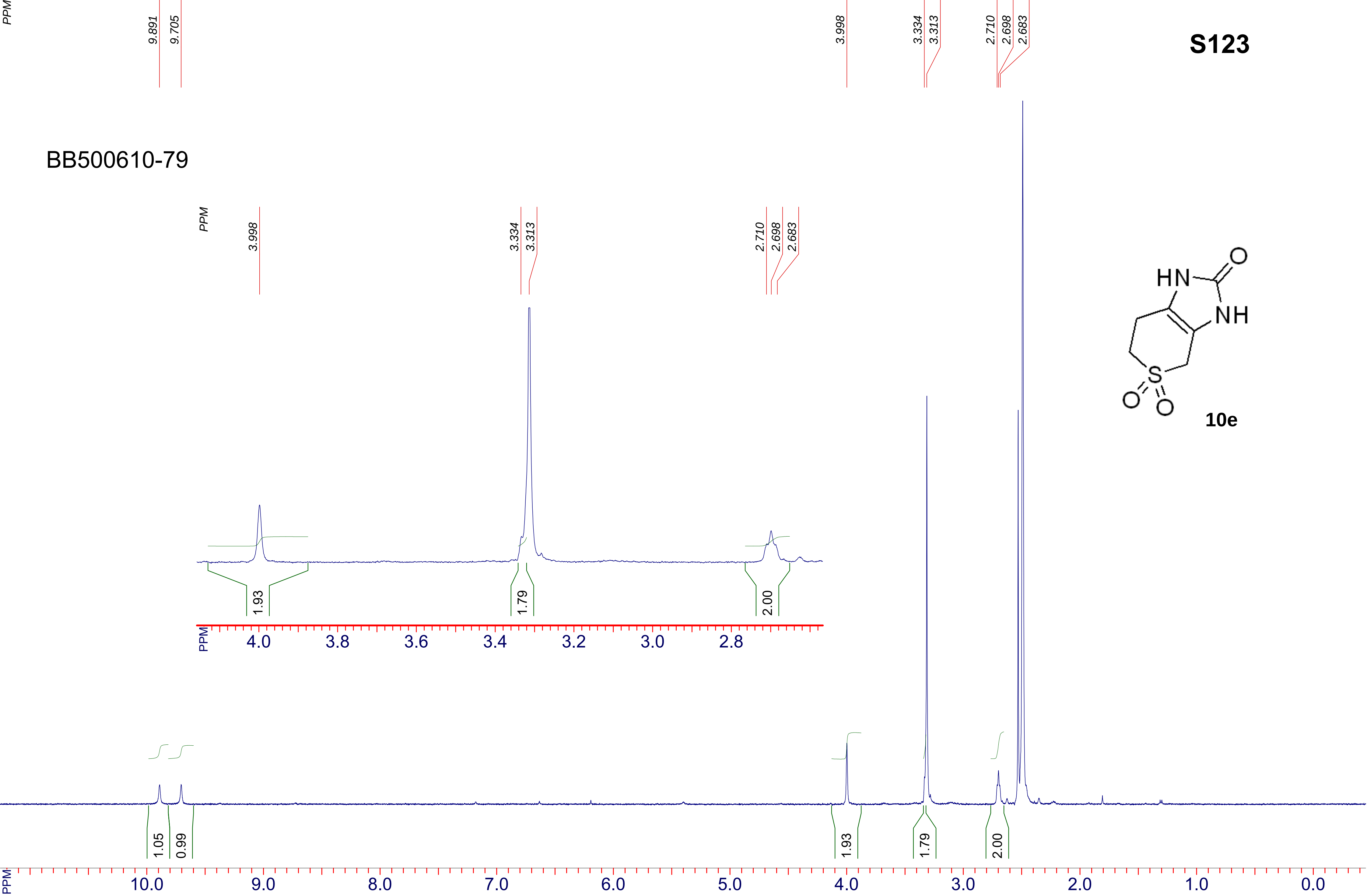

10e

**File name: BB500610-79**	Operator: root	SF: 499.6730 MHz	NSC: 1	PW: 0.00 usec, RG: 32	SI: 32768
Date: 23-Jun-2023	Solvent: DMSO	SW: 8993 Hz	TE: 683 K	AQ: 1.82 sec, RD: 0.00 sec	

BB500610-42_C13

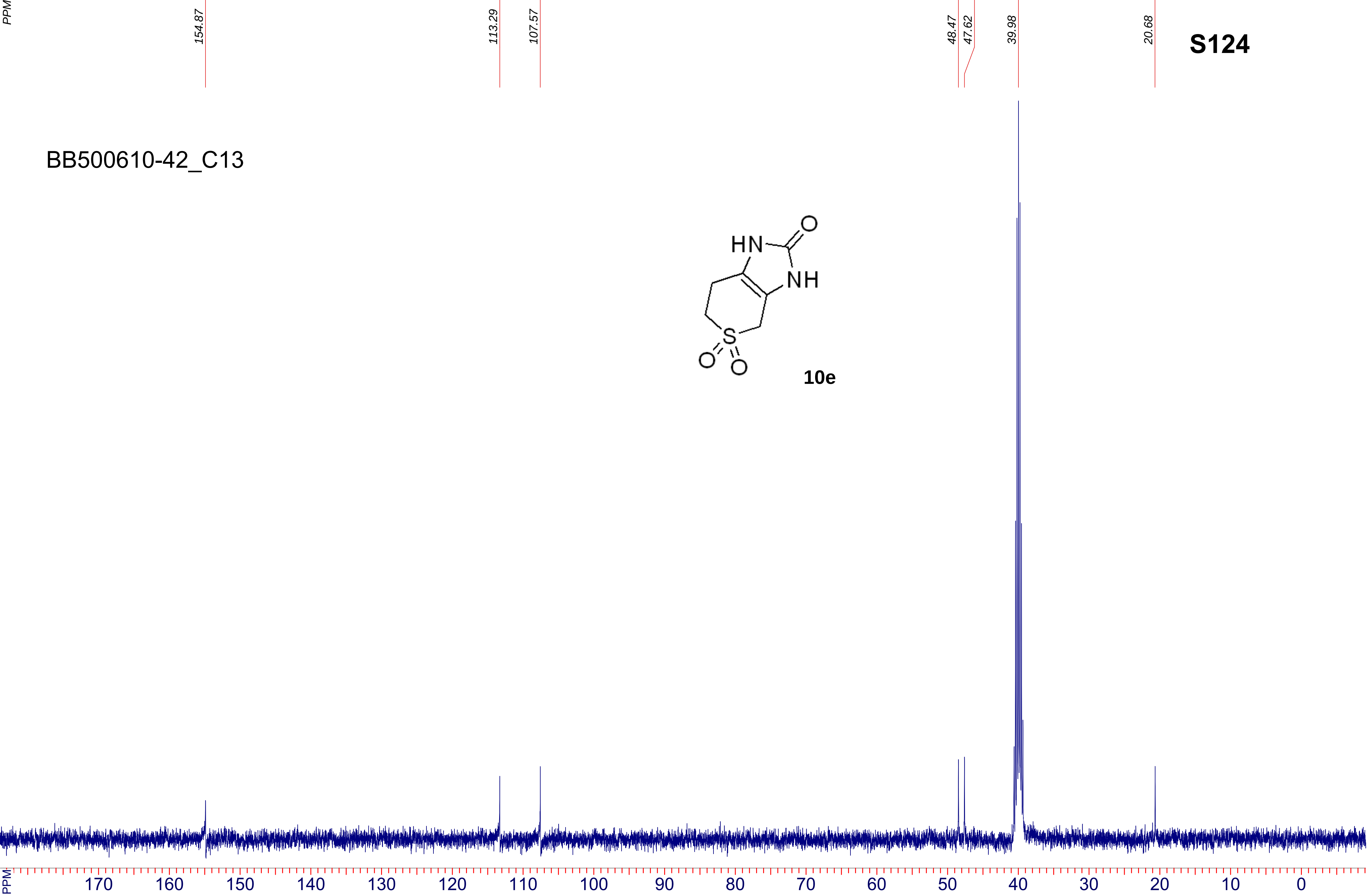

10e

**File name: BB500610-42_C13**	Operator: nmr	SF: 100.6128 MHz	NSC: 206	PW: 0.00 usec, RG: 2050	SI: 32768
Date: 20-Jun-2023	Solvent: DMSO	SW: 26042 Hz	TE: 300 K	AQ: 0.98 sec, RD: 0.00 sec	Parameter file, TOPSPINVersion 2.1

Target Compound Screening Report

**Data File**	12.d	**Sample Name**	26
**Sample Type**	Sample	**Position**	P1-B3
**Instrument Name**	Instrument 1	**User Name**	Denis V.Bylina
**Acq Method**	Fast_Gradient_HRMS_pos_Lock_01312023.m	**Acquired Time**	7/10/2023 10:53:33 AM (UTC+03:00)
**IRM Calibration Status**	Success	**DA Method**	1.m
**Comment**	Lysenko		
**Sample Group**		**Info.**	Agilent 6224 TOF LC/MS
**MFC**	C6H8N2O3S	**Stream Name**	LC 1
**Acquisition Time (Local)**	7/10/2023 10:53:33 AM (UTC+03:00)	**Acquisition SW Version**	6200 series TOF/6500 series Q-TOF B.08.00 (B8058.0)
**TOF Driver Version**	8.00.00	**TOF Firmware Version**	8.643
**Tune Mass Range Max.**	1700		

Compound Table

Label	Tgt Score	Mass Error (ppm)	Tgt Formula	Obs. RT	Ref. Mass	Obs. Mass
Cpd 1: C6 H8 N2 O3 S; 0.456	98.33	2.07	C6 H8 N2 O3 S	0.456	188.02556	188.02595

Obs. *m/z*	Obs. RT	Obs. Mass	Tgt Formula	Tgt Mass	Tgt Mass Error (ppm)	RT Diff.	Find Cpds Algorithm
189.03323	0.456	188.02595	C6 H8 N2 O3 S	188.02556	2.07	Find By Formula	

Compound Chromatograms

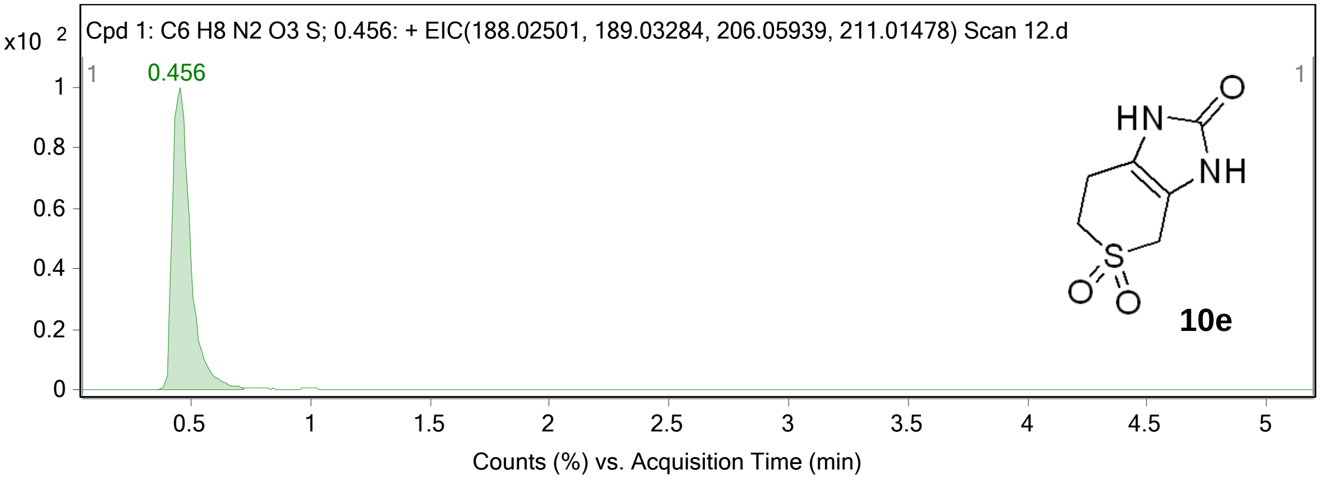

MS Zoomed Spectrum

Target Compound Screening Report

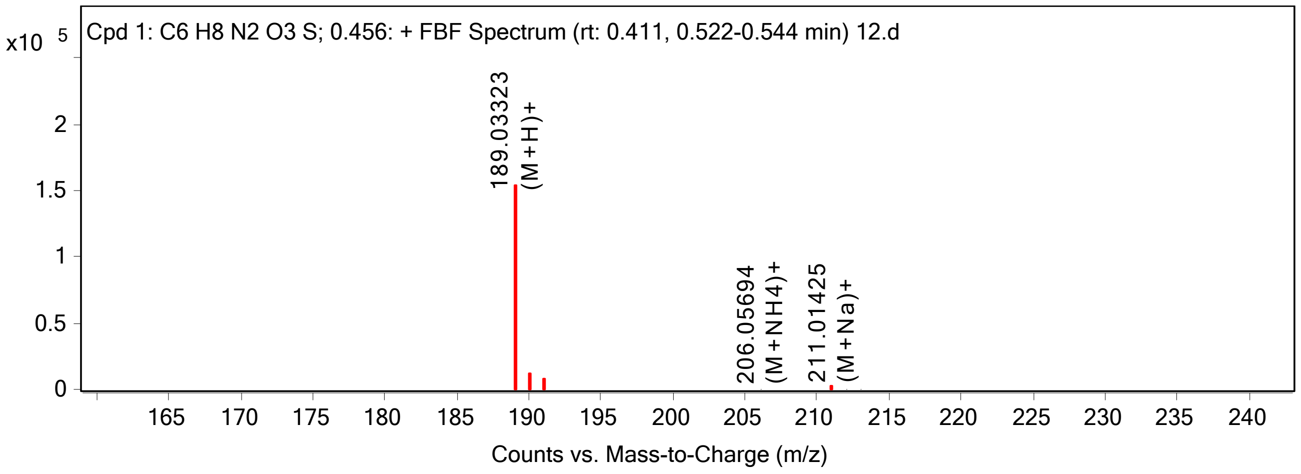

MS Spectrum Peak List

**Obs. *m/z***	**Charge**	**Abund**	**Ion/Isotope**
189.03323	1	154435.7	(M+H)+
190.0361	1	11578.34	(M+H)+
191.03045	1	6778.51	(M+H)+
206.05694	1	343.05	(M+NH4)+
211.01425	1	2446.09	(M+Na)+
212.01538	1	393.61	(M+Na)+
213.01155	1	359.34	(M+Na)+

MS Zoomed Spectrum

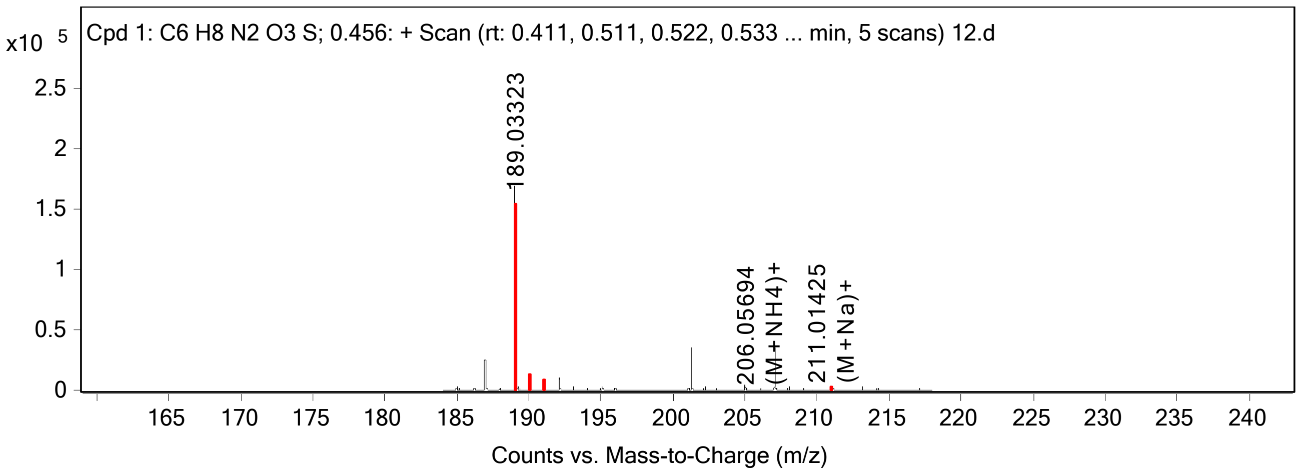

MS Spectrum Peak List

**Obs. *m/z***	**Charge**	**Abund**	**Ion/Isotope**	**Tgt Mass Error (ppm)**
189.03323		170418.43		
189.03323	1	154435.7	(M+H)+	-2.06
190.0361	1	11578.34	(M+H)+	-4.23
191.03045	1	6778.51	(M+H)+	-1.81
206.05694	1	343.05	(M+NH4)+	11.86
211.01425	1	2446.09	(M+Na)+	2.52
212.01538	1	393.61	(M+Na)+	8.76
213.01155	1	359.34	(M+Na)+	2.33

--- End Of Report ---

S127

lv17.fid

11a

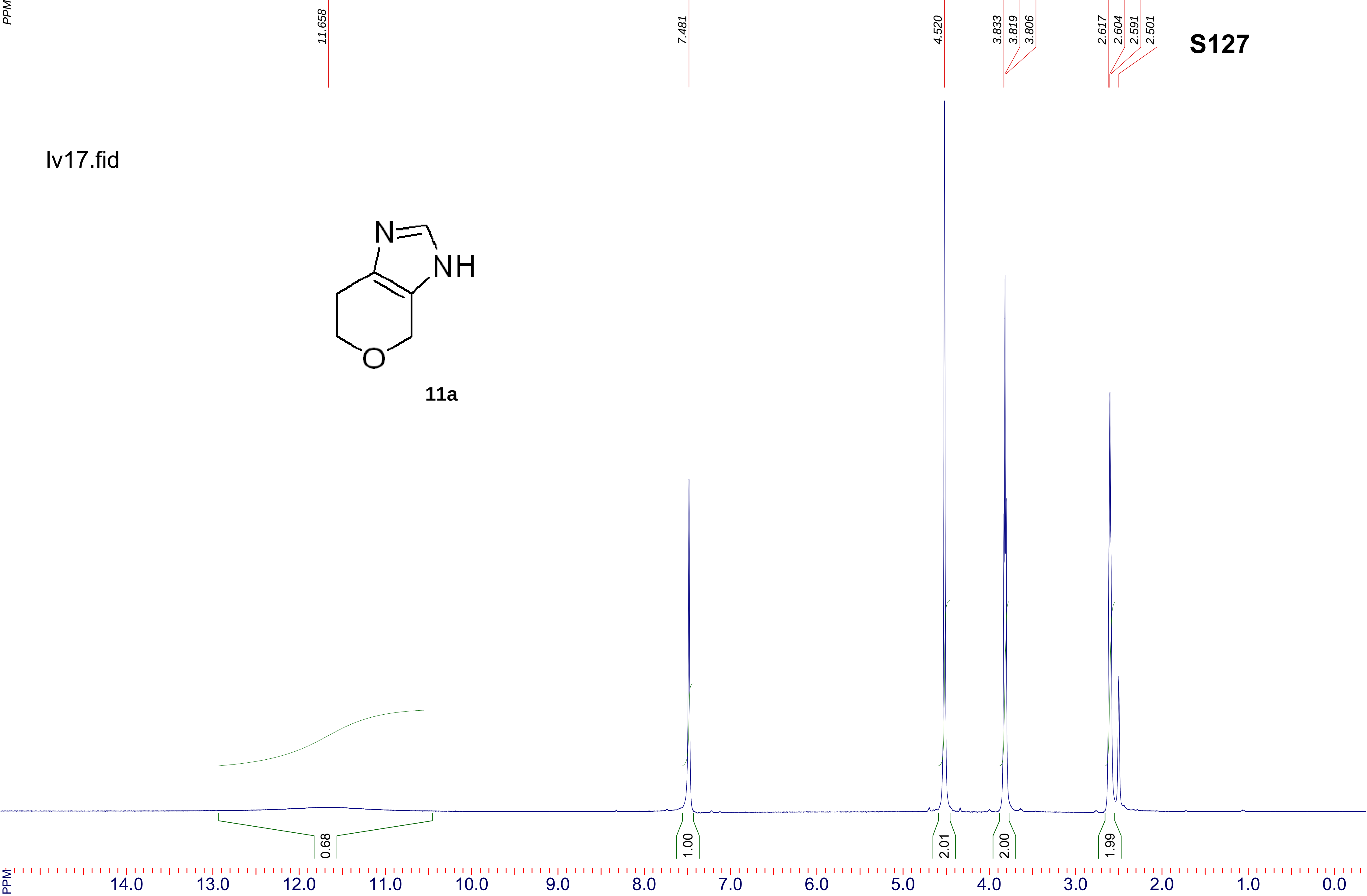

**File name: lv17.fid**	Operator:	SF: 399.9733 MHz	NSC: 0	PW: 10.90 usec, RG: 24	SI: 32768
Date: 09-Mar-2023	Solvent: dmso	SW: 8000 Hz	TE: 298 K	AQ: 2.00 sec, RD: 0.00 sec	

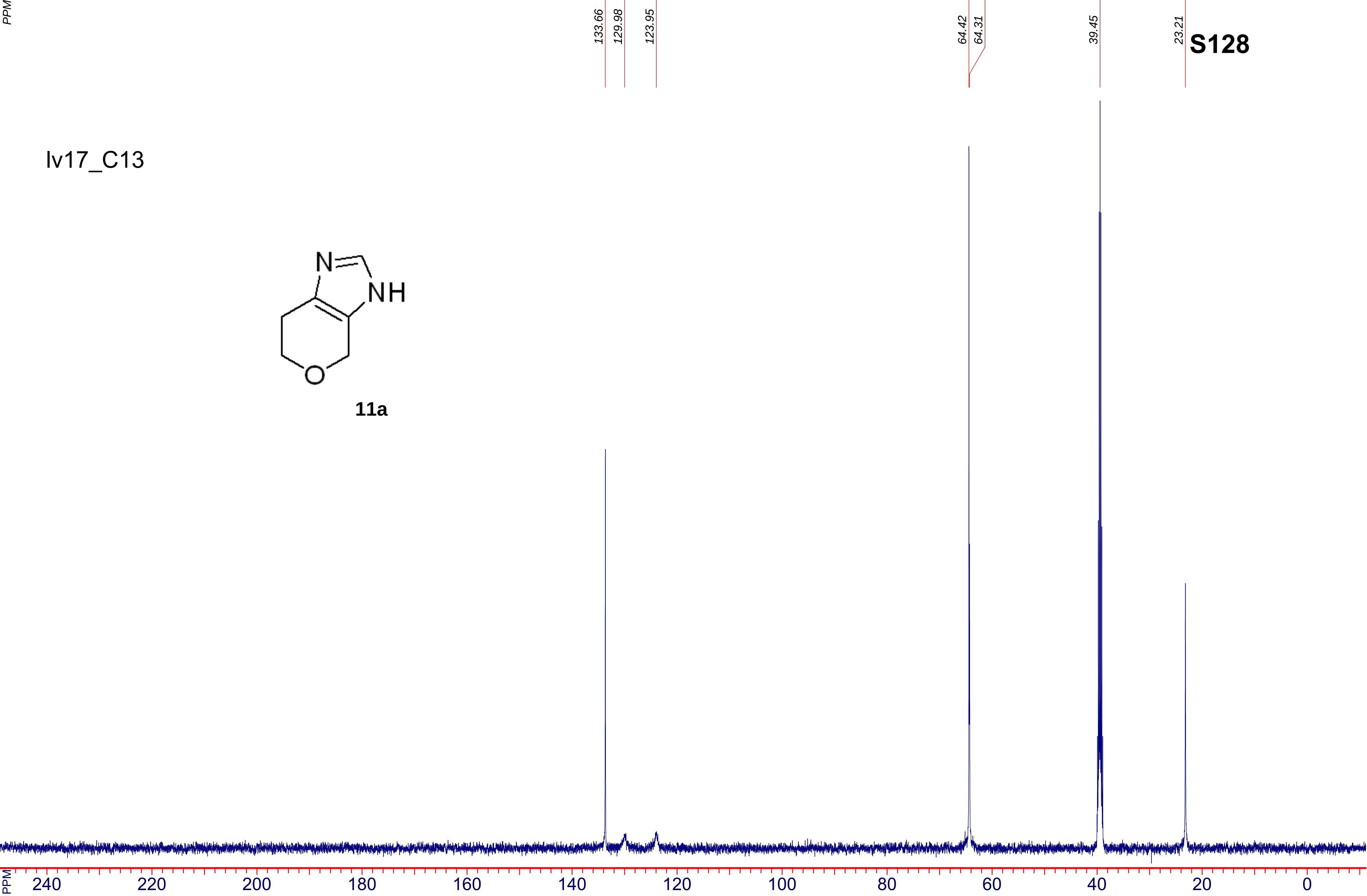

11a

**File name: lv17_C13**	Operator: root	SF: 125.6429 MHz	NSC: 259	PW: 0.00 usec, RG: 51200	SI: 131072
Date: 11-Mar-2023	Solvent: DMSO	SW: 32680 Hz	TE: 683 K	AQ: 1.00 sec, RD: 0.00 sec	

Target Compound Screening Report

**Data File**	22a.d	**Sample Name**	17
**Sample Type**	Sample	**Position**	P1-C4
**Instrument Name**	Instrument 1	**User Name**	Denis V.Bylina
**Acq Method**	Fast_Gradient_HRMS_pos_Lock_08272019.m	**Acquired Time**	4/3/2023 7:03:12 PM (UTC+03:00)
**IRM Calibration Status**	Success	**DA Method**	1.m
**Comment**	Lysenko		
**Sample Group**		**Info.**	Agilent 6224 TOF LC/MS
**MFC**	C6H8N2O	**Stream Name**	LC 1
**Acquisition Time (Local)**	4/3/2023 7:03:12 PM (UTC+03:00)	**Acquisition SW Version**	6200 series TOF/6500 series Q-TOF B.08.00 (B8058.0)
**TOF Driver Version**	8.00.00	**TOF Firmware Version**	8.643
**Tune Mass Range Max.**	1700		

Compound Table

Label	Tgt Score	Mass Error (ppm)	Tgt Formula	Obs. RT	Ref. Mass	Obs. Mass
Cpd 1: C6 H8 N2 O; 1.136	47.58	-0.8	C6 H8 N2 O	1.136	124.0637	124.0636

Obs. *m/z*	Obs. RT	Obs. Mass	Tgt Formula	Tgt Mass	Tgt Mass Error (ppm)	RT Diff.	Find Cpds Algorithm
147.0528	1.136	124.0636	C6 H8 N2 O	124.0637	-0.8	Find By Formula	

Compound Chromatograms

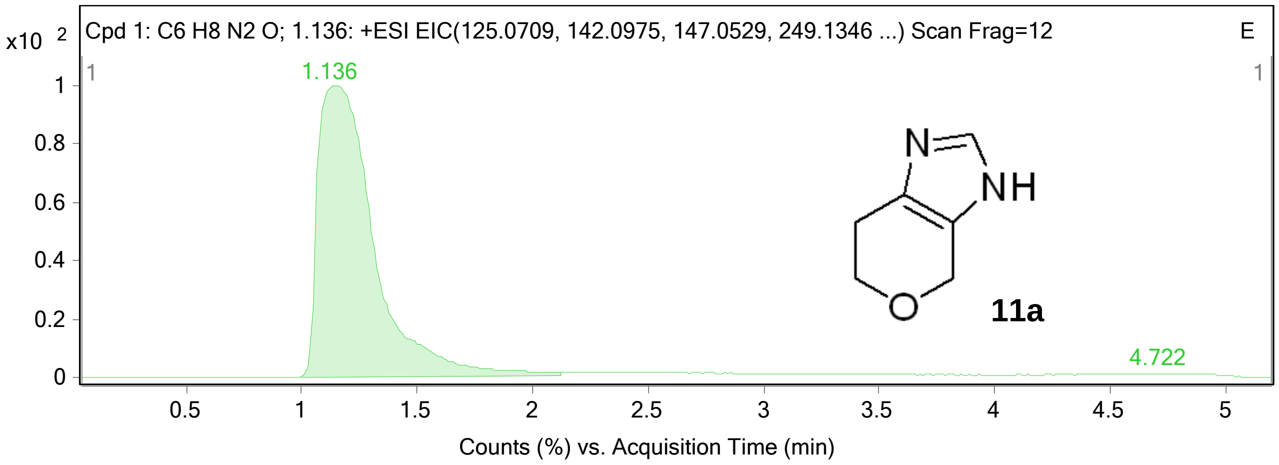

MS Zoomed Spectrum

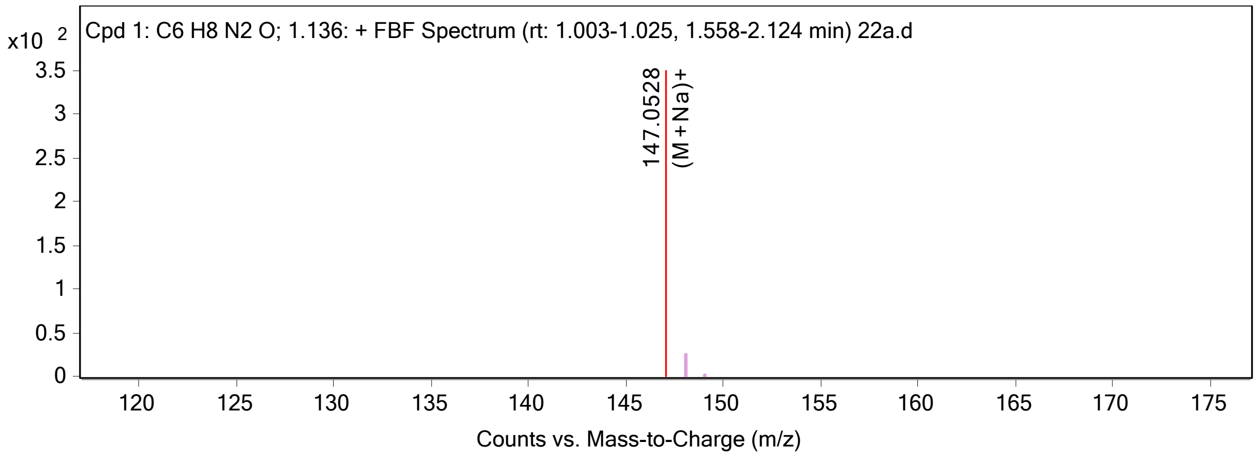

Target Compound Screening Report

MS Spectrum Peak List

Obs. *m/z*	Charge	Abund	Ion/Isotope
147.0528	1	350	(M+Na)+

MS Zoomed Spectrum

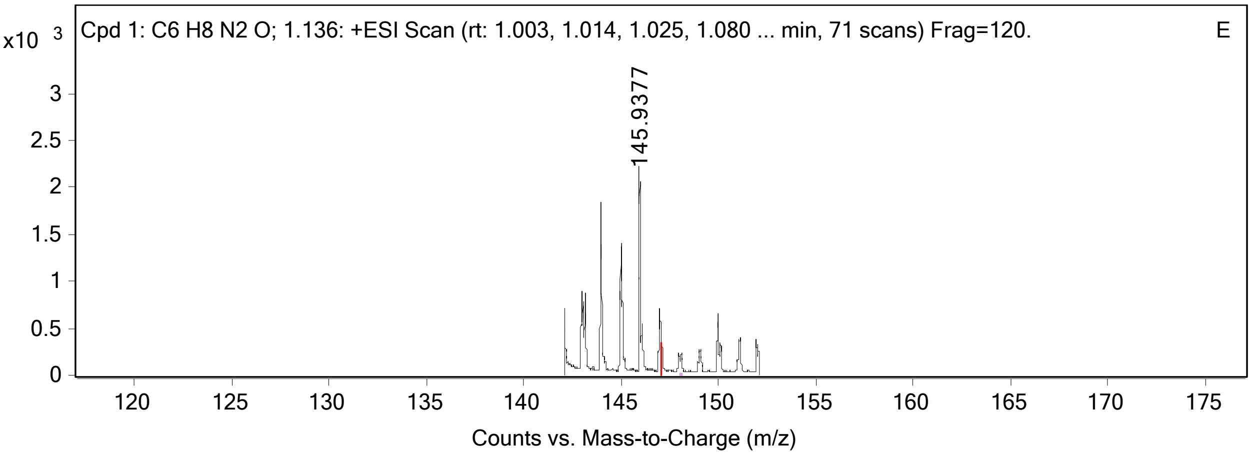

MS Spectrum Peak List

Obs. *m/z*	Charge	Abund	Ion/Isotope	Tgt Mass Error (ppm)
145.9377		2226.38		
147.0528	1	350	(M+Na)+	0.68

--- End Of Report ---

BB500610-66

11c

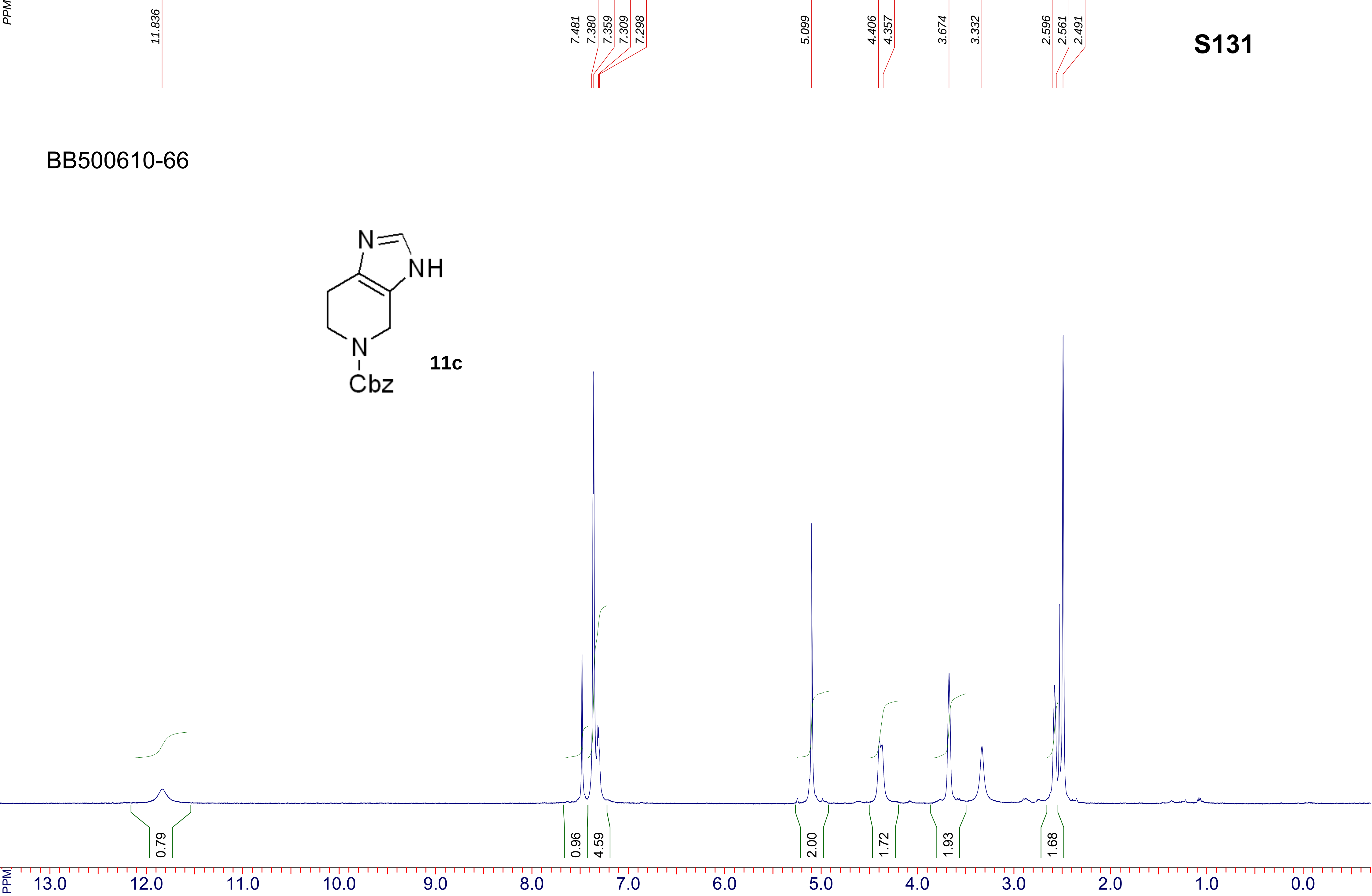

**File name: BB500610-66**	Operator: root	SF: 499.6730 MHz	NSC: 1	PW: 0.00 usec, RG: 32	SI: 32768
Date: 23-Jun-2023	Solvent: DMSO	SW: 8993 Hz	TE: 683 K	AQ: 1.82 sec, RD: 0.00 sec	

S132

BB500610-81_C13

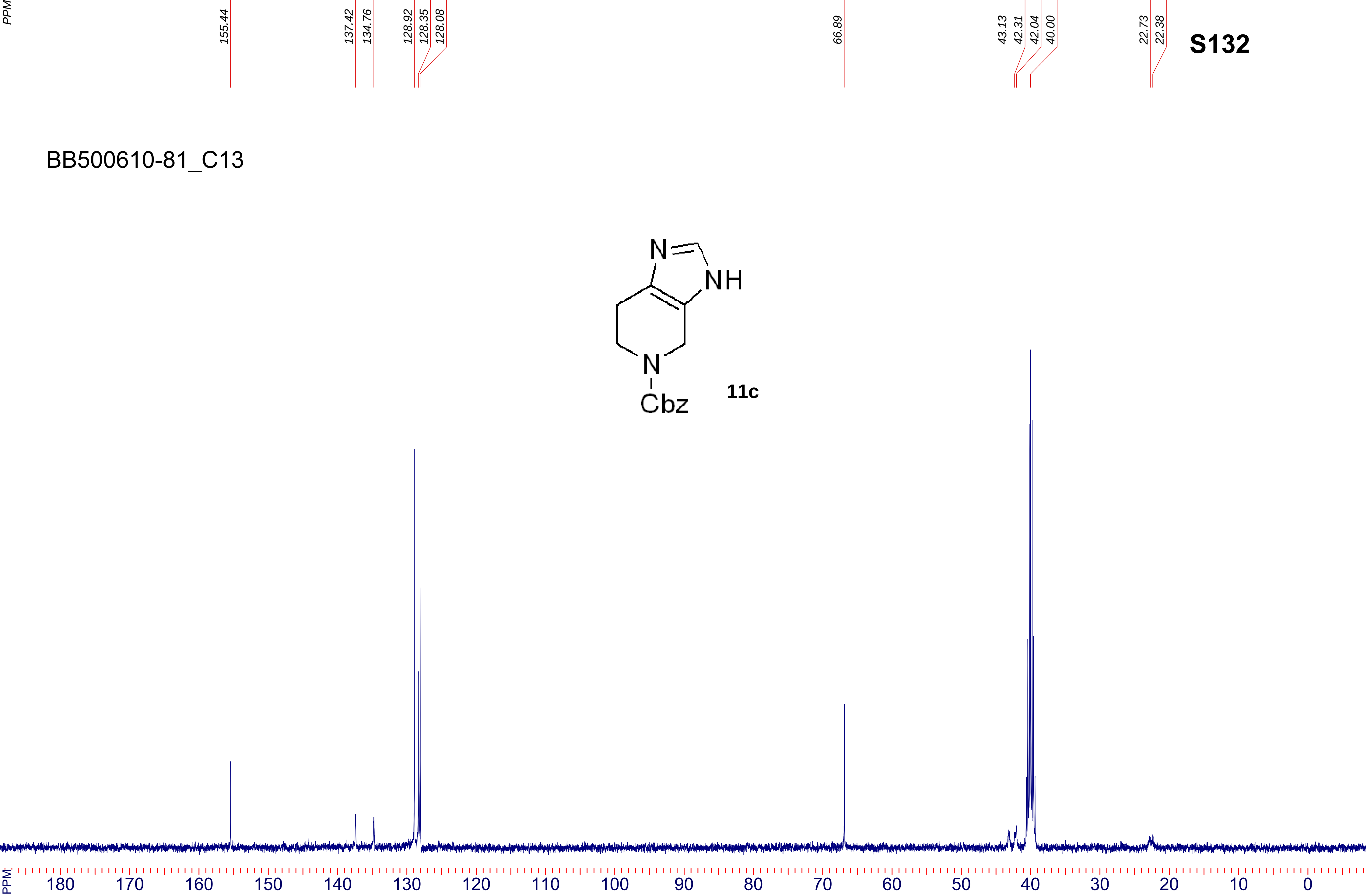

11c

**File name: BB500610-81_C13**	Operator: nmr	SF: 100.6128 MHz	NSC: 310	PW: 0.00 usec, RG: 2050	SI: 32768
Date: 23-Jun-2023	Solvent: DMSO	SW: 26042 Hz	TE: 300 K	AQ: 0.98 sec, RD: 0.00 sec	

Target Compound Screening Report

**Data File**	14.d	**Sample Name**	19
**Sample Type**	Sample	**Position**	P1-B5
**Instrument Name**	Instrument 1	**User Name**	Denis V.Bylina
**Acq Method**	Fast_Gradient_HRMS_pos_Lock_01312023.m	**Acquired Time**	7/10/2023 11:05:38 AM (UTC+03:00)
**IRM Calibration Status**	Success	**DA Method**	1.m
**Comment**	Lysenko		
**Sample Group**		**Info.**	Agilent 6224 TOF LC/MS
**MFC**	C14H15N3O2	**Stream Name**	LC 1
**Acquisition Time (Local)**	7/10/2023 11:05:38 AM (UTC+03:00)	**Acquisition SW Version**	6200 series TOF/6500 series Q-TOF B.08.00 (B8058.0)
**TOF Driver Version**	8.00.00	**TOF Firmware Version**	8.643
**Tune Mass Range Max.**	1700		

Compound Table

Label	Tgt Score	Mass Error (ppm)	Tgt Formula	Obs. RT	Ref. Mass	Obs. Mass
Cpd 3: C14 H15 N3 O2; 1.510	85.4	2.42	C14 H15 N3 O2	1.51	257.11643	257.11705

Obs. *m/z*	Obs. RT	Obs. Mass	Tgt Formula	Tgt Mass	Tgt Mass Error (ppm)	RT Diff.	Find Cpds Algorithm
258.12433	1.51	257.11705	C14 H15 N3 O2	257.11643	2.42	Find By Formula	

Compound Chromatograms

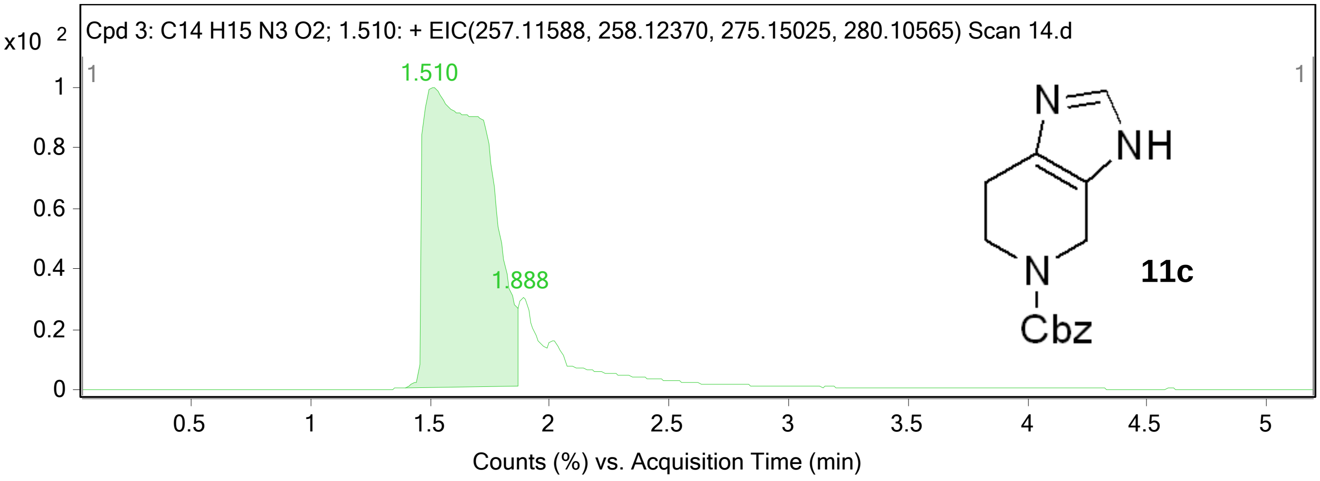

MS Zoomed Spectrum

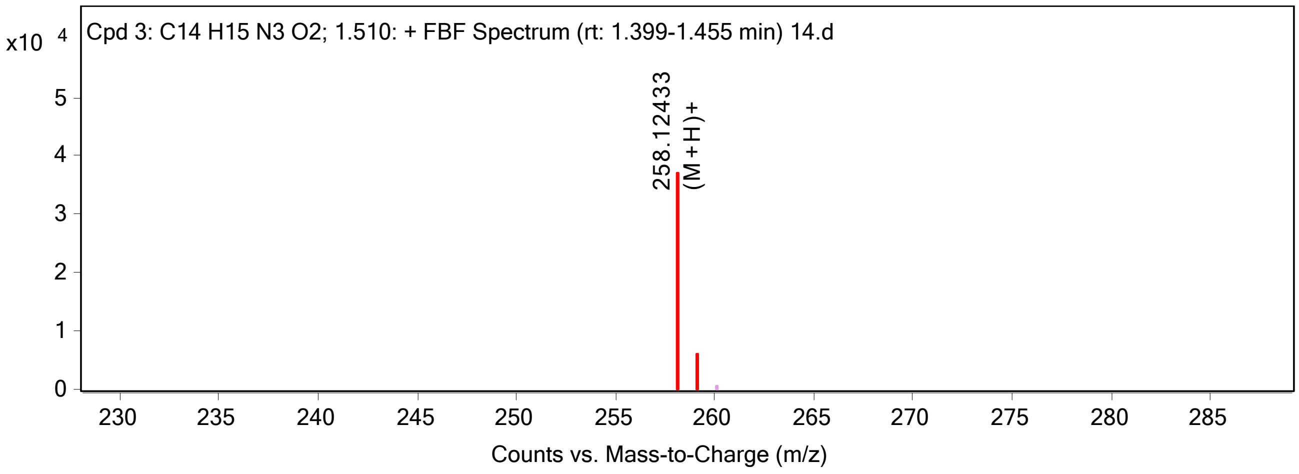

MS Spectrum Peak List

Obs. *m/z*	Charge	Abund	Ion/Isotope
258.12433	1	37132.64	(M+H)+
259.12728	1	5969.58	(M+H)+

MS Zoomed Spectrum

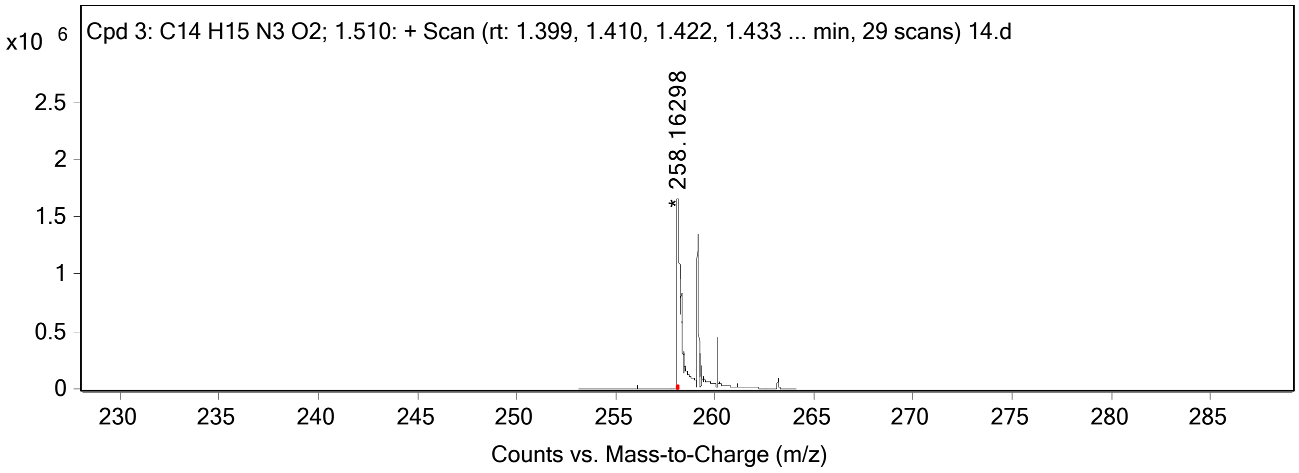

MS Spectrum Peak List

Obs. *m/z*	Charge	Abund	Ion/Isotope	Tgt Mass Error (ppm)
258.12433	1	37132.64	(M+H)+	-2.42
258.16298		1666108.21		
259.12728	1	5969.58	(M+H)+	-2.35

--- End Of Report ---

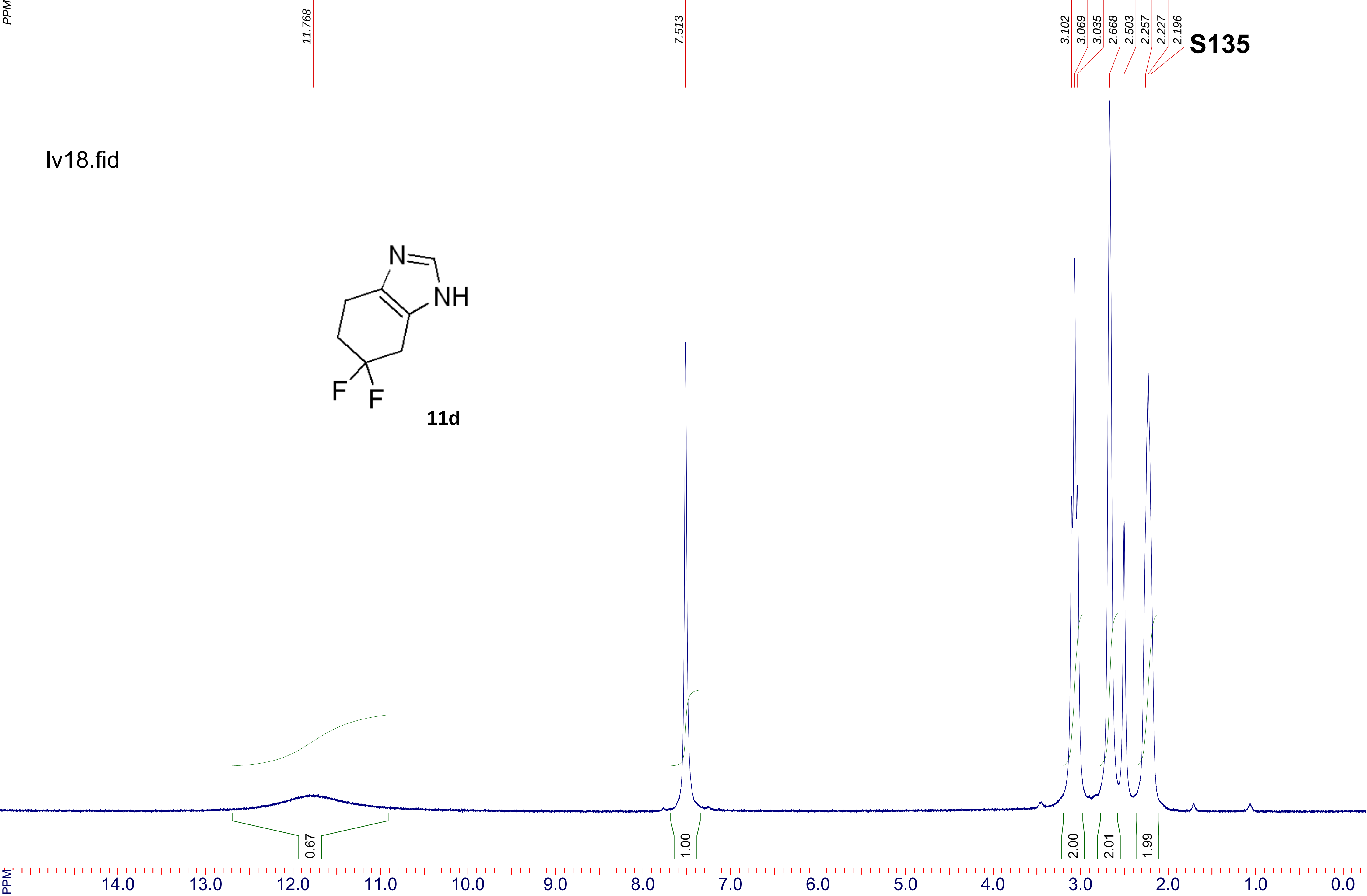

**File name: lv18.fid**	Operator:	SF: 399.9733 MHz	NSC: 0	PW: 10.90 usec, RG: 24	SI: 32768
Date: 09-Mar-2023	Solvent: dmso	SW: 8000 Hz	TE: 298 K	AQ: 2.00 sec, RD: 0.00 sec	

lv18_C13

11d

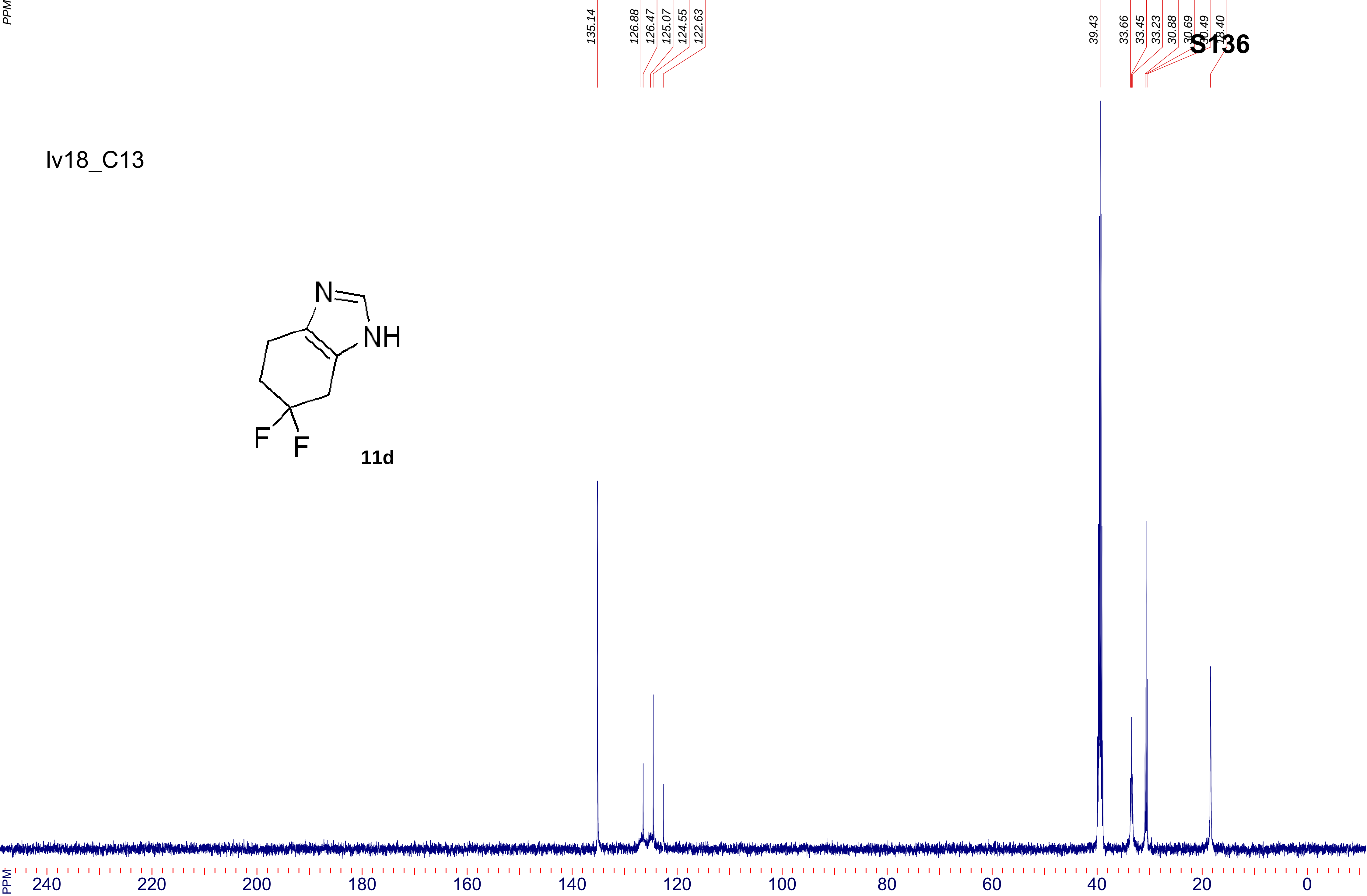

**File name: lv18_C13**	Operator: root	SF: 125.6429 MHz	NSC: 284	PW: 0.00 usec, RG: 51200	SI: 131072
Date: 01-Apr-2023	Solvent: DMSO	SW: 32680 Hz	TE: 683 K	AQ: 1.00 sec, RD: 0.00 sec	

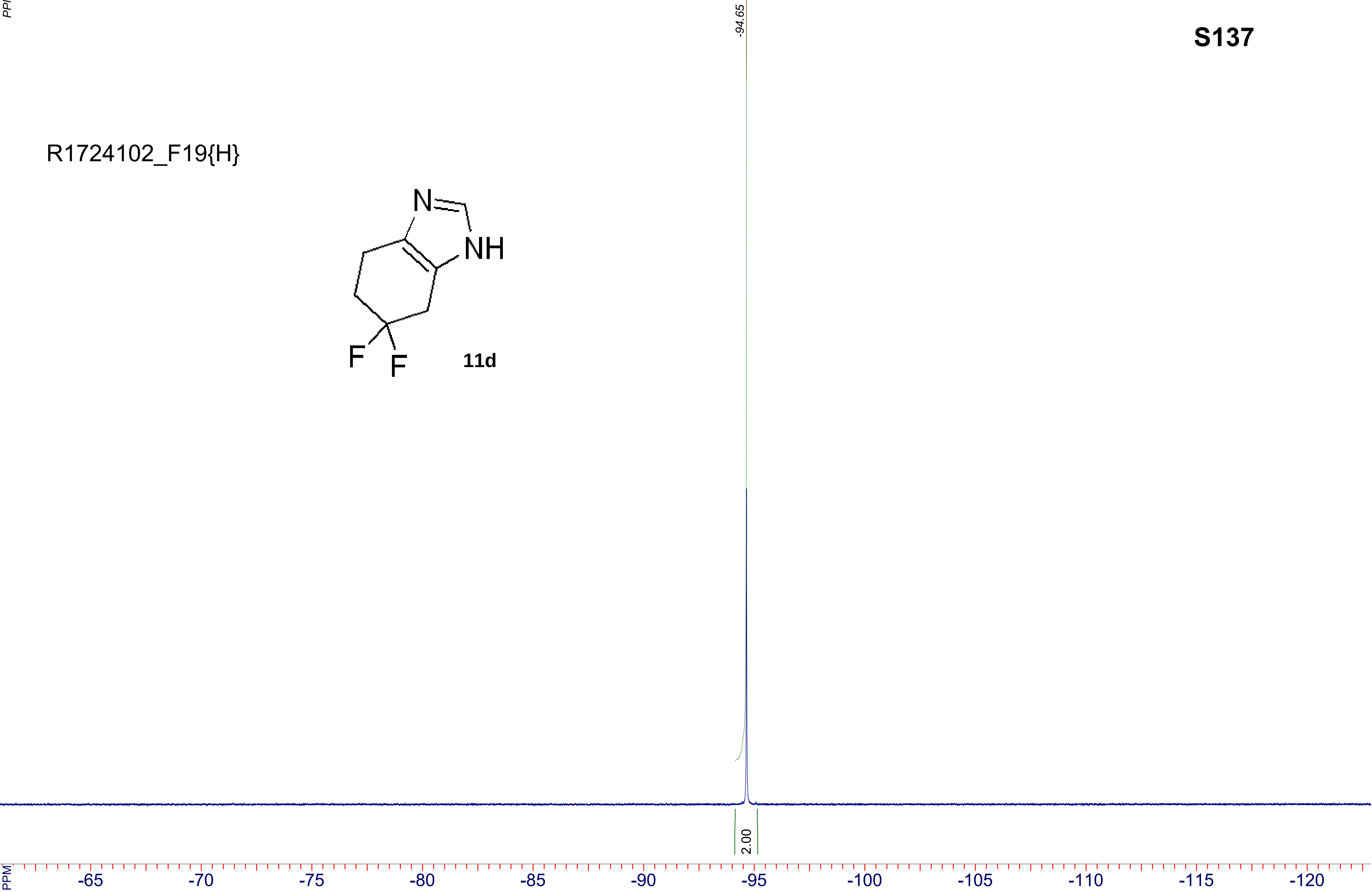

**File name: R1724102_F19{H}**	Operator: nmr	SF: 376.4986 MHz	NSC: 4	PW: 0.00 usec, RG: 2050	SI: 262144
Date: 16-Apr-2020	Solvent: DMSO	SW: 138889 Hz	TE: 297 K	AQ: 0.94 sec, RD: 0.00 sec	19F-{1H}

Target Compound Screening Report

**Data File**	23c.d	**Sample Name**	18
**Sample Type**	Sample	**Position**	P1-C5
**Instrument Name**	Instrument 1	**User Name**	Denis V.Bylina
**Acq Method**	Fast_Gradient_HRMS_pos_Lock_01312023.m	**Acquired Time**	4/7/2023 4:57:02 PM (UTC+03:00)
**IRM Calibration Status**	Success	**DA Method**	1.m
**Comment**	Lysenko		
**Sample Group**		**Info.**	Agilent 6224 TOF LC/MS
**MFC**	C7H8F2N2	**Stream Name**	LC 1
**Acquisition Time (Local)**	4/7/2023 4:57:02 PM (UTC+03:00)	**Acquisition SW Version**	6200 series TOF/6500 series Q-TOF B.08.00 (B8058.0)
**TOF Driver Version**	8.00.00	**TOF Firmware Version**	8.643
**Tune Mass Range Max.**	1700		

Compound Table

Label	Tgt Score	Mass Error (ppm)	Tgt Formula	Obs. RT	Ref. Mass	Obs. Mass
Cpd 1: C7 H8 F2 N2; 2.224	87.75	-0.94	C7 H8 F2 N2	2.224	158.0656	158.0654

Obs. *m/z*	Obs. RT	Obs. Mass	Tgt Formula	Tgt Mass	Tgt Mass Error (ppm)	RT Diff.	Find Cpds Algorithm
159.0727	2.224	158.0654	C7 H8 F2 N2	158.0656	-0.94	Find By Formula	

Compound Chromatograms

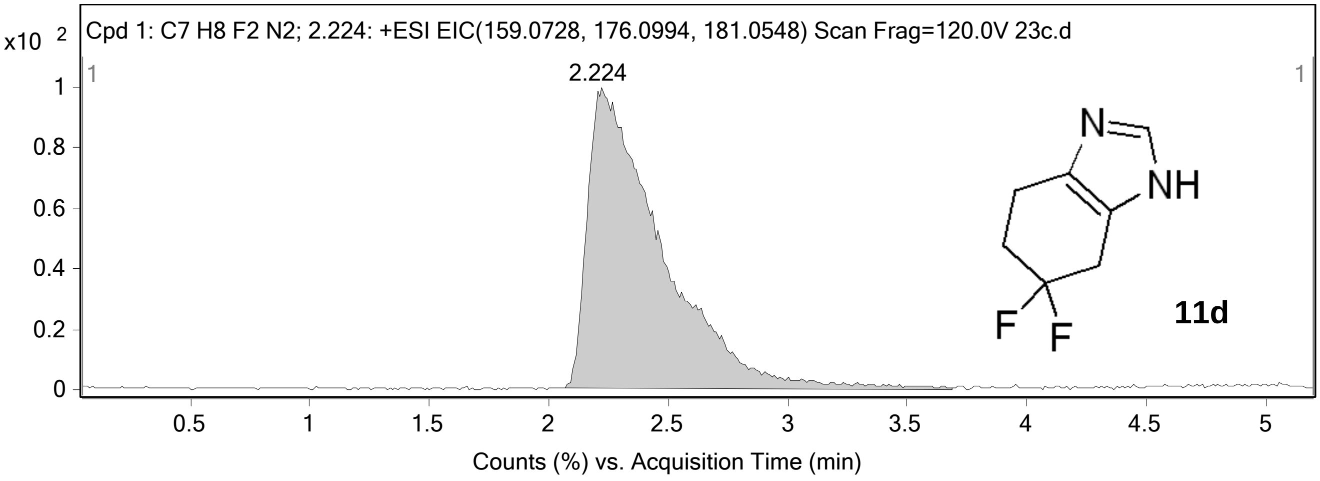

MS Zoomed Spectrum

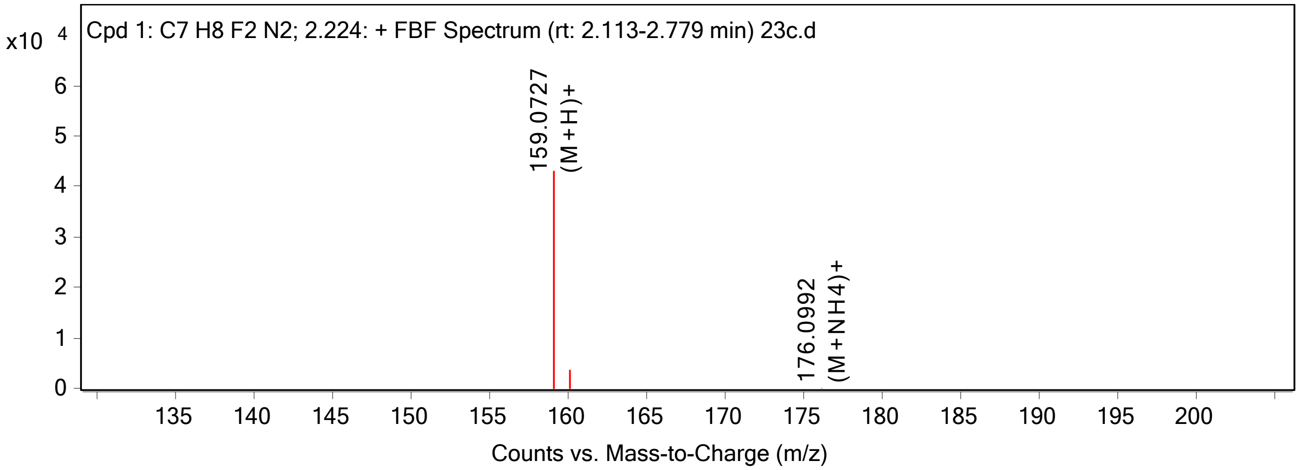

MS Spectrum Peak List

Obs. *m/z*	Charge	Abund	Ion/Isotope
159.0727	1	42920.29	(M+H)+
160.0758	1	3582.2	(M+H)+
176.0992	1	126.95	(M+NH4)+

MS Zoomed Spectrum

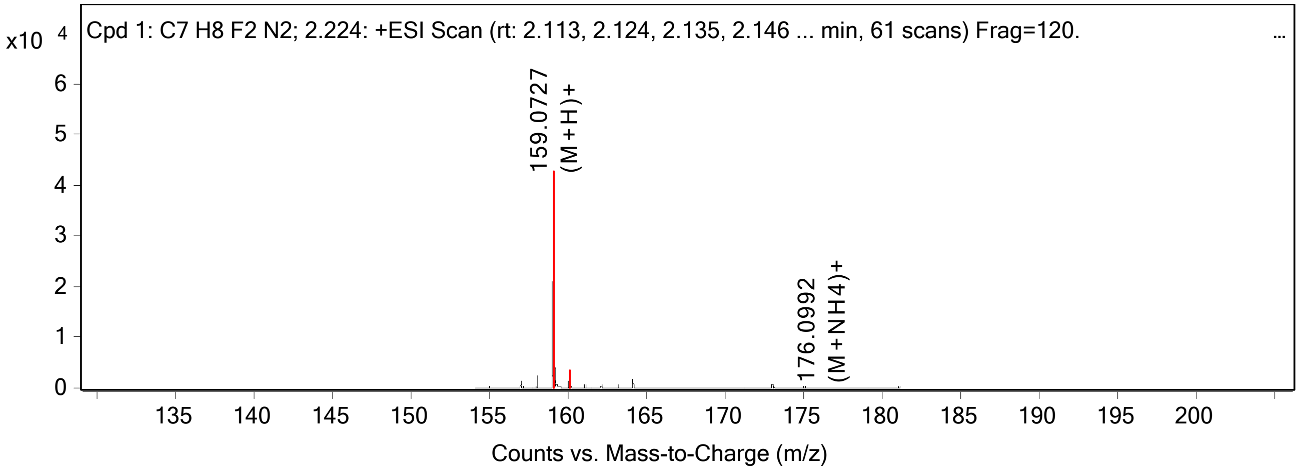

MS Spectrum Peak List

Obs. *m/z*	Charge	Abund	Ion/Isotope	Tgt Mass Error (ppm)
159.0727	1	42920.29	(M+H)+	1.08
160.0758	1	3582.2	(M+H)+	-0.92
176.0992	1	126.95	(M+NH4)+	1.28

--- End Of Report ---

S140

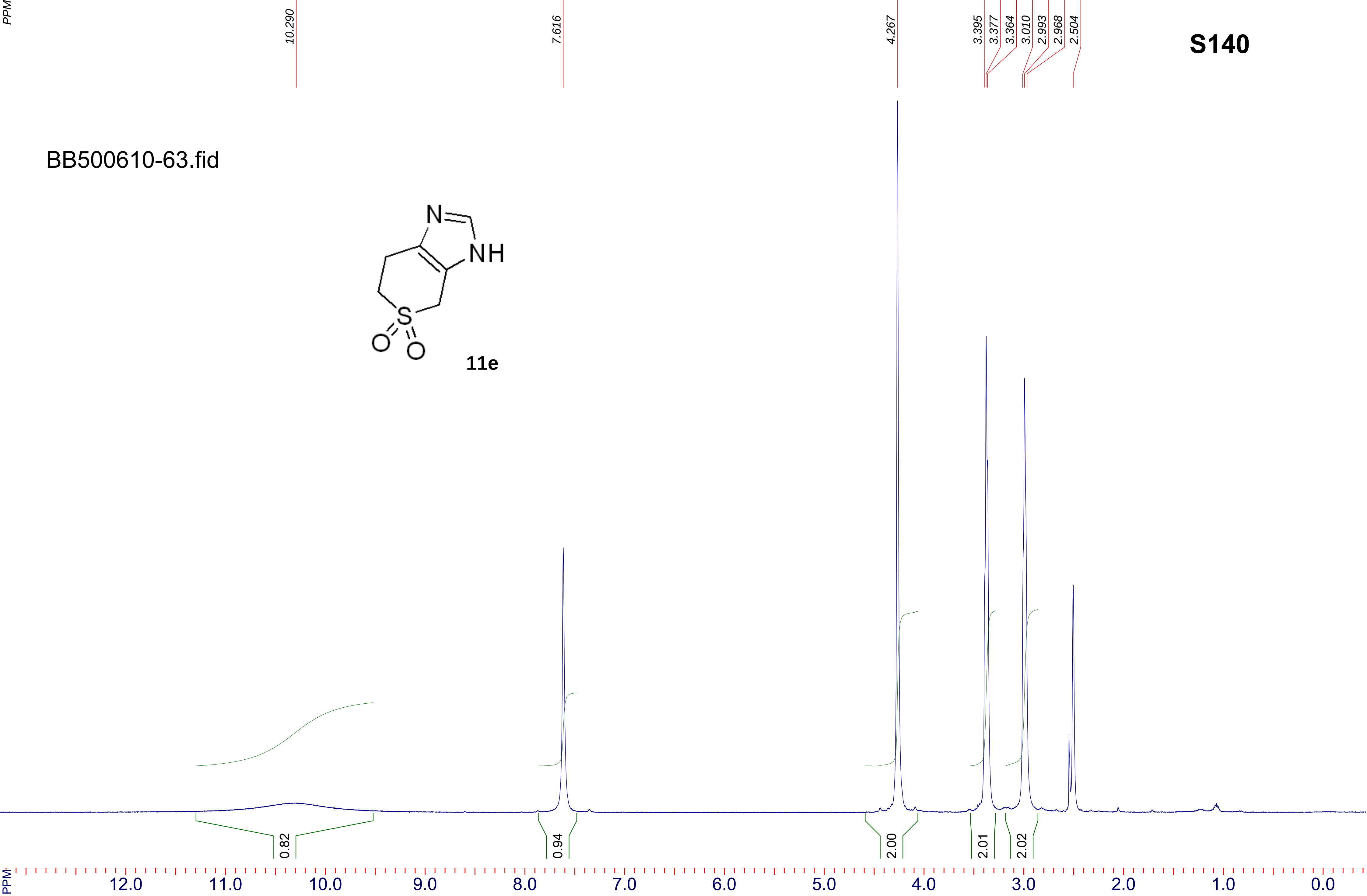

**File name: BB500610-63.fid**	Operator:	SF: 399.9753 MHz	NSC: 0	PW: 10.90 usec, RG: 20	SI: 32768
Date: 22-Jun-2023	Solvent: dmso	SW: 8000 Hz	TE: 298 K	AQ: 2.00 sec, RD: 0.00 sec	

BB500610-63_C13

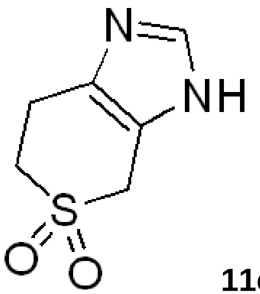

11e

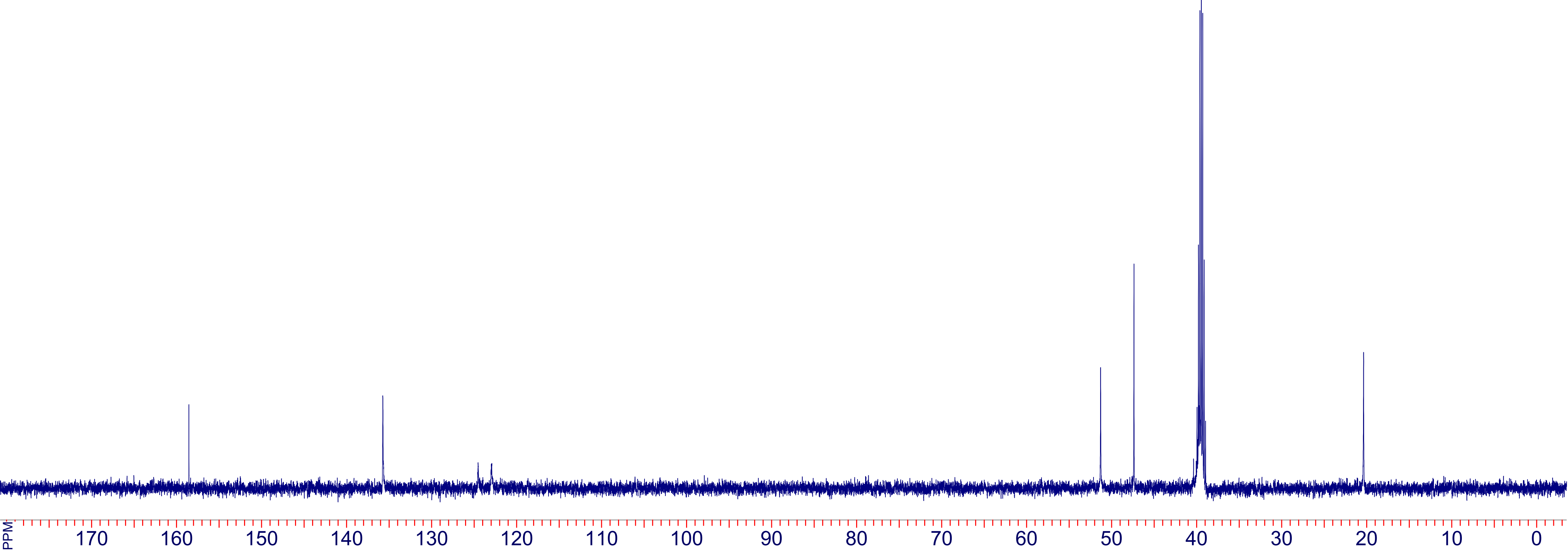

File name: BB500610-63_C13	Operator: root	SF: 125.6429 MHz	NSC: 87	PW: 0.00 usec, RG: 51200	SI: 131072
Date: 22-Jun-2023	Solvent: DMSO	SW: 32680 Hz	TE: 683 K	AQ: 0.78 sec, RD: 0.00 sec	

Target Compound Screening Report

**Data File**	13.d	**Sample Name**	28
**Sample Type**	Sample	**Position**	P1-B4
**Instrument Name**	Instrument 1	**User Name**	Denis V.Bylina
**Acq Method**	Fast_Gradient_HRMS_pos_Lock_01312023.m	**Acquired Time**	7/10/2023 10:59:35 AM (UTC+03:00)
**IRM Calibration Status**	Success	**DA Method**	1.m
**Comment**	Lysenko		
**Sample Group**		**Info.**	Agilent 6224 TOF LC/MS
**MFC**	C6H8N2O2S	**Stream Name**	LC 1
**Acquisition Time (Local)**	7/10/2023 10:59:35 AM (UTC+03:00)	**Acquisition SW Version**	6200 series TOF/6500 series Q-TOF B.08.00 (B8058.0)
**TOF Driver Version**	8.00.00	**TOF Firmware Version**	8.643
**Tune Mass Range Max.**	1700		

Compound Table

Label	Tgt Score	Mass Error (ppm)	Tgt Formula	Obs. RT	Ref. Mass	Obs. Mass
Cpd 1: C6 H8 N2 O2 S; 0.349	99.63	0.6	C6 H8 N2 O2 S	0.349	172.03065	172.03075

Obs. *m/z*	Obs. RT	Obs. Mass	Tgt Formula	Tgt Mass	Tgt Mass Error (ppm)	RT Diff.	Find Cpds Algorithm
173.03808	0.349	172.03075	C6 H8 N2 O2 S	172.03065	0.6	Find By Formula	

Compound Chromatograms

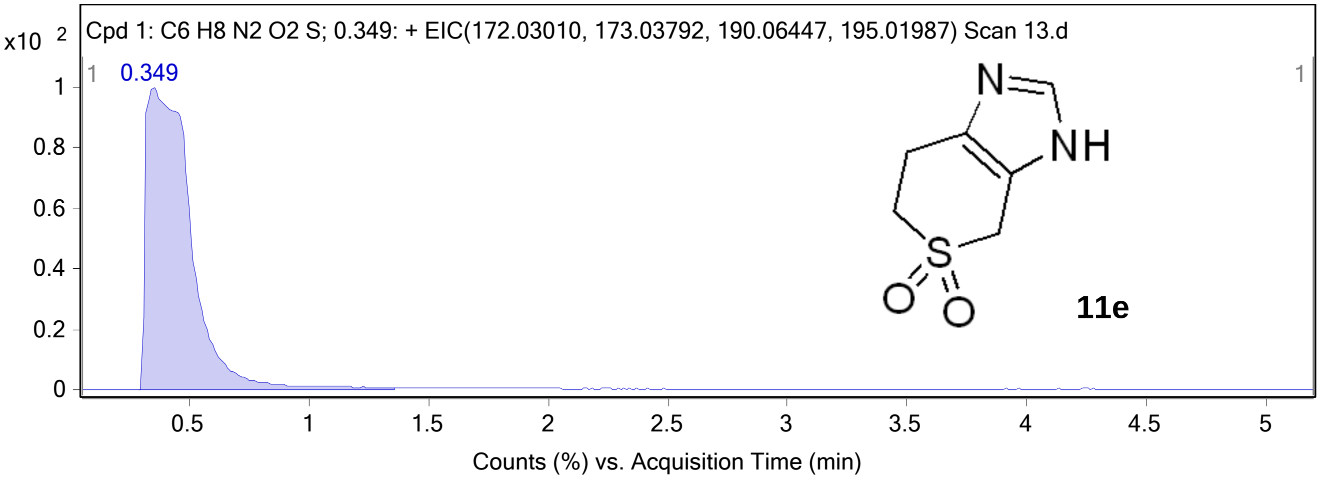

MS Zoomed Spectrum

Target Compound Screening Report

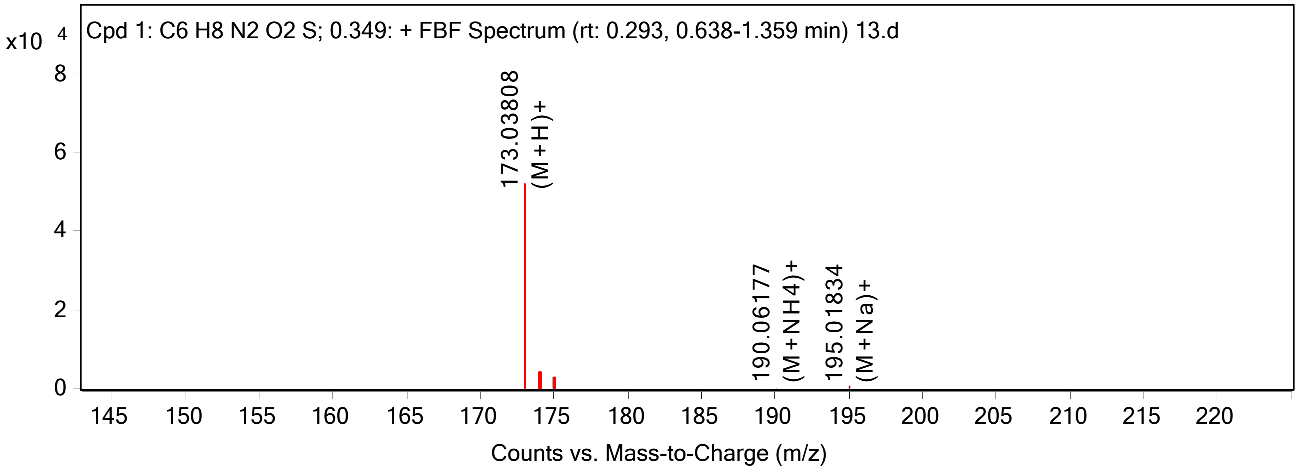

MS Spectrum Peak List

Obs. *m/z*	Charge	Abund	Ion/Isotope
173.03808	1	52058.83	(M+H)+
174.04025	1	4151.36	(M+H)+
175.0348	1	2431.22	(M+H)+
190.06177	1	218.84	(M+NH4)+
195.01834	1	308.95	(M+Na)+

MS Zoomed Spectrum

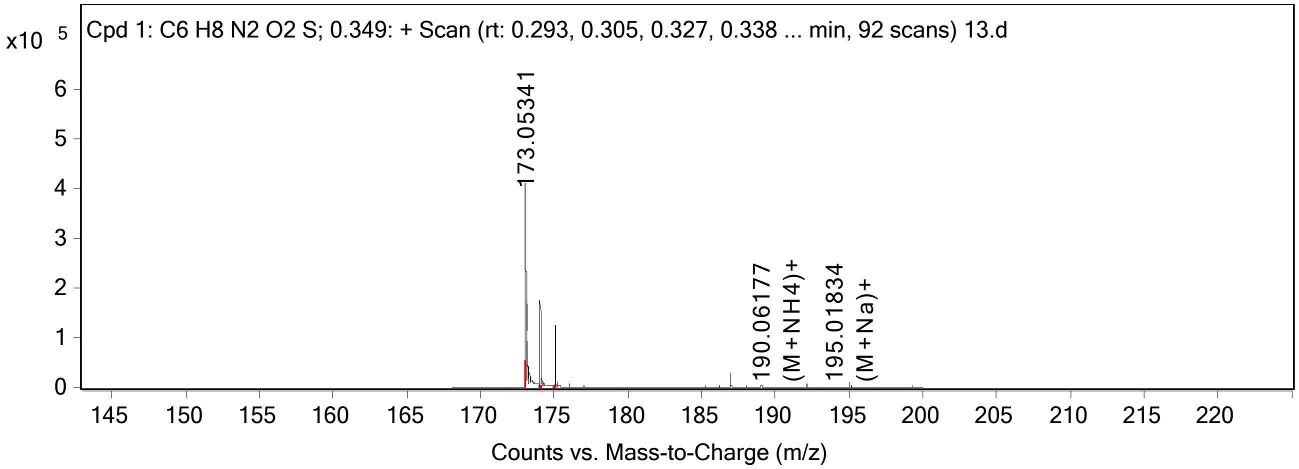

MS Spectrum Peak List

Obs. *m/z*	Charge	Abund	Ion/Isotope	Tgt Mass Error (ppm)
173.03808	1	52058.83	(M+H)+	-0.89
173.05341		415214.37		
174.04025	1	4151.36	(M+H)+	0.76
175.0348	1	2431.22	(M+H)+	0.62
190.06177	1	218.84	(M+NH4)+	14.25
195.01834	1	308.95	(M+Na)+	7.83

--- End Of Report ---

S144

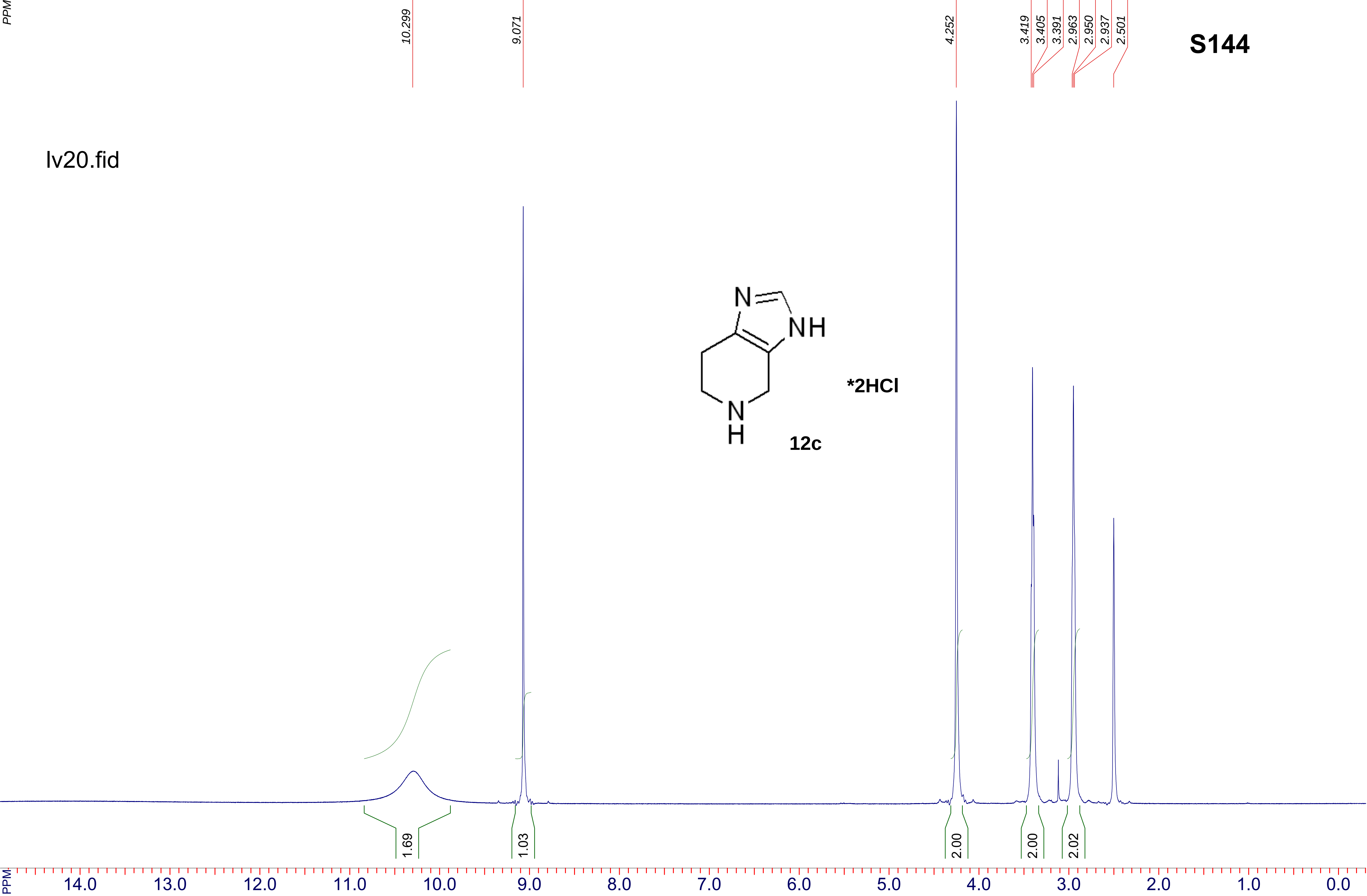

**File name: lv20.fid**	Operator:	SF: 399.9733 MHz	NSC: 0	PW: 10.90 usec, RG: 24	SI: 32768
Date: 08-Dec-2022	Solvent: dmso	SW: 8000 Hz	TE: 298 K	AQ: 2.00 sec, RD: 0.00 sec	

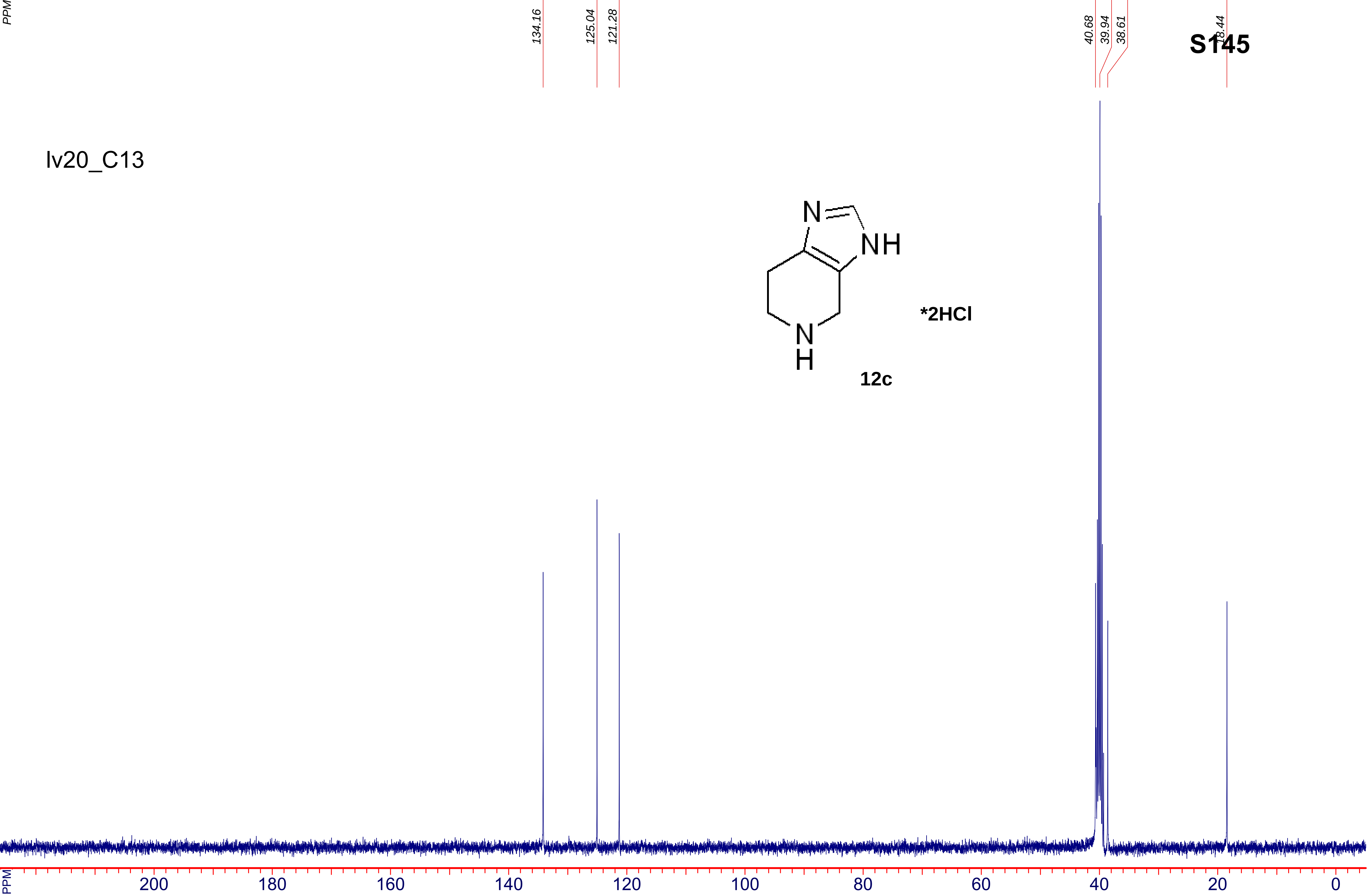

**File name: lv20_C13**	Operator: nmr	SF: 100.6128 MHz	NSC: 353	PW: 0.00 usec, RG: 2050	SI: 32768
Date: 10-Dec-2022	Solvent: DMSO	SW: 26042 Hz	TE: 300 K	AQ: 0.98 sec, RD: 0.00 sec	

Target Compound Screening Report

**Data File**	24a1.d	**Sample Name**	20
**Sample Type**	Sample	**Position**	P1-C6
**Instrument Name**	Instrument 1	**User Name**	Denis V.Bylina
**Acq Method**	Fast_Gradient_HRMS_pos_Lock_01312023.m	**Acquired Time**	4/7/2023 2:36:37 PM (UTC+03:00)
**IRM Calibration Status**	Success	**DA Method**	1.m
**Comment**	Lysenko		
**Sample Group**		**Info.**	Agilent 6224 TOF LC/MS
**MFC**	C6H9N3	**Stream Name**	LC 1
**Acquisition Time (Local)**	4/7/2023 2:36:37 PM (UTC+03:00)	**Acquisition SW Version**	6200 series TOF/6500 series Q-TOF B.08.00 (B8058.0)
**TOF Driver Version**	8.00.00	**TOF Firmware Version**	8.643
**Tune Mass Range Max.**	1700		

Compound Table

Label	Tgt Score	Mass Error (ppm)	Tgt Formula	Obs. RT	Ref. Mass	Obs. Mass
Cpd 1: C6 H9 N3; 0.765	99.64	0.56	C6 H9 N3	0.765	123.0796	123.0797

Obs. *m/z*	Obs. RT	Obs. Mass	Tgt Formula	Tgt Mass	Tgt Mass Error (ppm)	RT Diff.	Find Cpds Algorithm
124.087	0.765	123.0797	C6 H9 N3	123.0796	0.56	Find By Formula	

Compound Chromatograms

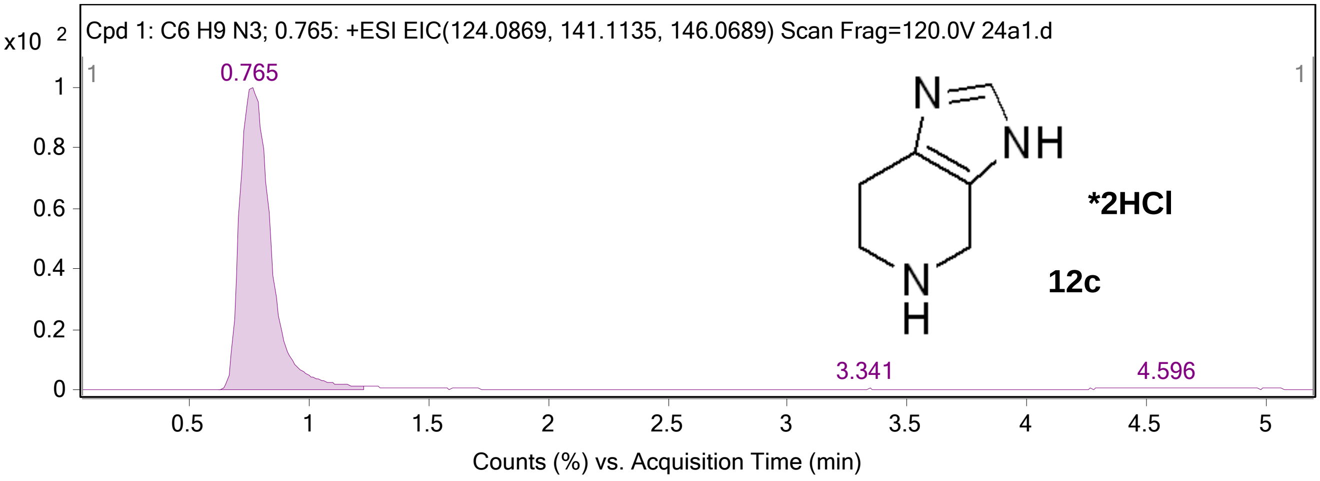

MS Zoomed Spectrum

Target Compound Screening Report

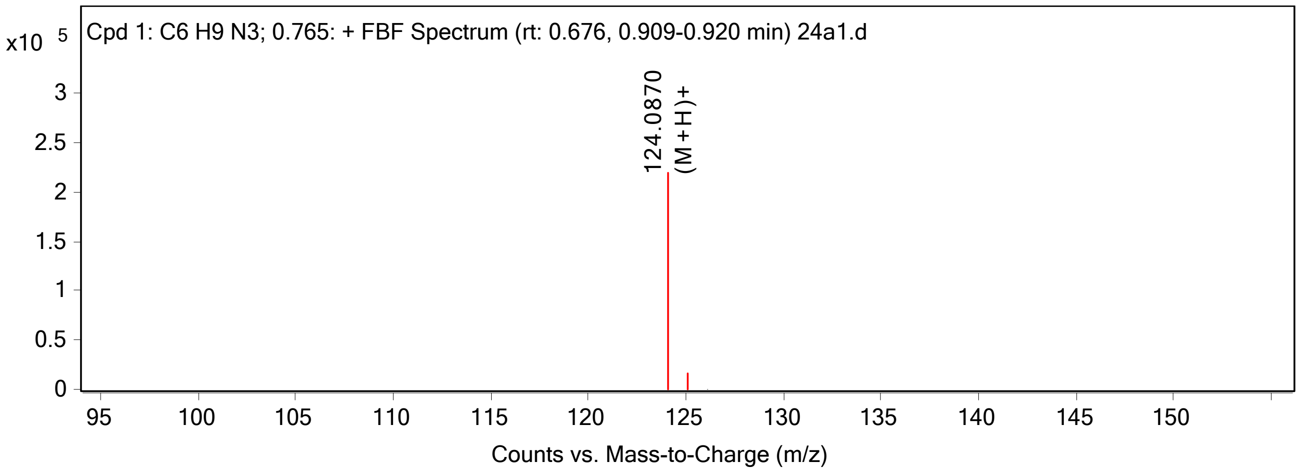

MS Spectrum Peak List

Obs. *m/z*	Charge	Abund	Ion/Isotope
124.087	1	219439.3	(M+H)+
125.0898	1	15303.28	(M+H)+
126.091	1	629.68	(M+H)+

MS Zoomed Spectrum

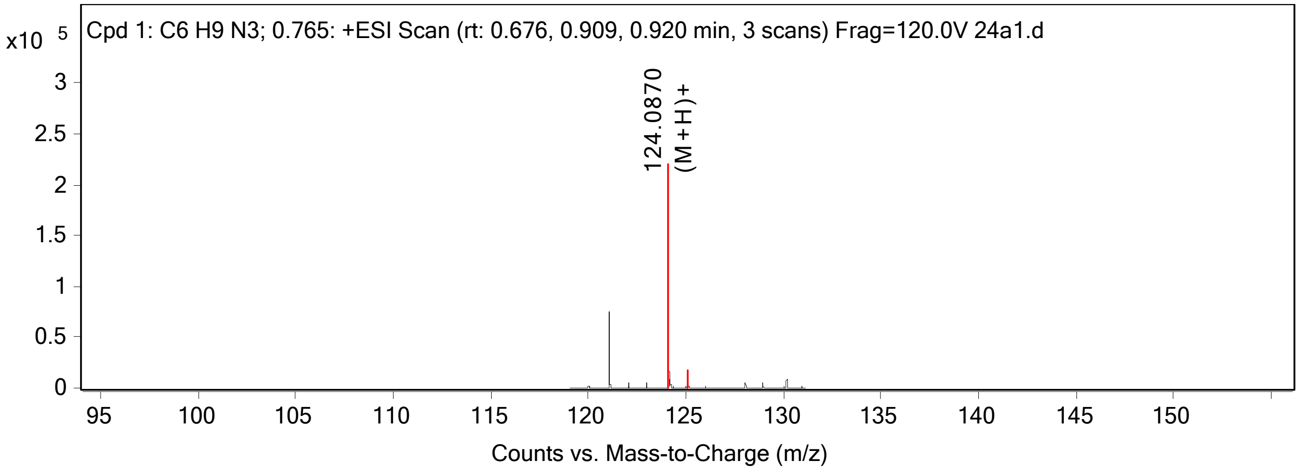

MS Spectrum Peak List

Obs. *m/z*	Charge	Abund	Ion/Isotope	Tgt Mass Error (ppm)
124.087	1	219439.29	(M+H)+	-0.42
125.0898	1	15303.28	(M+H)+	-2.84
126.091	1	629.68	(M+H)+	5.8

--- End Of Report ---

S148

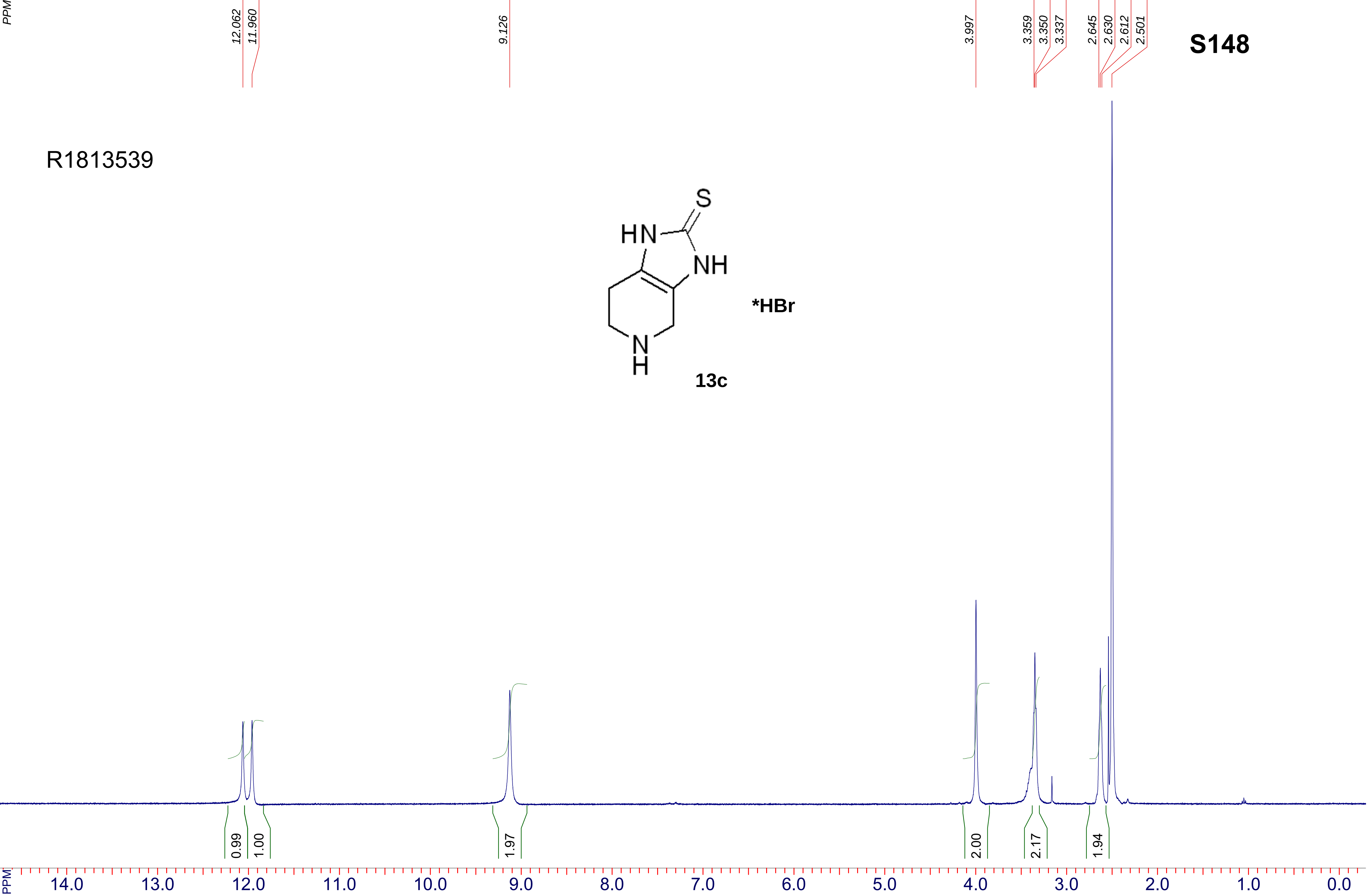

**File name: R1813539**	Operator:	SF: 399.9755 MHz	NSC: 0	PW: 11.60 usec, RG: 40	SI: 32768
Date: 24-Jul-2020	Solvent: dmso	SW: 7599 Hz	TE: 294 K	AQ: 2.11 sec, RD: 0.00 sec	

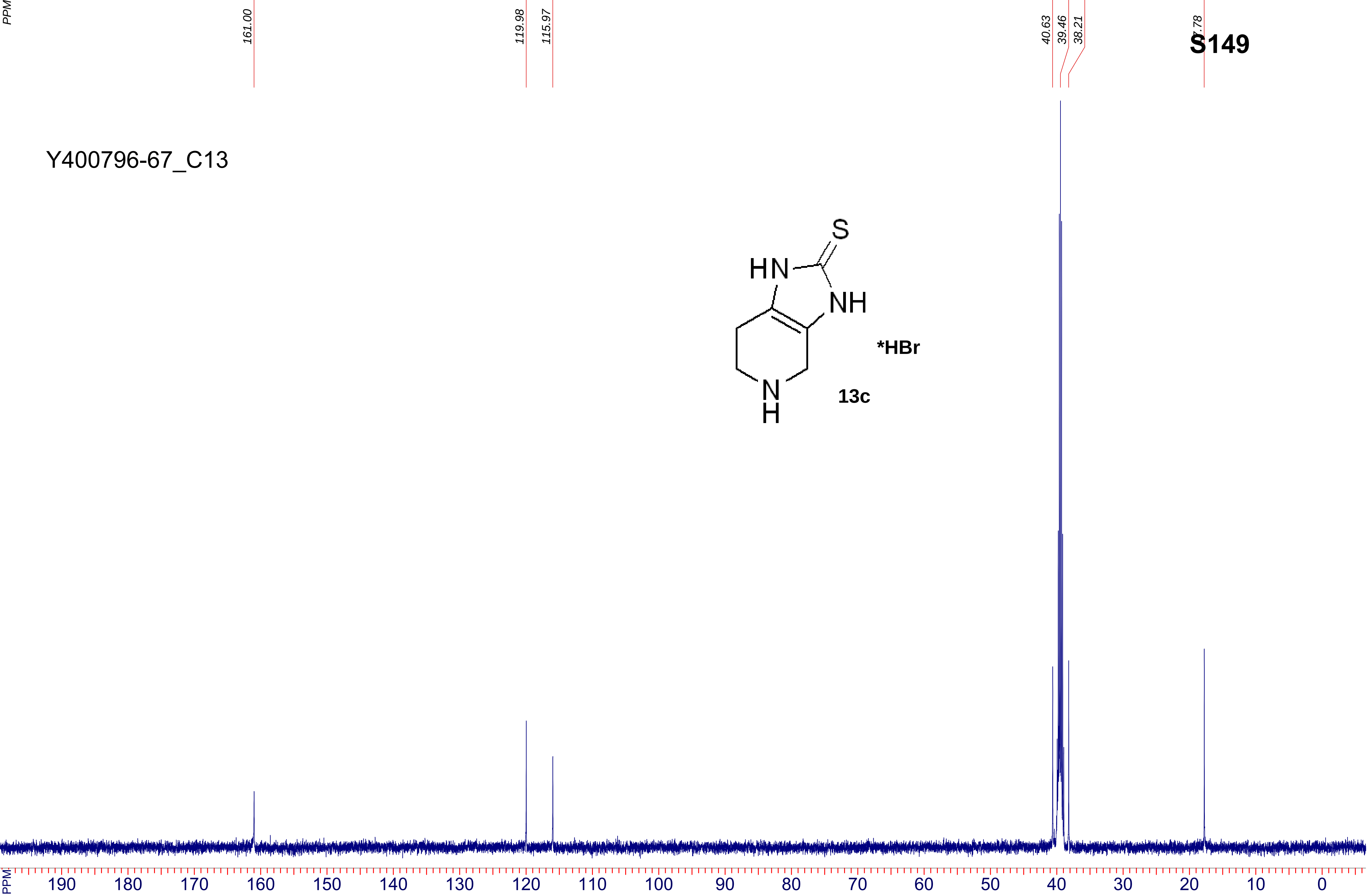

**File name: Y400796-67_C13**	Operator: root	SF: 125.6429 MHz	NSC: 300	PW: 0.00 usec, RG: 51200	SI: 131072
Date: 02-Jun-2023	Solvent: DMSO	SW: 32680 Hz	TE: 683 K	AQ: 0.78 sec, RD: 0.00 sec	

Target Compound Screening Report

**Data File**	20.d	**Sample Name**	R1813539
**Sample Type**	Sample	**Position**	P1-F4
**Instrument Name**	Instrument 1	**User Name**	Denis V.Bylina
**Acq Method**	Fast_Gradient_HRMS_pos_Lock_01312023.m	**Acquired Time**	6/5/2023 5:45:59 PM (UTC+03:00)
**IRM Calibration Status**	Success	**DA Method**	1.m
**Comment**	H000108		
**Sample Group**		**Info.**	Agilent 6224 TOF LC/MS
**MFC**	C6H9N3S	**Stream Name**	LC 1
**Acquisition Time (Local)**	6/5/2023 5:45:59 PM (UTC+03:00)	**Acquisition SW Version**	6200 series TOF/6500 series Q-TOF B.08.00 (B8058.0)
**TOF Driver Version**	8.00.00	**TOF Firmware Version**	8.643
**Tune Mass Range Max.**	1700		

Compound Table

Label	Tgt Score	Mass Error (ppm)	Tgt Formula	Obs. RT	Ref. Mass	Obs. Mass
Cpd 1: C6 H9 N3 S; 0.342	93.13	-0.29	C6 H9 N3 S	0.342	155.05172	155.05167

Obs. *m/z*	Obs. RT	Obs. Mass	Tgt Formula	Tgt Mass	Tgt Mass Error (ppm)	RT Diff.	Find Cpds Algorithm
156.05917	0.342	155.05167	C6 H9 N3 S	155.05172	-0.29	Find By Formula	

Compound Chromatograms

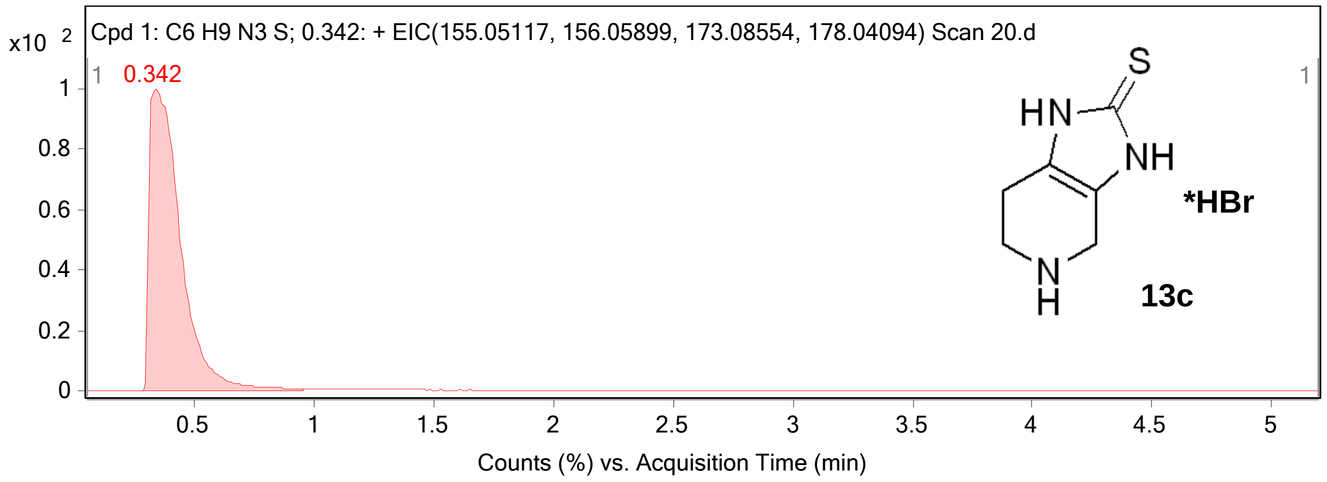

MS Zoomed Spectrum

Target Compound Screening Report

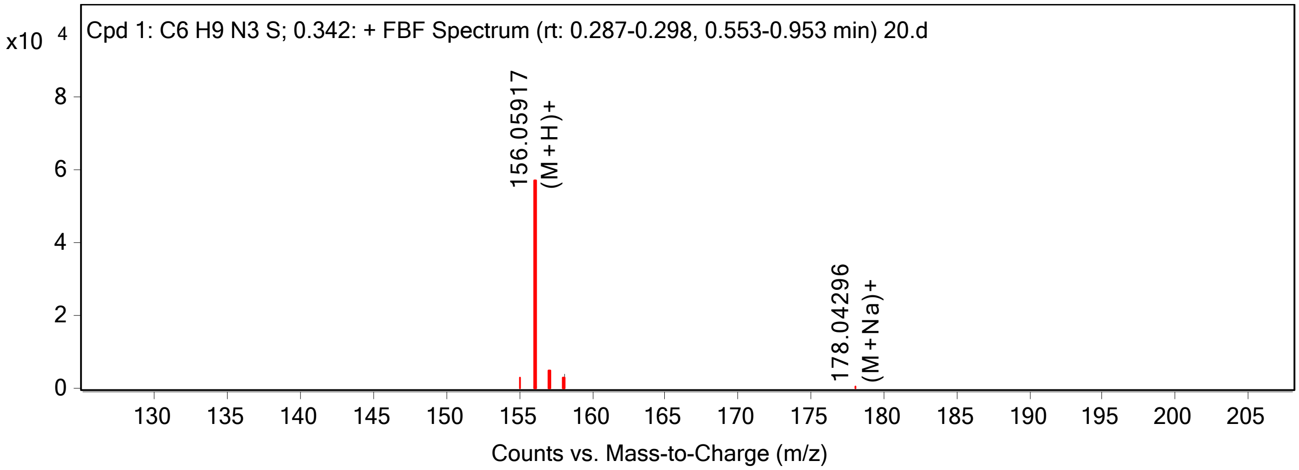

MS Spectrum Peak List

Obs. *m/z*	Charge	Abund	Ion/Isotope
155.0515	1	2894.15	M+
156.05917	1	56395.63	(M+H)+
157.06123	1	4537.97	(M+H)+
158.05137	1	3692.78	(M+H)+
178.04296	1	403.52	(M+Na)+

MS Zoomed Spectrum

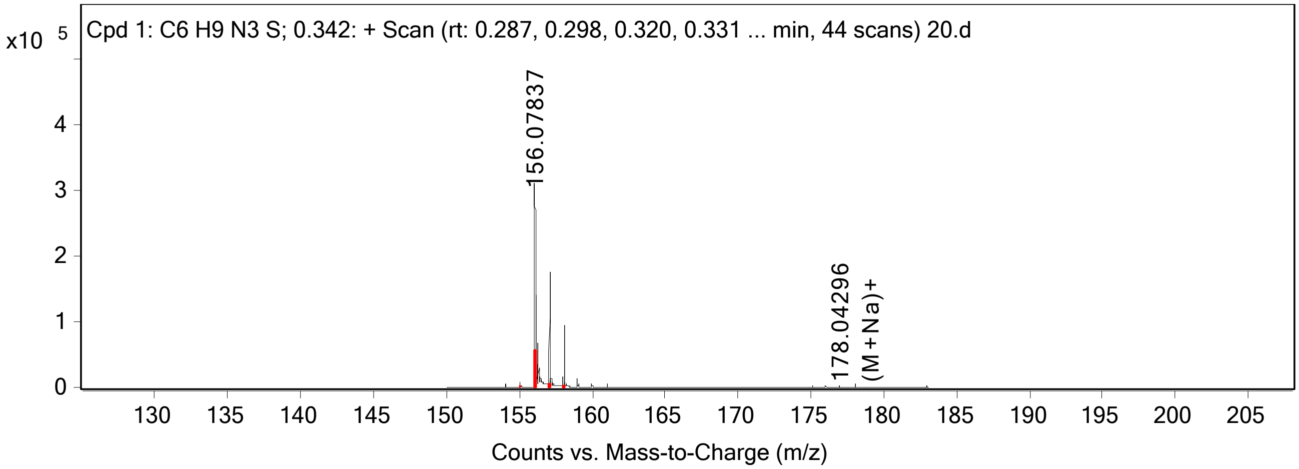

MS Spectrum Peak List

Obs. *m/z*	Charge	Abund	Ion/Isotope	Tgt Mass Error (ppm)
155.0515	1	2894.15	M+	-2.12
156.05917	1	56395.63	(M+H)+	-1.11
156.07837		311825.67		
157.06123	1	4537.97	(M+H)+	-0.15
158.05137	1	3692.78	(M+H)+	25.23
178.04296	1	403.52	(M+Na)+	-11.37

--- End Of Report ---

S152

20

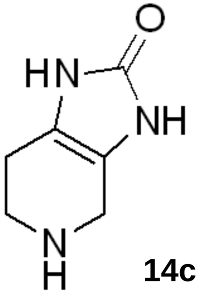

14c

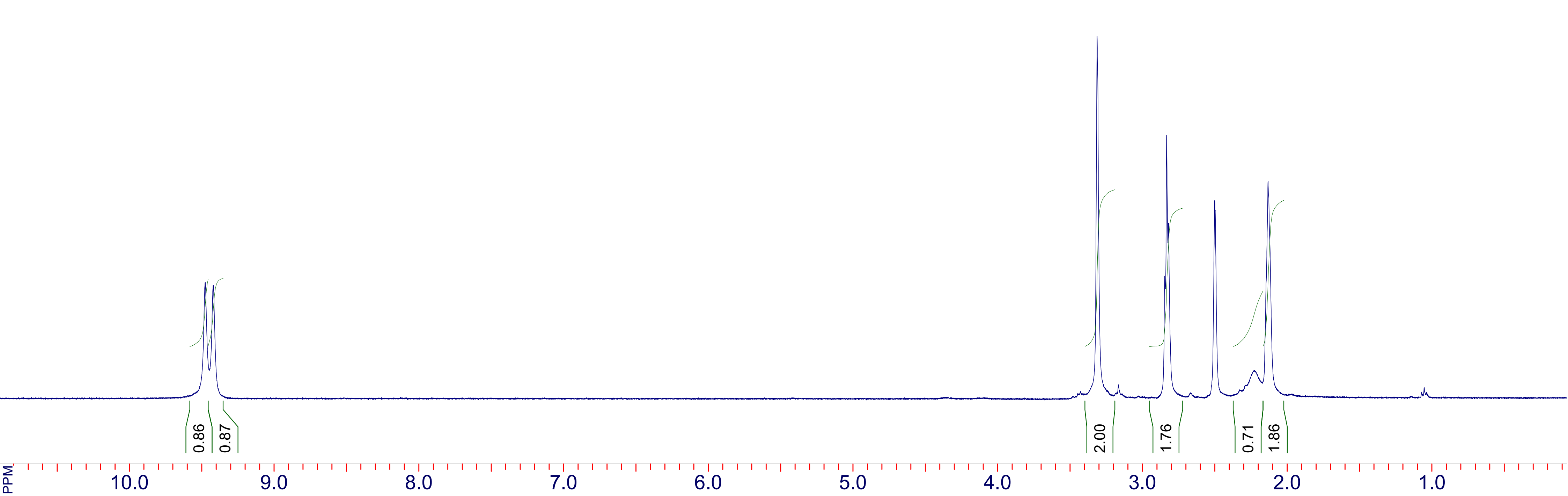

**File name: 20**	Operator:	SF: 399.9800 MHz	NSC: 0	PW: 11.30 usec, RG: 30	SI: 32768
Date: 18-Mar-2020	Solvent: dmso	SW: 7599 Hz	TE: 294 K	AQ: 2.16 sec, RD: 0.00 sec	

S153

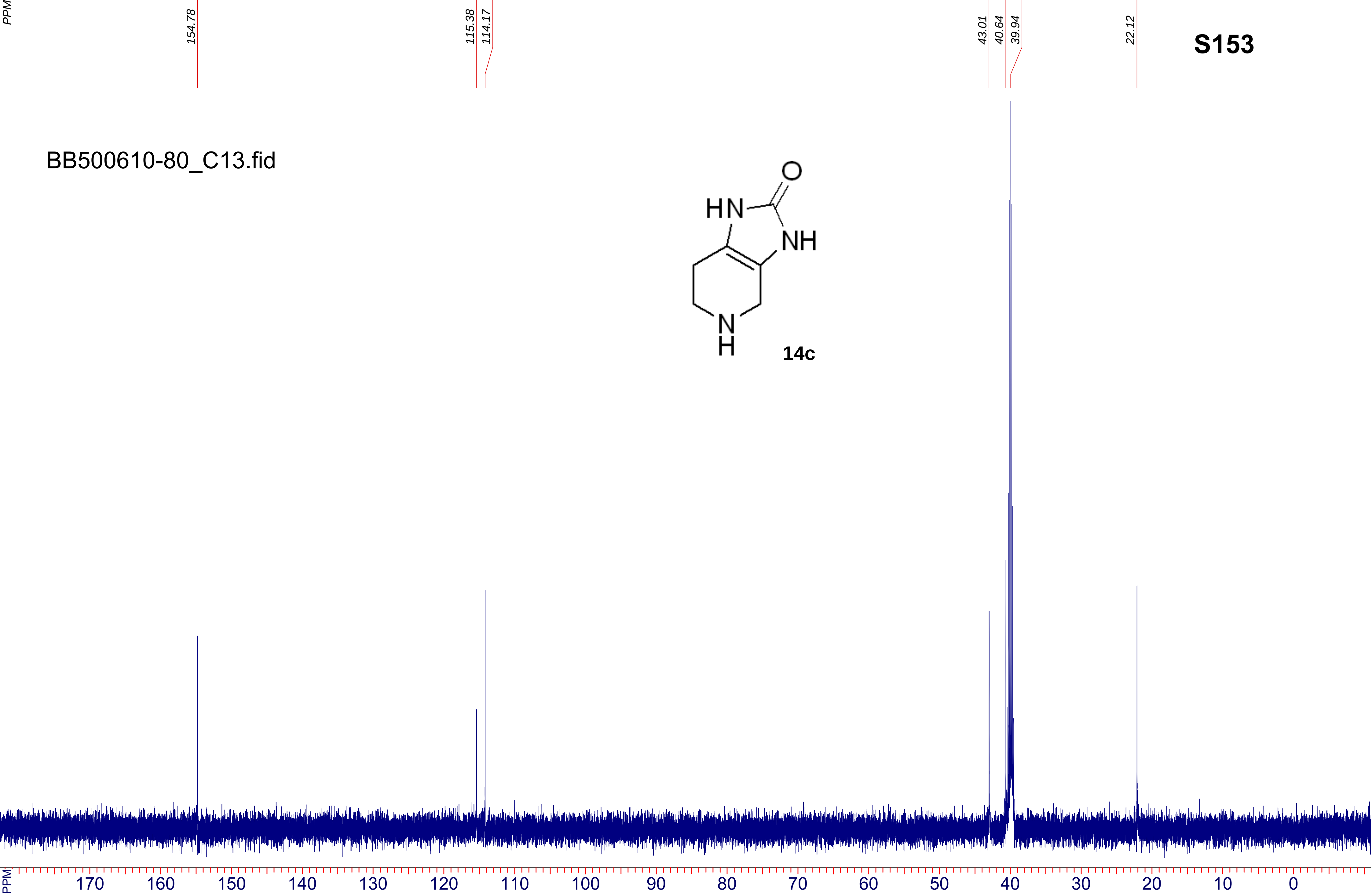

File name: BB500610-80_C13.fid	Operator:	SF: 150.8304 MHz	NSC: 0	PW: 3.09 usec, RG: 60	SI: 65536
Date: 23-Jun-2023	Solvent: dmso	SW: 39063 Hz	TE: 298 K	AQ: 0.84 sec, RD: 0.00 sec	

Target Compound Screening Report

**Data File**	26c.d	**Sample Name**	25
**Sample Type**	Sample	**Position**	P1-C8
**Instrument Name**	Instrument 1	**User Name**	Denis V.Bylina
**Acq Method**	Fast_Gradient_HRMS_pos_Lock_01312023.m	**Acquired Time**	4/7/2023 5:02:59 PM (UTC+03:00)
**IRM Calibration Status**	Success	**DA Method**	1.m
**Comment**	Lysenko		
**Sample Group**		**Info.**	Agilent 6224 TOF LC/MS
**MFC**	C6H9N3O	**Stream Name**	LC 1
**Acquisition Time (Local)**	4/7/2023 5:02:59 PM (UTC+03:00)	**Acquisition SW Version**	6200 series TOF/6500 series Q-TOF B.08.00 (B8058.0)
**TOF Driver Version**	8.00.00	**TOF Firmware Version**	8.643
**Tune Mass Range Max.**	1700		

Compound Table

Label	Tgt Score	Mass Error (ppm)	Tgt Formula	Obs. RT	Ref. Mass	Obs. Mass
Cpd 1: C6 H9 N3 O; 1.171	85.54	-1.14	C6 H9 N3 O	1.171	139.0746	139.0744

Obs. *m/z*	Obs. RT	Obs. Mass	Tgt Formula	Tgt Mass	Tgt Mass Error (ppm)	RT Diff.	Find Cpds Algorithm
140.0816	1.171	139.0744	C6 H9 N3 O	139.0746	-1.14	Find By Formula	

Compound Chromatograms

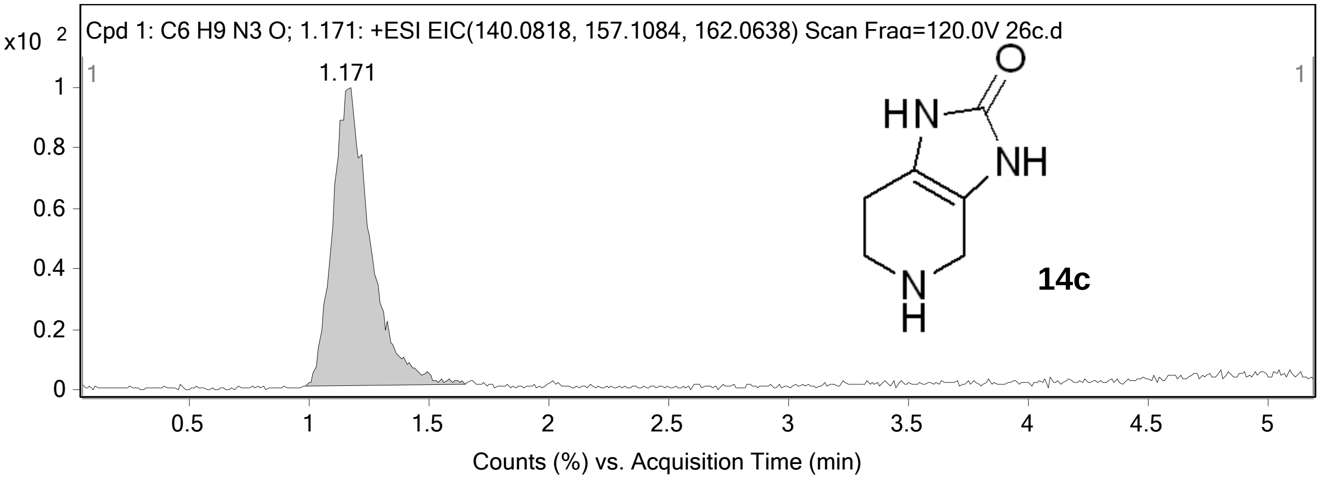

MS Zoomed Spectrum

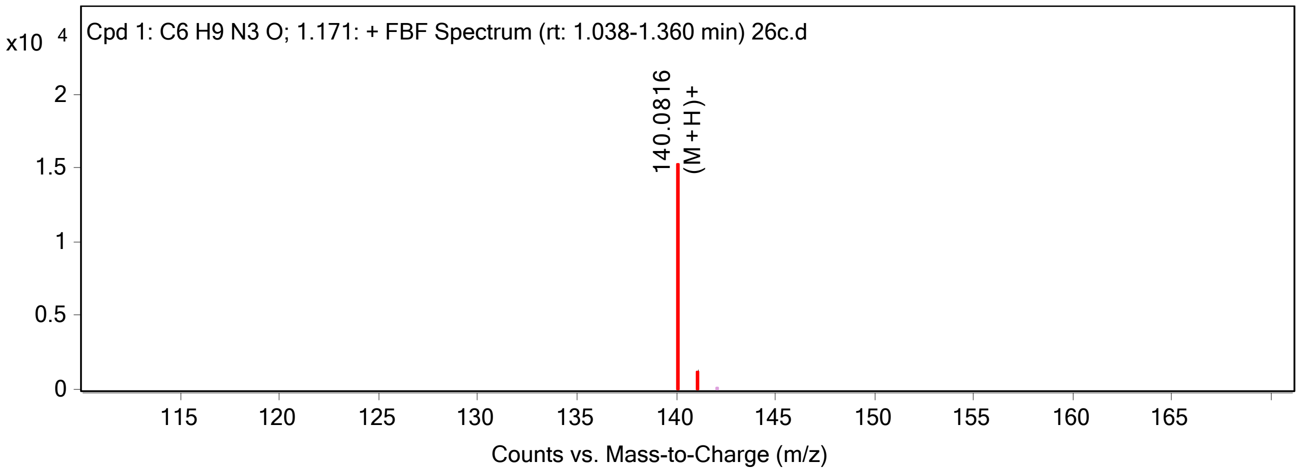

MS Spectrum Peak List

Obs. *m/z*	Charge	Abund	Ion/Isotope
140.0816	1	15101.71	(M+H)+
141.085	1	1383.06	(M+H)+

MS Zoomed Spectrum

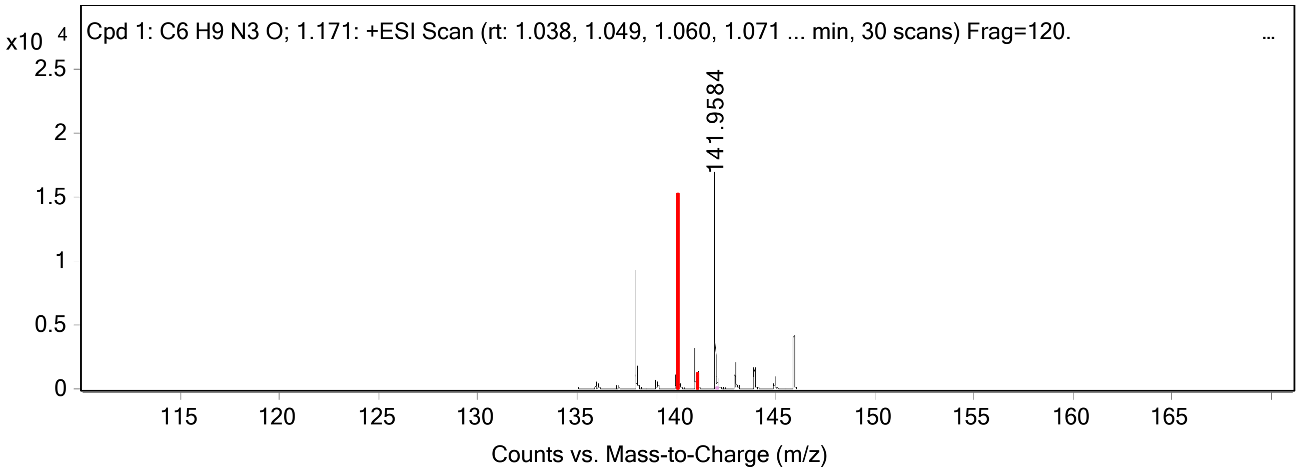

MS Spectrum Peak List

Obs. *m/z*	Charge	Abund	Ion/Isotope	Tgt Mass Error (ppm)
140.0816	1	15101.71	(M+H)+	1.65
141.085	1	1383.06	(M+H)+	-4.48
141.9584		17204.16		

--- End Of Report ---

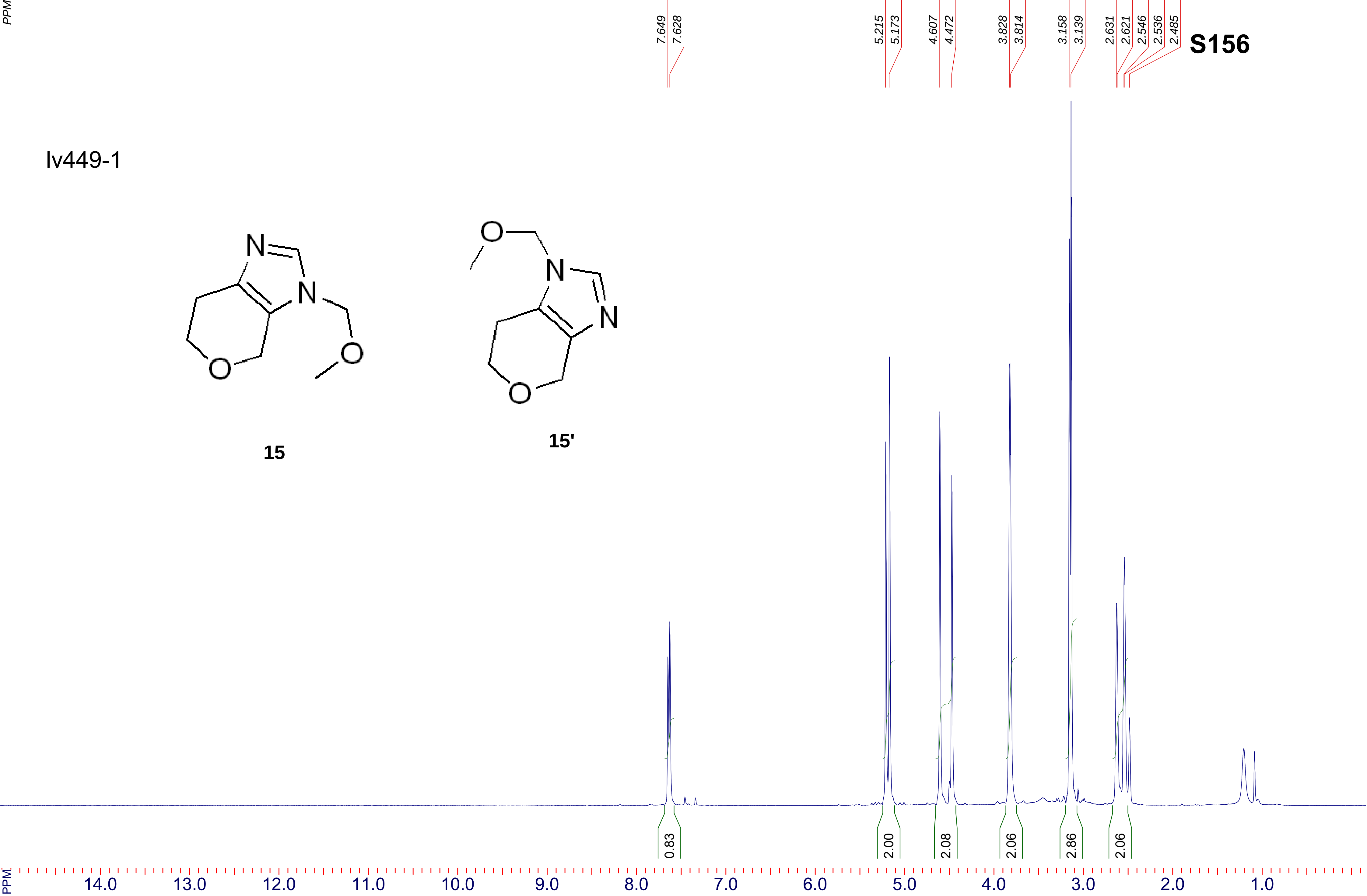

**File name: lv449-1**	Operator: root	SF: 499.7730 MHz	NSC: 1	PW: 0.00 usec, RG: 22	SI: 32768
Date: 02-Sep-2022	Solvent: DMSO	SW: 10776 Hz	TE: 683 K	AQ: 1.52 sec, RD: 0.00 sec	

lv-449-1_C13

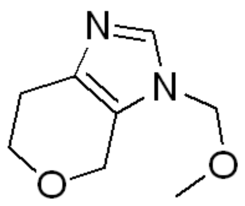

15

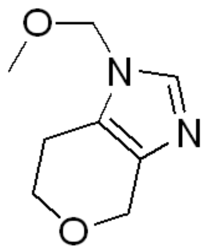

15'

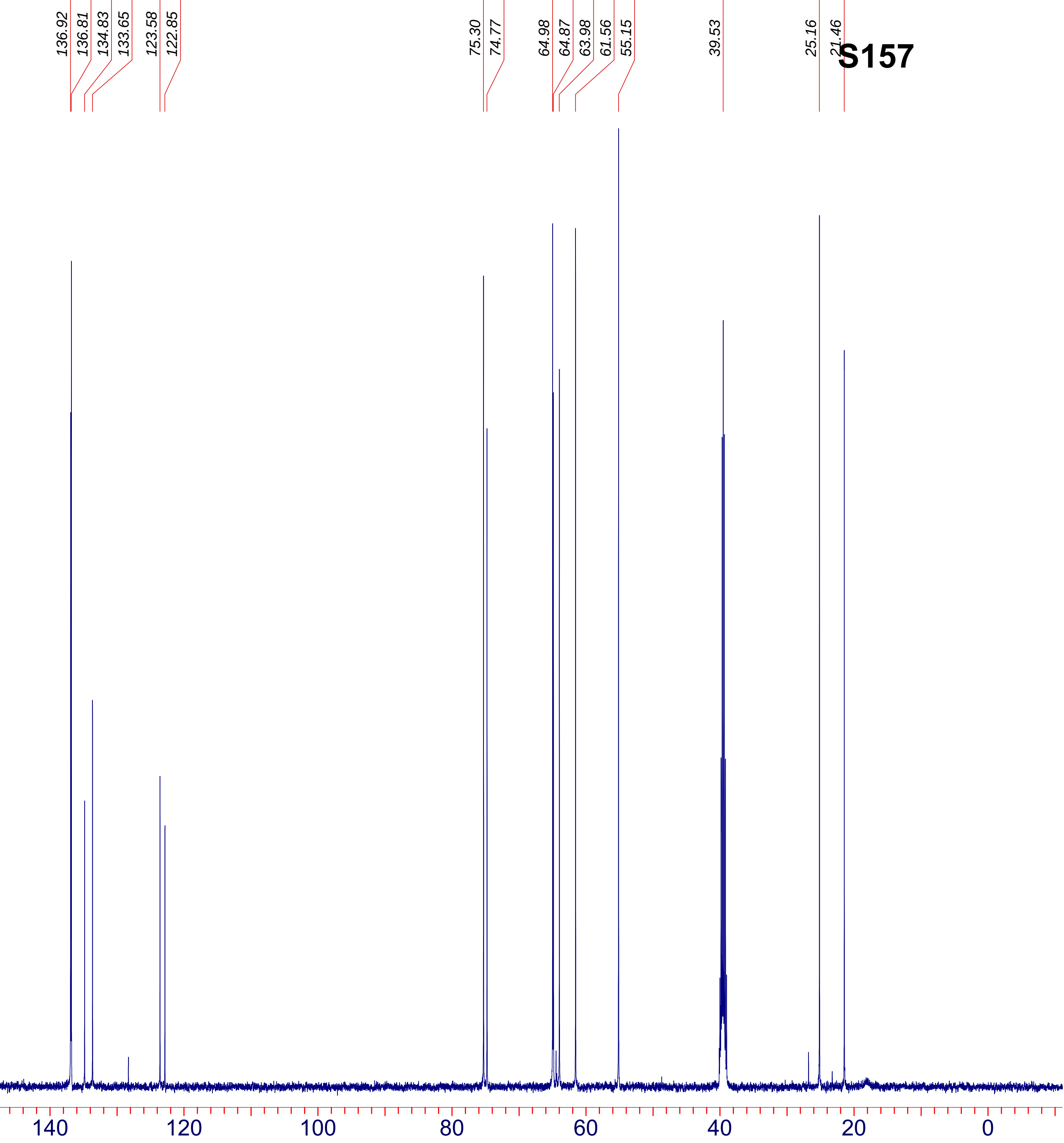

**File name: lv-449-1_C13**	Operator: root	SF: 125.6681 MHz	NSC: 500	PW: 0.00 usec, RG: 51200	SI: 131072
Date: 03-Sep-2022	Solvent: DMSO	SW: 32680 Hz	TE: 683 K	AQ: 0.78 sec, RD: 0.00 sec	

lv-449-1_C13APT

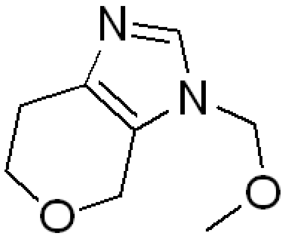

15

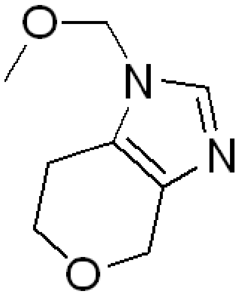

15'

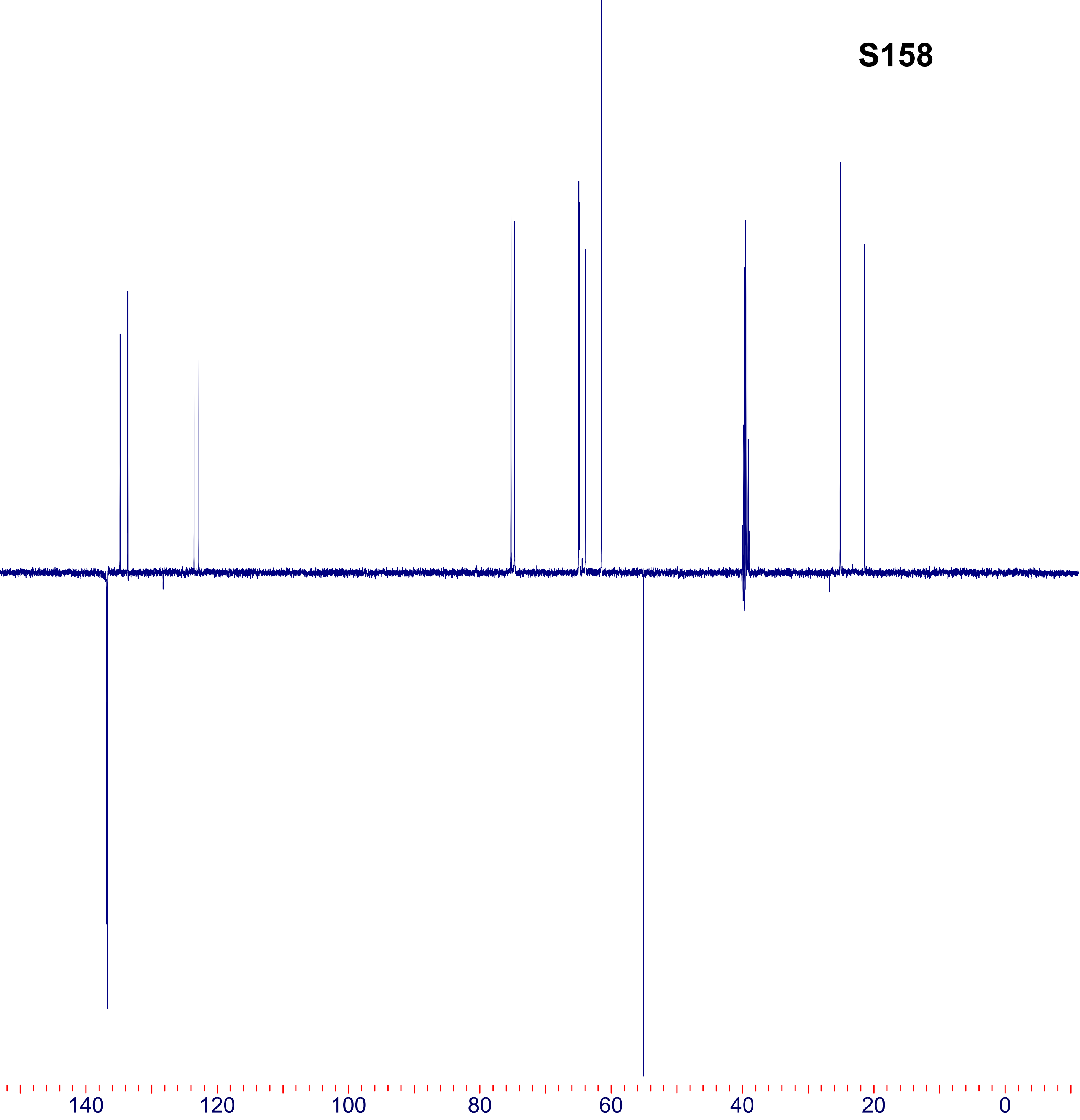

File name: lv-449-1_C13APT	Operator: root	SF: 125.6681 MHz	NSC: 358	PW: 0.00 usec, RG: 51200	SI: 65536
Date: 03-Sep-2022	Solvent: DMSO	SW: 32680 Hz	TE: 683 K	AQ: 1.57 sec, RD: 0.00 sec	

MaxPeak: 54.56%
Ret_Time: 0.582 min

CLQ320025

S159

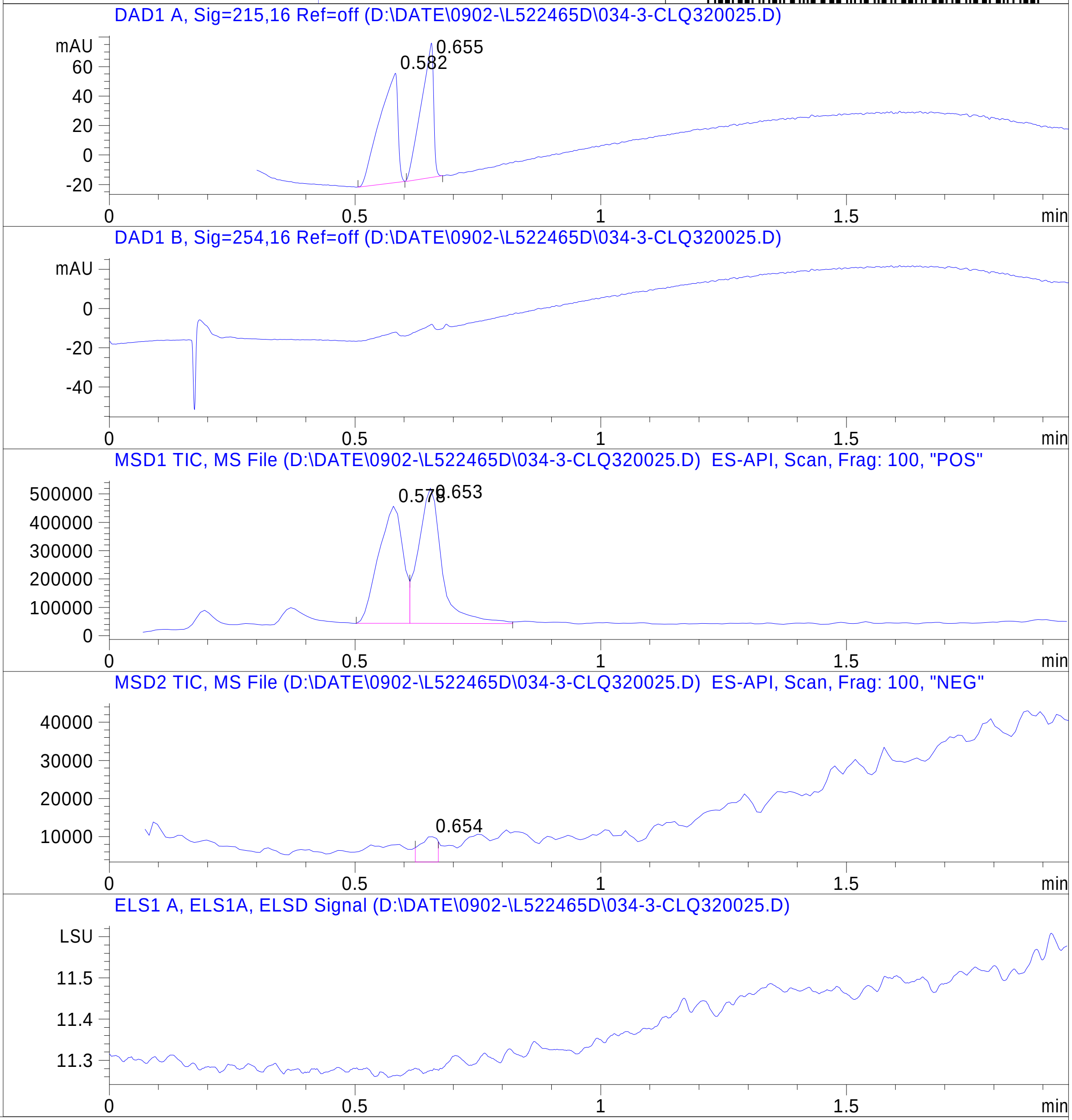

Mol Wt 0

Exact Mass

#	Time	Area%
1	0.582	54.56
2	0.655	45.44

RT 0.578

RT 0.653

RT 0.654

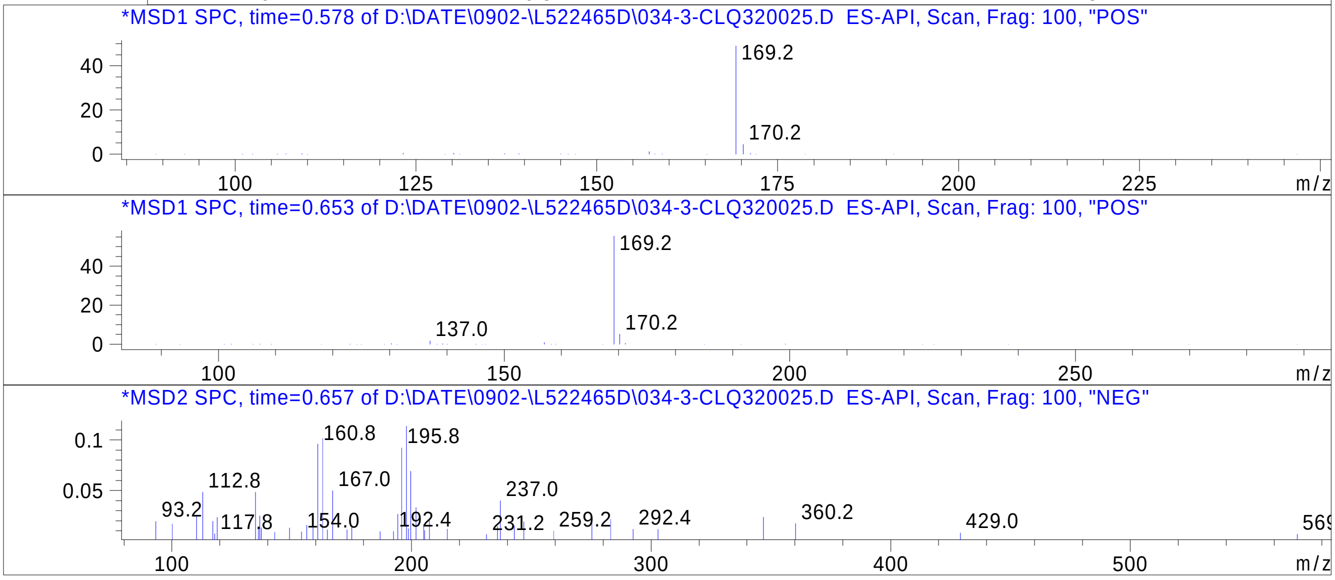

15

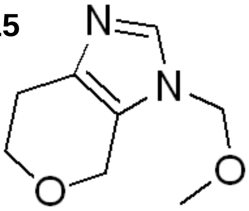

15'

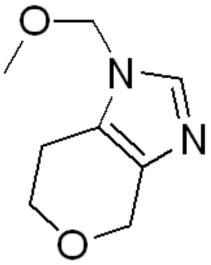

Inj.Date 9/2/2022

C:\Chem32\-> ->

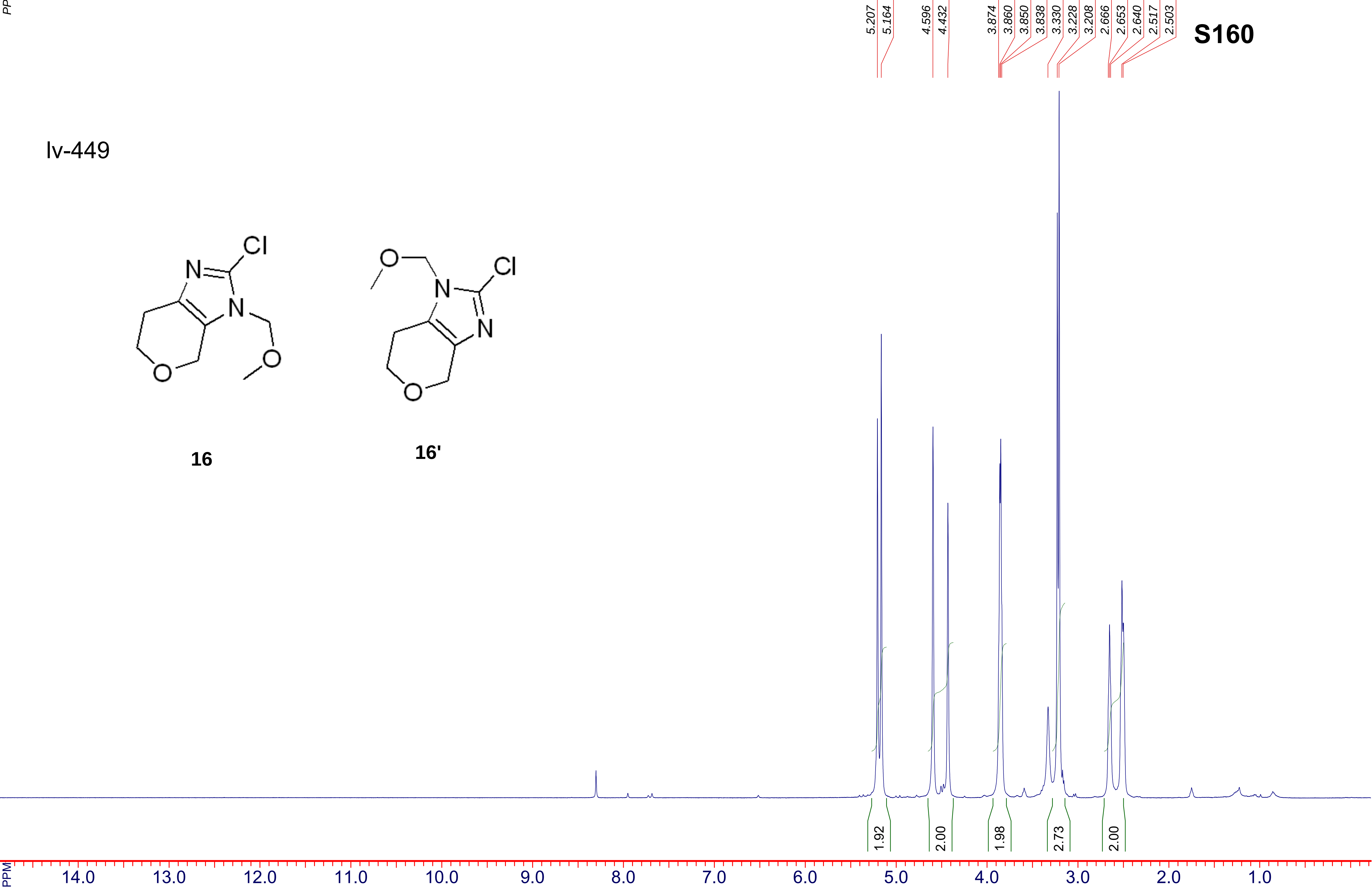

**File name: lv-449**	Operator: nmr	SF: 400.1300 MHz	NSC: 1	PW: 0.00 usec, RG: 25	SI: 32768
Date: 06-Sep-2022	Solvent: DMSO	SW: 8224 Hz	TE: 300 K	AQ: 1.99 sec, RD: 0.00 sec	

lv-449_C13

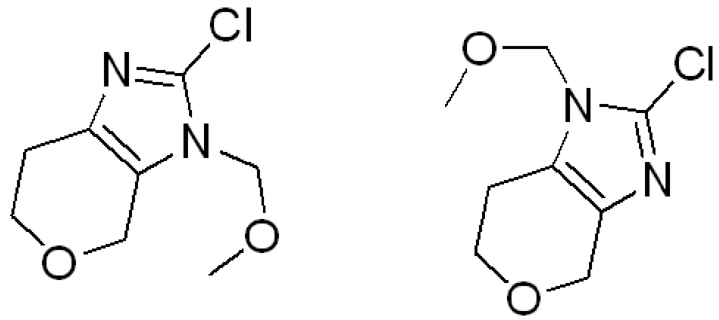

16 **16'**

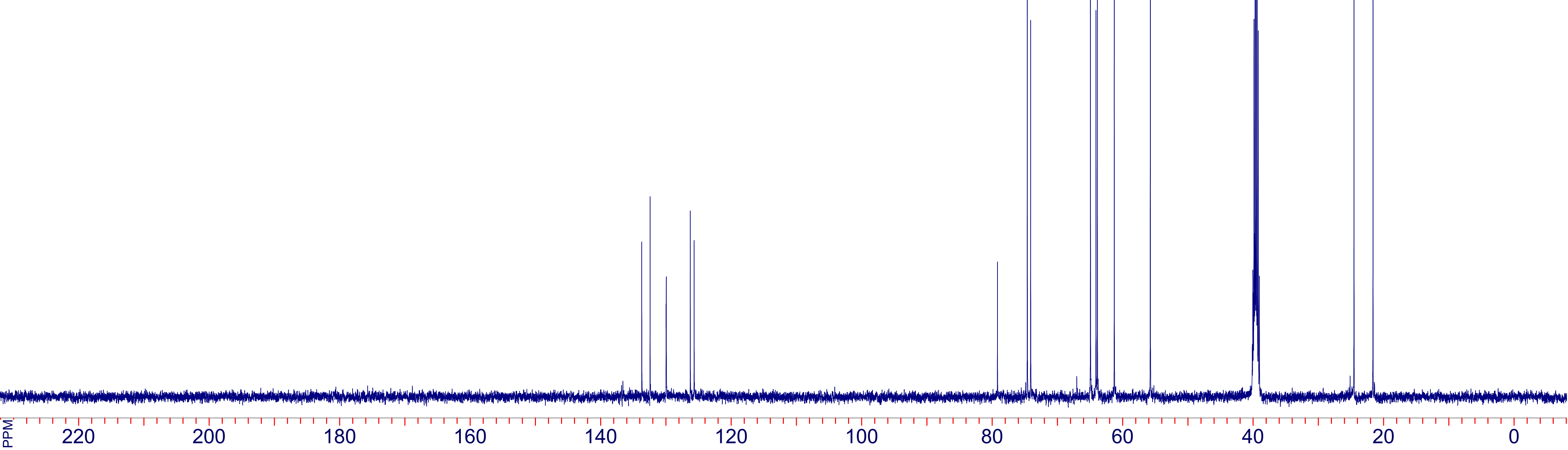

**File name: lv-449_C13**	Operator: root	SF: 125.6681 MHz	NSC: 388	PW: 0.00 usec, RG: 51200	SI: 131072
Date: 06-Sep-2022	Solvent: DMSO	SW: 32680 Hz	TE: 683 K	AQ: 0.78 sec, RD: 0.00 sec	

lv-449_C13APT

S162

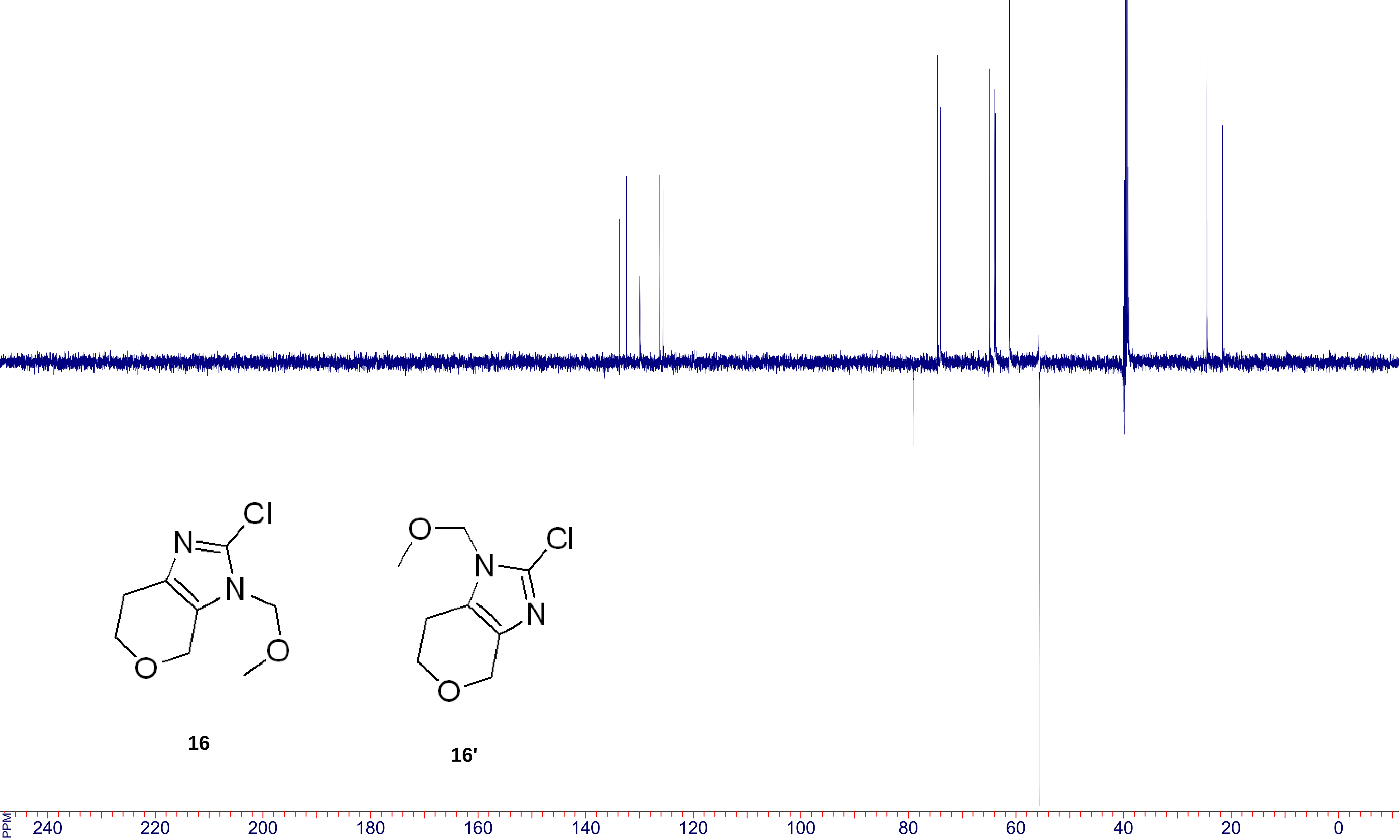

16

16'

**File name: lv-449_C13APT**	Operator: root	SF: 125.6681 MHz	NSC: 283	PW: 0.00 usec, RG: 51200	SI: 65536
Date: 06-Sep-2022	Solvent: DMSO	SW: 32680 Hz	TE: 683 K	AQ: 1.00 sec, RD: 0.00 sec	

CLQ320025

MaxPeak: 54.56%
Ret_Time: 0.582 min

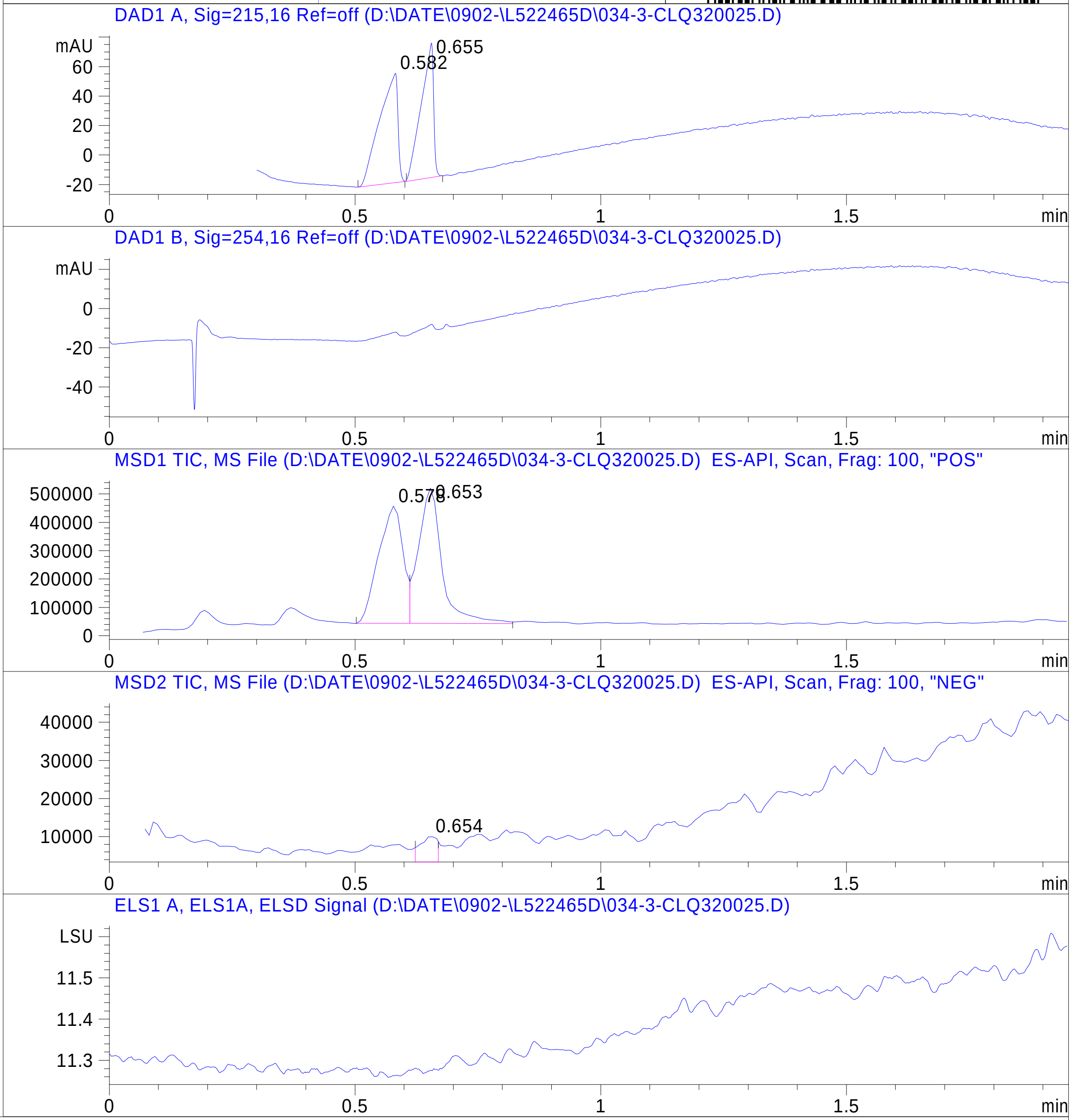

Mol Wt 0

Exact Mass

#	Time	Area%
1	0.582	54.56
2	0.655	45.44

RT 0.578

RT 0.653

RT 0.654

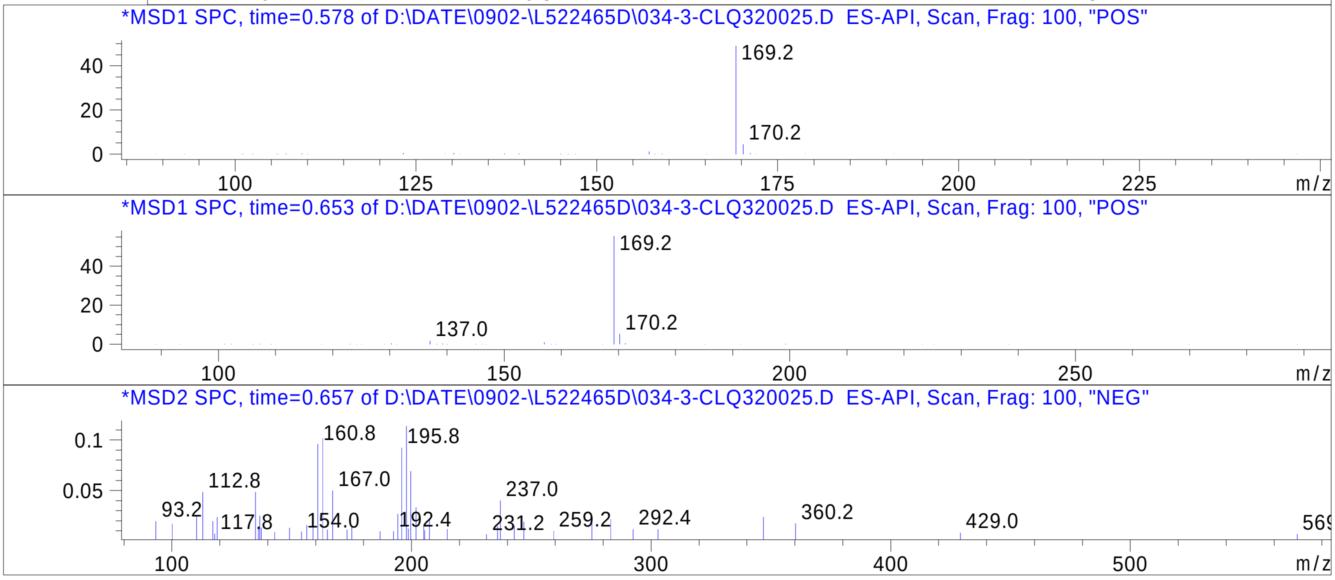

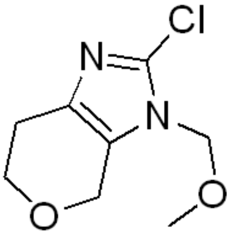

16

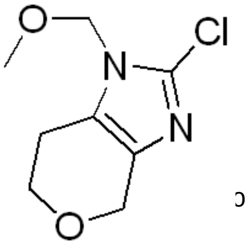

16'

Inj.Date 9/2/2022

od C:\Chem32\-> ->

R3209059

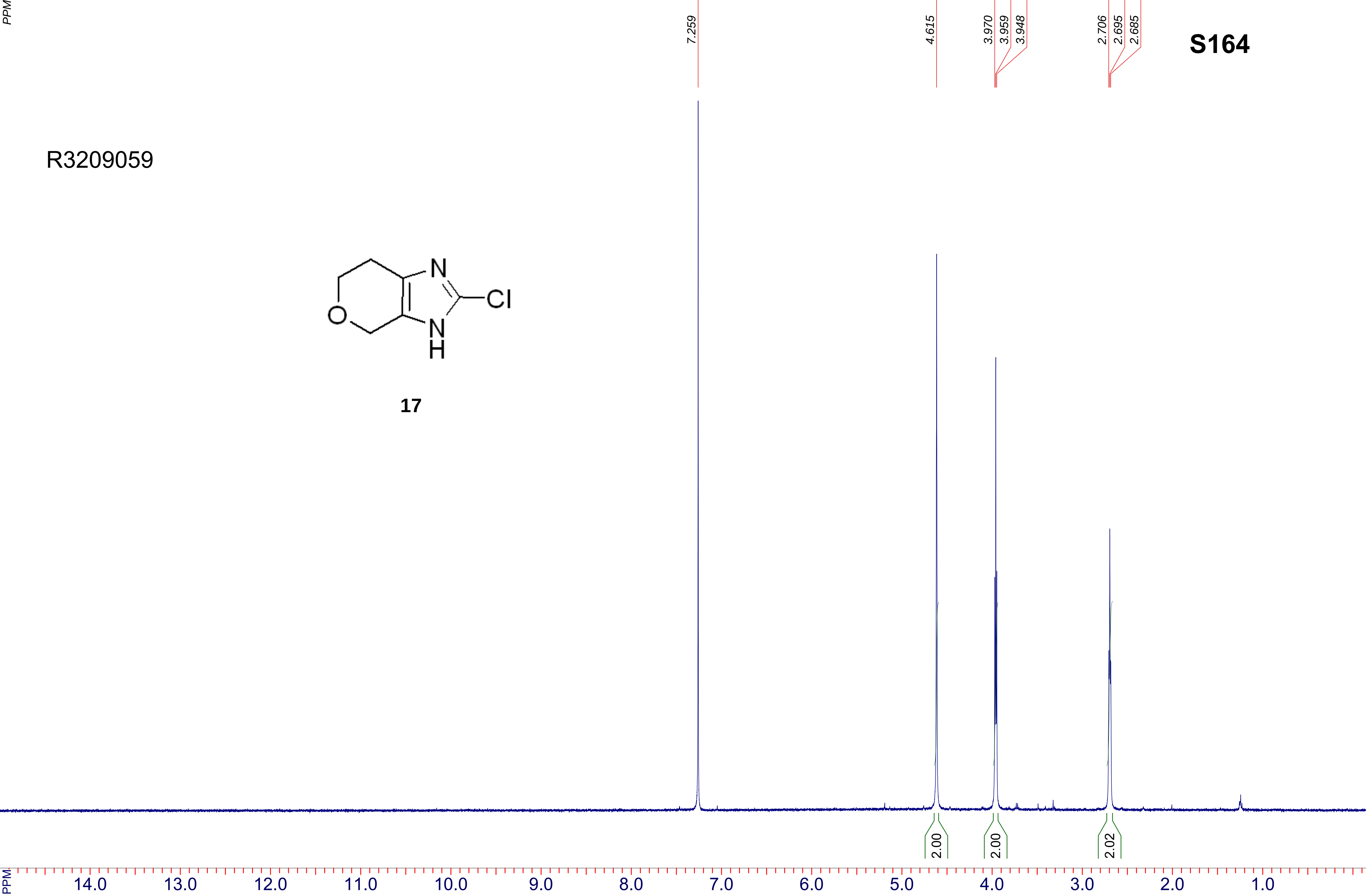
17

**File name: R3209059**	Operator:	SF: 499.8180 MHz	NSC: 0	PW: 13.60 usec, RG: 12	SI: 32768
Date: 13-Oct-2022	Solvent: cdcl3	SW: 9328 Hz	TE: 298 K	AQ: 1.72 sec, RD: 0.00 sec	

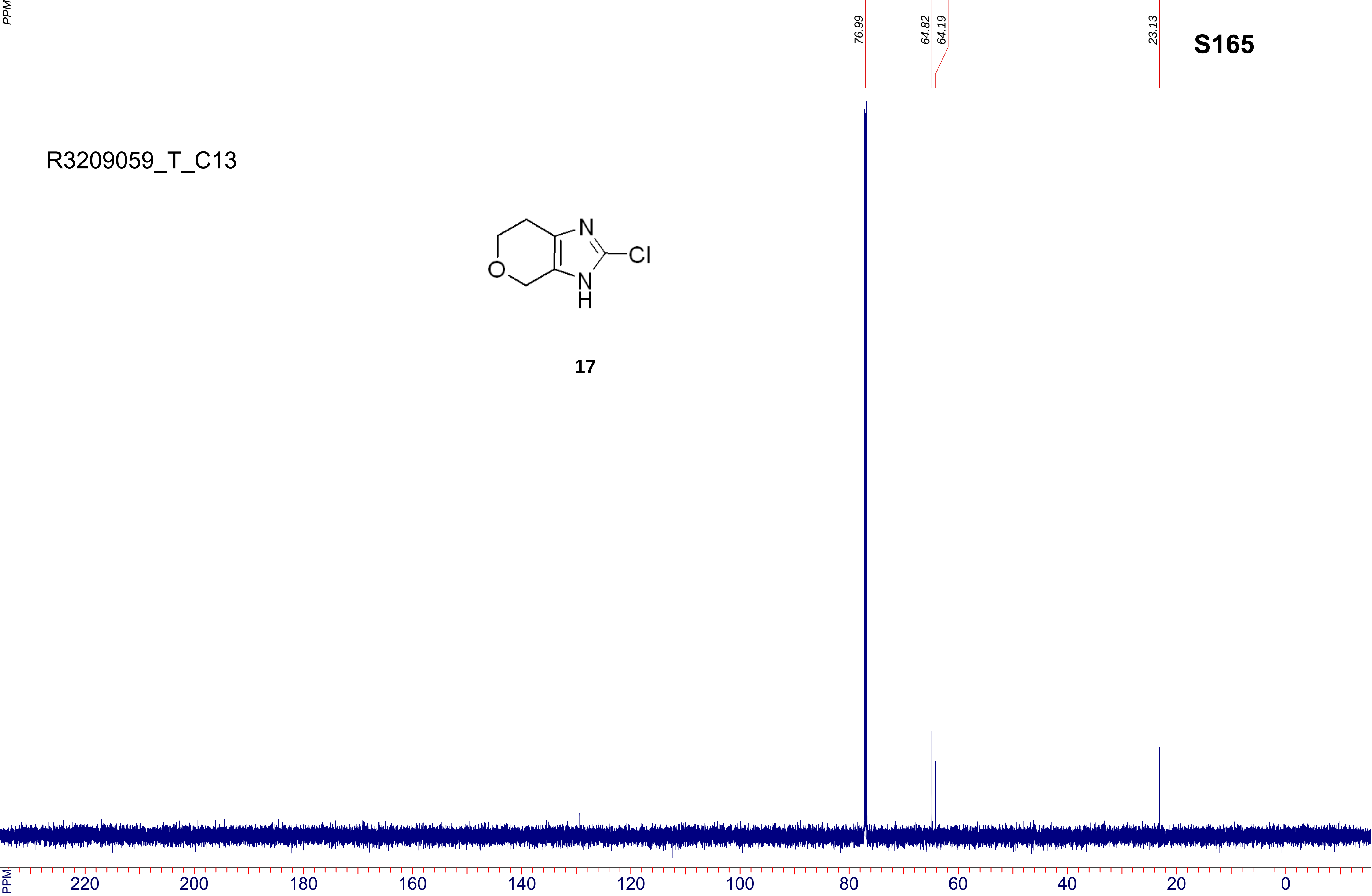

File name: R3209059_T_C1:	Operator:	SF: 150.8297 MHz	NSC: 0	PW: 3.09 usec, RG: 60	SI: 655

Library Search Report

S166

```
Data Path : D:\MassHunter\GCMS\1\data\10_14\
Data File : R3209059.D
Acq On    : 14 Oct 2022  14:52
Operator  :
Sample    : R3209059
Misc      : CH3OH
ALS Vial  : 42   Sample Multiplier: 1

Search Libraries:   C:\Database\EMPTY.L              Minimum Quality:   0

Unknown Spectrum:   Apex
Integration Events: ChemStation Integrator - autoint1.e
```

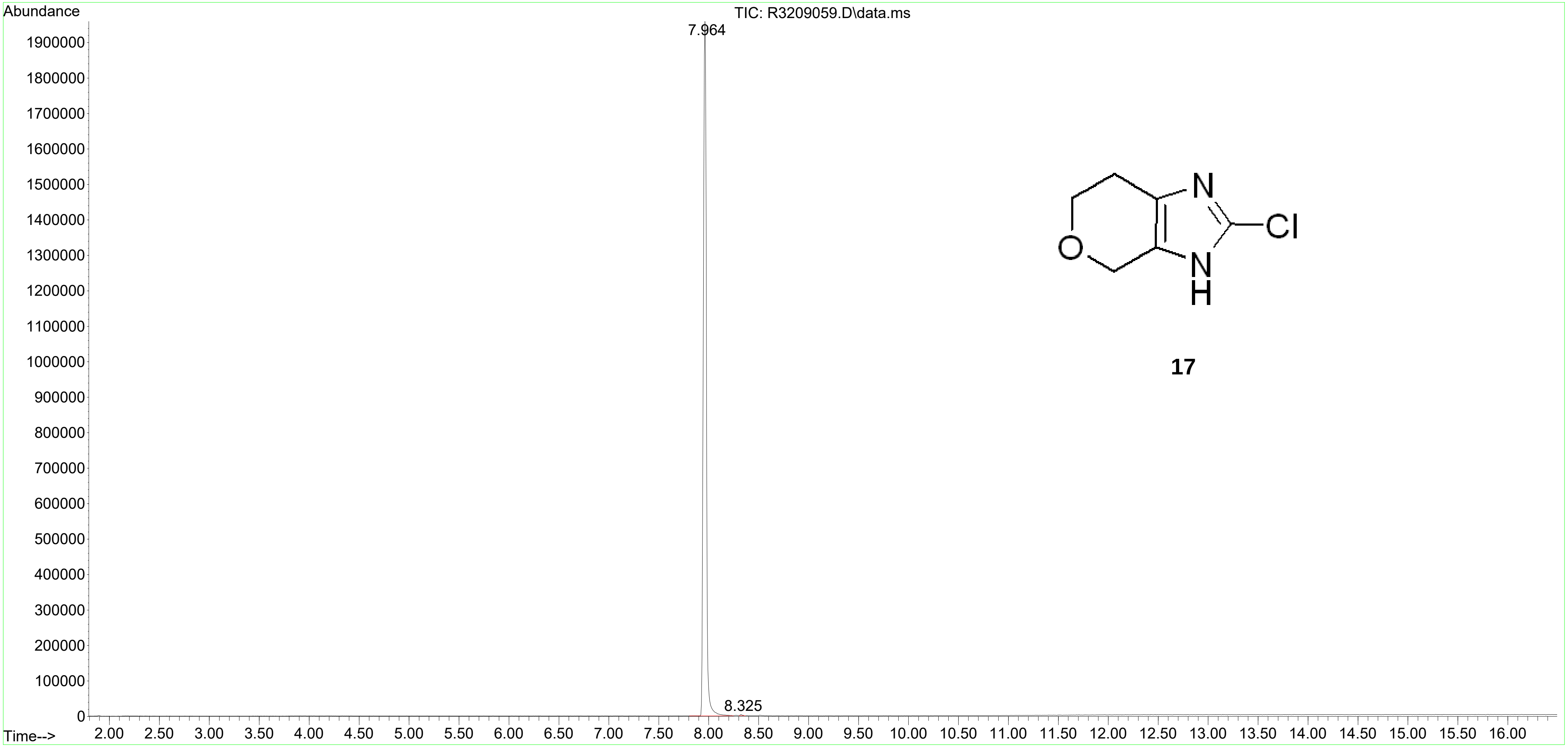

17

Unknown Spectrum based on Apex

S167

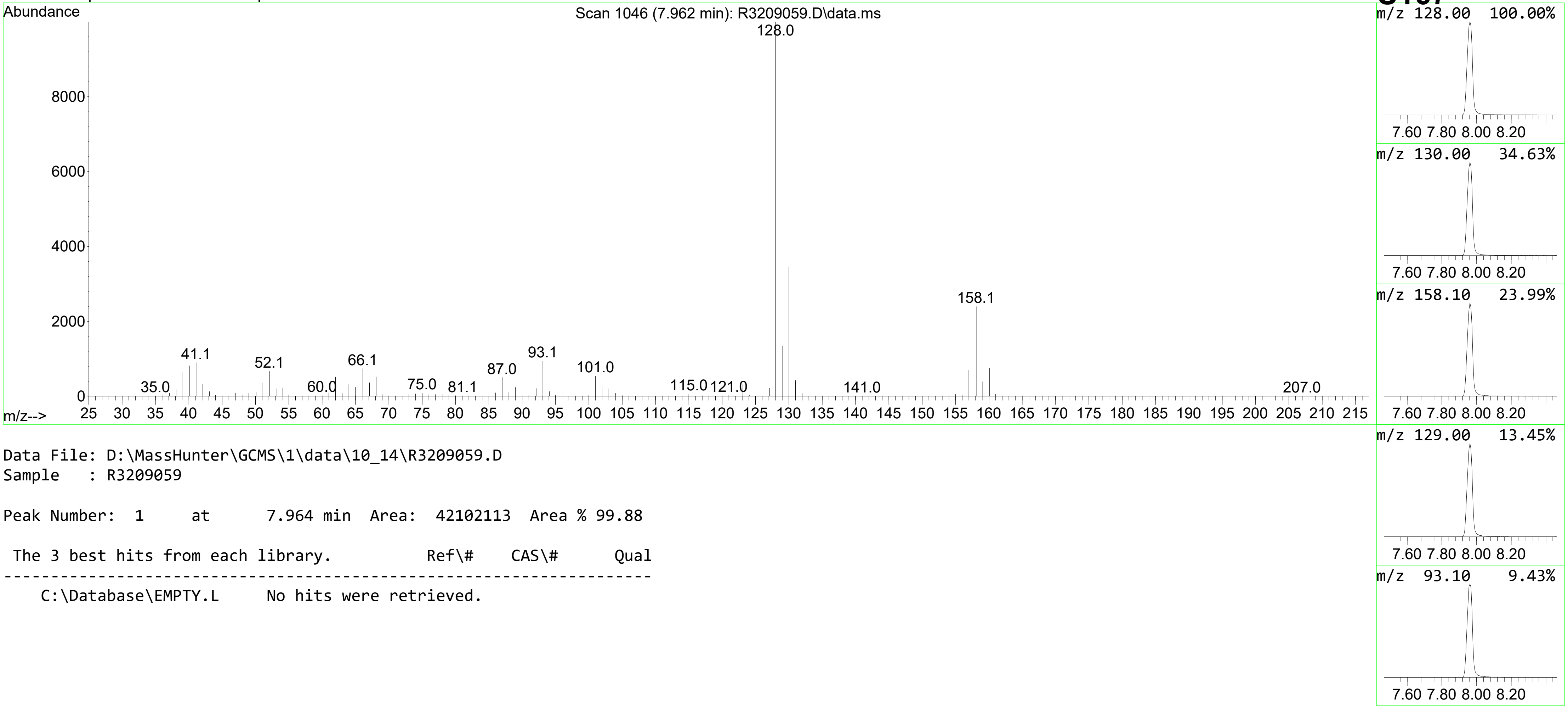

Data File: D:\MassHunter\GCMS\1\data\10_14\R3209059.D
Sample : R3209059

Peak Number: 1 at 7.964 min Area: 42102113 Area % 99.88

The 3 best hits from each library.	Ref\#	CAS\#	Qual
C:\Database\EMPTY.L No hits were retrieved.			

Unknown Spectrum based on Apex

S168

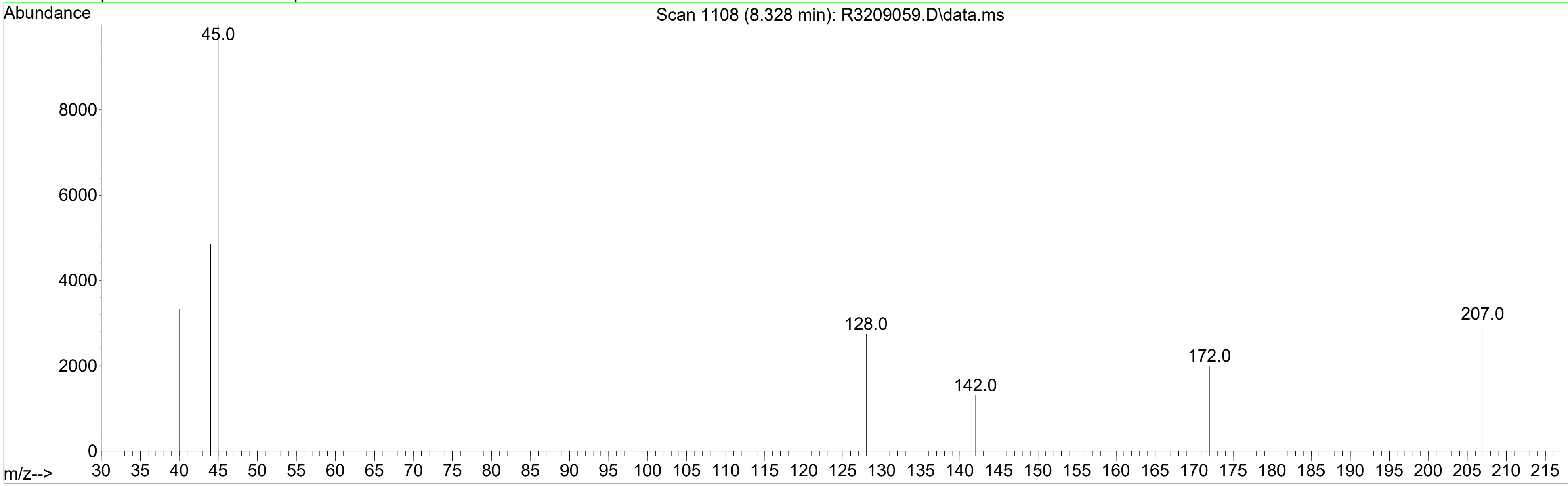

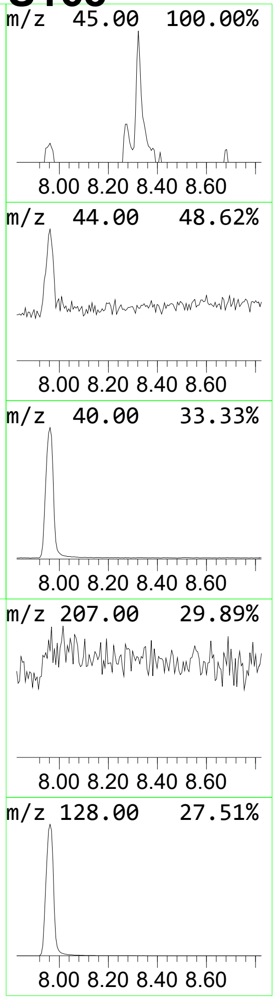

Data File: D:\MassHunter\GCMS\1\data\10_14\R3209059.D
Sample : R3209059

Peak Number: 2 at 8.325 min Area: 50677 Area % 0.12

The 3 best hits from each library. Ref\# CAS\# Qual

C:\Database\EMPTY.L No hits were retrieved.

Single-crystal X-ray diffraction study

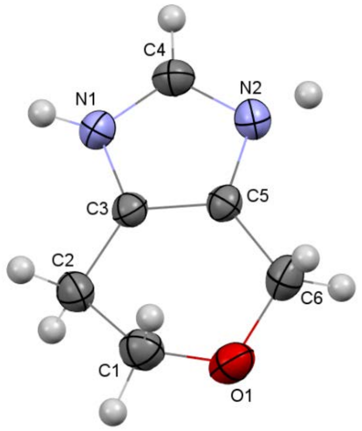

Figure S1. Molecular structure of compound **12a** according to X-ray diffraction data. Thermal ellipsoids of non-hydrogen atoms are shown at 50% probability level.

The molecular and crystal structure of compound **12a** (Fig. 1) was studied by the single crystal X-ray diffraction method. The very close values of the C5–N2/C3–N1 (1.378(2) Å and 1.377(2) Å) and N2–C4/N1–C4 (1.337(2) Å and 1.334(2) Å) bond lengths as well as disordering of the hydrogen atom between two nitrogen atoms.

The tetrahydroheterocycle adopts a half-chair conformation where the C2, C3, C5, C6 atoms lie in the plane with an accuracy of 0.003 Å and the C1 and O1 atoms deviate from this plane by 0.332(3) Å and -0.397(3) Å, respectively.

X-ray experimental part.

The colourless crystals of compound **12a** ($C_6H_8N_2O$) are monoclinic. At 173 K a = 7.9880(7), b = 5.8913(5), c = 13.2710(11) Å, β = 100.151(6)°, V = 614.75(9) Å^3, M_r = 124.14, Z = 4, space group $P2_1/n$, d_{calc}= 1.341 g/cm^3, μ(MoK_α) = 0.095 mm^{-1}, F(000) = 264. Intensities of 8076 reflections (1073 independent, R_{int}=0.0504) were measured on the Bruker APEX II diffractometer (graphite monochromated MoK_α radiation, CCD detector, φ- and ω-scaning, $2\Theta_{max}$ = 50°). The structure was solved by direct method using SHELXTL package [1]. Positions of the hydrogen atoms were located from electron density difference maps and refined using “riding” model with U_{iso} = 1.2U_{eq} of the carrier atom. Full-matrix least-squares refinement against F^2 in anisotropic approximation for non-hydrogen atoms using 1073 reflections was converged to wR_2 = 0.1007 (R_1 = 0.0401 for 827 reflections with F>4σ(F), S = 1.047). The final atomic coordinates, and crystallographic data for molecule **12a** have been deposited to with the

Cambridge Crystallographic Data Centre, 12 Union Road, CB2 1EZ, UK (fax: +44-1223-336033; e-mail: deposit@ccdc.cam.ac.uk) and are available on request quoting the deposition numbers CCDC 2369787).

Literature

1. Sheldrick G.M. // Acta Crystallogr., Sect. A, 2008, A64, p.112-122.
